# Supplementary material for: Genomic reconstruction of the SARS-CoV-2 epidemic in England
Source: Nature. 2021 Oct 14;600(7889):506–11. doi: 10.1038/s41586-021-04069-y (PMC8674138; doi:10.1038/s41586-021-04069-y)
Supplement: Supplementary file 1 — Supplementary Notes 1–3. [file 41586_2021_4069_MOESM1_ESM.pdf]

nature portfolio

https://doi.org/10.1038/s41586-021-04069-y

Supplementary information

Genomic reconstruction of the SARS-CoV-2 epidemic in England

In the format provided by the authors and unedited

Genomic reconstruction of the SARS-CoV-2 epidemic in England

Harald S. Vöhringer[1,2], Theo Sanderson[3,4], Matthew Sinnot[3,4], Nicola De Maio[1], Thuy Nguyen[3], Richard Goater[3], Frank Schwach[3,5], Ian Harrison[5], Joel Hellewell[6], Cristina Ariani[3], Sonia Gonçalves[3], David Jackson[3], Ian Johnston[3], Alexander W. Jung[1], Callum Saint[3], John Sillitoe[3], Maria Suciu[3], Nick Goldman[1], Jasmina Panovska-Griffiths[2,7], The Wellcome Sanger Institute Covid-19 Surveillance Team[8], The COVID-19 Genomics UK (COG-UK) Consortium[9], Ewan Birney[1], Erik Volz[10], Sebastian Funk[6], Dominic Kwiatkowski[3], Meera Chand[5,11], Inigo Martincorena[3], Jeffrey C. Barrett[3,*], and Moritz Gerstung[1,12,*]

[1] *European Molecular Biology Laboratory, European Bioinformatics Institute (EMBL-EBI), Hinxton, UK.*
[2] *Joint Biosecurity Center JBC*
[3] *Wellcome Sanger Institute, Hinxton, UK*
[4] *The Francis Crick Institute, London, UK*
[5] *Public Health England PHE*
[6] *London School of Hygiene & Tropical Medicine, London, UK*
[7] *The Big Data Institute, Nuffield Department of Medicine, University of Oxford, UK*
[8] *https://www.sanger.ac.uk/project/wellcome-sanger-institute-covid-19-surveillance-team/*
[9] *Full list of consortium names and affiliations are in the Appendix*
[10] *MRC Centre for Global Infectious Disease Analysis, Jameel Institute for Disease and Emergency Analytics, Imperial College London, London, UK*
[11] *Guy's and St Thomas' NHS Foundation Trust, UK*
[12] *Division for AI in Oncology, German Cancer Research Centre dkfz, Heidelberg, Germany*
[*] *to whom correspondence should be addressed*

Abstract

The evolution of the SARS-CoV-2 pandemic continuously produces new variants, which warrant timely epidemiological characterisation. Here we use the dense genomic surveillance generated by the COVID-19 Genomics UK Consortium to reconstruct the dynamics of 71 different lineages in each of 315 English local authorities between September 2020 and June 2021. This analysis reveals a series of sub-epidemics that peaked in the early autumn of 2020, followed by a jump in transmissibility of the B.1.1.7/Alpha lineage. Alpha grew when other lineages declined during the second national lockdown and regionally tiered restrictions between November and December 2020. A third more stringent national lockdown suppressed Alpha and eliminated nearly all other lineages in early 2021. However, a series of variants (mostly containing the spike E484K mutation) defied these trends and persisted at moderately increasing proportions. Accounting for sustained introductions, however, indicates that their transmissibility is unlikely to have exceeded that of Alpha. Finally, B.1.617.2/Delta was repeatedly introduced to England and grew rapidly in the early summer of 2021, constituting approximately 98% of sampled SARS-CoV-2 genomes on June 26.

Contents

1 Supplementary Note 1 3

2 Supplementary Note 2 11

3 Supplementary Note 3 92

1 Supplementary Note 1

Supplementary Note 1 Fig. 1: **Observed proportions of sequenced SARS-Cov2 lineages in 315 LTLA in England.** The titles in each tile refer to LTLA look up codes (Supplementary Note 3) and colours indicate major SARS-CoV-2 lineages (blue: B.1, purple: B.1.1, green: B.1.177, red: B.1.1.7 (Alpha), orange: other VOC/VUI, brown: B.1.617.2 (Delta) and grey: Other (A & B)).

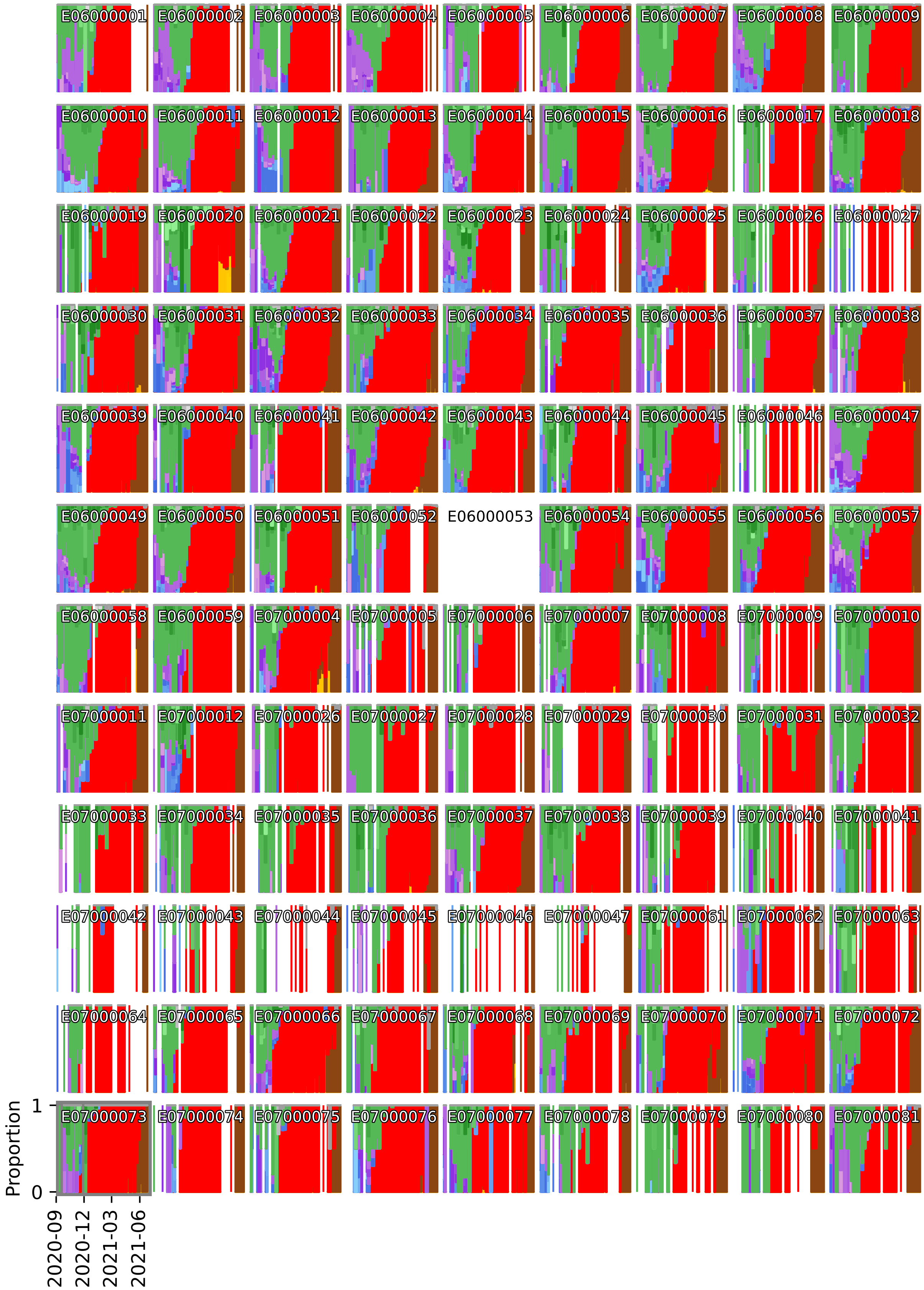
E06000001
E06000002
E06000003
E06000004
E06000005
E06000006
E06000007
E06000008
E06000009
E06000010
E06000011
E06000012
E06000013
E06000014
E06000015
E06000016
E06000017
E06000018
E06000019
E06000020
E06000021
E06000022
E06000023
E06000024
E06000025
E06000026
E06000027
E06000030
E06000031
E06000032
E06000033
E06000034
E06000035
E06000036
E06000037
E06000038
E06000039
E06000040
E06000041
E06000042
E06000043
E06000044
E06000045
E06000046
E06000047
E06000049
E06000050
E06000051
E06000052
E06000053
E06000054
E06000055
E06000056
E06000057
E06000058
E06000059
E07000004
E07000005
E07000006
E07000007
E07000008
E07000009
E07000010
E07000011
E07000012
E07000026
E07000027
E07000028
E07000029
E07000030
E07000031
E07000032
E07000033
E07000034
E07000035
E07000036
E07000037
E07000038
E07000039
E07000040
E07000041
E07000042
E07000043
E07000044
E07000045
E07000046
E07000047
E07000061
E07000062
E07000063
E07000064
E07000065
E07000066
E07000067
E07000068
E07000069
E07000070
E07000071
E07000072
E07000073
E07000074
E07000075
E07000076
E07000077
E07000078
E07000079
E07000080
E07000081
Proportion
1
0
2020-09
2020-12
2021-03
2021-06

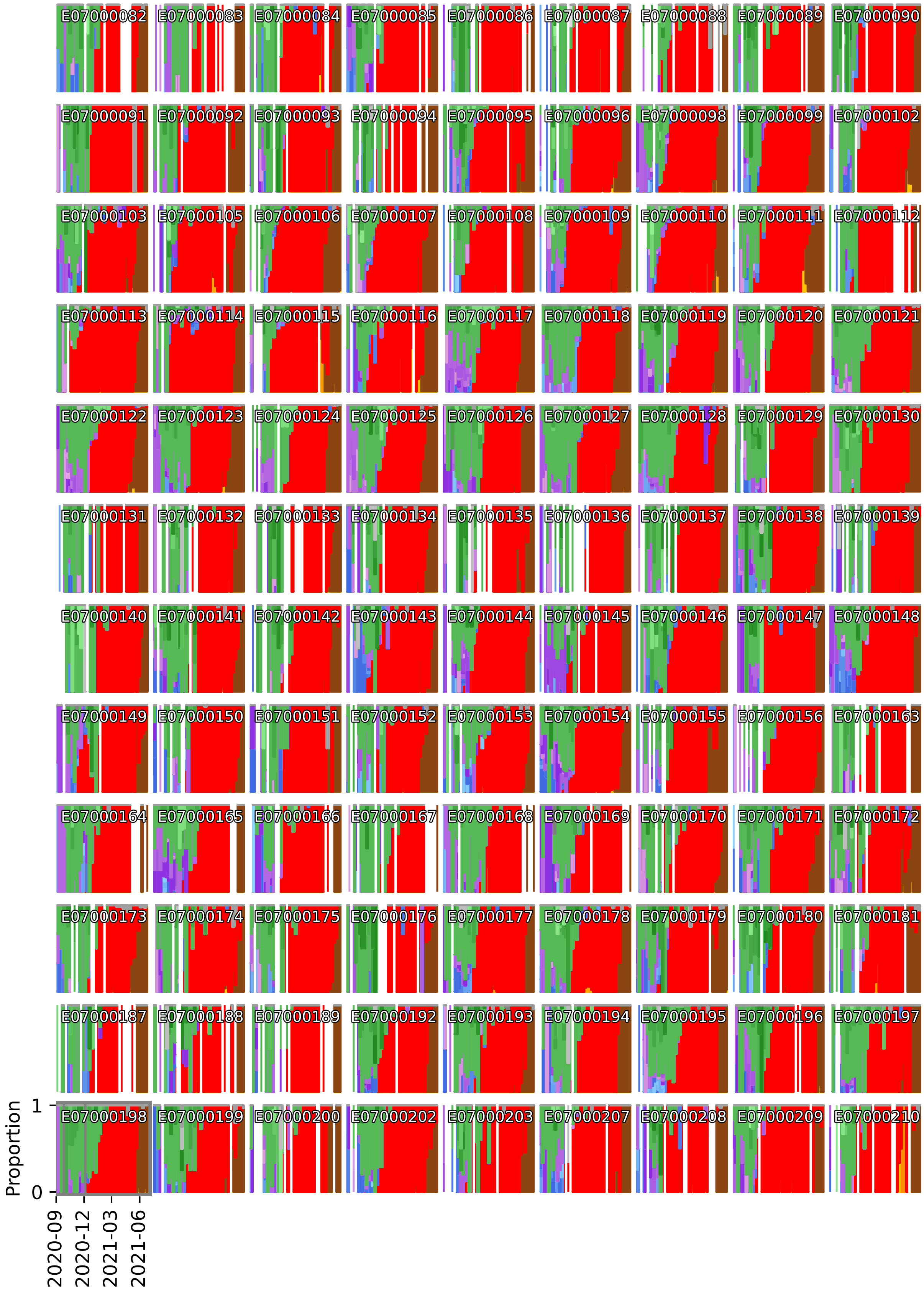
E07000082
E07000083
E07000084
E07000085
E07000086
E07000087
E07000088
E07000089
E07000090
E07000091
E07000092
E07000093
E07000094
E07000095
E07000096
E07000098
E07000099
E07000102
E07000103
E07000105
E07000106
E07000107
E07000108
E07000109
E07000110
E07000111
E07000112
E07000113
E07000114
E07000115
E07000116
E07000117
E07000118
E07000119
E07000120
E07000121
E07000122
E07000123
E07000124
E07000125
E07000126
E07000127
E07000128
E07000129
E07000130
E07000131
E07000132
E07000133
E07000134
E07000135
E07000136
E07000137
E07000138
E07000139
E07000140
E07000141
E07000142
E07000143
E07000144
E07000145
E07000146
E07000147
E07000148
E07000149
E07000150
E07000151
E07000152
E07000153
E07000154
E07000155
E07000156
E07000163
E07000164
E07000165
E07000166
E07000167
E07000168
E07000169
E07000170
E07000171
E07000172
E07000173
E07000174
E07000175
E07000176
E07000177
E07000178
E07000179
E07000180
E07000181
E07000187
E07000188
E07000189
E07000192
E07000193
E07000194
E07000195
E07000196
E07000197
E07000198
E07000199
E07000200
E07000202
E07000203
E07000207
E07000208
E07000209
E07000210
Proportion
1
0
2020-09
2020-12
2021-03
2021-06

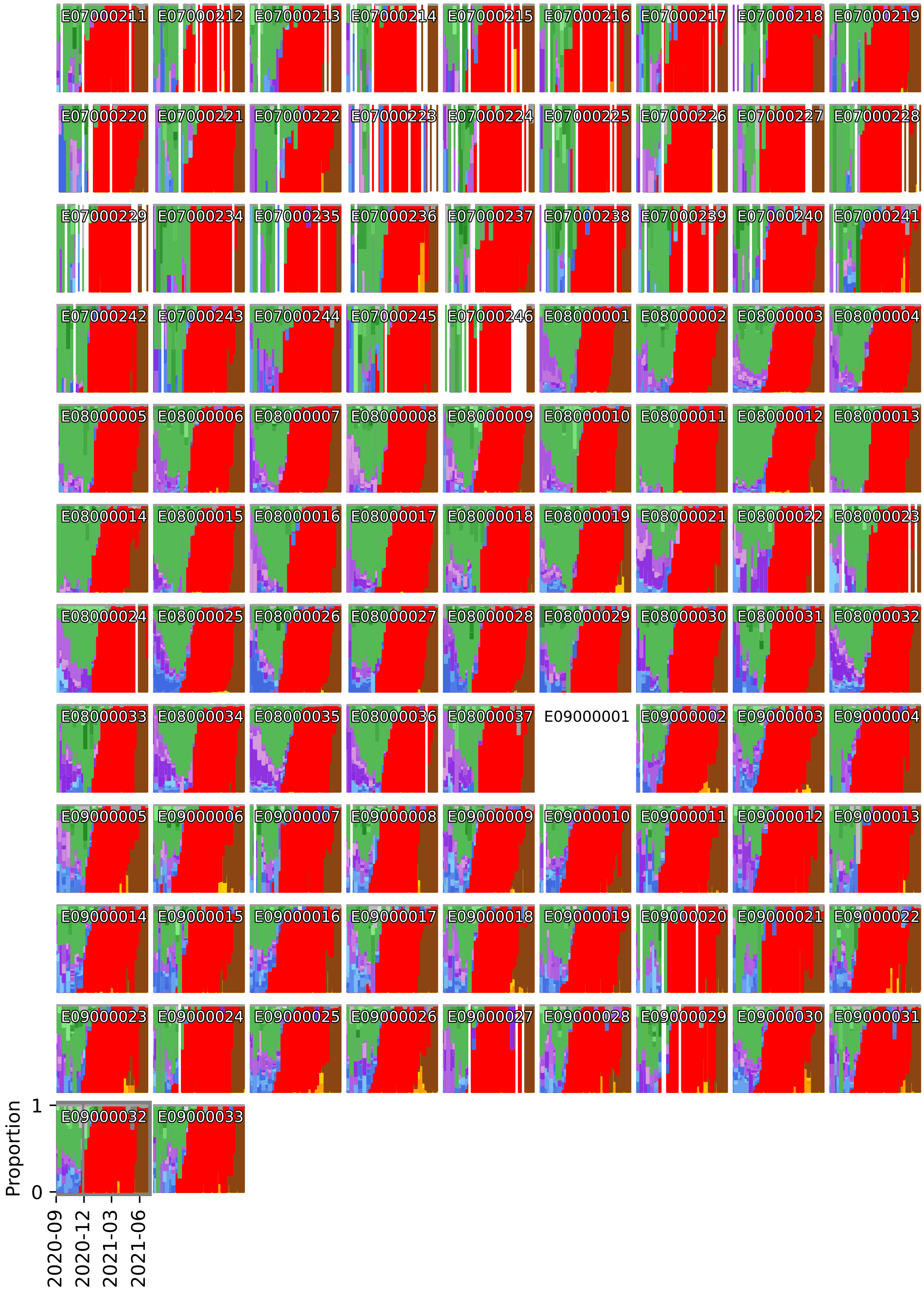
E07000211
E07000212
E07000213
E07000214
E07000215
E07000216
E07000217
E07000218
E07000219
E07000220
E07000221
E07000222
E07000223
E07000224
E07000225
E07000226
E07000227
E07000228
E07000229
E07000234
E07000235
E07000236
E07000237
E07000238
E07000239
E07000240
E07000241
E07000242
E07000243
E07000244
E07000245
E07000246
E08000001
E08000002
E08000003
E08000004
E08000005
E08000006
E08000007
E08000008
E08000009
E08000010
E08000011
E08000012
E08000013
E08000014
E08000015
E08000016
E08000017
E08000018
E08000019
E08000021
E08000022
E08000023
E08000024
E08000025
E08000026
E08000027
E08000028
E08000029
E08000030
E08000031
E08000032
E08000033
E08000034
E08000035
E08000036
E08000037
E09000001
E09000002
E09000003
E09000004
E09000005
E09000006
E09000007
E09000008
E09000009
E09000010
E09000011
E09000012
E09000013
E09000014
E09000015
E09000016
E09000017
E09000018
E09000019
E09000020
E09000021
E09000022
E09000023
E09000024
E09000025
E09000026
E09000027
E09000028
E09000029
E09000030
E09000031
E09000032
E09000033
Proportion
1
0
2020-09
2020-12
2021-03
2021-06

Supplementary Note 1 Fig. 2: **Modelled proportions of sequenced SARS-Cov2 lineages in 315 LTLA in England.** The titles in each tile refer to LTLA look up codes (Supplementary Note 3) and colours indicate major SARS-CoV-2 lineages (blue: B.1, purple: B.1.1, green: B.1.177, red: B.1.1.7 (Alpha), orange: other VOC/VUI, brown: B.1.617.2 (Delta) and grey: Other (A & B)).

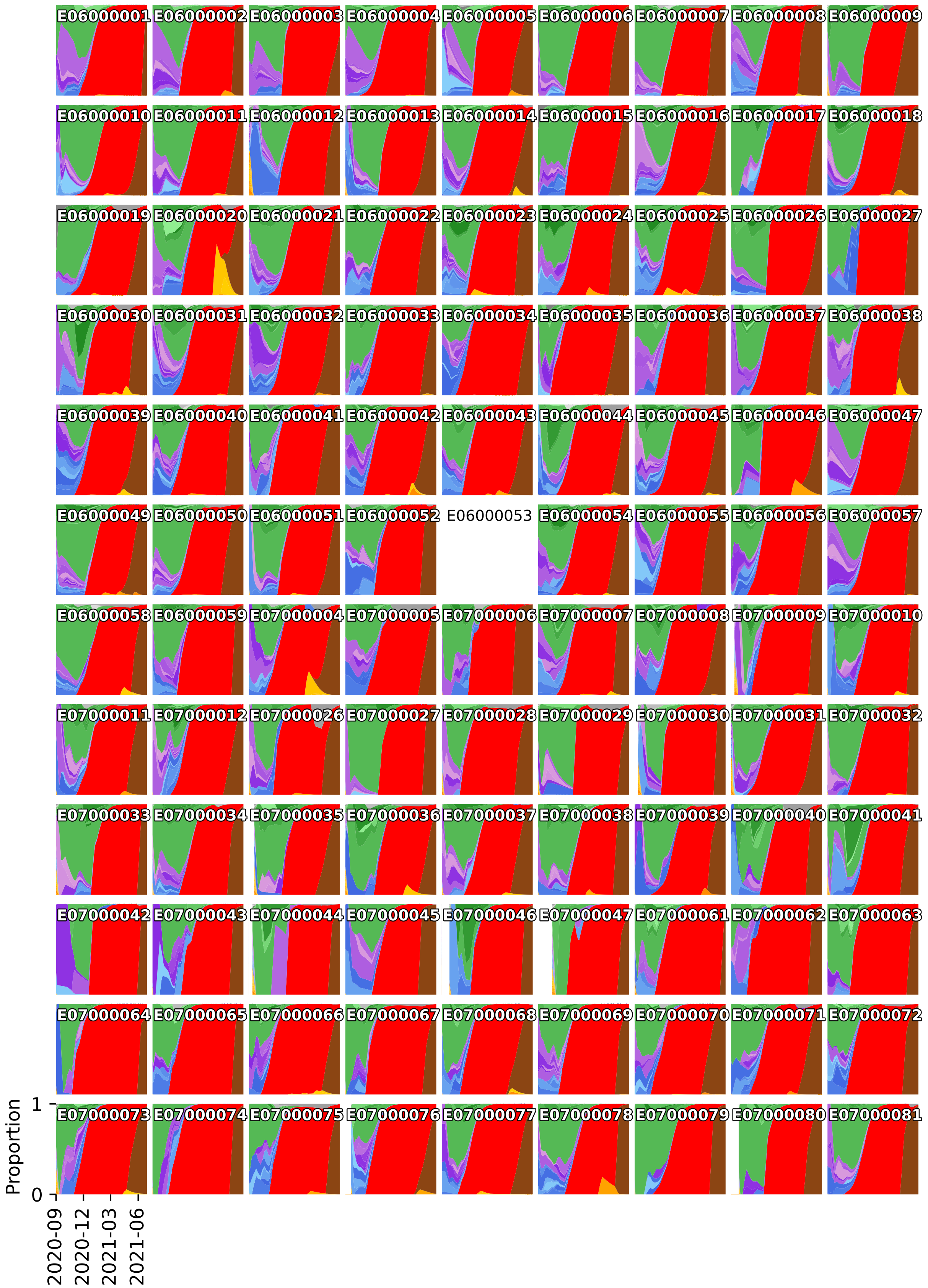
E06000001
E06000002
E06000003
E06000004
E06000005
E06000006
E06000007
E06000008
E06000009
E06000010
E06000011
E06000012
E06000013
E06000014
E06000015
E06000016
E06000017
E06000018
E06000019
E06000020
E06000021
E06000022
E06000023
E06000024
E06000025
E06000026
E06000027
E06000030
E06000031
E06000032
E06000033
E06000034
E06000035
E06000036
E06000037
E06000038
E06000039
E06000040
E06000041
E06000042
E06000043
E06000044
E06000045
E06000046
E06000047
E06000049
E06000050
E06000051
E06000052
E06000053
E06000054
E06000055
E06000056
E06000057
E06000058
E06000059
E07000004
E07000005
E07000006
E07000007
E07000008
E07000009
E07000010
E07000011
E07000012
E07000026
E07000027
E07000028
E07000029
E07000030
E07000031
E07000032
E07000033
E07000034
E07000035
E07000036
E07000037
E07000038
E07000039
E07000040
E07000041
E07000042
E07000043
E07000044
E07000045
E07000046
E07000047
E07000061
E07000062
E07000063
E07000064
E07000065
E07000066
E07000067
E07000068
E07000069
E07000070
E07000071
E07000072
E07000073
E07000074
E07000075
E07000076
E07000077
E07000078
E07000079
E07000080
E07000081
Proportion
1
0
2020-09
2020-12
2021-03
2021-06

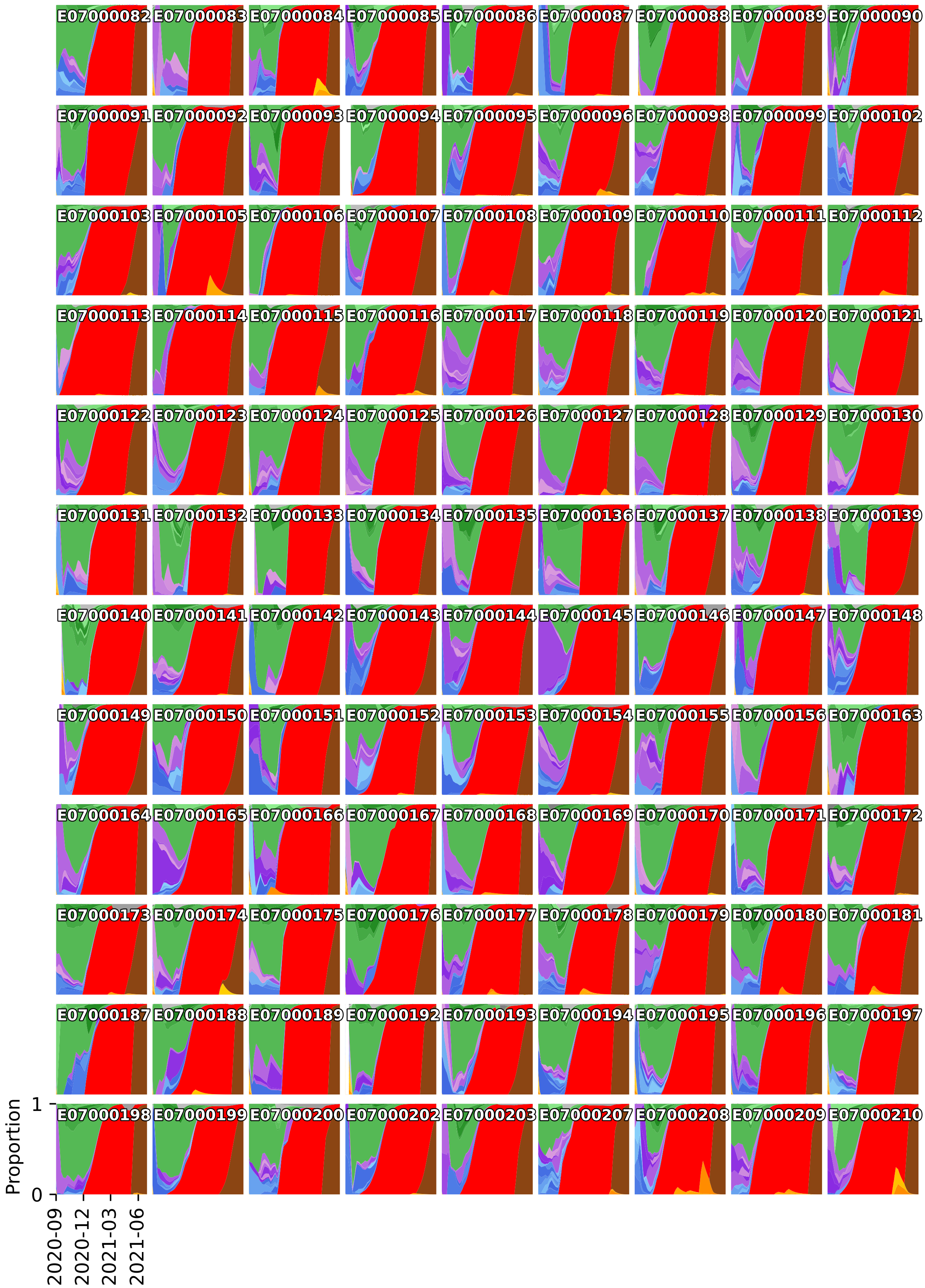

E07000082
E07000083
E07000084
E07000085
E07000086
E07000087
E07000088
E07000089
E07000090
E07000091
E07000092
E07000093
E07000094
E07000095
E07000096
E07000098
E07000099
E07000102
E07000103
E07000105
E07000106
E07000107
E07000108
E07000109
E07000110
E07000111
E07000112
E07000113
E07000114
E07000115
E07000116
E07000117
E07000118
E07000119
E07000120
E07000121
E07000122
E07000123
E07000124
E07000125
E07000126
E07000127
E07000128
E07000129
E07000130
E07000131
E07000132
E07000133
E07000134
E07000135
E07000136
E07000137
E07000138
E07000139
E07000140
E07000141
E07000142
E07000143
E07000144
E07000145
E07000146
E07000147
E07000148
E07000149
E07000150
E07000151
E07000152
E07000153
E07000154
E07000155
E07000156
E07000163
E07000164
E07000165
E07000166
E07000167
E07000168
E07000169
E07000170
E07000171
E07000172
E07000173
E07000174
E07000175
E07000176
E07000177
E07000178
E07000179
E07000180
E07000181
E07000187
E07000188
E07000189
E07000192
E07000193
E07000194
E07000195
E07000196
E07000197
E07000198
E07000199
E07000200
E07000202
E07000203
E07000207
E07000208
E07000209
E07000210
Proportion
1
0
2020-09
2020-12
2021-03
2021-06

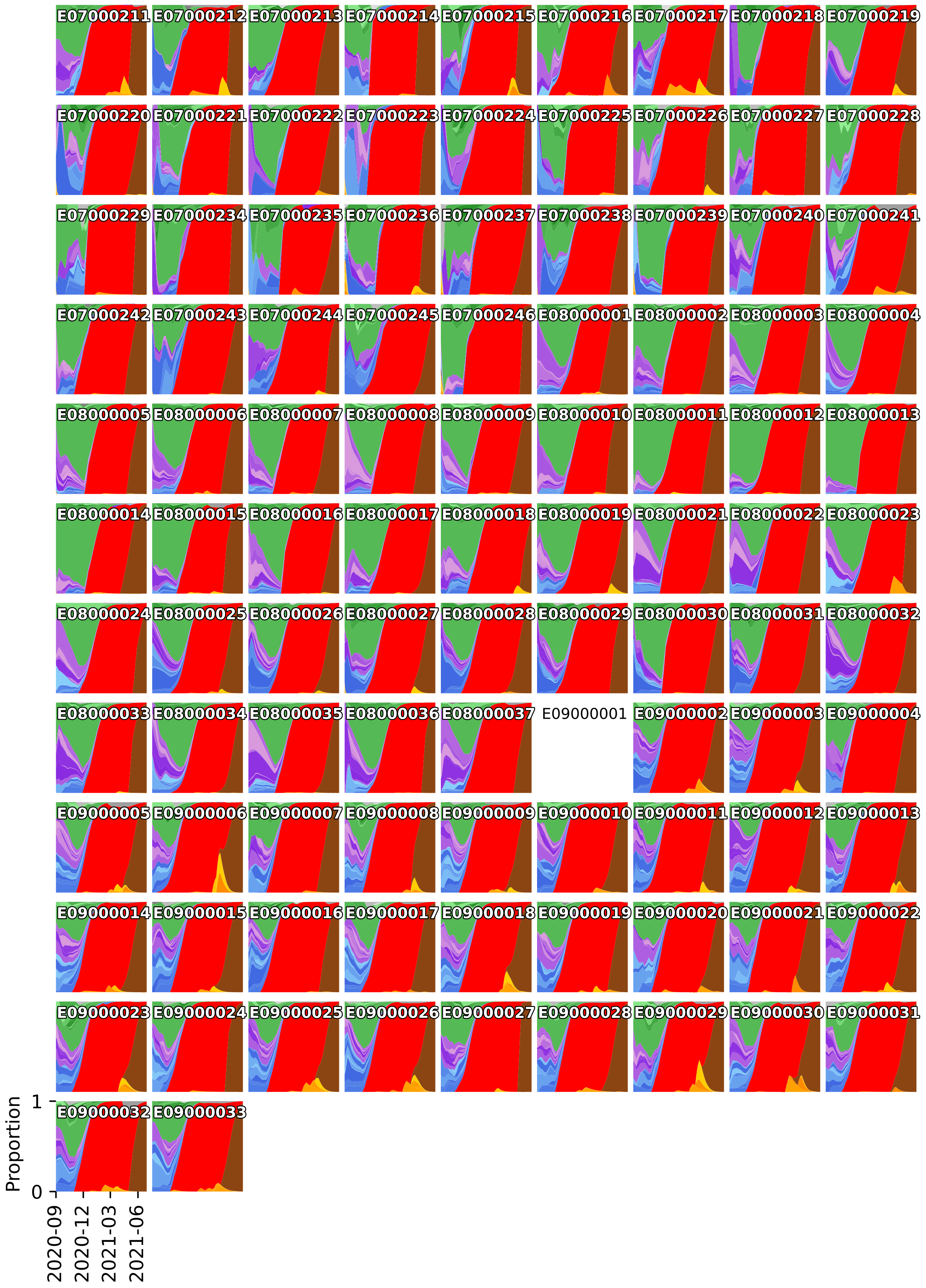
E07000211
E07000212
E07000213
E07000214
E07000215
E07000216
E07000217
E07000218
E07000219
E07000220
E07000221
E07000222
E07000223
E07000224
E07000225
E07000226
E07000227
E07000228
E07000229
E07000234
E07000235
E07000236
E07000237
E07000238
E07000239
E07000240
E07000241
E07000242
E07000243
E07000244
E07000245
E07000246
E08000001
E08000002
E08000003
E08000004
E08000005
E08000006
E08000007
E08000008
E08000009
E08000010
E08000011
E08000012
E08000013
E08000014
E08000015
E08000016
E08000017
E08000018
E08000019
E08000021
E08000022
E08000023
E08000024
E08000025
E08000026
E08000027
E08000028
E08000029
E08000030
E08000031
E08000032
E08000033
E08000034
E08000035
E08000036
E08000037
E09000001
E09000002
E09000003
E09000004
E09000005
E09000006
E09000007
E09000008
E09000009
E09000010
E09000011
E09000012
E09000013
E09000014
E09000015
E09000016
E09000017
E09000018
E09000019
E09000020
E09000021
E09000022
E09000023
E09000024
E09000025
E09000026
E09000027
E09000028
E09000029
E09000030
E09000031
E09000032
E09000033
Proportion
1
0
2020-09
2020-12
2021-03
2021-06

2 Supplementary Note 2

Supplementary Note 2 Fig. 1: **Spatiotemporal model of 71 SARS-CoV-2 lineages in 315 English LTLAs between September 2020 and June 2021.** Upper panel: Total and relative lineage-specific incidence of B.1.177, B.1.1.7 (Alpha), B.1.617.2 (Delta) and prevalent VUI/VOC (if present). Middle panel: Modelled proportions of B.1.177, B.1.1.7 (Alpha), B.1.617.2 (Delta) and prevalent VUI/VOC (if present). Bottom panel: Local lineage-specific R_t values for B.1.177, B.1.1.7 (Alpha), B.1.617.2 (Delta) and prevalent VUI/VOC (if present) and the average R_t value (growth per 5.1d) of all other lineages in the same periods. In a small number of LTLAs (Redcar and Cleveland, Southampton, County Durham, Cheshire West and Chester, Cornwall, Bournemouth, Erewash, Stockport) we noted very large R_t values at around the time of the introduction of the delta variant. These are more likely reflections of stochastic introduction events at times of very low incidence, rather than sustained transmission.

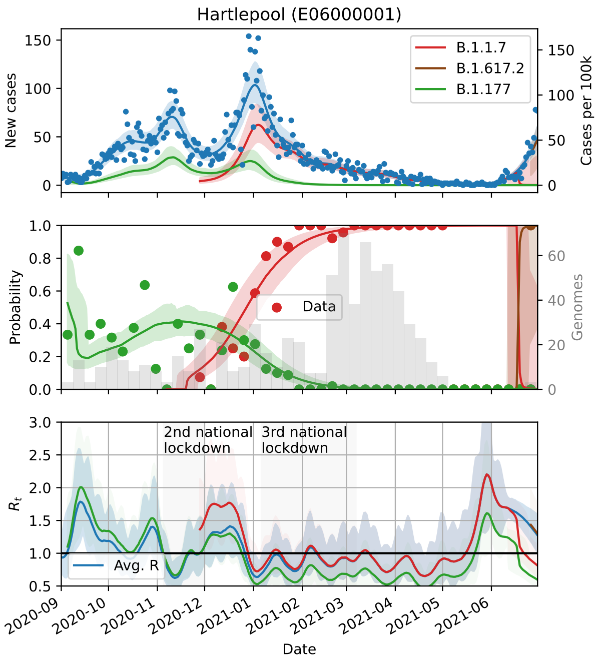
Hartlepool (E06000001)
New cases
Cases per 100k
B.1.1.7
B.1.617.2
B.1.177
Probability
Genomes
Data
R_t
2nd national lockdown
3rd national lockdown
Avg. R
Date

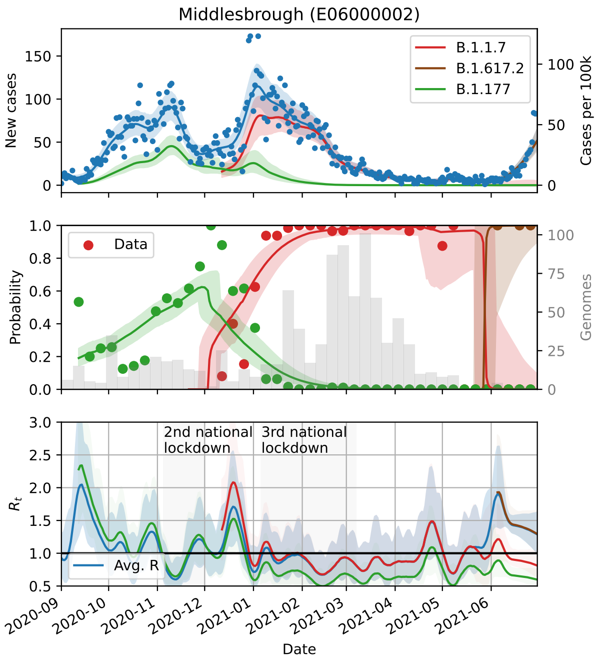
Middlesbrough (E06000002)
New cases
Cases per 100k
B.1.1.7
B.1.617.2
B.1.177
Probability
Genomes
Data
R_t
2nd national lockdown
3rd national lockdown
Avg. R
Date

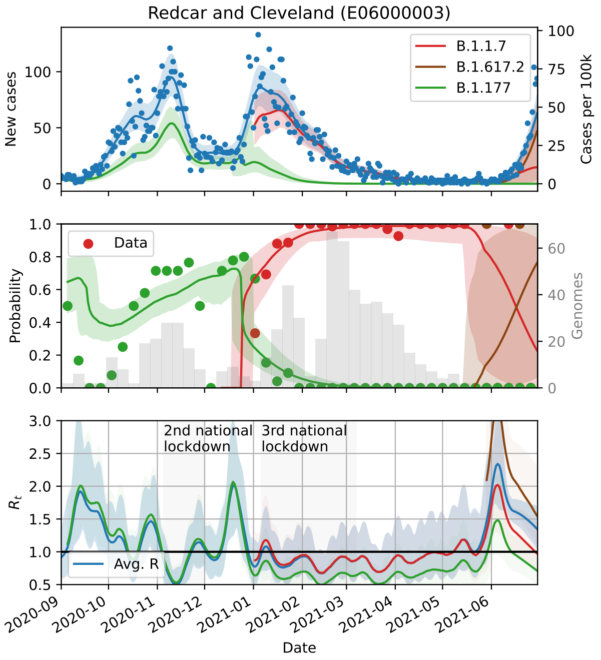
Redcar and Cleveland (E06000003)
New cases
Cases per 100k
B.1.1.7
B.1.617.2
B.1.177
Probability
Genomes
Data
R_t
2nd national lockdown
3rd national lockdown
Avg. R
Date

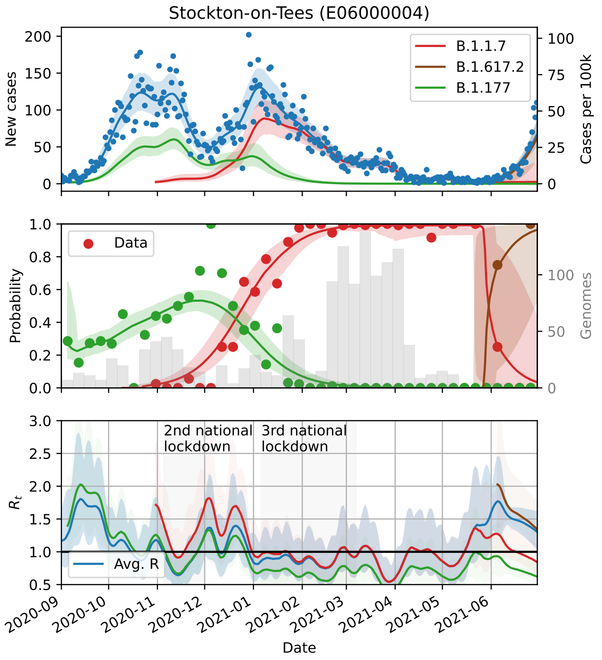
Stockton-on-Tees (E06000004)
New cases
Cases per 100k
B.1.1.7
B.1.617.2
B.1.177
Probability
Genomes
Data
R_t
2nd national lockdown
3rd national lockdown
Avg. R
Date

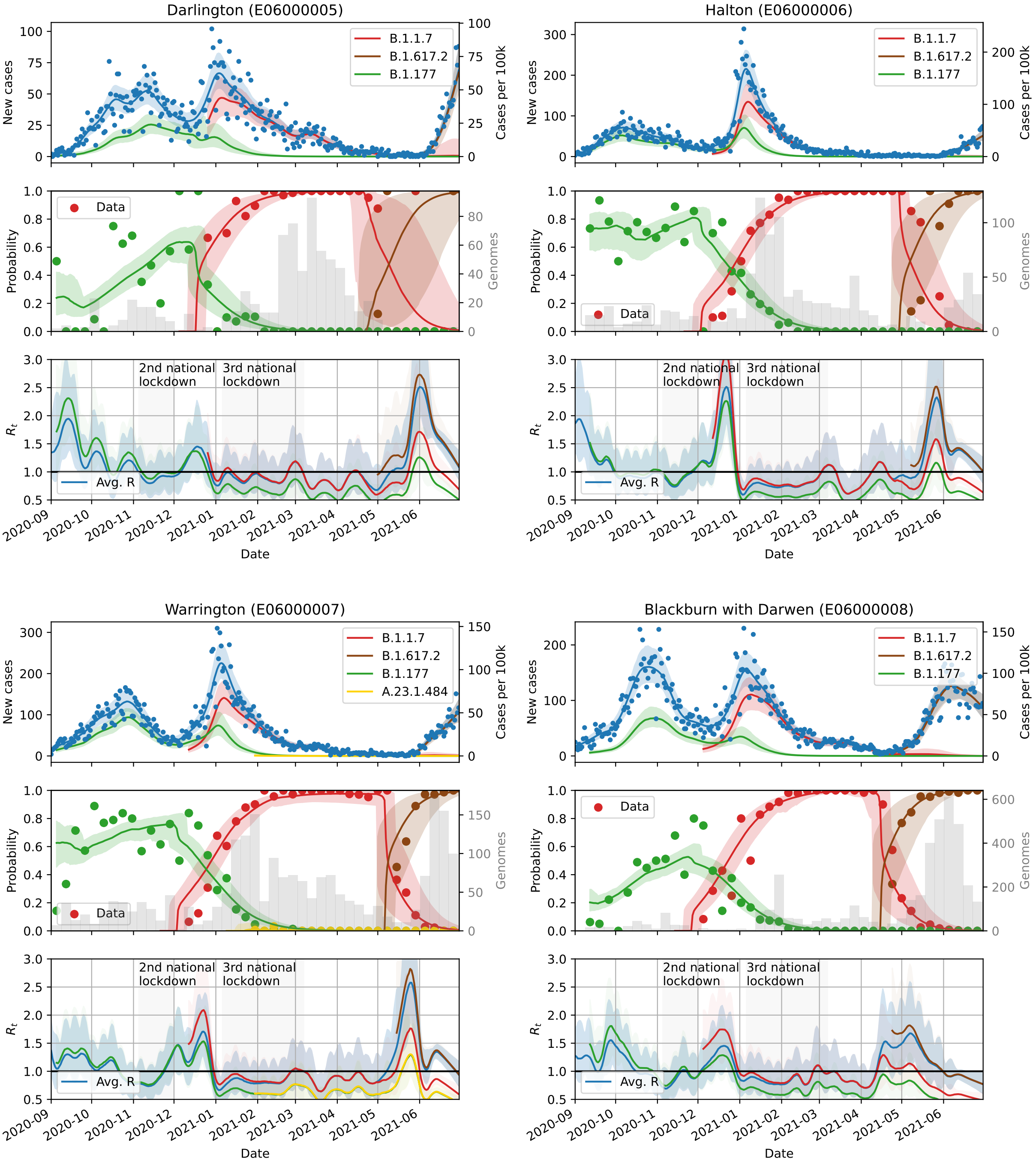

Darlington (E06000005)
Halton (E06000006)
Warrington (E06000007)
Blackburn with Darwen (E06000008)
B.1.1.7
B.1.617.2
B.1.177
A.23.1.484
New cases
Cases per 100k
Probability
Genomes
Data
R_t
Avg. R
2nd national lockdown
3rd national lockdown
Date

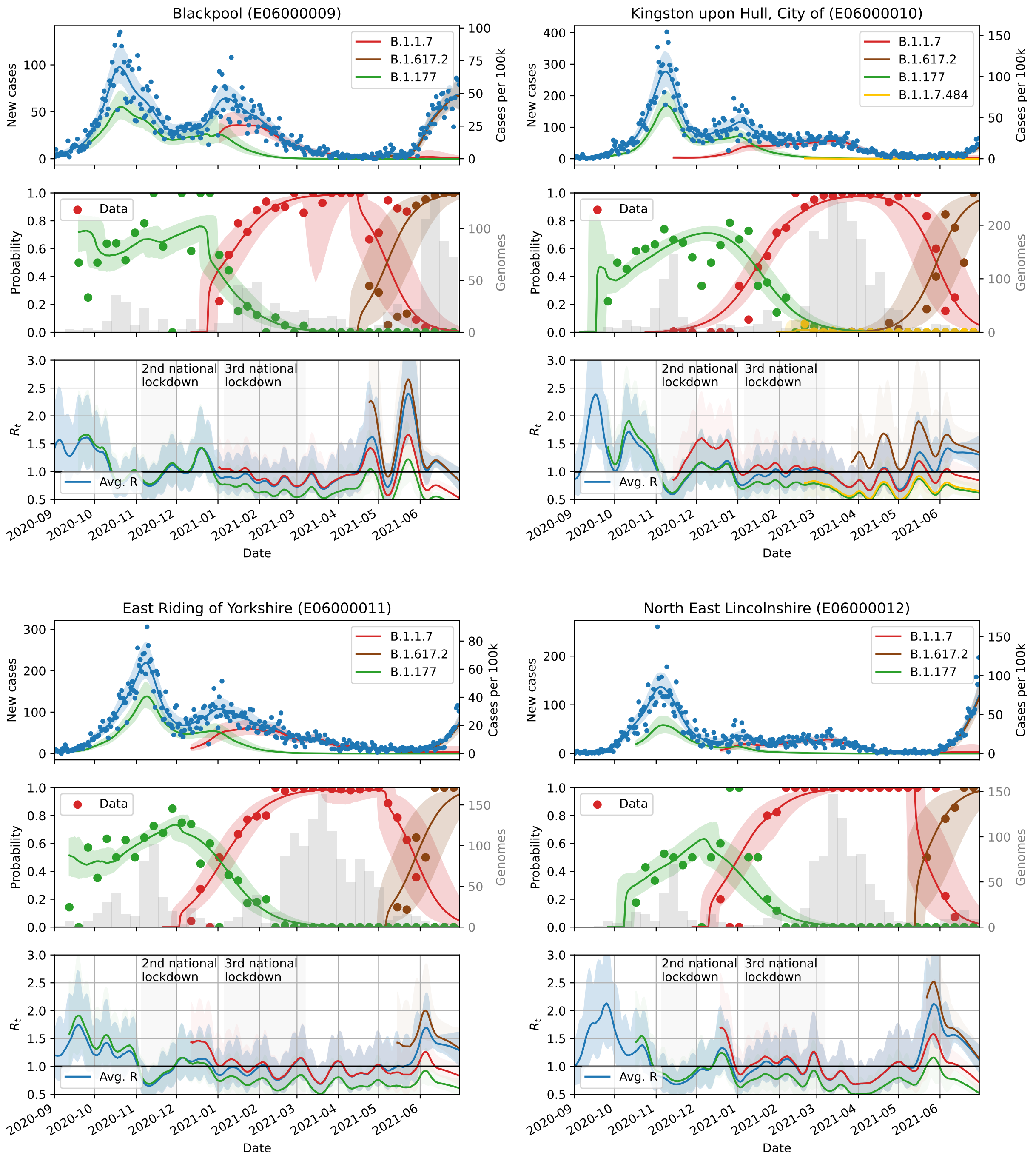

Blackpool (E06000009)
Kingston upon Hull, City of (E06000010)
East Riding of Yorkshire (E06000011)
North East Lincolnshire (E06000012)
New cases
Cases per 100k
B.1.1.7
B.1.617.2
B.1.177
B.1.1.7.484
Data
Probability
Genomes
R_t
Avg. R
2nd national lockdown
3rd national lockdown
Date
2020-09
2020-10
2020-11
2020-12
2021-01
2021-02
2021-03
2021-04
2021-05
2021-06

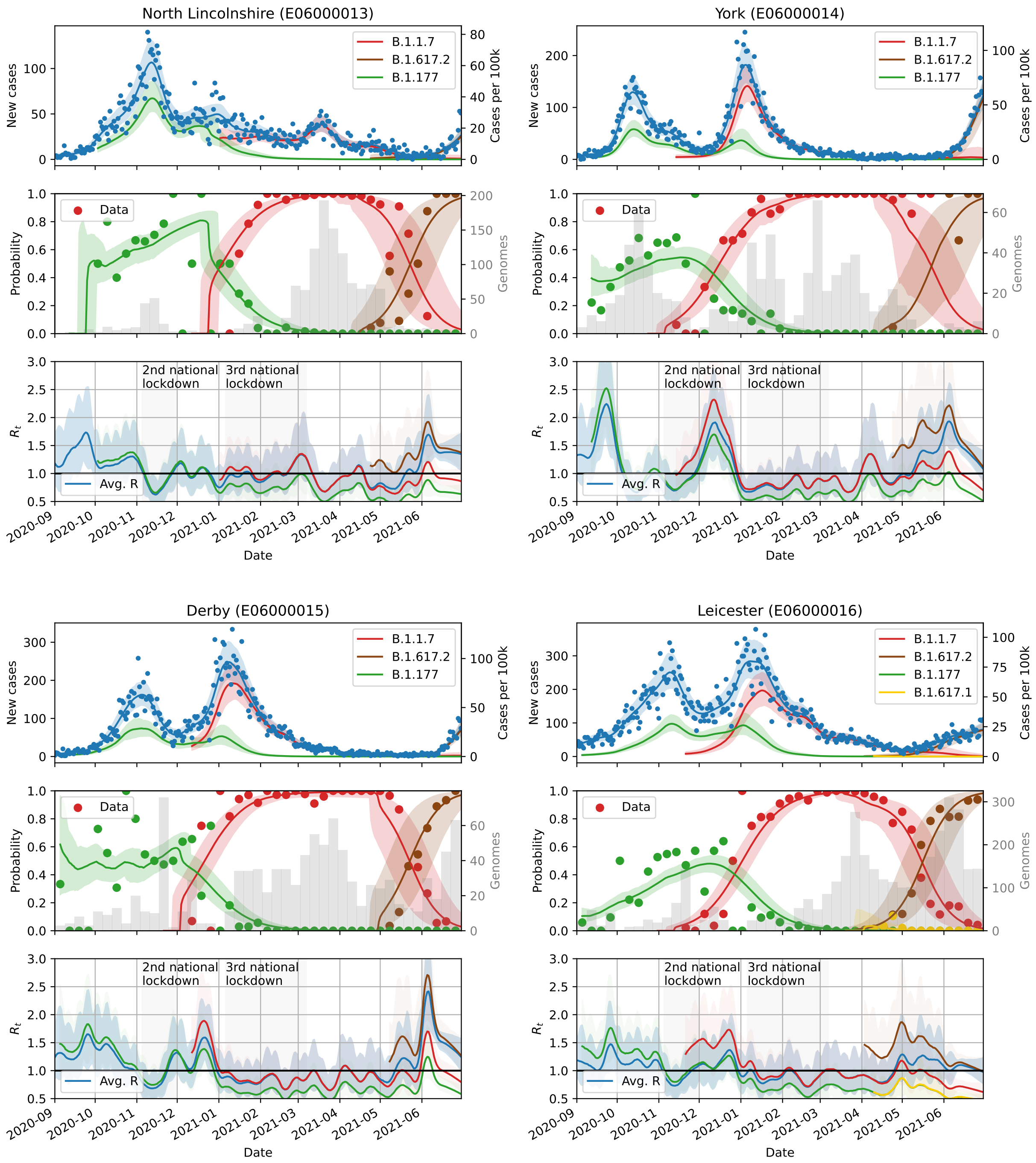
North Lincolnshire (E06000013)
York (E06000014)
Derby (E06000015)
Leicester (E06000016)
B.1.1.7
B.1.617.2
B.1.177
B.1.617.1
New cases
Cases per 100k
Data
Probability
Genomes
2nd national lockdown
3rd national lockdown
Avg. R
R_t
Date

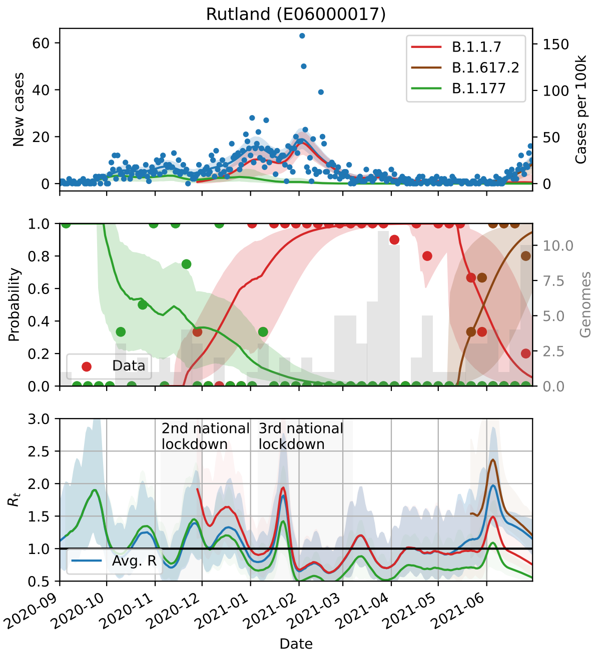
Rutland (E06000017)
B.1.1.7
B.1.617.2
B.1.177
New cases
Cases per 100k
Data
Probability
Genomes
2nd national lockdown
3rd national lockdown
Avg. R
Date

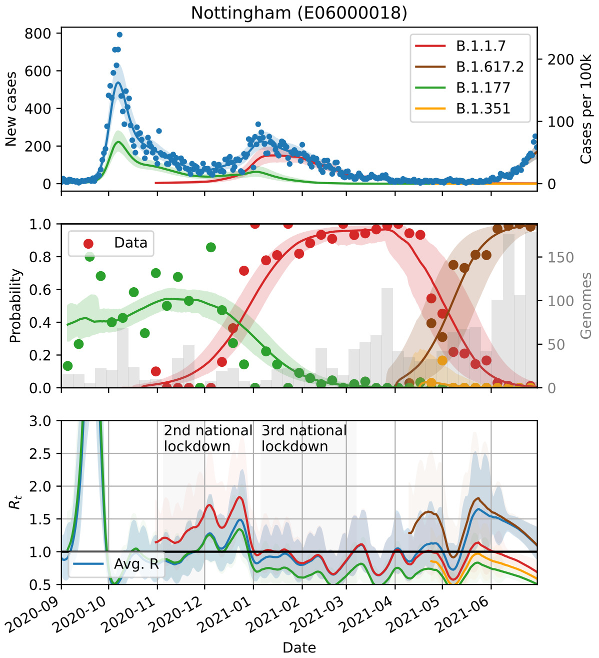
Nottingham (E06000018)
B.1.1.7
B.1.617.2
B.1.177
B.1.351
New cases
Cases per 100k
Data
Probability
Genomes
2nd national lockdown
3rd national lockdown
Avg. R
Date

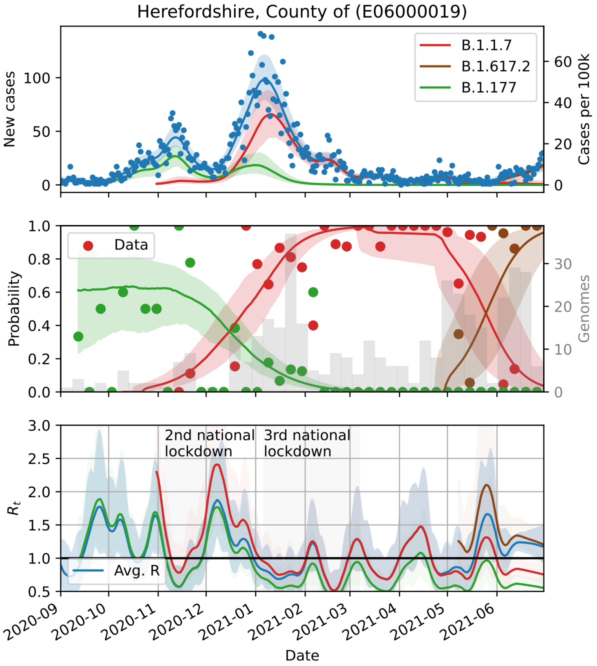
Herefordshire, County of (E06000019)
B.1.1.7
B.1.617.2
B.1.177
New cases
Cases per 100k
Data
Probability
Genomes
2nd national lockdown
3rd national lockdown
Avg. R
Date

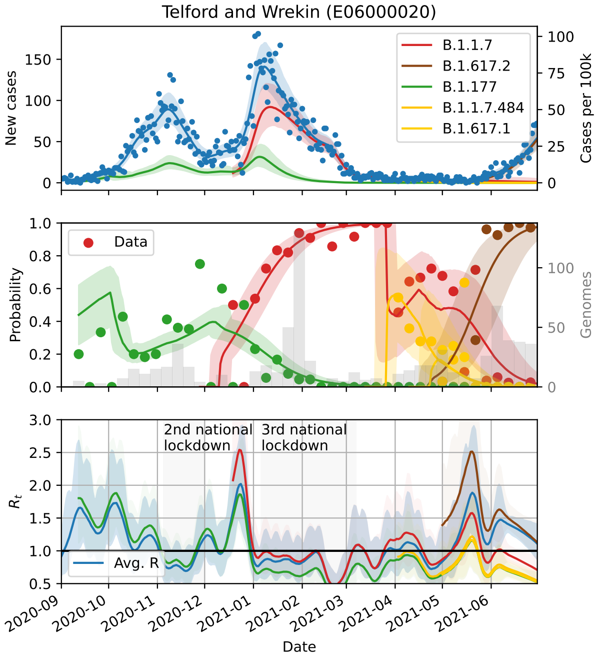
Telford and Wrekin (E06000020)
B.1.1.7
B.1.617.2
B.1.177
B.1.1.7.484
B.1.617.1
New cases
Cases per 100k
Data
Probability
Genomes
2nd national lockdown
3rd national lockdown
Avg. R
Date

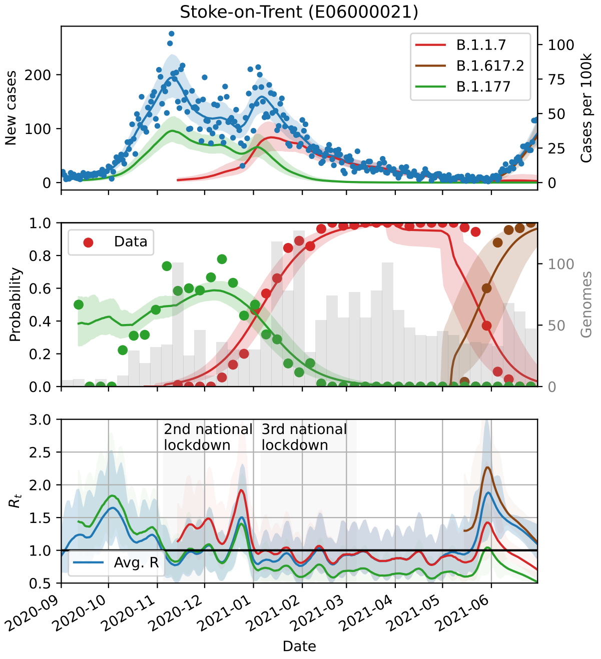
Stoke-on-Trent (E06000021)
New cases
Cases per 100k
B.1.1.7
B.1.617.2
B.1.177
Data
Probability
Genomes
2nd national lockdown
3rd national lockdown
R_t
Avg. R
Date

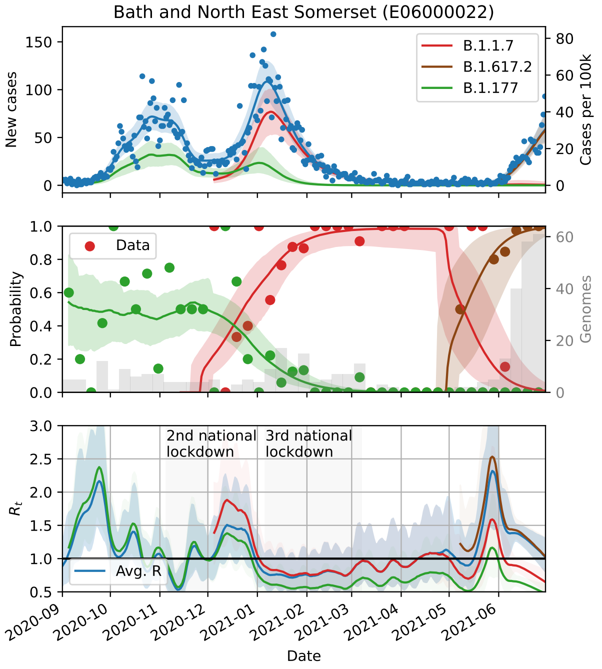
Bath and North East Somerset (E06000022)
New cases
Cases per 100k
B.1.1.7
B.1.617.2
B.1.177
Data
Probability
Genomes
2nd national lockdown
3rd national lockdown
R_t
Avg. R
Date

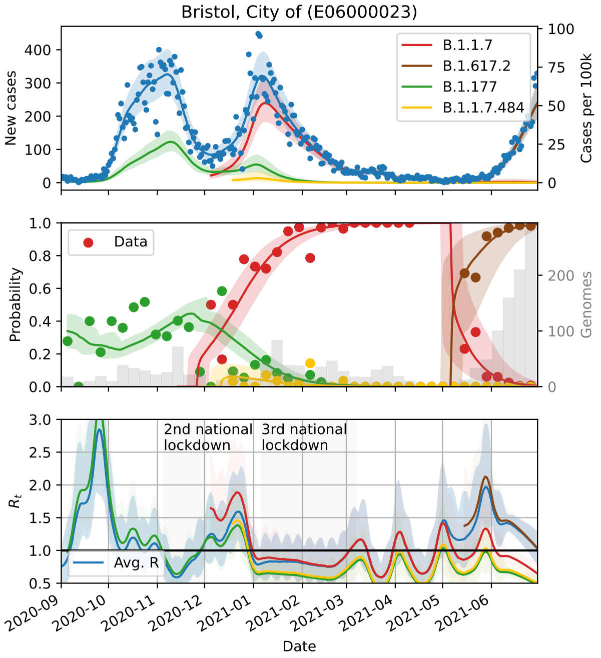
Bristol, City of (E06000023)
New cases
Cases per 100k
B.1.1.7
B.1.617.2
B.1.177
B.1.1.7.484
Data
Probability
Genomes
2nd national lockdown
3rd national lockdown
R_t
Avg. R
Date

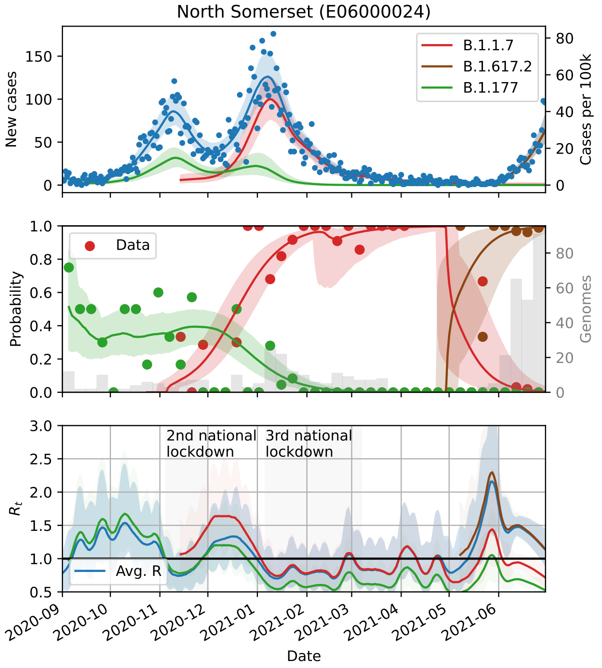
North Somerset (E06000024)
New cases
Cases per 100k
B.1.1.7
B.1.617.2
B.1.177
Data
Probability
Genomes
2nd national lockdown
3rd national lockdown
R_t
Avg. R
Date

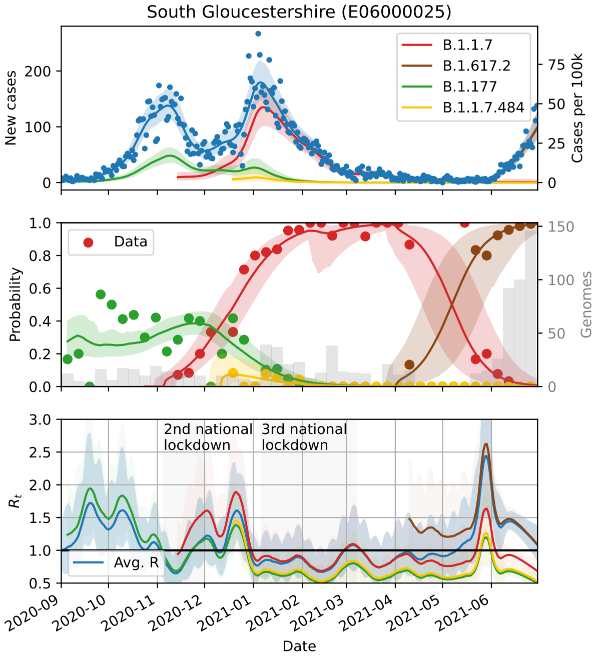
South Gloucestershire (E06000025)
B.1.1.7
B.1.617.2
B.1.177
B.1.1.7.484
New cases
Cases per 100k
Data
Probability
Genomes
2nd national lockdown
3rd national lockdown
Avg. R
R_t
Date

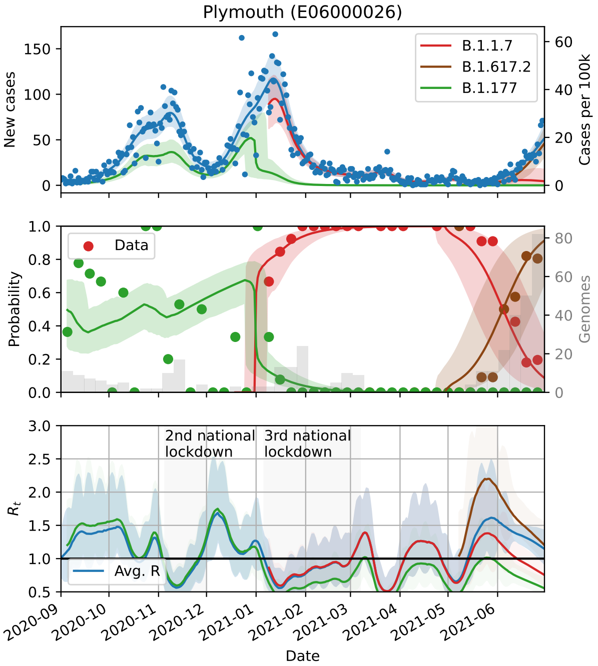
Plymouth (E06000026)
B.1.1.7
B.1.617.2
B.1.177
New cases
Cases per 100k
Data
Probability
Genomes
2nd national lockdown
3rd national lockdown
Avg. R
R_t
Date

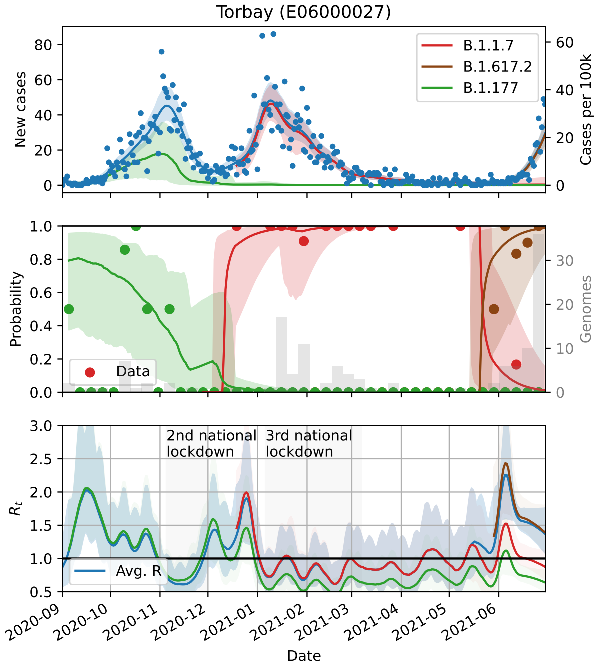
Torbay (E06000027)
B.1.1.7
B.1.617.2
B.1.177
New cases
Cases per 100k
Data
Probability
Genomes
2nd national lockdown
3rd national lockdown
Avg. R
R_t
Date

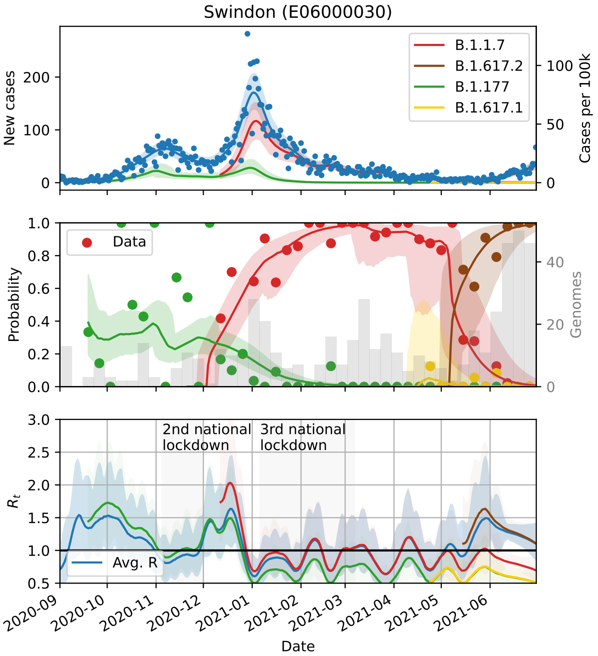
Swindon (E06000030)
B.1.1.7
B.1.617.2
B.1.177
B.1.617.1
New cases
Cases per 100k
Data
Probability
Genomes
2nd national lockdown
3rd national lockdown
Avg. R
R_t
Date

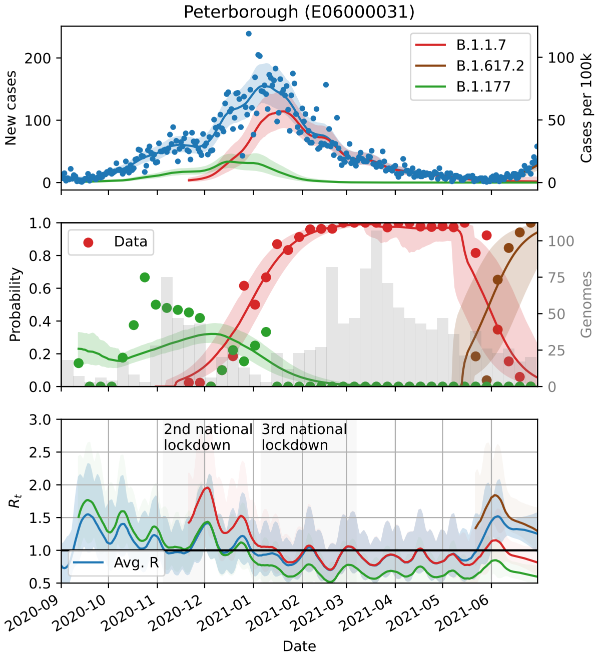
Peterborough (E06000031)
B.1.1.7
B.1.617.2
B.1.177
New cases
Cases per 100k
Data
Probability
Genomes
2nd national lockdown
3rd national lockdown
Avg. R
R_t
Date

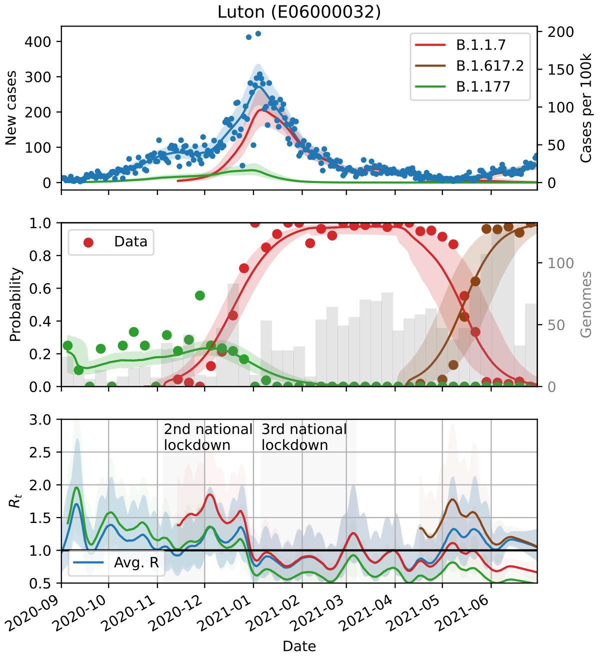
Luton (E06000032)
B.1.1.7
B.1.617.2
B.1.177
New cases
Cases per 100k
Data
Probability
Genomes
2nd national lockdown
3rd national lockdown
Avg. R
R_t
Date

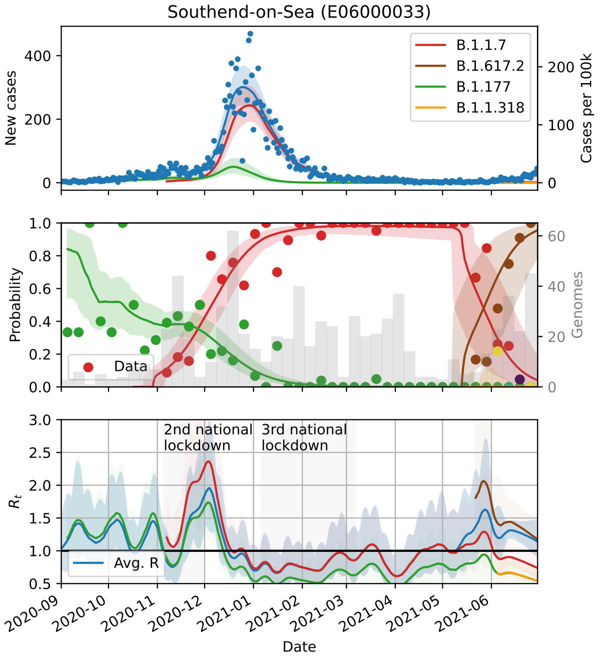
Southend-on-Sea (E06000033)
B.1.1.7
B.1.617.2
B.1.177
B.1.1.318
New cases
Cases per 100k
Data
Probability
Genomes
2nd national lockdown
3rd national lockdown
Avg. R
R_t
Date

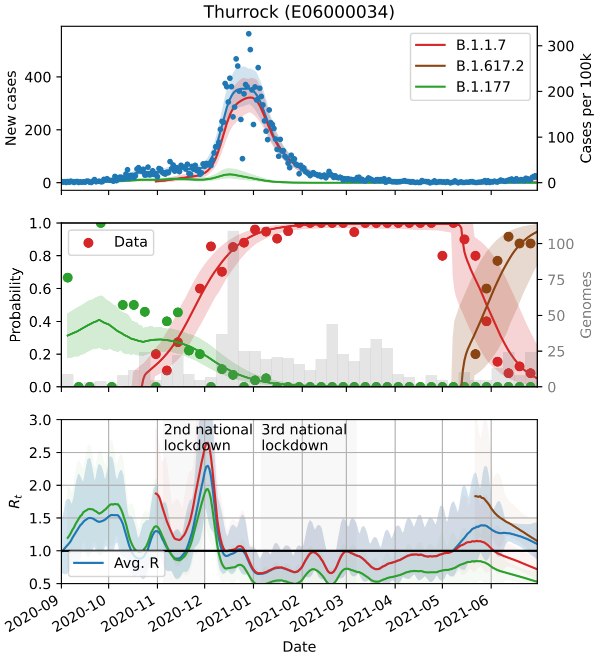
Thurrock (E06000034)
B.1.1.7
B.1.617.2
B.1.177
New cases
Cases per 100k
Data
Probability
Genomes
2nd national lockdown
3rd national lockdown
Avg. R
R_t
Date

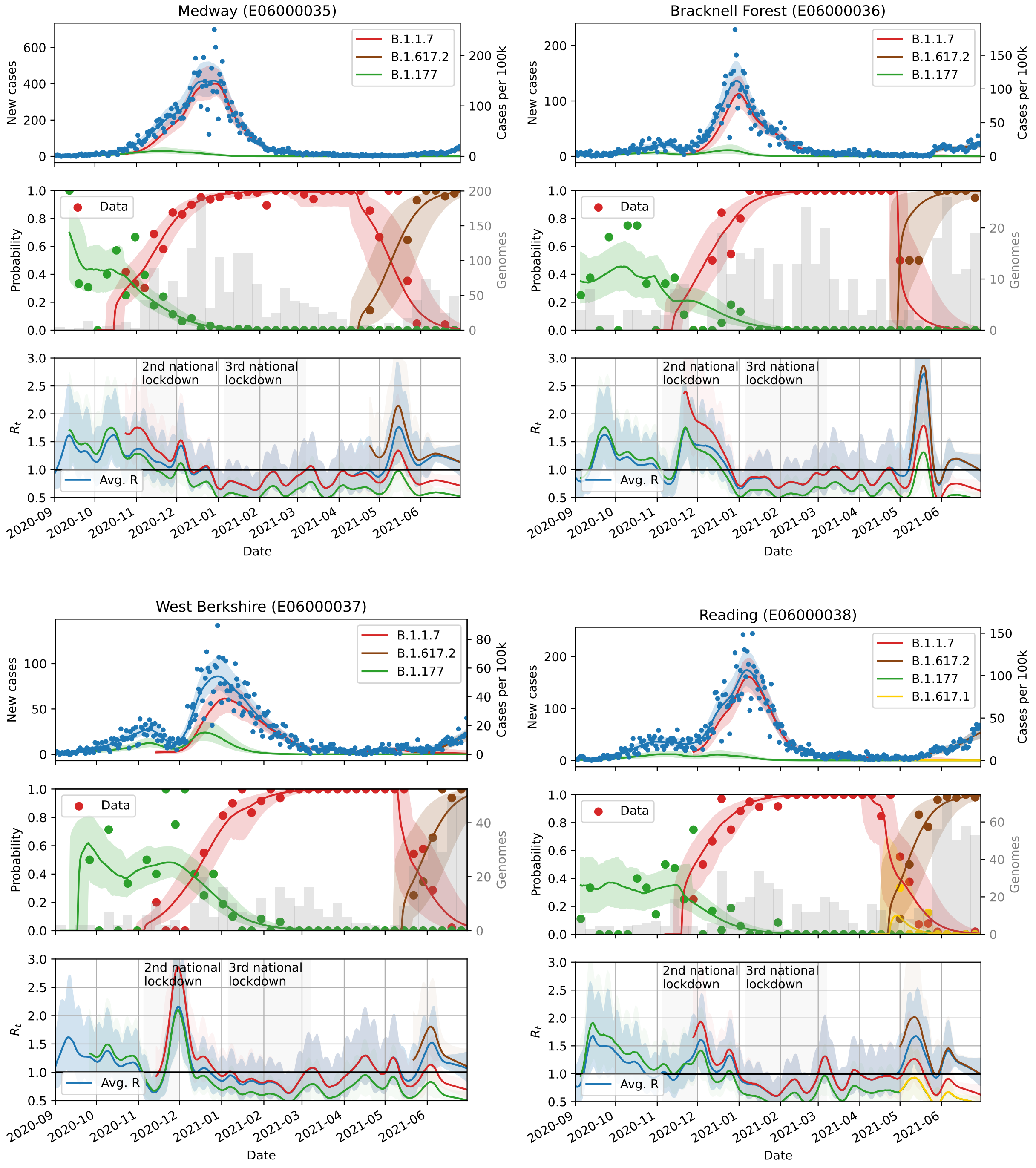

Medway (E06000035)
Bracknell Forest (E06000036)
West Berkshire (E06000037)
Reading (E06000038)
B.1.1.7
B.1.617.2
B.1.177
B.1.617.1
New cases
Cases per 100k
Data
Probability
Genomes
2nd national lockdown
3rd national lockdown
R_t
Avg. R
Date
2020-09
2020-10
2020-11
2020-12
2021-01
2021-02
2021-03
2021-04
2021-05
2021-06

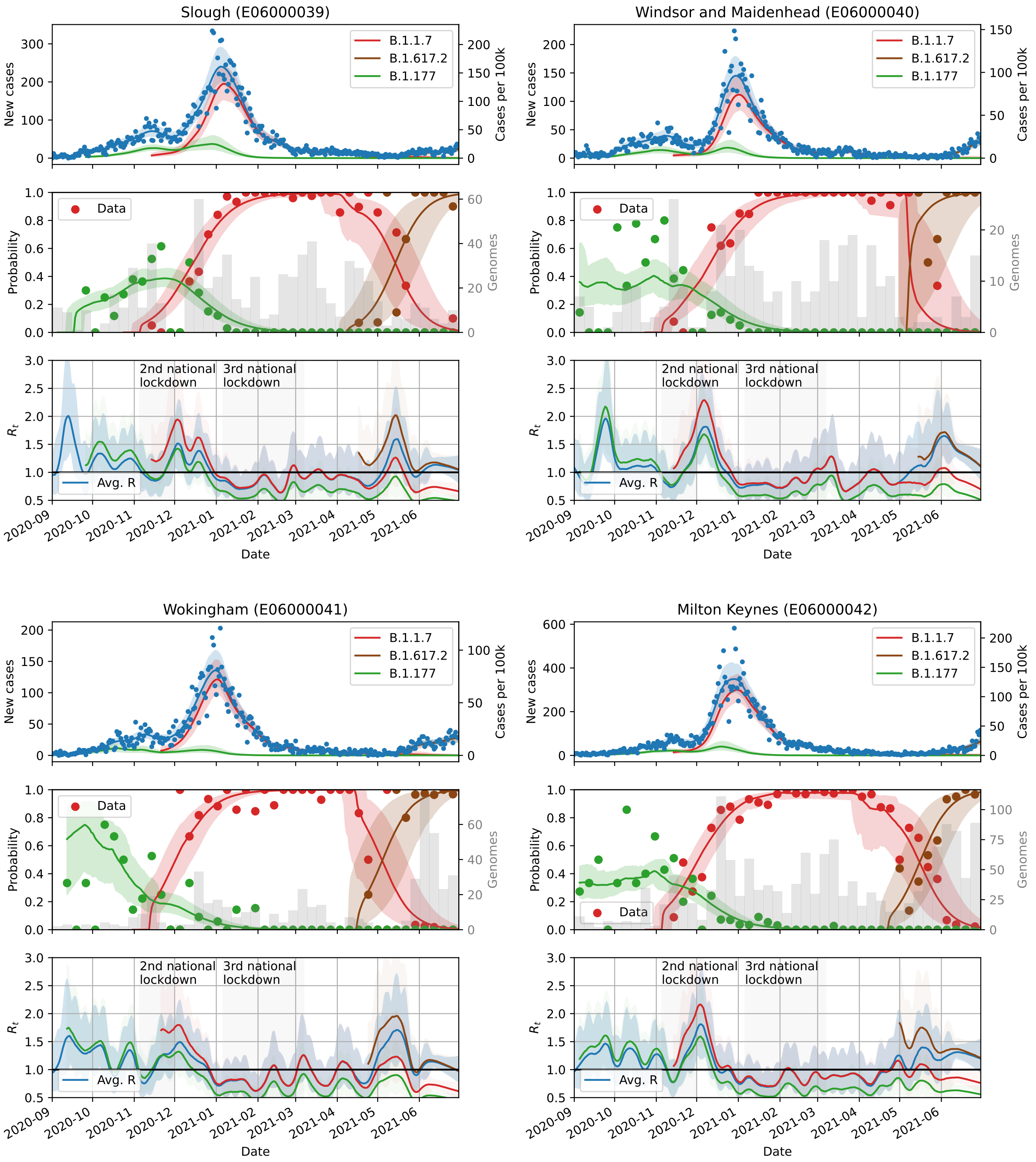
Slough (E06000039)
Windsor and Maidenhead (E06000040)
Wokingham (E06000041)
Milton Keynes (E06000042)
B.1.1.7
B.1.617.2
B.1.177
New cases
Cases per 100k
Data
Probability
Genomes
2nd national lockdown
3rd national lockdown
Avg. R
R_t
Date
2020-09
2020-10
2020-11
2020-12
2021-01
2021-02
2021-03
2021-04
2021-05
2021-06

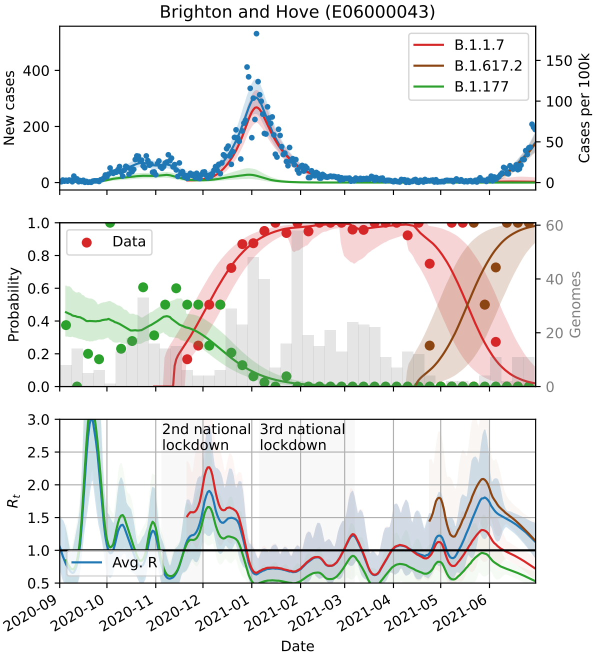
Brighton and Hove (E06000043)
New cases
Cases per 100k
B.1.1.7
B.1.617.2
B.1.177
Probability
Genomes
Data
R_t
2nd national lockdown
3rd national lockdown
Avg. R
Date

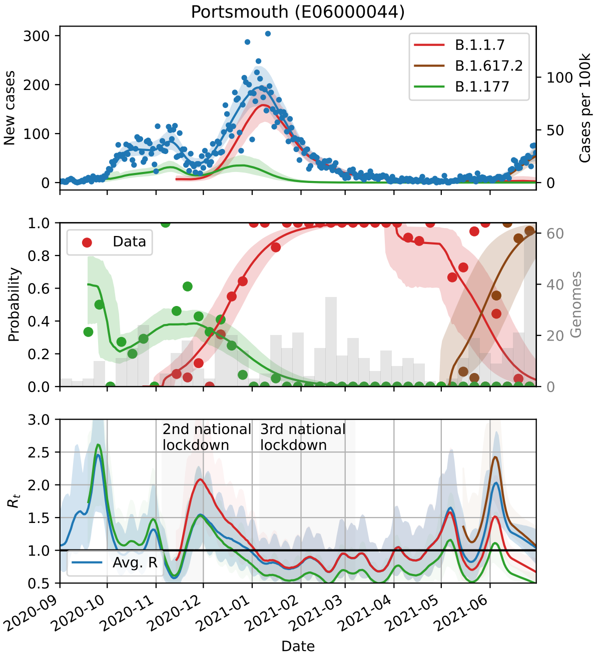
Portsmouth (E06000044)
New cases
Cases per 100k
B.1.1.7
B.1.617.2
B.1.177
Probability
Genomes
Data
R_t
2nd national lockdown
3rd national lockdown
Avg. R
Date

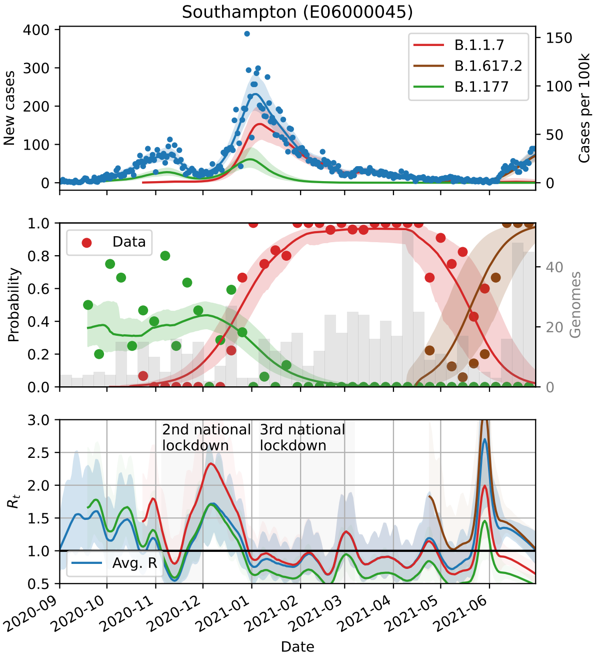
Southampton (E06000045)
New cases
Cases per 100k
B.1.1.7
B.1.617.2
B.1.177
Probability
Genomes
Data
R_t
2nd national lockdown
3rd national lockdown
Avg. R
Date

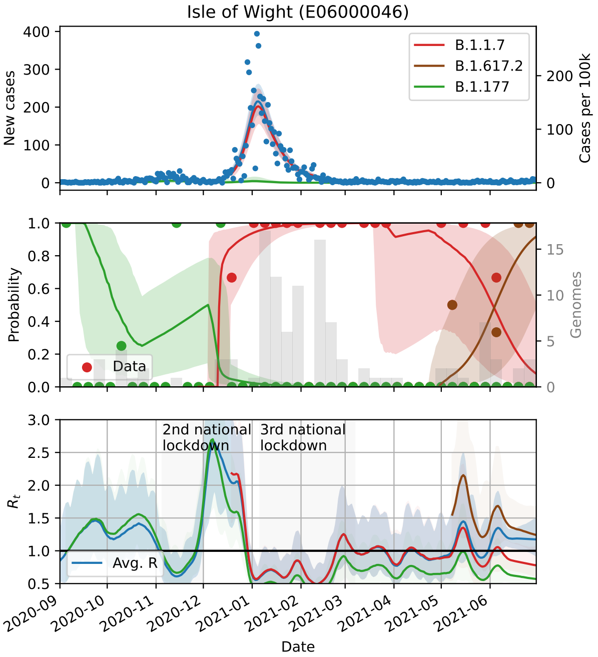
Isle of Wight (E06000046)
New cases
Cases per 100k
B.1.1.7
B.1.617.2
B.1.177
Probability
Genomes
Data
R_t
2nd national lockdown
3rd national lockdown
Avg. R
Date

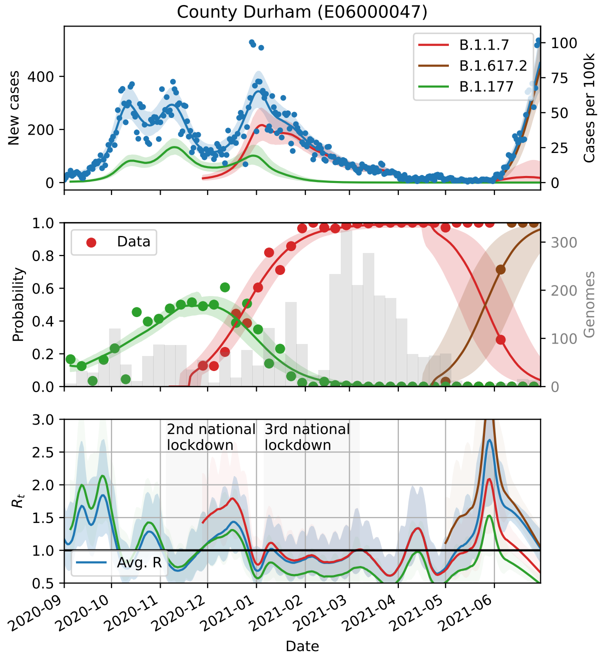
County Durham (E06000047)
B.1.1.7
B.1.617.2
B.1.177
New cases
Cases per 100k
Data
Probability
Genomes
2nd national lockdown
3rd national lockdown
Avg. R
R_t
Date

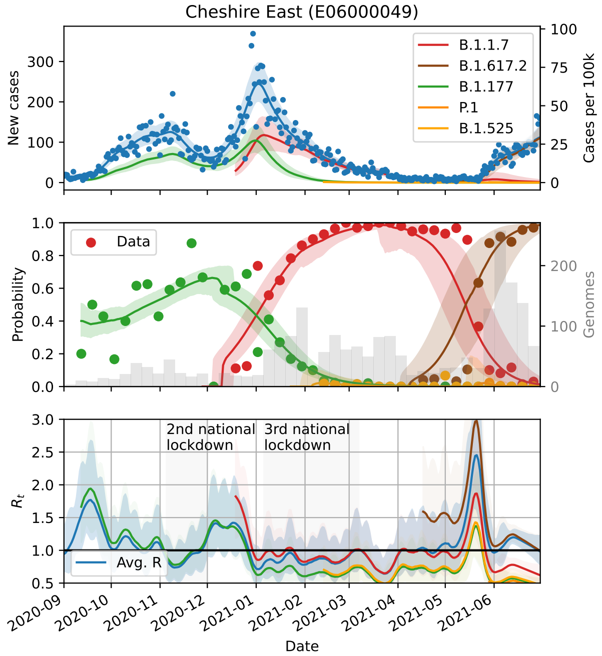
Cheshire East (E06000049)
B.1.1.7
B.1.617.2
B.1.177
P.1
B.1.525
New cases
Cases per 100k
Data
Probability
Genomes
2nd national lockdown
3rd national lockdown
Avg. R
R_t
Date

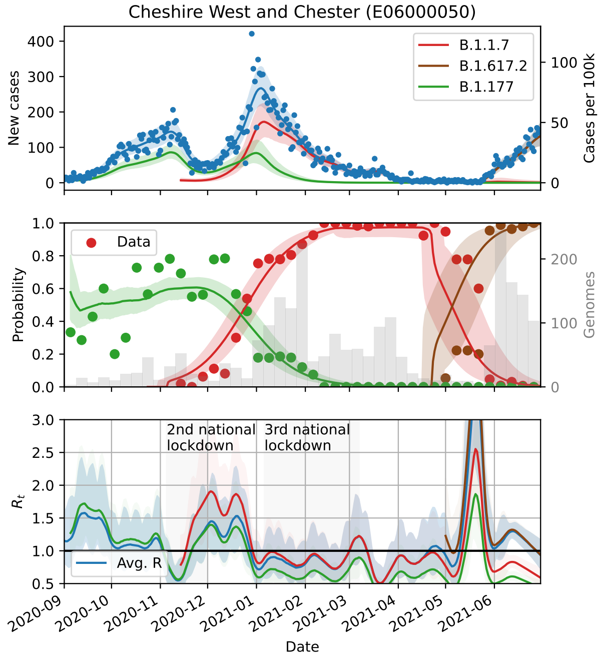
Cheshire West and Chester (E06000050)
B.1.1.7
B.1.617.2
B.1.177
New cases
Cases per 100k
Data
Probability
Genomes
2nd national lockdown
3rd national lockdown
Avg. R
R_t
Date

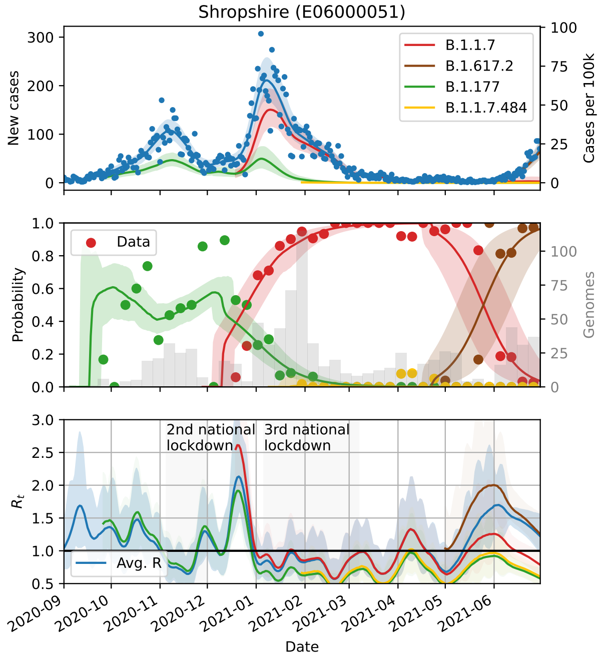
Shropshire (E06000051)
B.1.1.7
B.1.617.2
B.1.177
B.1.1.7.484
New cases
Cases per 100k
Data
Probability
Genomes
2nd national lockdown
3rd national lockdown
Avg. R
R_t
Date

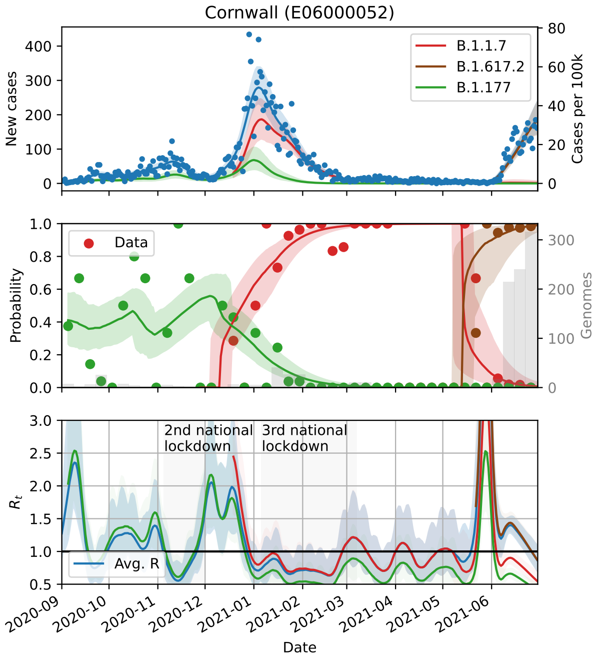
Cornwall (E06000052)
New cases
Cases per 100k
B.1.1.7
B.1.617.2
B.1.177
Probability
Genomes
Data
2nd national lockdown
3rd national lockdown
R_t
Avg. R
2020-09
2020-10
2020-11
2020-12
2021-01
2021-02
2021-03
2021-04
2021-05
2021-06
Date

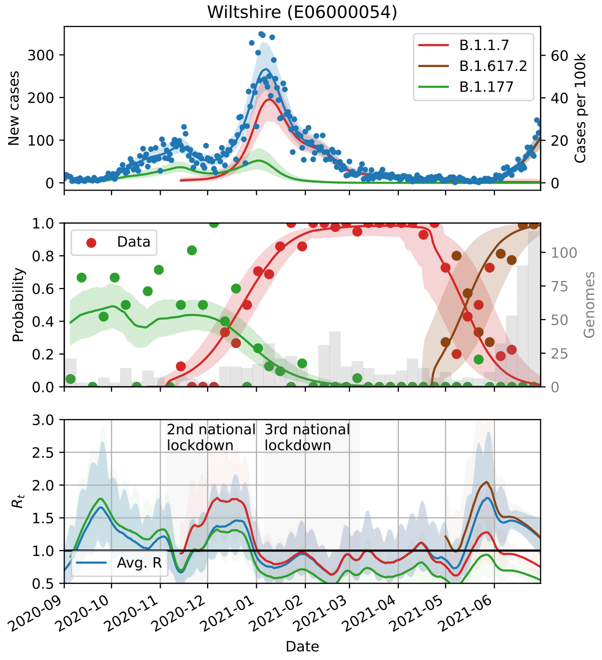
Wiltshire (E06000054)
New cases
Cases per 100k
B.1.1.7
B.1.617.2
B.1.177
Probability
Genomes
Data
2nd national lockdown
3rd national lockdown
R_t
Avg. R
2020-09
2020-10
2020-11
2020-12
2021-01
2021-02
2021-03
2021-04
2021-05
2021-06
Date

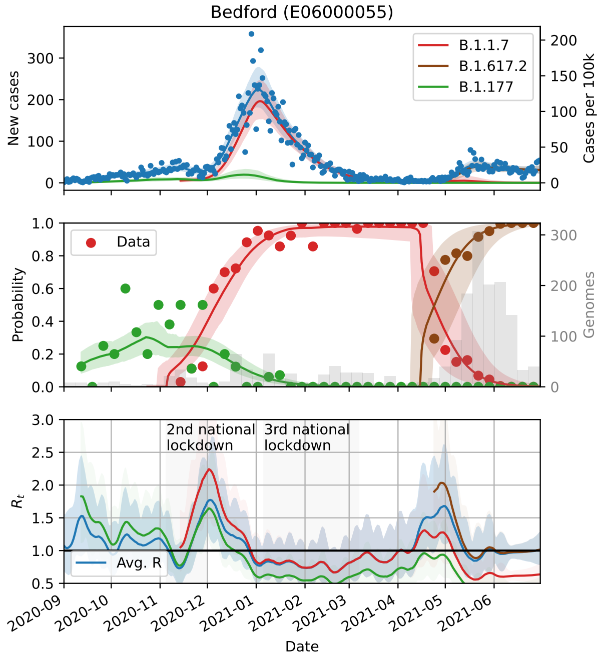
Bedford (E06000055)
New cases
Cases per 100k
B.1.1.7
B.1.617.2
B.1.177
Probability
Genomes
Data
2nd national lockdown
3rd national lockdown
R_t
Avg. R
2020-09
2020-10
2020-11
2020-12
2021-01
2021-02
2021-03
2021-04
2021-05
2021-06
Date

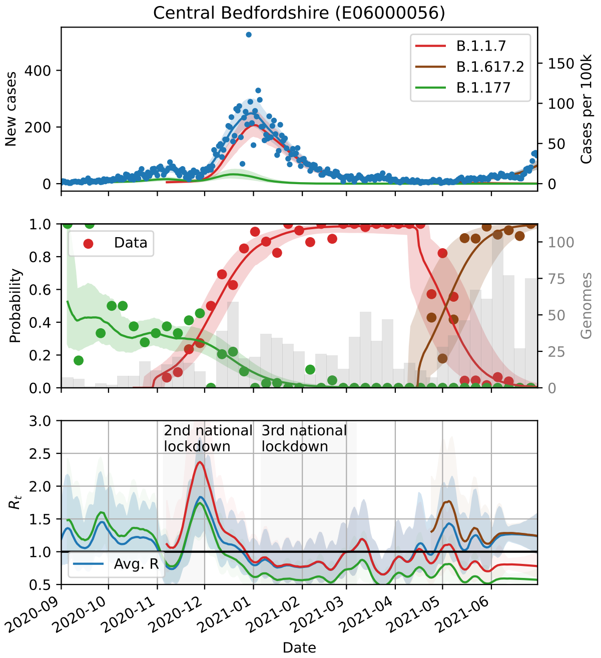
Central Bedfordshire (E06000056)
New cases
Cases per 100k
B.1.1.7
B.1.617.2
B.1.177
Probability
Data
Genomes
R_t
2nd national lockdown
3rd national lockdown
Avg. R
Date

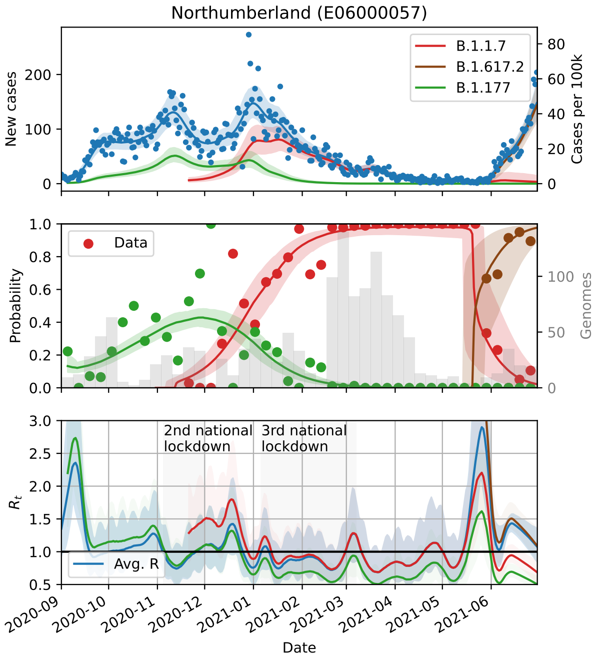
Northumberland (E06000057)
New cases
Cases per 100k
B.1.1.7
B.1.617.2
B.1.177
Probability
Data
Genomes
R_t
2nd national lockdown
3rd national lockdown
Avg. R
Date

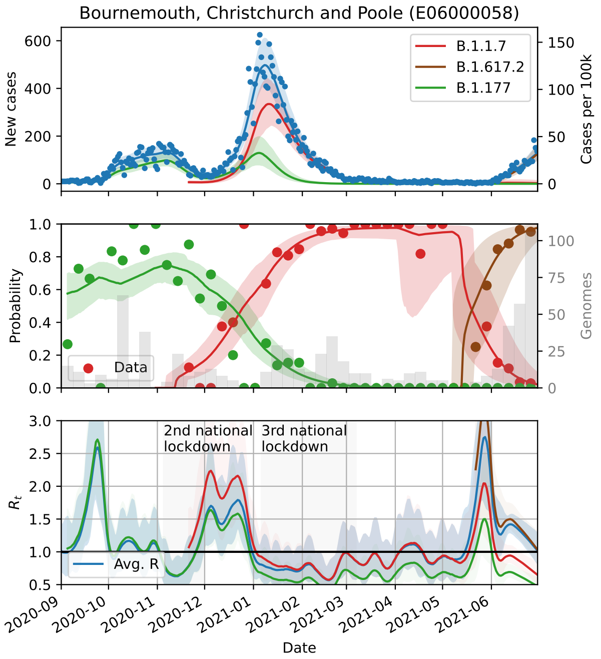
Bournemouth, Christchurch and Poole (E06000058)
New cases
Cases per 100k
B.1.1.7
B.1.617.2
B.1.177
Probability
Data
Genomes
R_t
2nd national lockdown
3rd national lockdown
Avg. R
Date

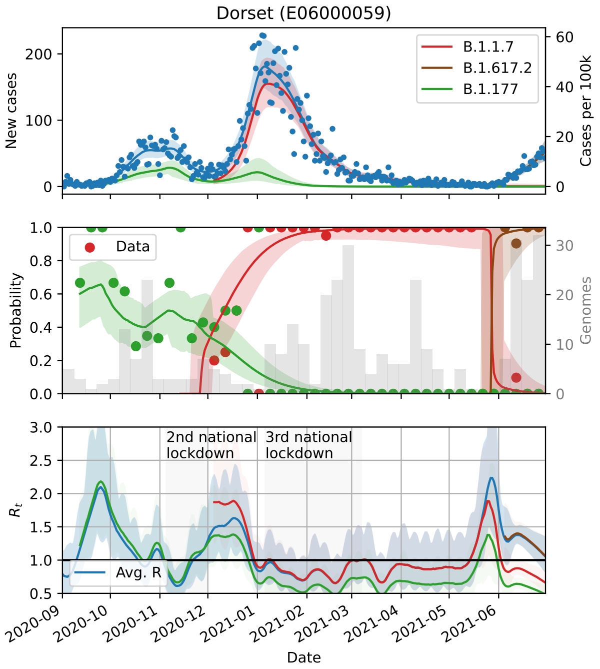
Dorset (E06000059)
New cases
Cases per 100k
B.1.1.7
B.1.617.2
B.1.177
Probability
Data
Genomes
R_t
2nd national lockdown
3rd national lockdown
Avg. R
Date

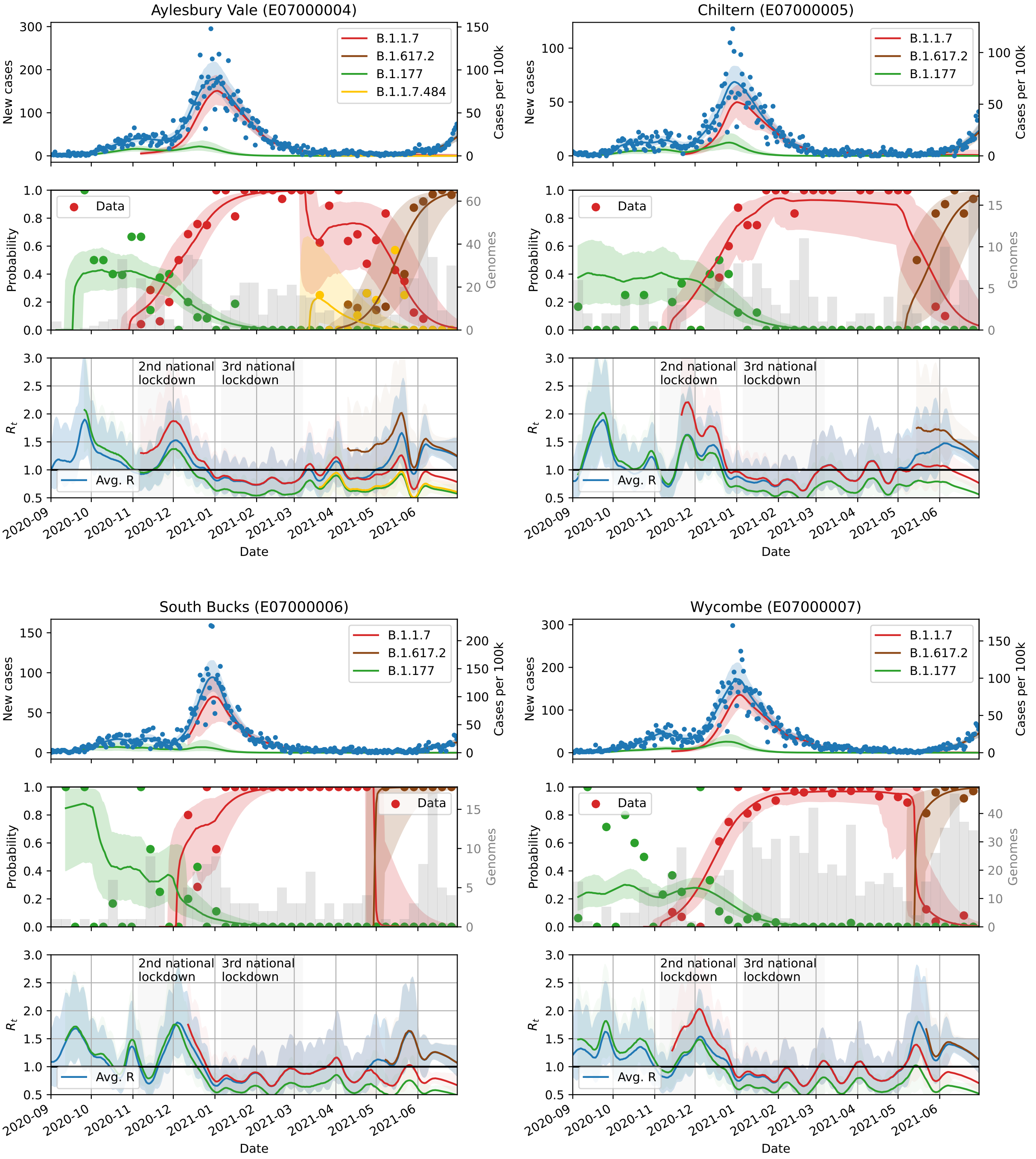

Aylesbury Vale (E07000004)
Chiltern (E07000005)
South Bucks (E07000006)
Wycombe (E07000007)
B.1.1.7
B.1.617.2
B.1.177
B.1.1.7.484
New cases
Cases per 100k
Data
Probability
Genomes
2nd national lockdown
3rd national lockdown
Avg. R
R_t
Date
2020-09
2020-10
2020-11
2020-12
2021-01
2021-02
2021-03
2021-04
2021-05
2021-06

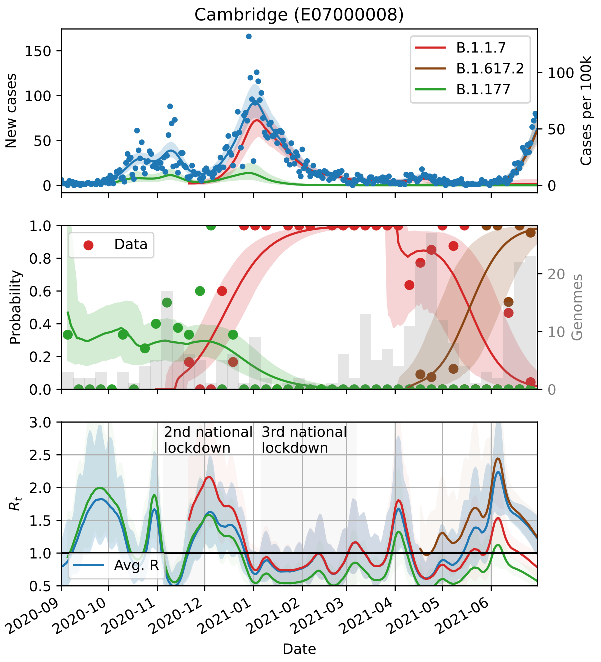
Cambridge (E07000008)
New cases
Cases per 100k
B.1.1.7
B.1.617.2
B.1.177
Data
Probability
Genomes
2nd national lockdown
3rd national lockdown
Avg. R
R_t
Date

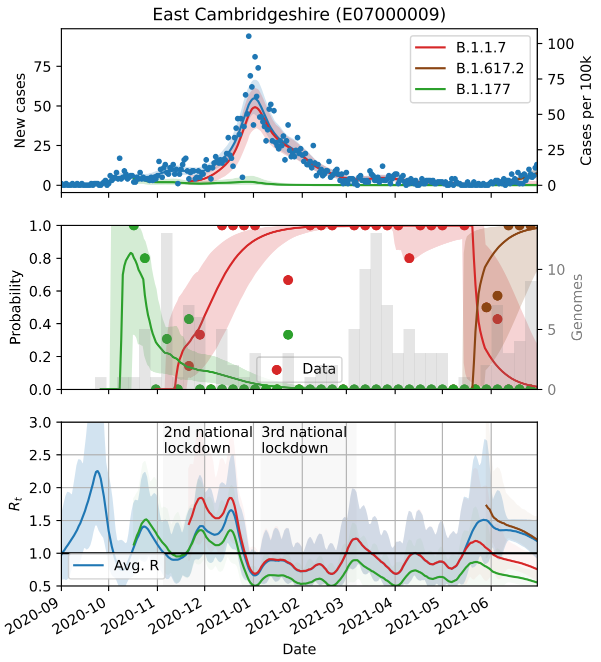
East Cambridgeshire (E07000009)
New cases
Cases per 100k
B.1.1.7
B.1.617.2
B.1.177
Data
Probability
Genomes
2nd national lockdown
3rd national lockdown
Avg. R
R_t
Date

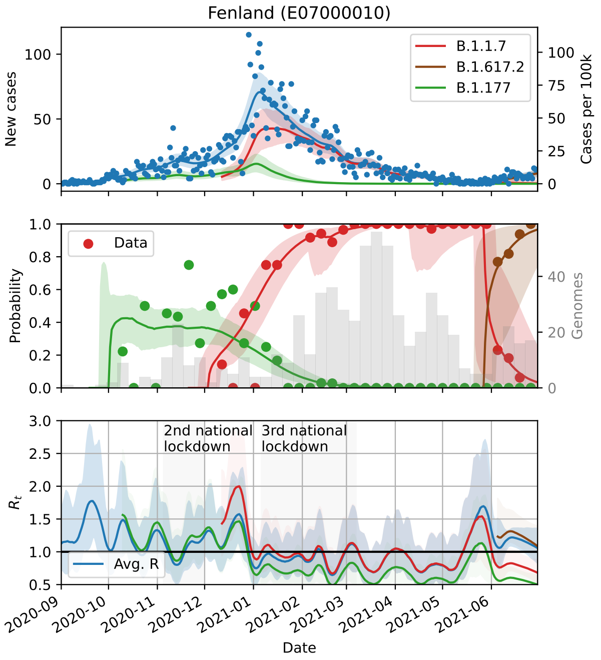
Fenland (E07000010)
New cases
Cases per 100k
B.1.1.7
B.1.617.2
B.1.177
Data
Probability
Genomes
2nd national lockdown
3rd national lockdown
Avg. R
R_t
Date

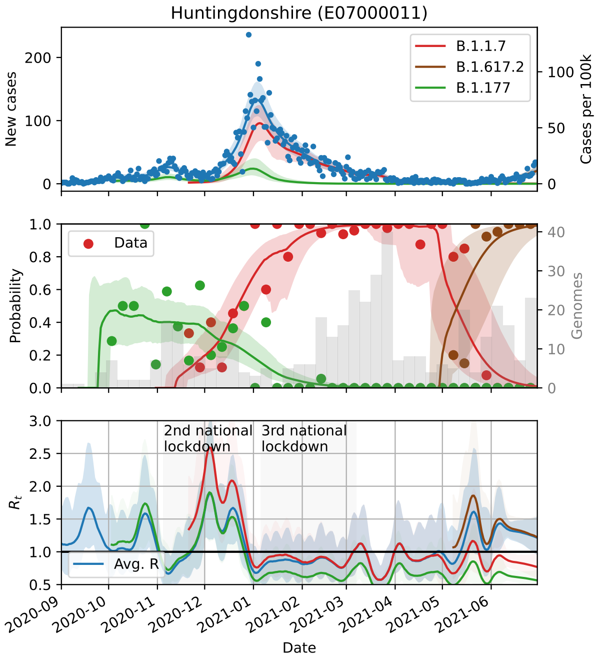
Huntingdonshire (E07000011)
New cases
Cases per 100k
B.1.1.7
B.1.617.2
B.1.177
Data
Probability
Genomes
2nd national lockdown
3rd national lockdown
Avg. R
R_t
Date

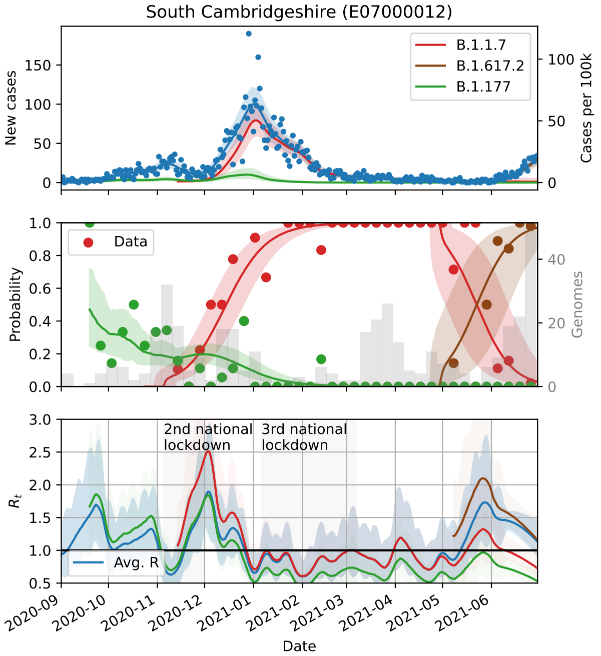
South Cambridgeshire (E07000012)
B.1.1.7
B.1.617.2
B.1.177
New cases
Cases per 100k
Data
Probability
Genomes
2nd national lockdown
3rd national lockdown
R_t
Avg. R
Date

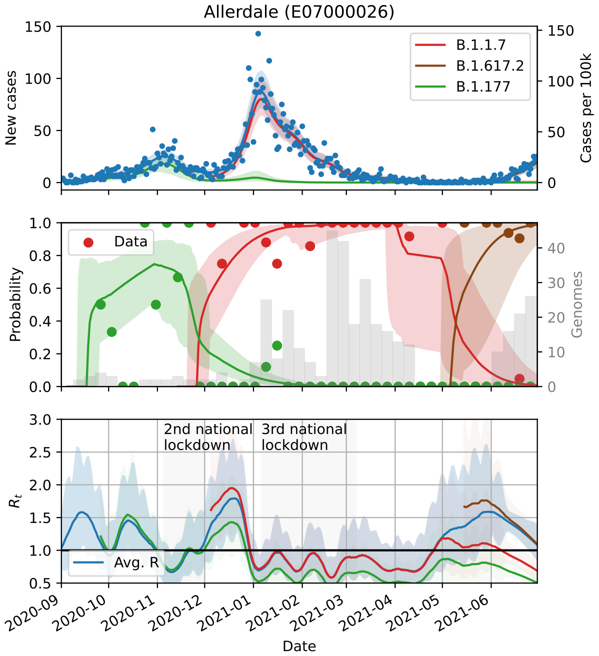
Allerdale (E07000026)
B.1.1.7
B.1.617.2
B.1.177
New cases
Cases per 100k
Data
Probability
Genomes
2nd national lockdown
3rd national lockdown
R_t
Avg. R
Date

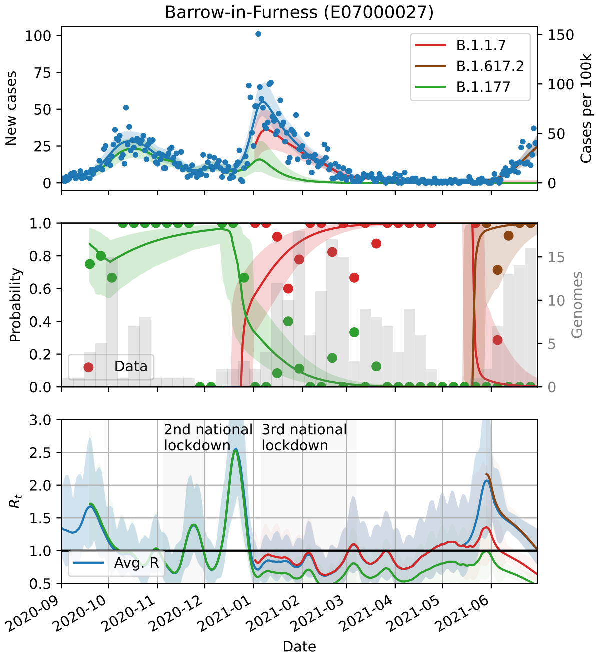
Barrow-in-Furness (E07000027)
B.1.1.7
B.1.617.2
B.1.177
New cases
Cases per 100k
Data
Probability
Genomes
2nd national lockdown
3rd national lockdown
R_t
Avg. R
Date

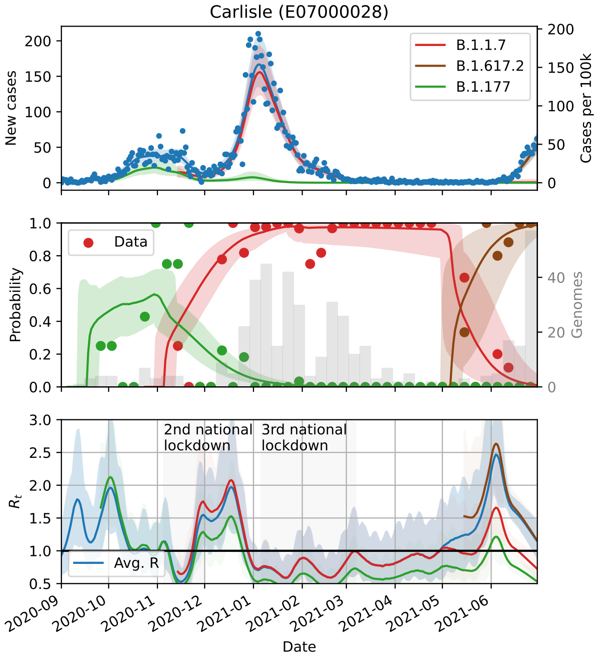
Carlisle (E07000028)
B.1.1.7
B.1.617.2
B.1.177
New cases
Cases per 100k
Data
Probability
Genomes
2nd national lockdown
3rd national lockdown
R_t
Avg. R
Date

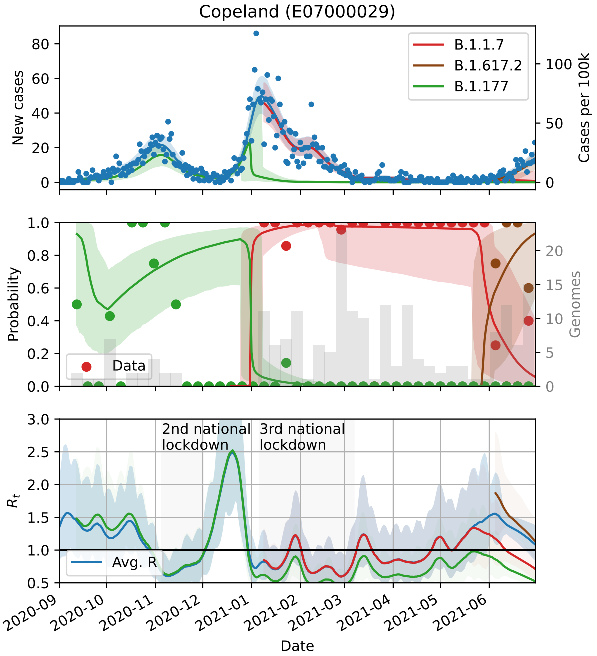
Copeland (E07000029)
New cases
Cases per 100k
B.1.1.7
B.1.617.2
B.1.177
Probability
Genomes
Data
R_t
2nd national lockdown
3rd national lockdown
Avg. R
Date

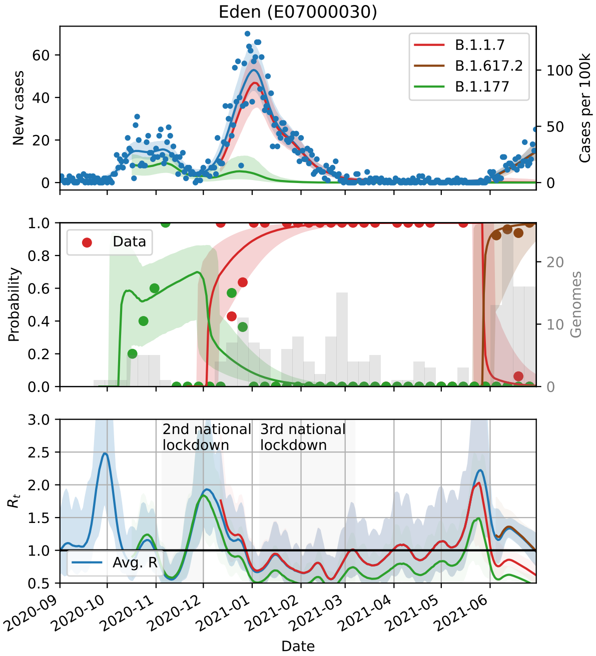
Eden (E07000030)
New cases
Cases per 100k
B.1.1.7
B.1.617.2
B.1.177
Probability
Genomes
Data
R_t
2nd national lockdown
3rd national lockdown
Avg. R
Date

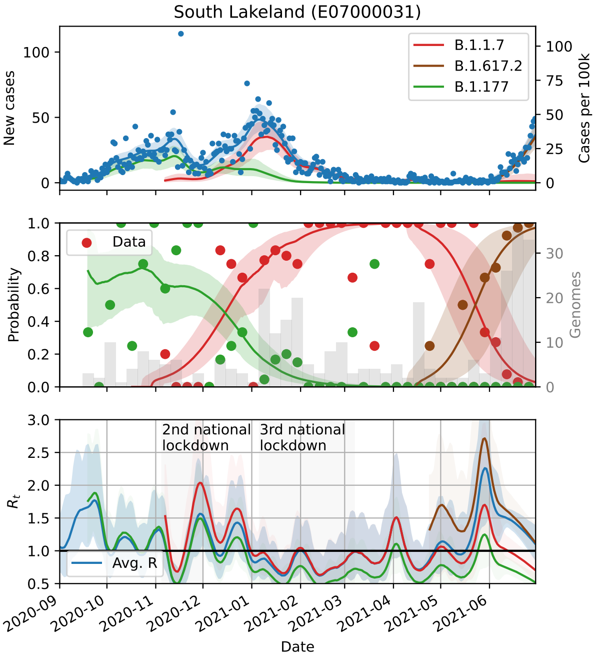
South Lakeland (E07000031)
New cases
Cases per 100k
B.1.1.7
B.1.617.2
B.1.177
Probability
Genomes
Data
R_t
2nd national lockdown
3rd national lockdown
Avg. R
Date

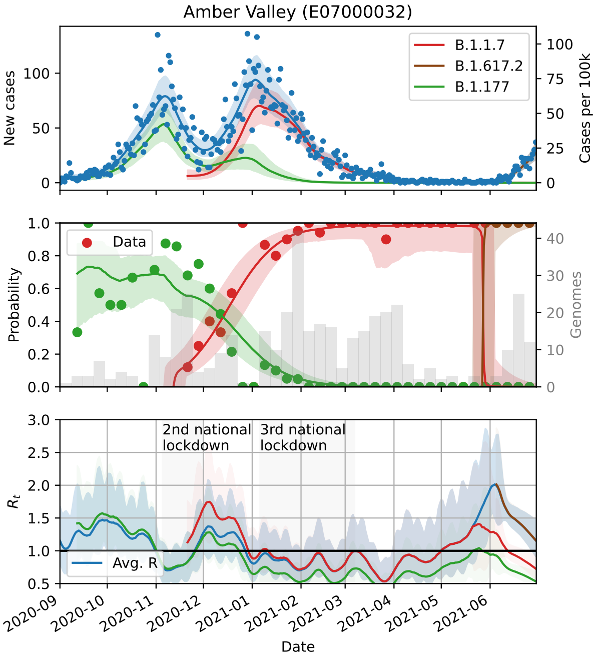
Amber Valley (E07000032)
New cases
Cases per 100k
B.1.1.7
B.1.617.2
B.1.177
Probability
Genomes
Data
R_t
2nd national lockdown
3rd national lockdown
Avg. R
Date

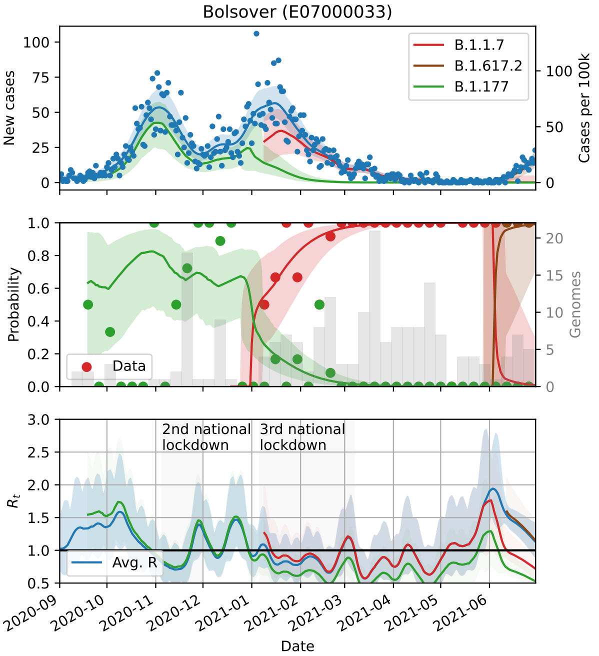
Bolsover (E07000033)
New cases
Cases per 100k
B.1.1.7
B.1.617.2
B.1.177
Probability
Genomes
Data
R_t
2nd national lockdown
3rd national lockdown
Avg. R
Date

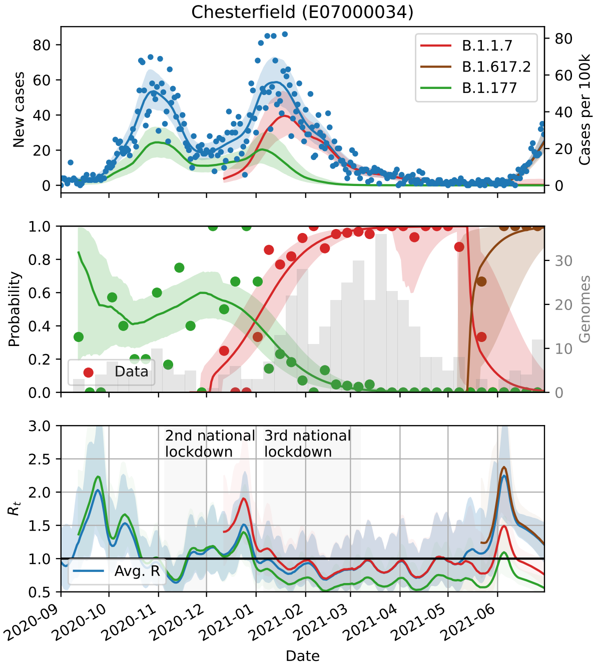
Chesterfield (E07000034)
New cases
Cases per 100k
B.1.1.7
B.1.617.2
B.1.177
Probability
Genomes
Data
R_t
2nd national lockdown
3rd national lockdown
Avg. R
Date

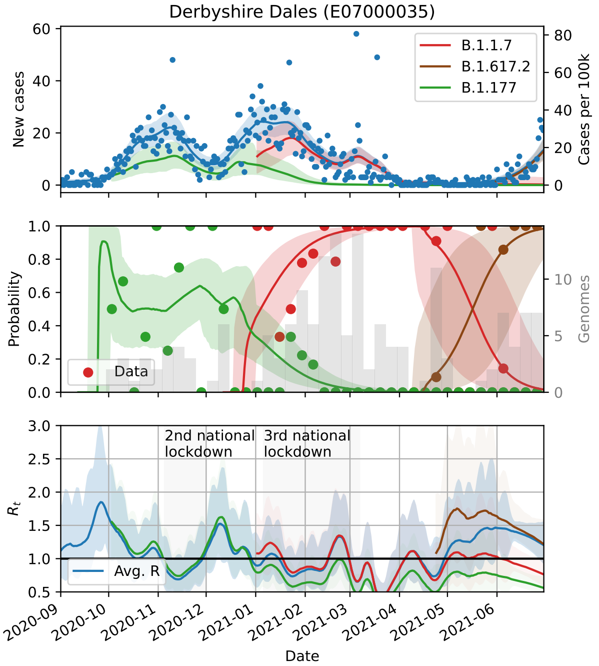
Derbyshire Dales (E07000035)
New cases
Cases per 100k
B.1.1.7
B.1.617.2
B.1.177
Probability
Genomes
Data
R_t
2nd national lockdown
3rd national lockdown
Avg. R
Date

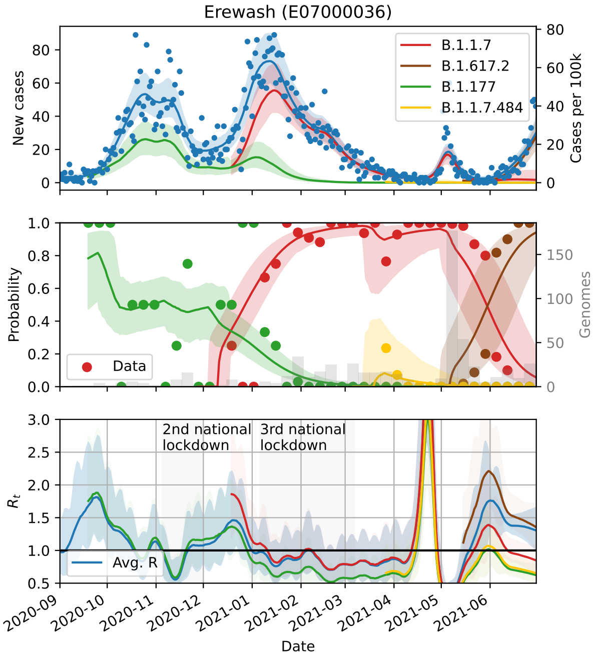
Erewash (E07000036)
New cases
Cases per 100k
B.1.1.7
B.1.617.2
B.1.177
B.1.1.7.484
Probability
Genomes
Data
R_t
2nd national lockdown
3rd national lockdown
Avg. R
Date

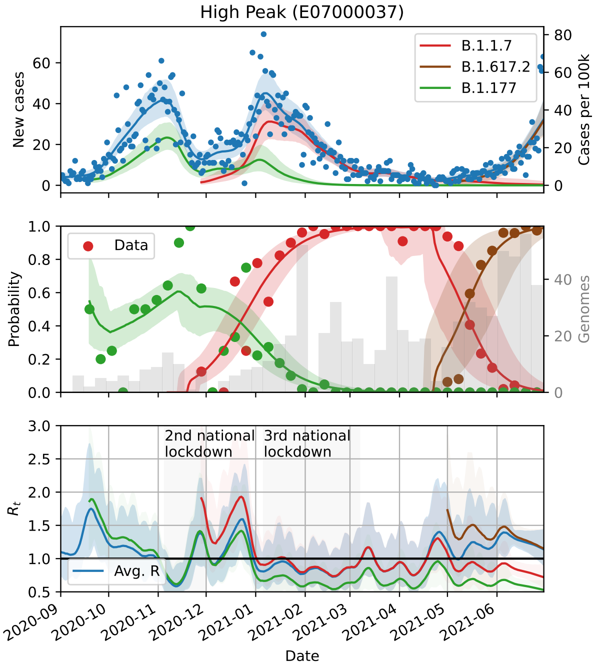
High Peak (E07000037)
New cases
Cases per 100k
B.1.1.7
B.1.617.2
B.1.177
Data
Probability
Genomes
2nd national lockdown
3rd national lockdown
R_t
Avg. R
Date

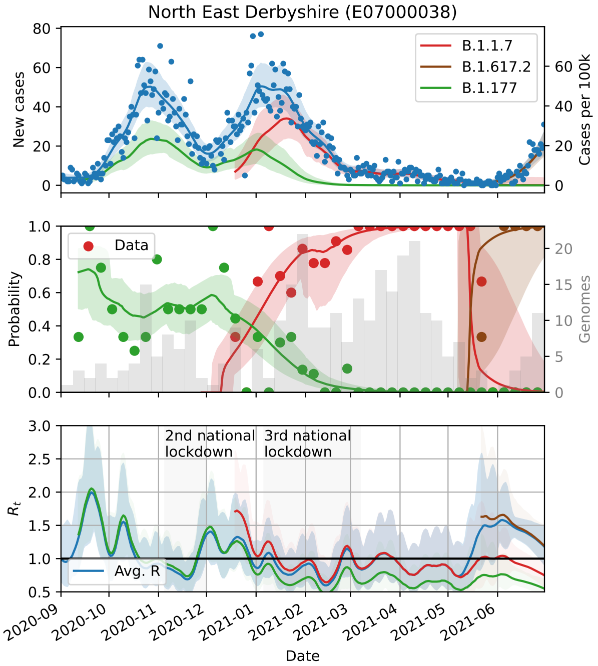
North East Derbyshire (E07000038)
New cases
Cases per 100k
B.1.1.7
B.1.617.2
B.1.177
Data
Probability
Genomes
2nd national lockdown
3rd national lockdown
R_t
Avg. R
Date

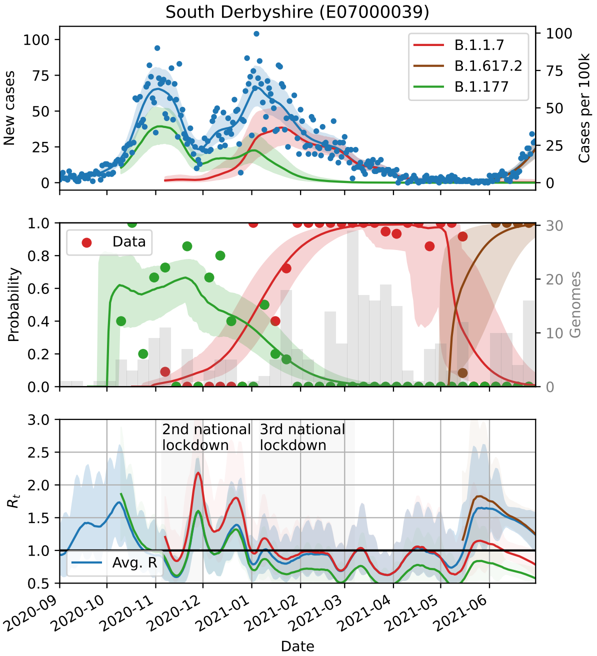
South Derbyshire (E07000039)
New cases
Cases per 100k
B.1.1.7
B.1.617.2
B.1.177
Data
Probability
Genomes
2nd national lockdown
3rd national lockdown
R_t
Avg. R
Date

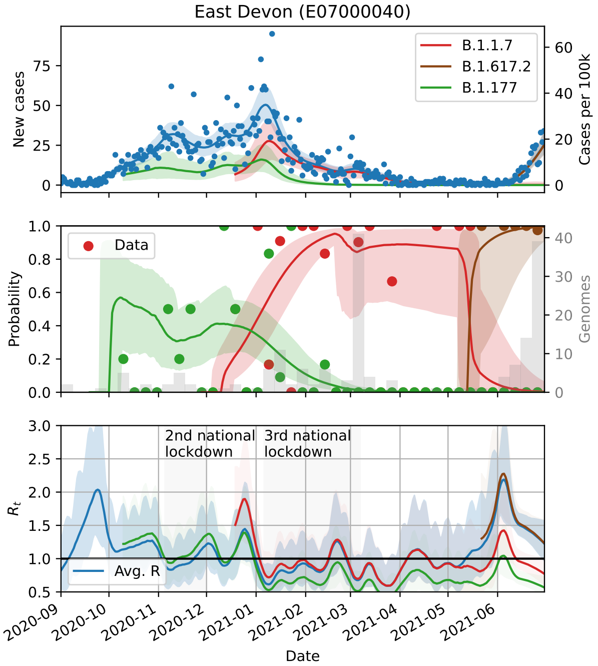
East Devon (E07000040)
New cases
Cases per 100k
B.1.1.7
B.1.617.2
B.1.177
Data
Probability
Genomes
2nd national lockdown
3rd national lockdown
R_t
Avg. R
Date

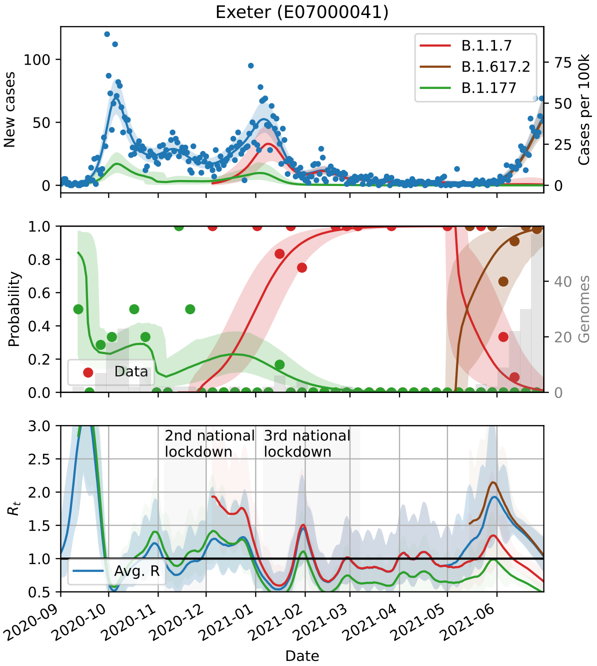
Exeter (E07000041)
B.1.1.7
B.1.617.2
B.1.177
New cases
Cases per 100k
Probability
Genomes
Data
2nd national lockdown
3rd national lockdown
Avg. R
R_t
Date

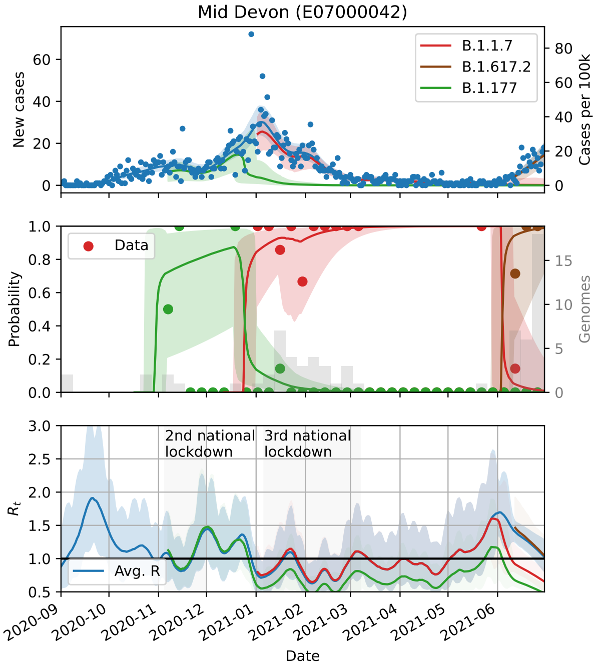
Mid Devon (E07000042)
B.1.1.7
B.1.617.2
B.1.177
New cases
Cases per 100k
Probability
Genomes
Data
2nd national lockdown
3rd national lockdown
Avg. R
R_t
Date

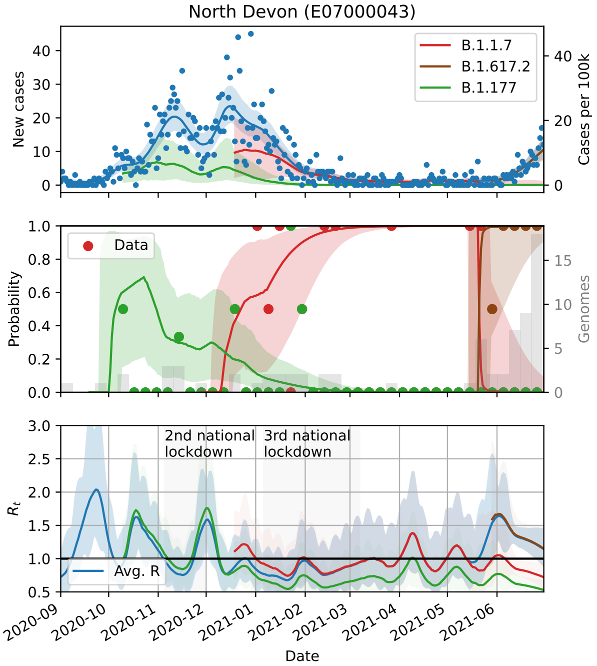
North Devon (E07000043)
B.1.1.7
B.1.617.2
B.1.177
New cases
Cases per 100k
Probability
Genomes
Data
2nd national lockdown
3rd national lockdown
Avg. R
R_t
Date

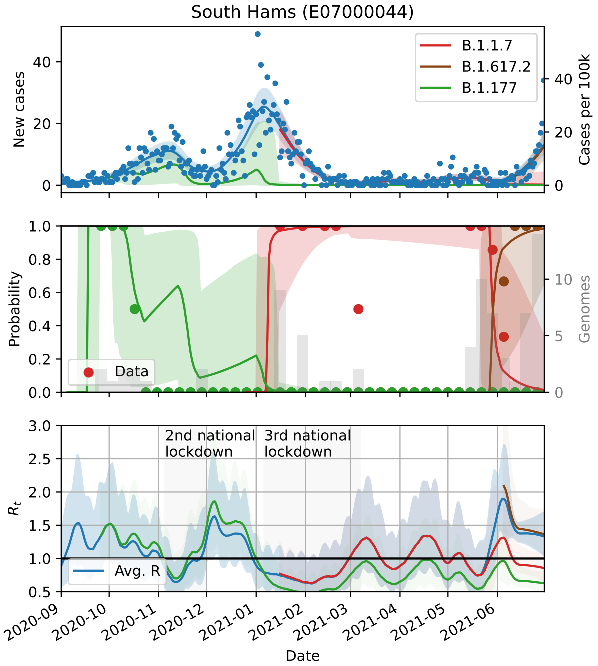
South Hams (E07000044)
B.1.1.7
B.1.617.2
B.1.177
New cases
Cases per 100k
Probability
Genomes
Data
2nd national lockdown
3rd national lockdown
Avg. R
R_t
Date

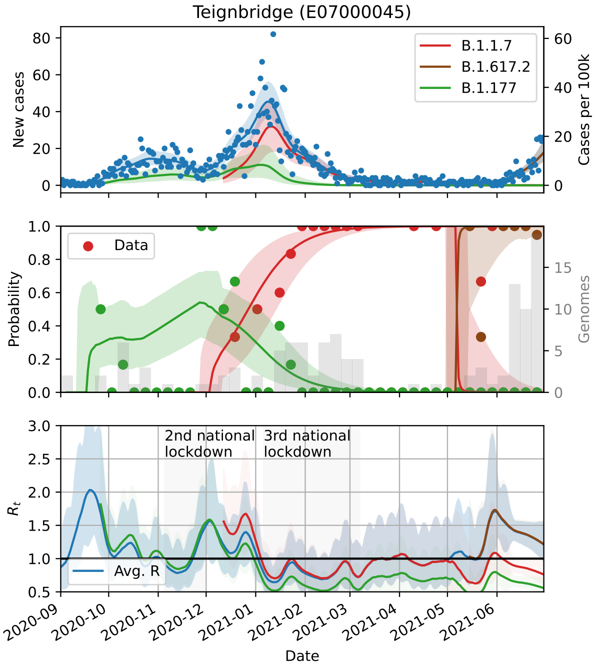
Teignbridge (E07000045)
B.1.1.7
B.1.617.2
B.1.177
New cases
Cases per 100k
Data
Probability
Genomes
2nd national lockdown
3rd national lockdown
Avg. R
R_t
Date

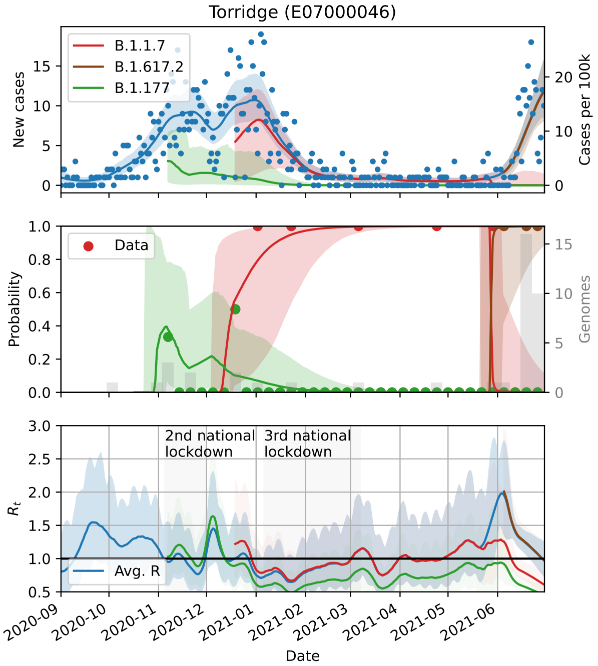
Torridge (E07000046)
B.1.1.7
B.1.617.2
B.1.177
New cases
Cases per 100k
Data
Probability
Genomes
2nd national lockdown
3rd national lockdown
Avg. R
R_t
Date

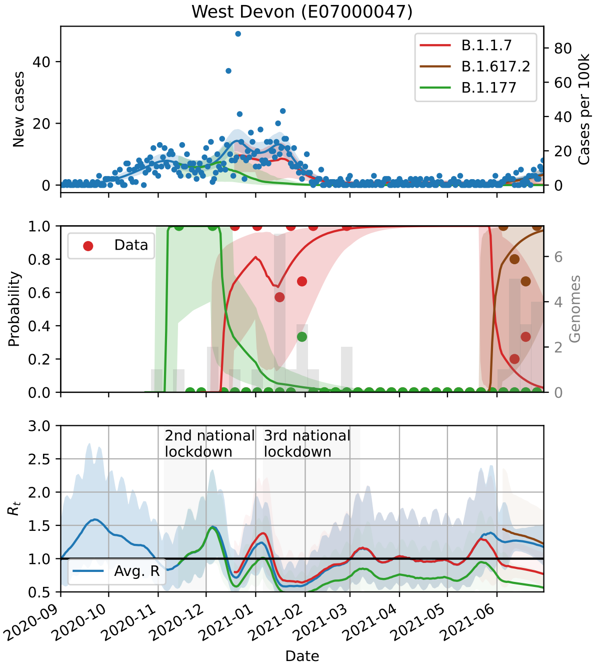
West Devon (E07000047)
B.1.1.7
B.1.617.2
B.1.177
New cases
Cases per 100k
Data
Probability
Genomes
2nd national lockdown
3rd national lockdown
Avg. R
R_t
Date

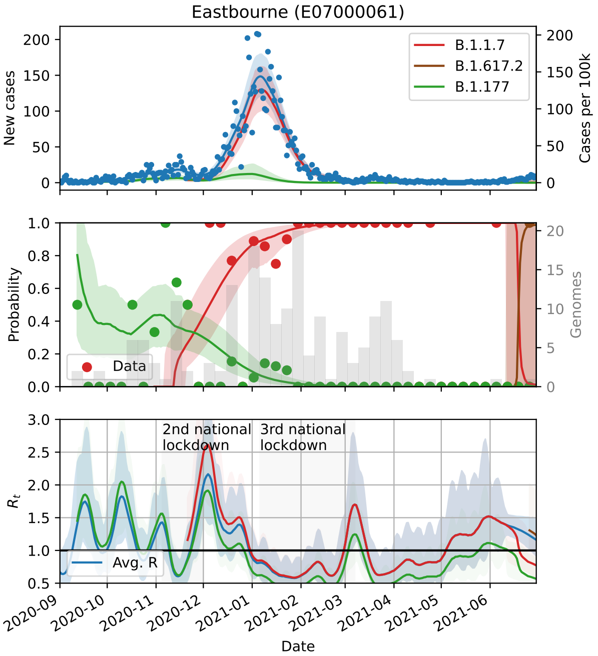
Eastbourne (E07000061)
B.1.1.7
B.1.617.2
B.1.177
New cases
Cases per 100k
Data
Probability
Genomes
2nd national lockdown
3rd national lockdown
Avg. R
R_t
Date

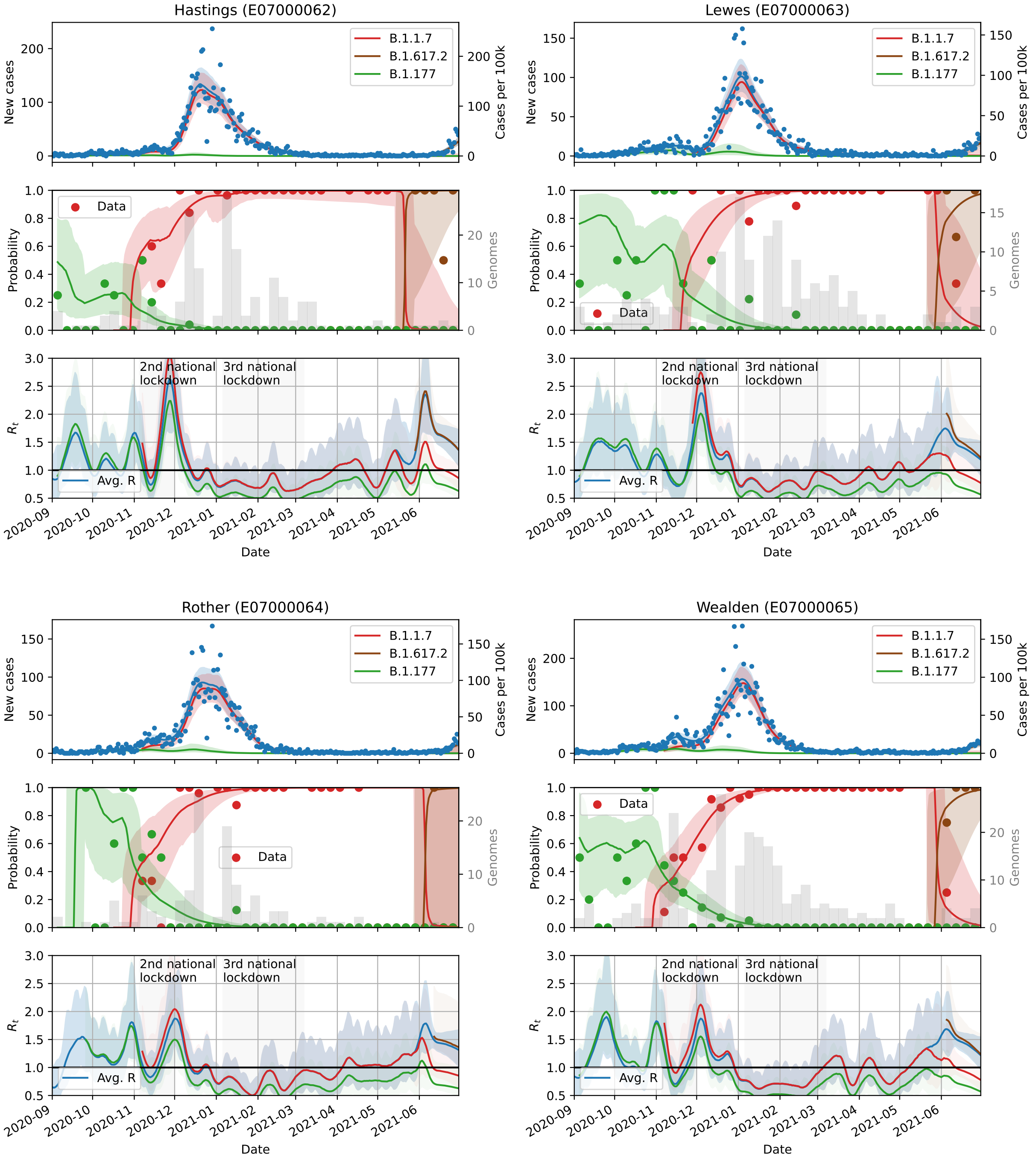
Hastings (E07000062)
Lewes (E07000063)
Rother (E07000064)
Wealden (E07000065)
B.1.1.7
B.1.617.2
B.1.177
New cases
Cases per 100k
Data
Probability
Genomes
2nd national lockdown
3rd national lockdown
R_t
Avg. R
2020-09
2020-10
2020-11
2020-12
2021-01
2021-02
2021-03
2021-04
2021-05
2021-06
Date

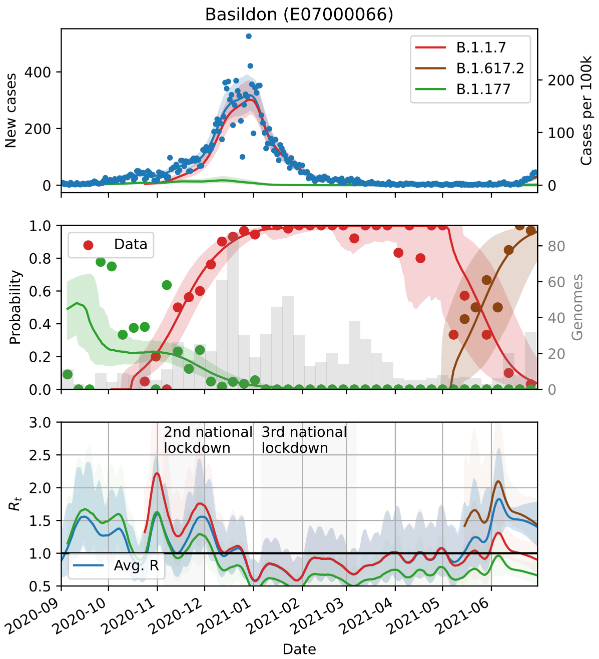

Basildon (E07000066)
New cases
Cases per 100k
B.1.1.7
B.1.617.2
B.1.177
Data
Probability
Genomes
2nd national lockdown
3rd national lockdown
R_t
Avg. R
Date

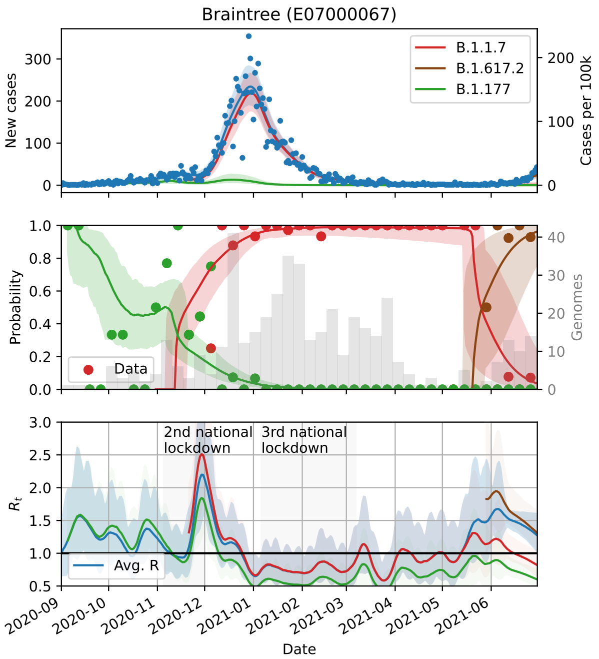

Braintree (E07000067)
New cases
Cases per 100k
B.1.1.7
B.1.617.2
B.1.177
Data
Probability
Genomes
2nd national lockdown
3rd national lockdown
R_t
Avg. R
Date

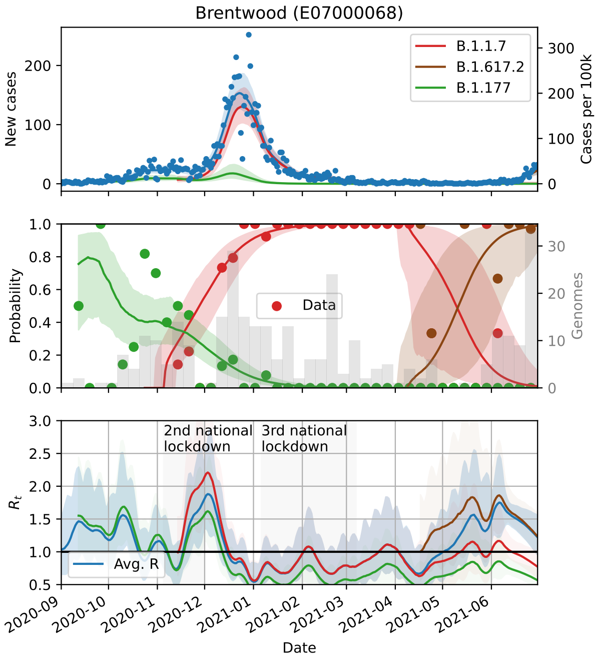

Brentwood (E07000068)
New cases
Cases per 100k
B.1.1.7
B.1.617.2
B.1.177
Data
Probability
Genomes
2nd national lockdown
3rd national lockdown
R_t
Avg. R
Date

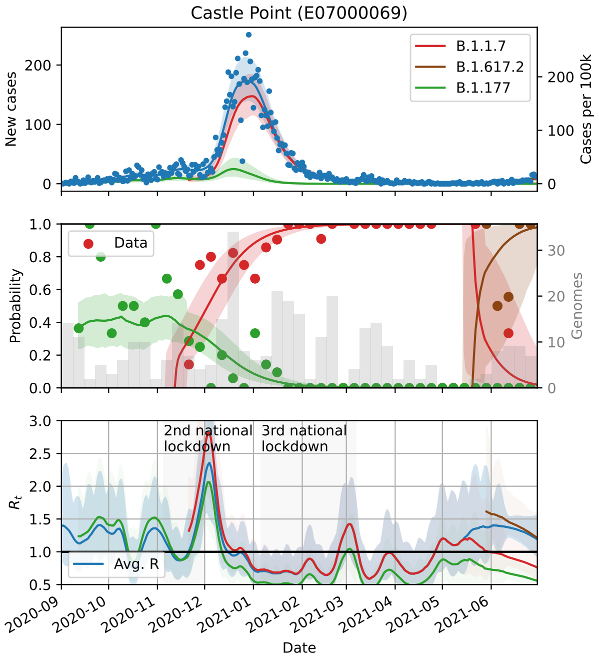

Castle Point (E07000069)
New cases
Cases per 100k
B.1.1.7
B.1.617.2
B.1.177
Data
Probability
Genomes
2nd national lockdown
3rd national lockdown
R_t
Avg. R
Date

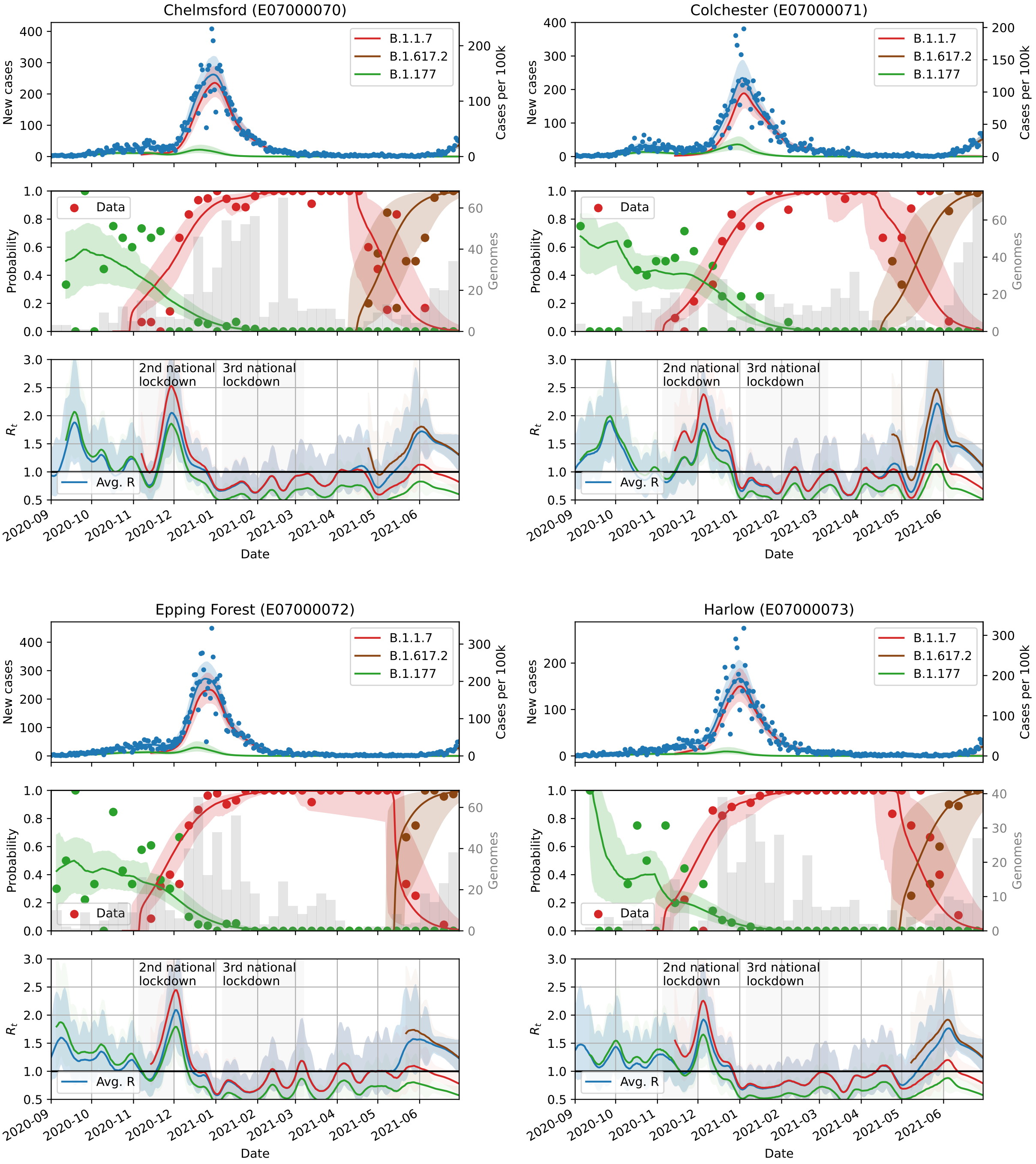

Chelmsford (E07000070)
Colchester (E07000071)
Epping Forest (E07000072)
Harlow (E07000073)
New cases
Cases per 100k
B.1.1.7
B.1.617.2
B.1.177
Probability
Genomes
Data
R_t
Avg. R
2nd national lockdown
3rd national lockdown
2020-09
2020-10
2020-11
2020-12
2021-01
2021-02
2021-03
2021-04
2021-05
2021-06
Date

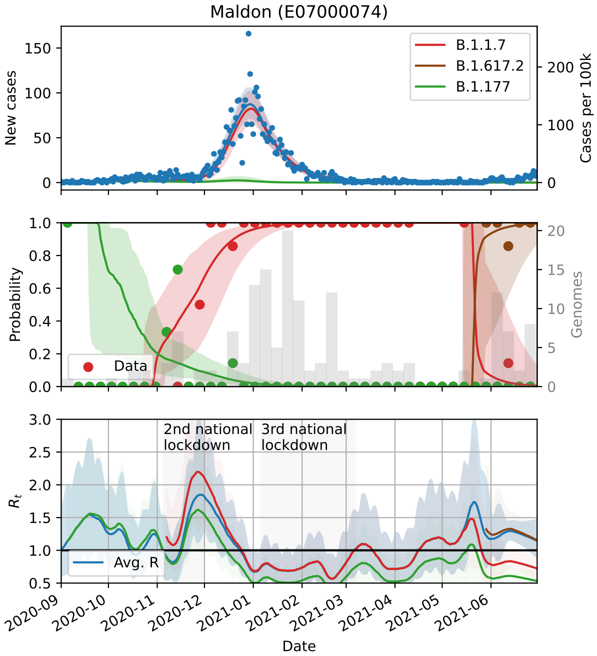
Maldon (E07000074)
B.1.1.7
B.1.617.2
B.1.177
New cases
Cases per 100k
Data
Probability
Genomes
2nd national lockdown
3rd national lockdown
Avg. R
R_t
Date

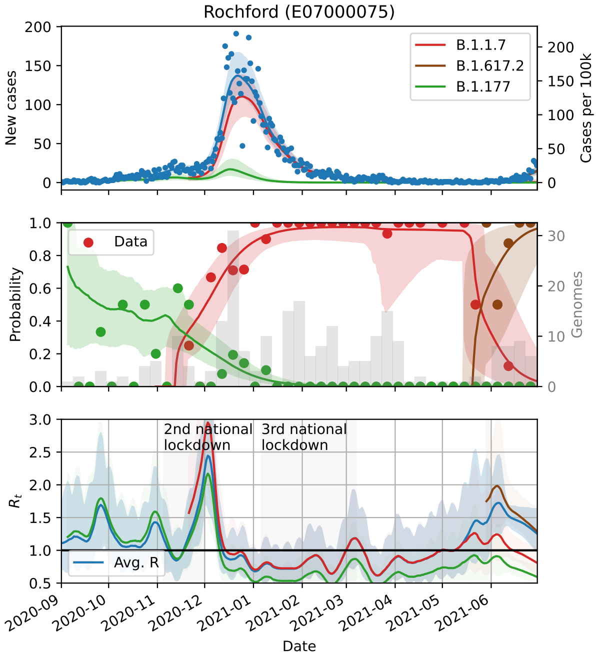
Rochford (E07000075)
B.1.1.7
B.1.617.2
B.1.177
New cases
Cases per 100k
Data
Probability
Genomes
2nd national lockdown
3rd national lockdown
Avg. R
R_t
Date

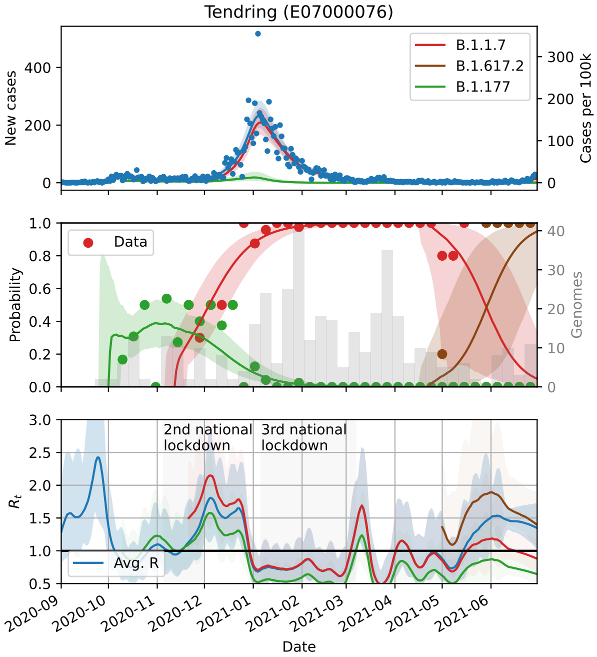
Tendring (E07000076)
B.1.1.7
B.1.617.2
B.1.177
New cases
Cases per 100k
Data
Probability
Genomes
2nd national lockdown
3rd national lockdown
Avg. R
R_t
Date

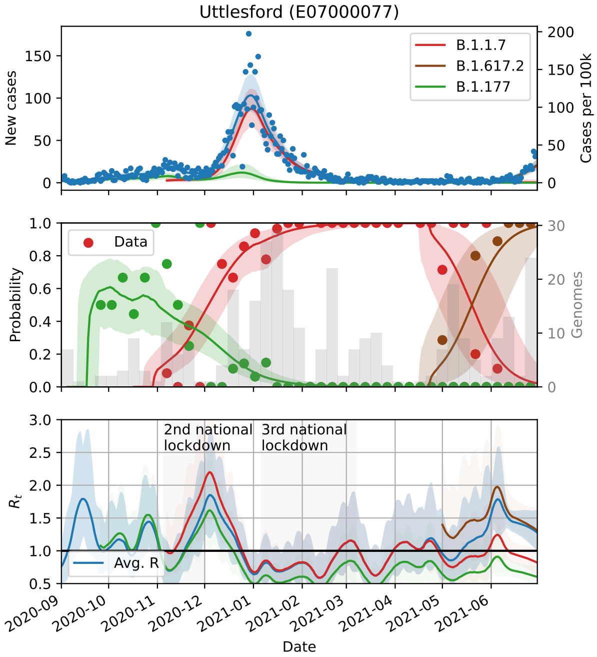
Uttlesford (E07000077)
B.1.1.7
B.1.617.2
B.1.177
New cases
Cases per 100k
Data
Probability
Genomes
2nd national lockdown
3rd national lockdown
Avg. R
R_t
Date

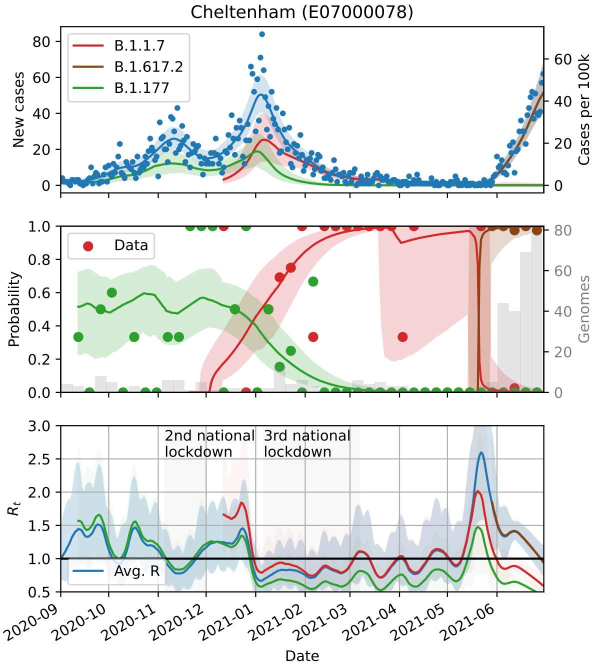
Cheltenham (E07000078)
B.1.1.7
B.1.617.2
B.1.177
New cases
Cases per 100k
Data
Probability
Genomes
2nd national lockdown
3rd national lockdown
Avg. R
R_t
Date

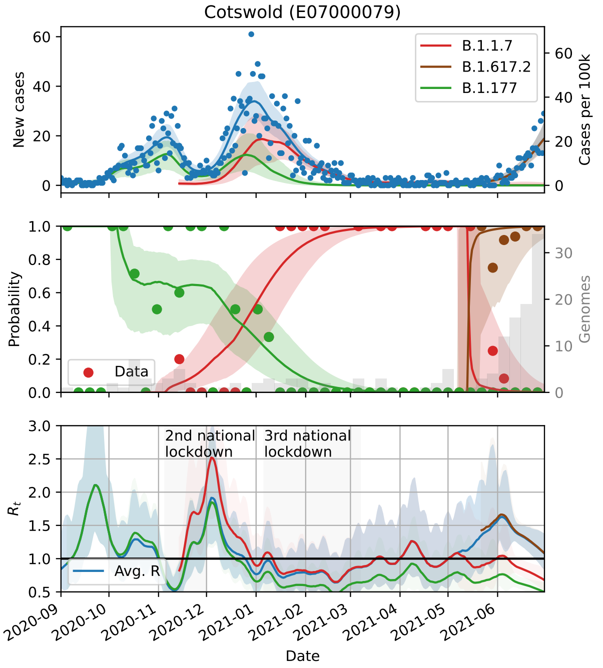
Cotswold (E07000079)
B.1.1.7
B.1.617.2
B.1.177
New cases
Cases per 100k
Data
Probability
Genomes
2nd national lockdown
3rd national lockdown
Avg. R
R_t
Date

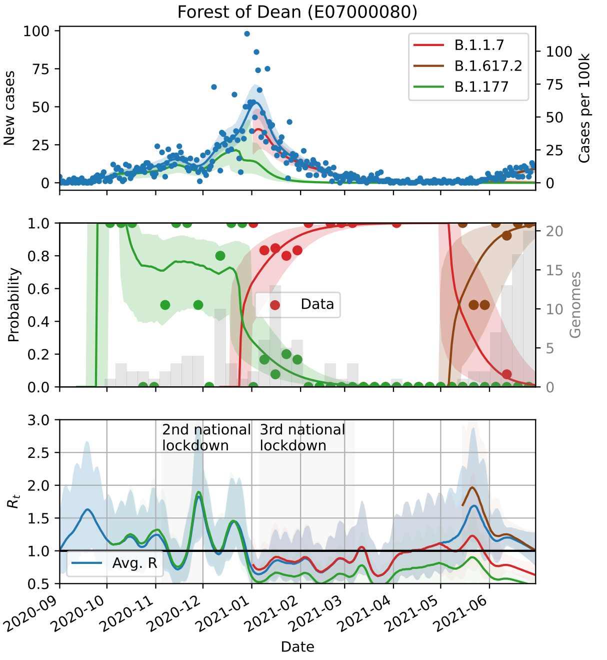
Forest of Dean (E07000080)
B.1.1.7
B.1.617.2
B.1.177
New cases
Cases per 100k
Data
Probability
Genomes
2nd national lockdown
3rd national lockdown
Avg. R
R_t
Date

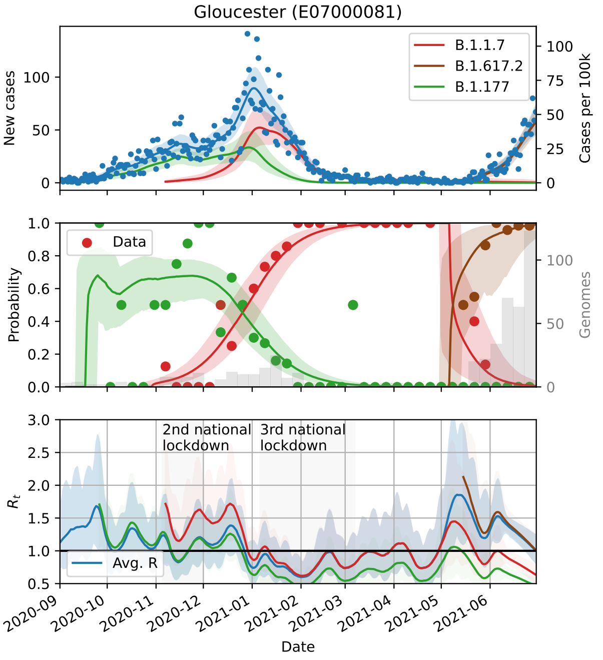
Gloucester (E07000081)
B.1.1.7
B.1.617.2
B.1.177
New cases
Cases per 100k
Data
Probability
Genomes
2nd national lockdown
3rd national lockdown
Avg. R
R_t
Date

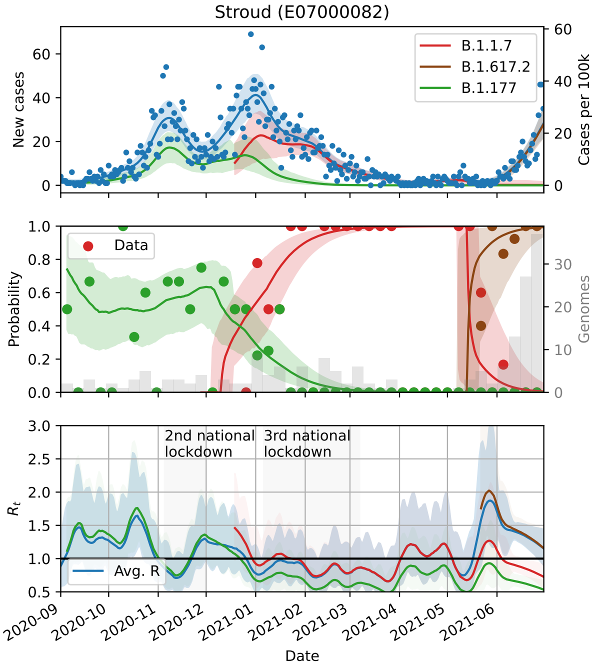

Stroud (E07000082)
B.1.1.7
B.1.617.2
B.1.177
New cases
Cases per 100k
Data
Probability
Genomes
2nd national lockdown
3rd national lockdown
Avg. R
R_t
Date

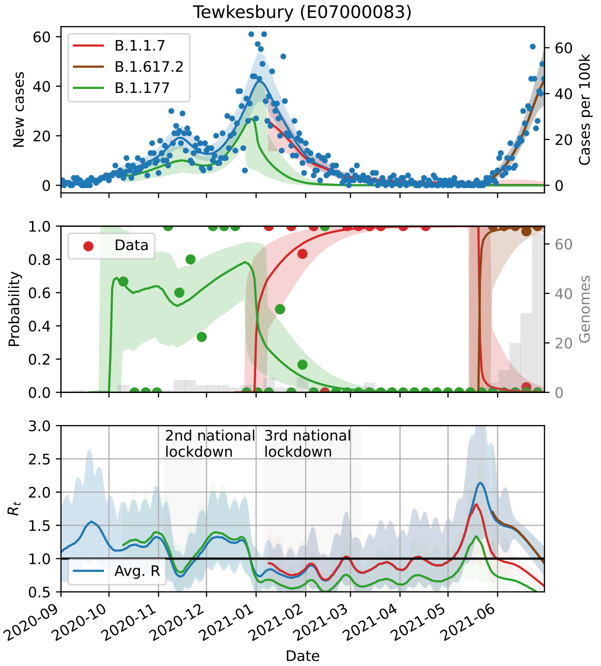

Tewkesbury (E07000083)
B.1.1.7
B.1.617.2
B.1.177
New cases
Cases per 100k
Data
Probability
Genomes
2nd national lockdown
3rd national lockdown
Avg. R
R_t
Date

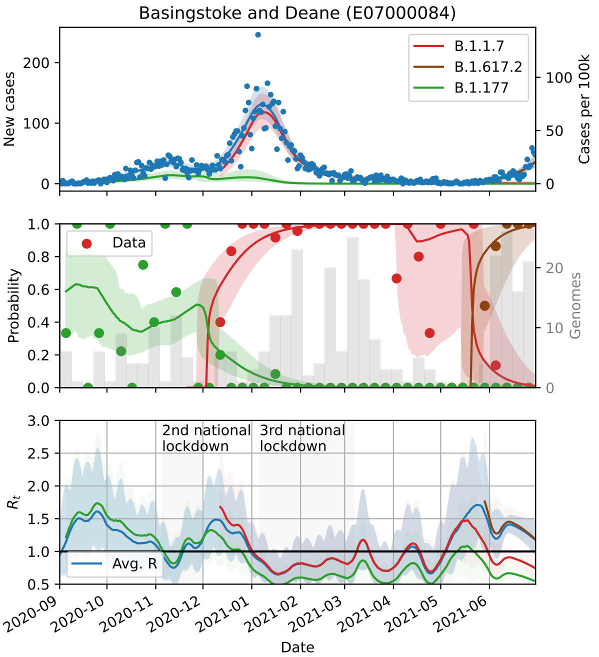

Basingstoke and Deane (E07000084)
B.1.1.7
B.1.617.2
B.1.177
New cases
Cases per 100k
Data
Probability
Genomes
2nd national lockdown
3rd national lockdown
Avg. R
R_t
Date

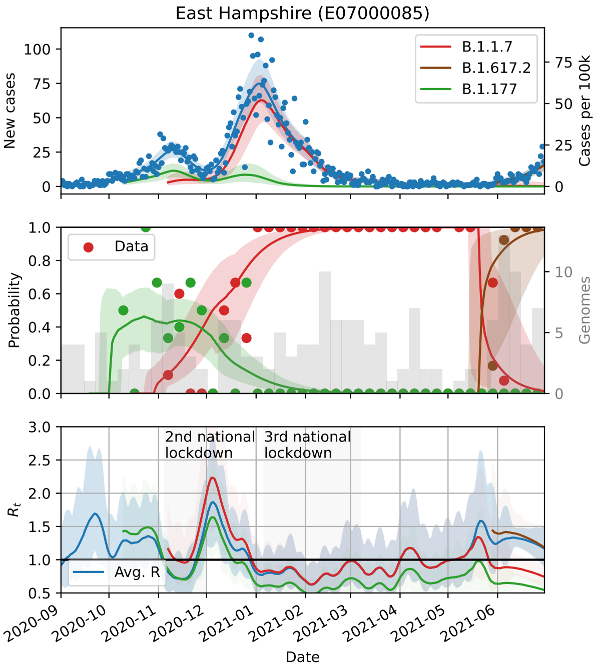

East Hampshire (E07000085)
B.1.1.7
B.1.617.2
B.1.177
New cases
Cases per 100k
Data
Probability
Genomes
2nd national lockdown
3rd national lockdown
Avg. R
R_t
Date

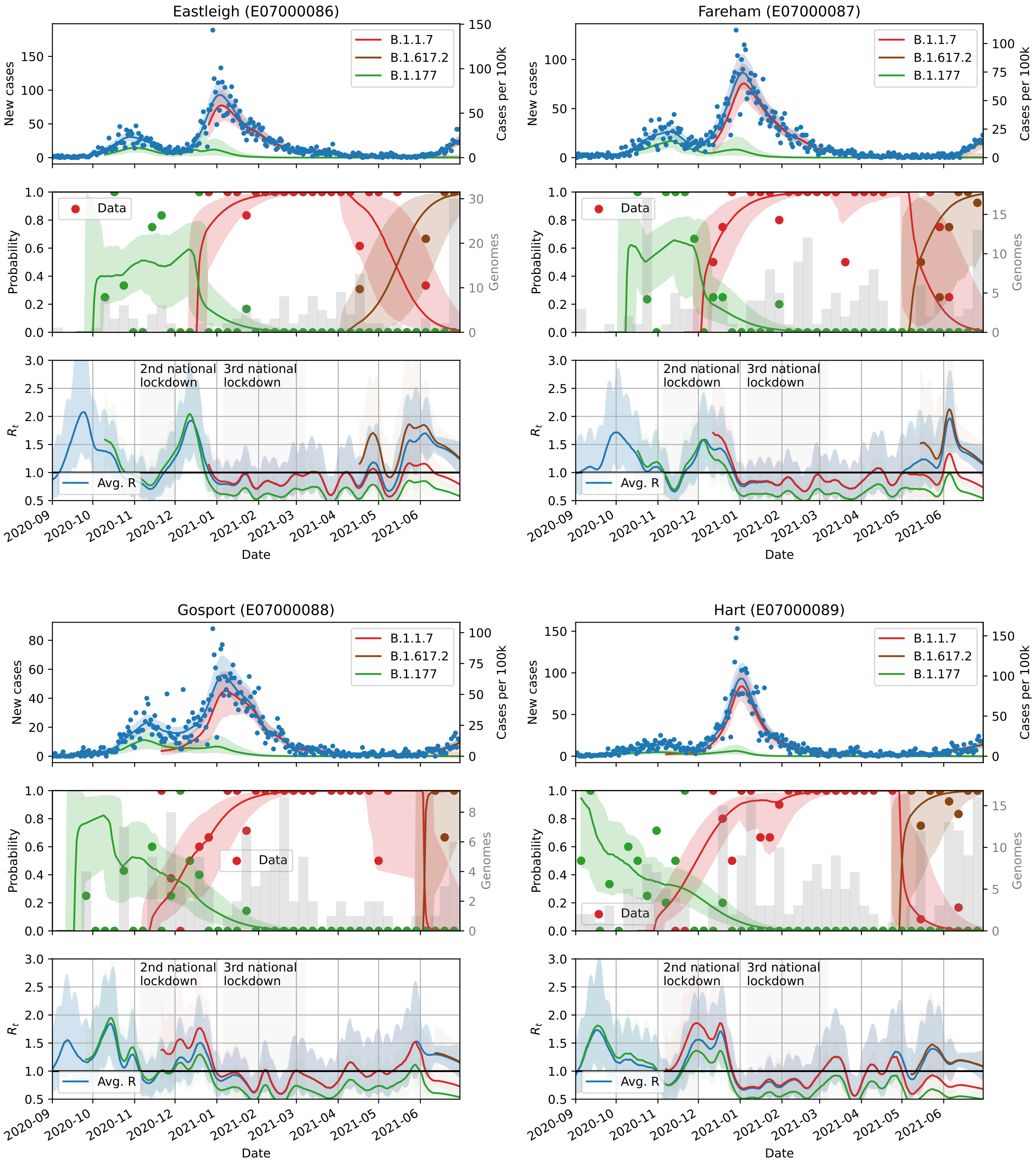
Eastleigh (E07000086)
Fareham (E07000087)
Gosport (E07000088)
Hart (E07000089)
B.1.1.7
B.1.617.2
B.1.177
New cases
Cases per 100k
Data
Probability
Genomes
2nd national lockdown
3rd national lockdown
Avg. R
R_t
Date

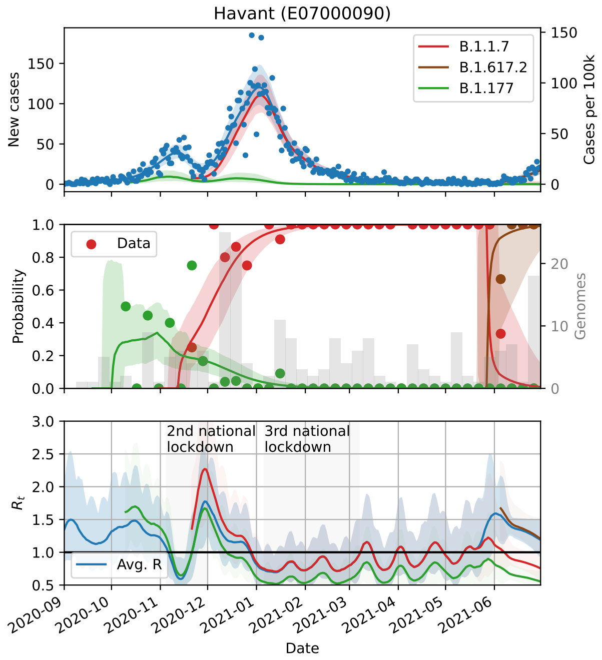
Havant (E07000090)
B.1.1.7
B.1.617.2
B.1.177
New cases
Cases per 100k
Data
Probability
Genomes
2nd national lockdown
3rd national lockdown
R_t
Avg. R
Date

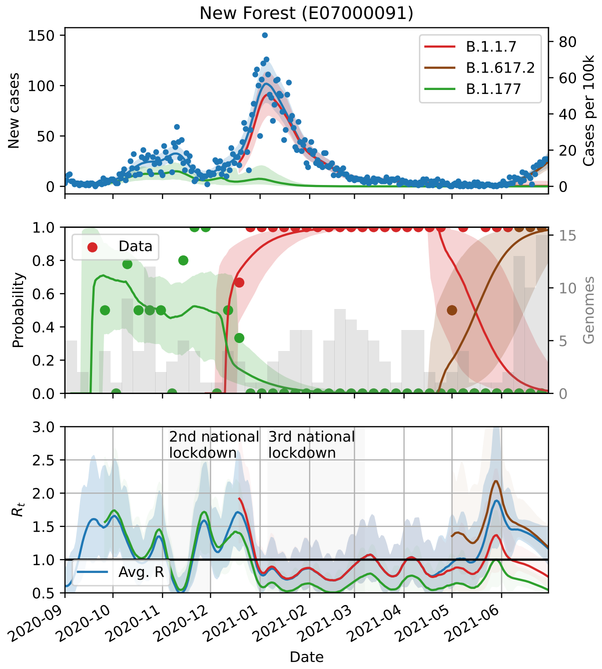
New Forest (E07000091)
B.1.1.7
B.1.617.2
B.1.177
New cases
Cases per 100k
Data
Probability
Genomes
2nd national lockdown
3rd national lockdown
R_t
Avg. R
Date

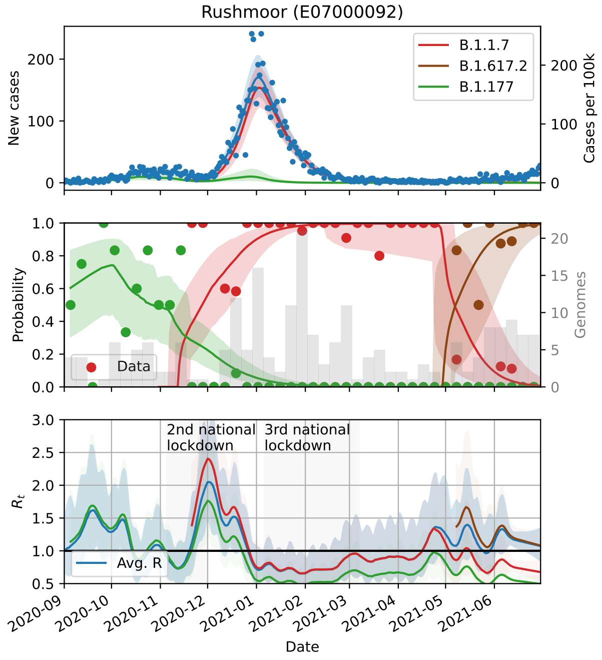
Rushmoor (E07000092)
B.1.1.7
B.1.617.2
B.1.177
New cases
Cases per 100k
Data
Probability
Genomes
2nd national lockdown
3rd national lockdown
R_t
Avg. R
Date

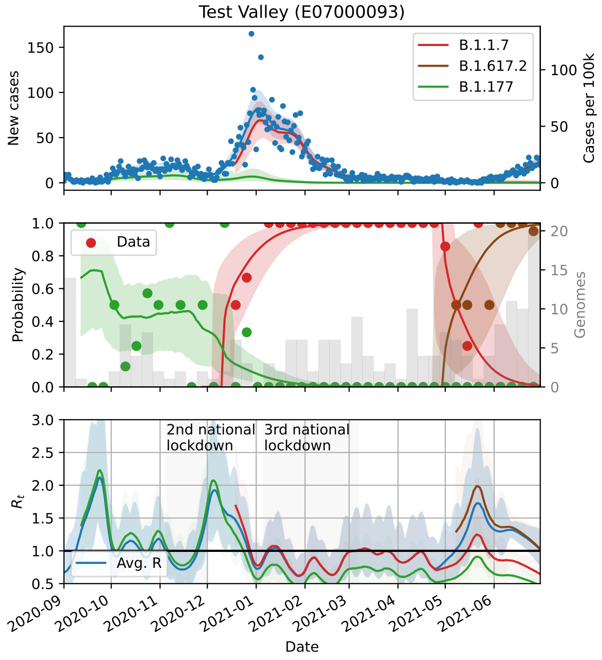
Test Valley (E07000093)
B.1.1.7
B.1.617.2
B.1.177
New cases
Cases per 100k
Data
Probability
Genomes
2nd national lockdown
3rd national lockdown
R_t
Avg. R
Date

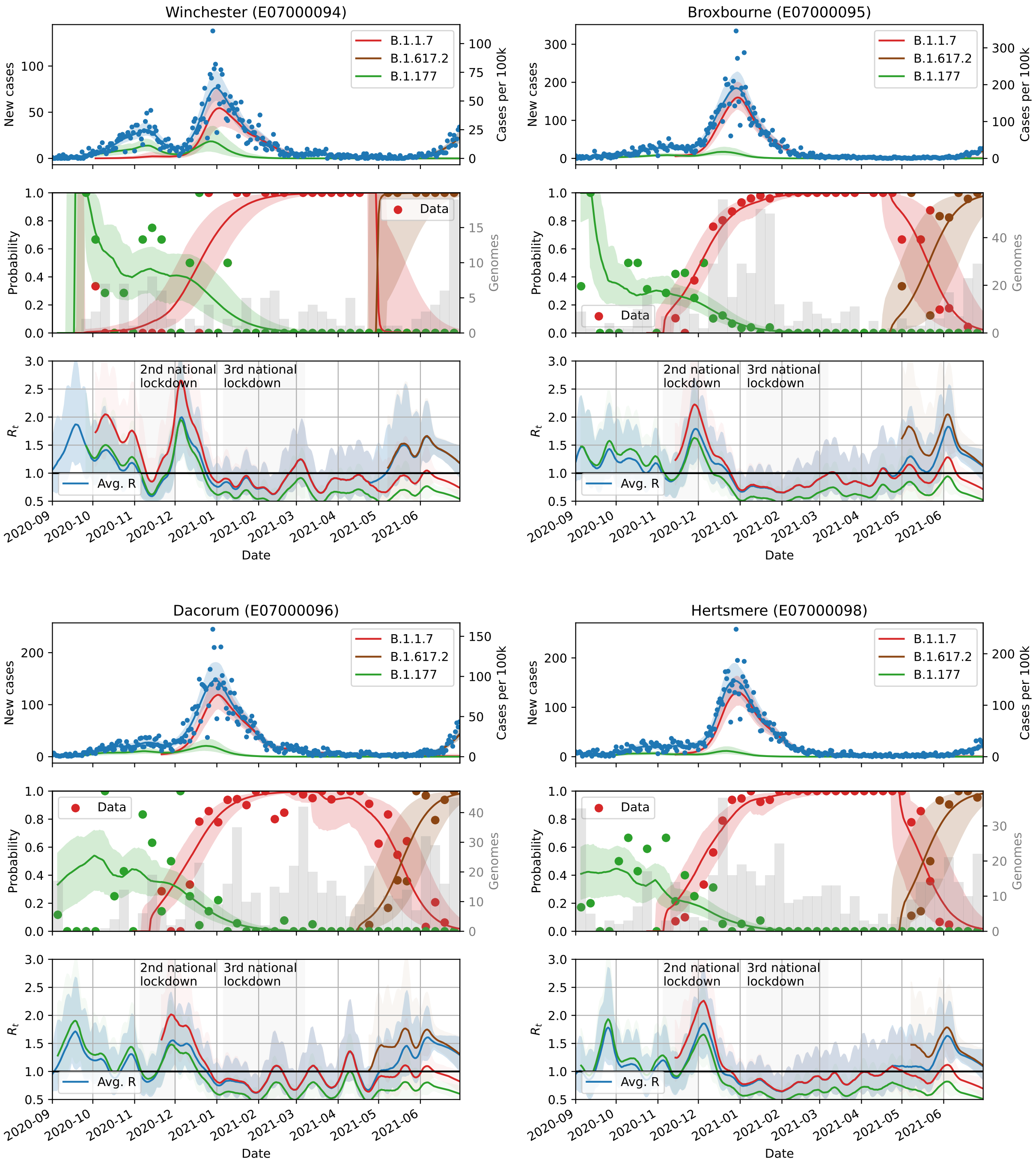

Winchester (E07000094)
Broxbourne (E07000095)
Dacorum (E07000096)
Hertsmere (E07000098)
B.1.1.7
B.1.617.2
B.1.177
Data
Avg. R
2nd national lockdown
3rd national lockdown
New cases
Cases per 100k
Probability
Genomes
Date

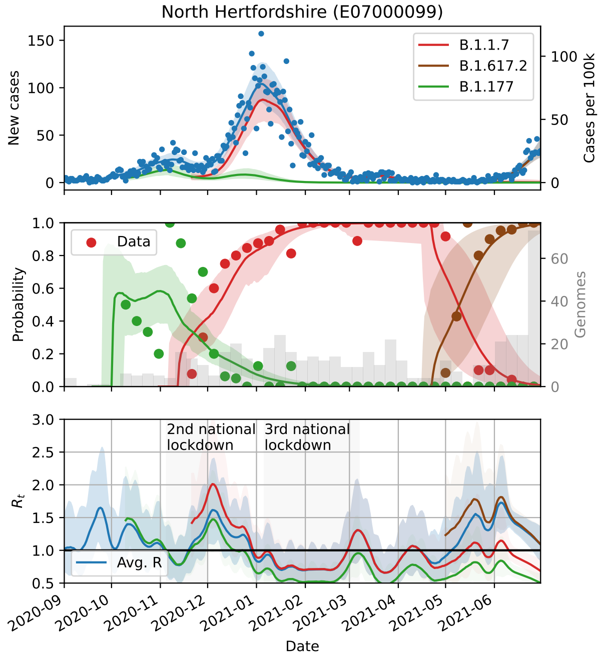
North Hertfordshire (E07000099)
B.1.1.7
B.1.617.2
B.1.177
New cases
Cases per 100k
Data
Probability
Genomes
2nd national lockdown
3rd national lockdown
Avg. R
R_t
Date
2020-09 2020-10 2020-11 2020-12 2021-01 2021-02 2021-03 2021-04 2021-05 2021-06

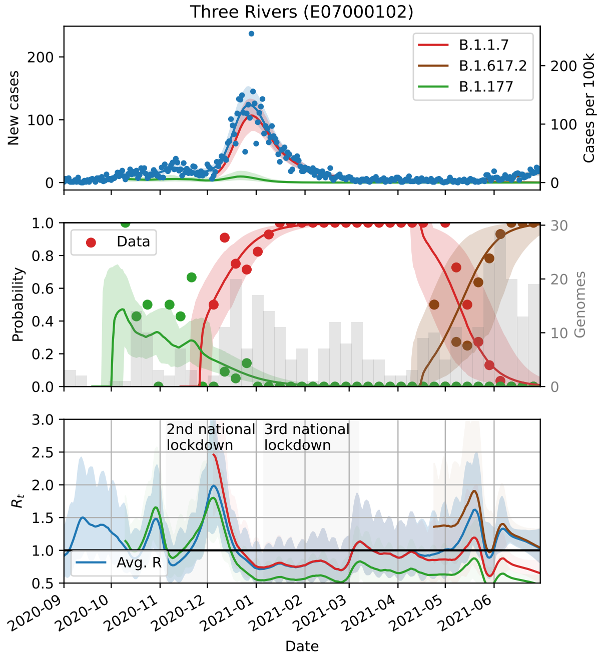
Three Rivers (E07000102)
B.1.1.7
B.1.617.2
B.1.177
New cases
Cases per 100k
Data
Probability
Genomes
2nd national lockdown
3rd national lockdown
Avg. R
R_t
Date
2020-09 2020-10 2020-11 2020-12 2021-01 2021-02 2021-03 2021-04 2021-05 2021-06

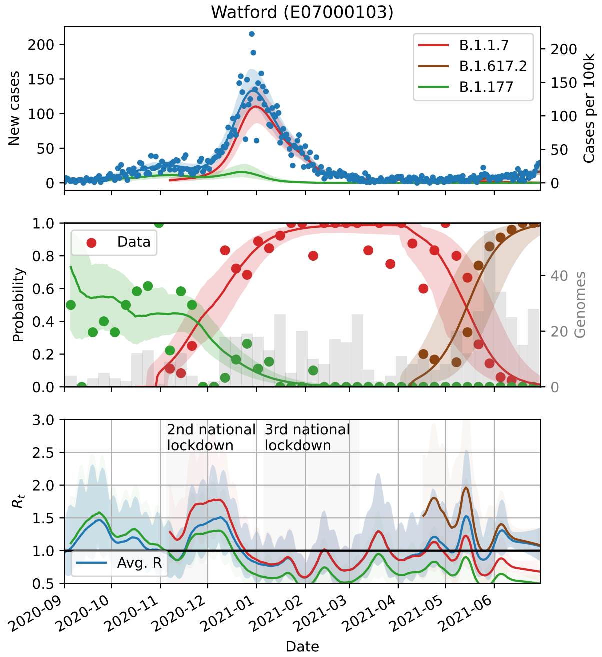
Watford (E07000103)
B.1.1.7
B.1.617.2
B.1.177
New cases
Cases per 100k
Data
Probability
Genomes
2nd national lockdown
3rd national lockdown
Avg. R
R_t
Date
2020-09 2020-10 2020-11 2020-12 2021-01 2021-02 2021-03 2021-04 2021-05 2021-06

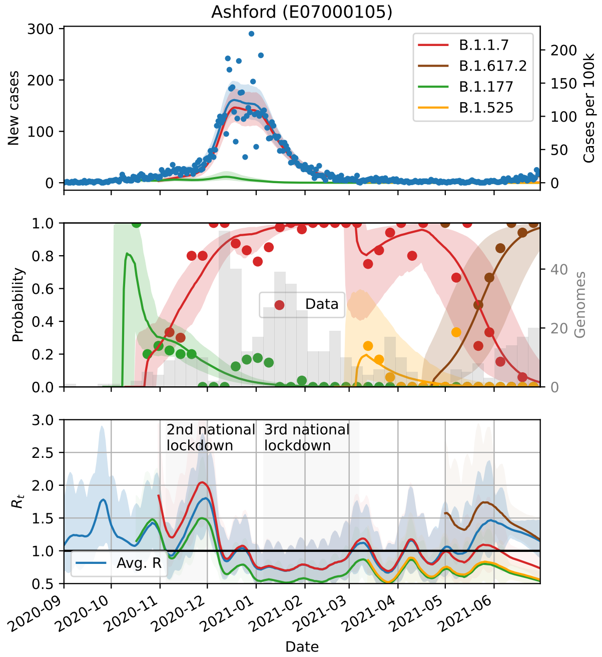
Ashford (E07000105)
B.1.1.7
B.1.617.2
B.1.177
B.1.525
New cases
Cases per 100k
Data
Probability
Genomes
2nd national lockdown
3rd national lockdown
Avg. R
R_t
Date
2020-09 2020-10 2020-11 2020-12 2021-01 2021-02 2021-03 2021-04 2021-05 2021-06

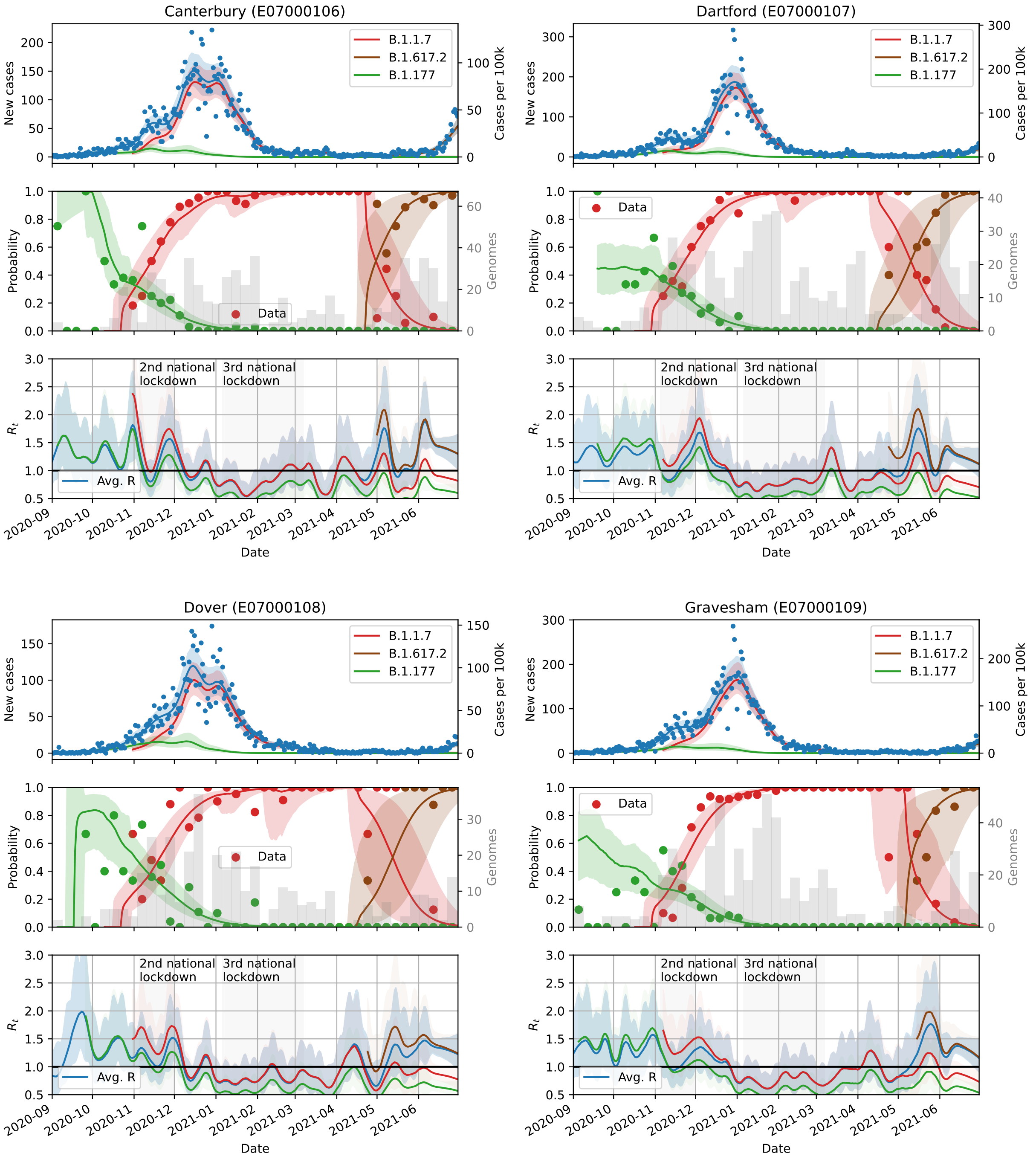
Canterbury (E07000106)
Dartford (E07000107)
Dover (E07000108)
Gravesham (E07000109)
B.1.1.7
B.1.617.2
B.1.177
New cases
Cases per 100k
Probability
Genomes
Data
R_t
Avg. R
2nd national lockdown
3rd national lockdown
Date
2020-09
2020-10
2020-11
2020-12
2021-01
2021-02
2021-03
2021-04
2021-05
2021-06

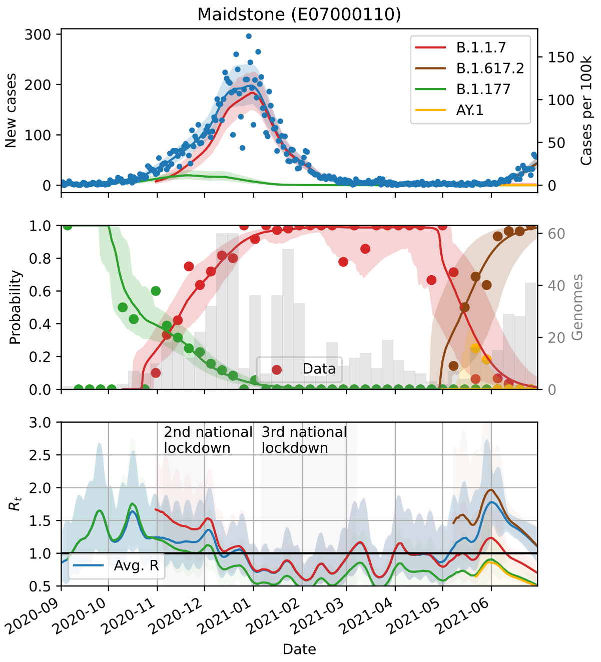
Maidstone (E07000110)
New cases
Cases per 100k
B.1.1.7
B.1.617.2
B.1.177
AY.1
Probability
Genomes
Data
R_t
2nd national lockdown
3rd national lockdown
Avg. R
Date

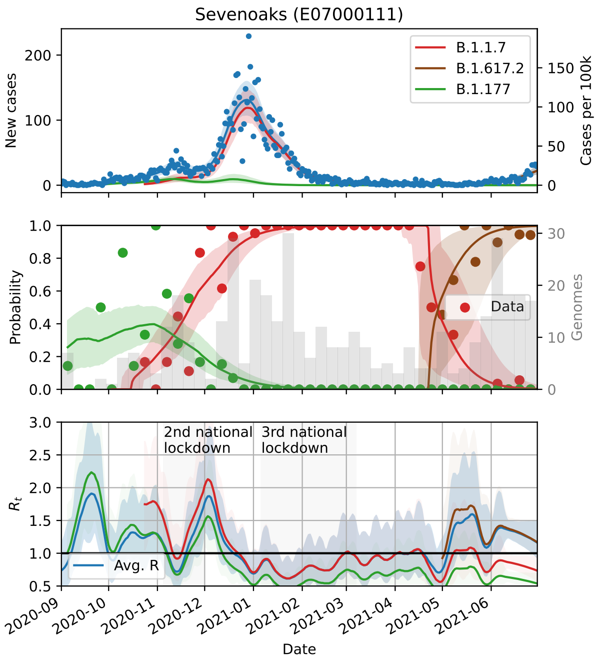
Sevenoaks (E07000111)
New cases
Cases per 100k
B.1.1.7
B.1.617.2
B.1.177
Probability
Genomes
Data
R_t
2nd national lockdown
3rd national lockdown
Avg. R
Date

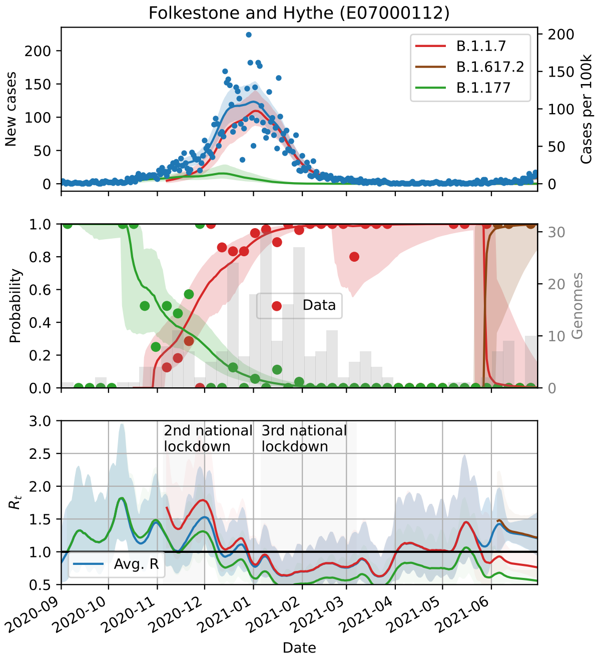
Folkestone and Hythe (E07000112)
New cases
Cases per 100k
B.1.1.7
B.1.617.2
B.1.177
Probability
Genomes
Data
R_t
2nd national lockdown
3rd national lockdown
Avg. R
Date

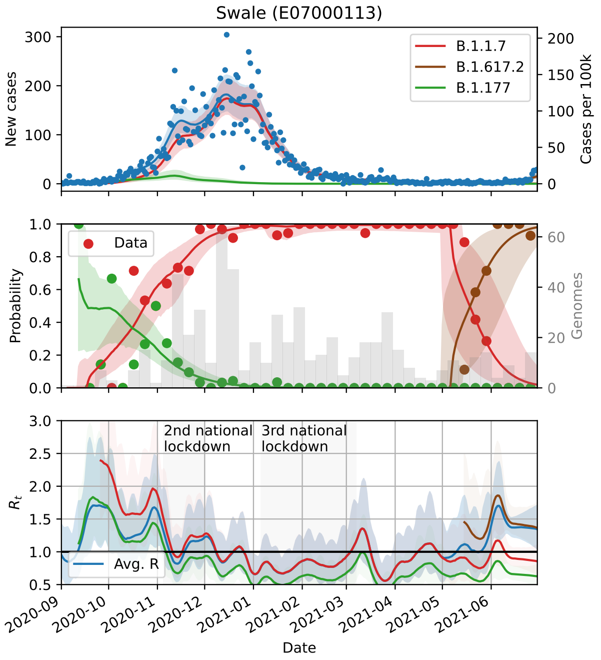
Swale (E07000113)
New cases
Cases per 100k
B.1.1.7
B.1.617.2
B.1.177
Probability
Genomes
Data
R_t
2nd national lockdown
3rd national lockdown
Avg. R
Date

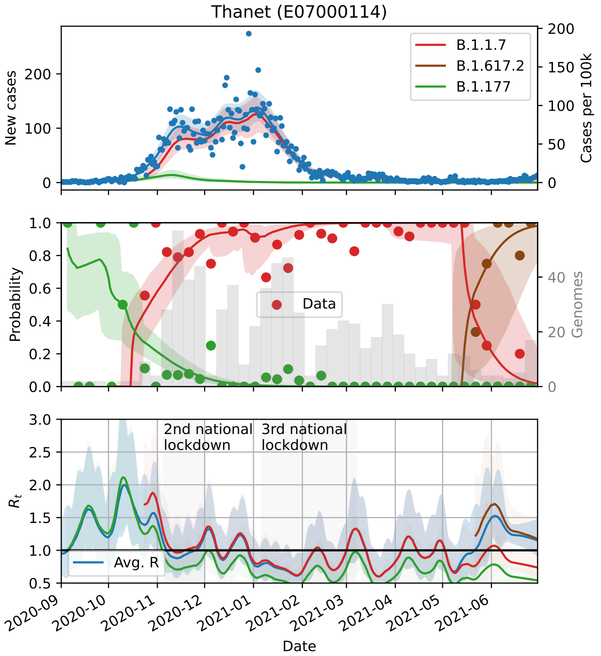
Thanet (E07000114)
B.1.1.7
B.1.617.2
B.1.177
New cases
Cases per 100k
Probability
Genomes
Data
R_t
2nd national lockdown
3rd national lockdown
Avg. R
Date

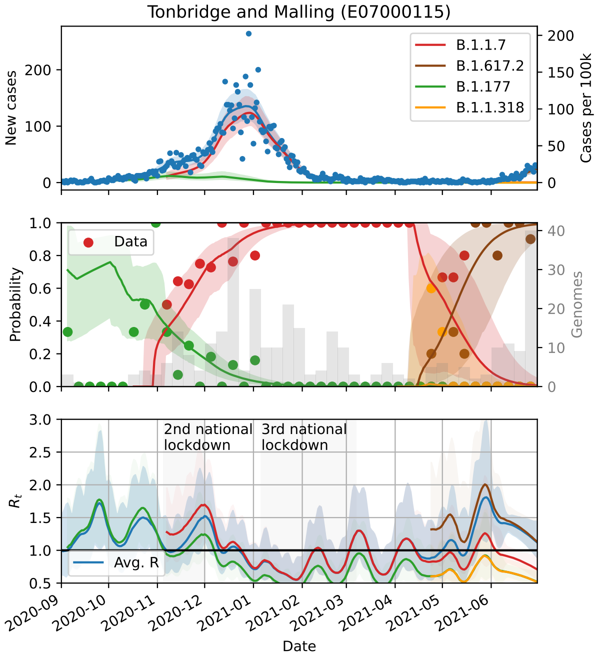
Tonbridge and Malling (E07000115)
B.1.1.7
B.1.617.2
B.1.177
B.1.1.318
New cases
Cases per 100k
Probability
Genomes
Data
R_t
2nd national lockdown
3rd national lockdown
Avg. R
Date

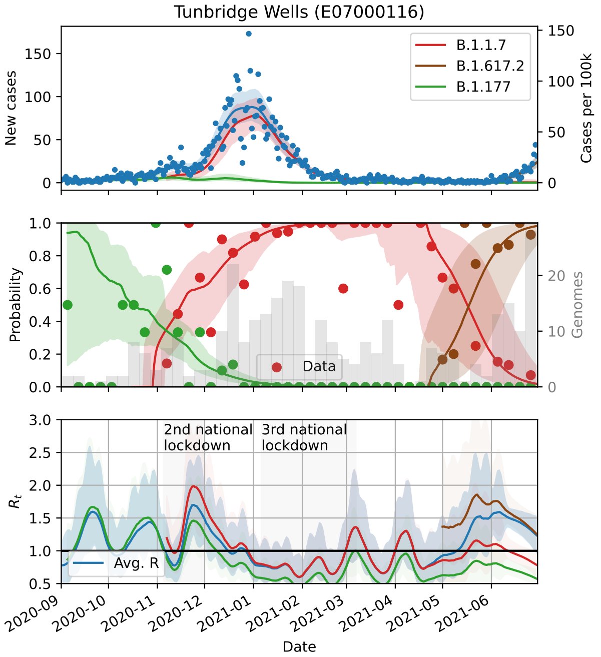
Tunbridge Wells (E07000116)
B.1.1.7
B.1.617.2
B.1.177
New cases
Cases per 100k
Probability
Genomes
Data
R_t
2nd national lockdown
3rd national lockdown
Avg. R
Date

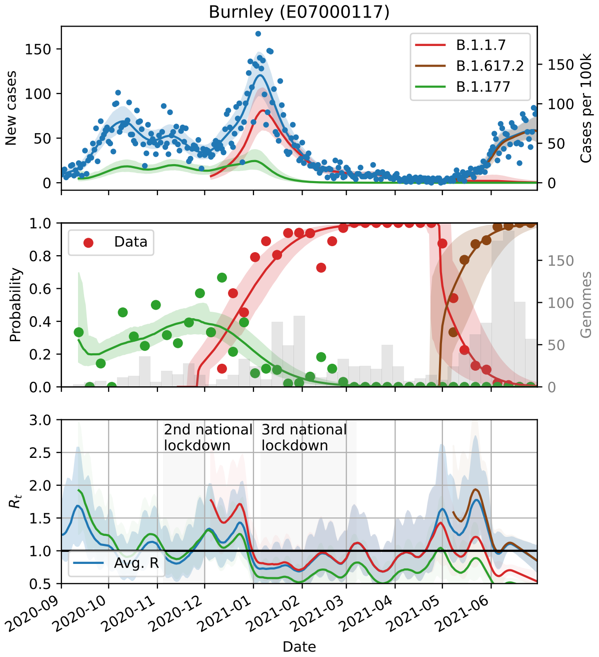
Burnley (E07000117)
B.1.1.7
B.1.617.2
B.1.177
New cases
Cases per 100k
Probability
Genomes
Data
R_t
2nd national lockdown
3rd national lockdown
Avg. R
Date

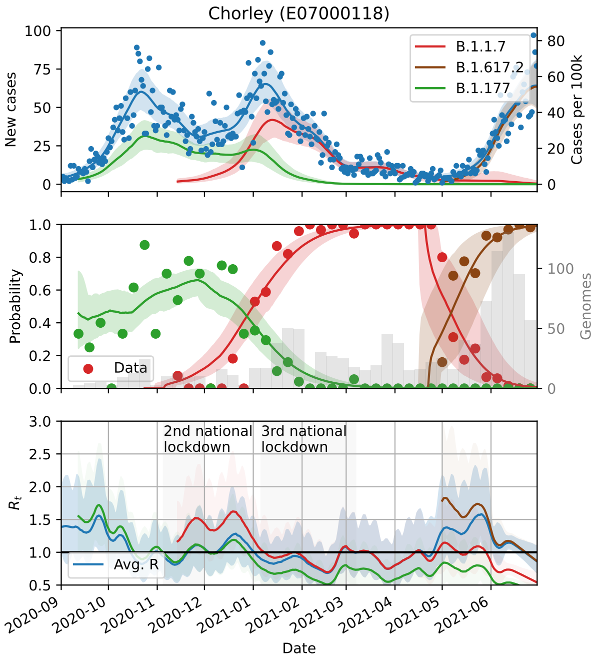
Chorley (E07000118)
New cases
Cases per 100k
B.1.1.7
B.1.617.2
B.1.177
Probability
Genomes
Data
R_t
2nd national lockdown
3rd national lockdown
Avg. R
Date

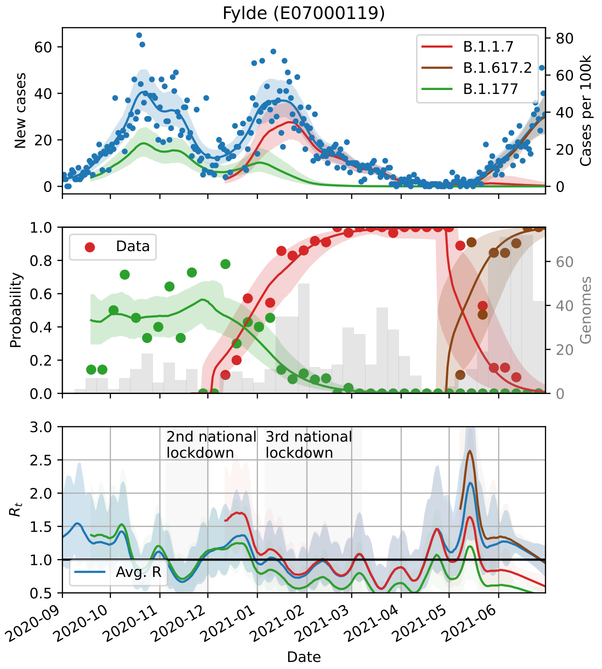
Fylde (E07000119)
New cases
Cases per 100k
B.1.1.7
B.1.617.2
B.1.177
Probability
Genomes
Data
R_t
2nd national lockdown
3rd national lockdown
Avg. R
Date

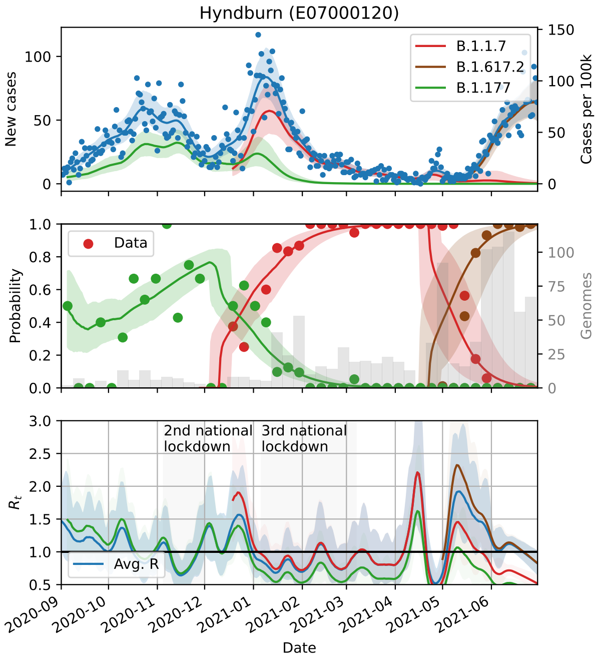
Hyndburn (E07000120)
New cases
Cases per 100k
B.1.1.7
B.1.617.2
B.1.177
Probability
Genomes
Data
R_t
2nd national lockdown
3rd national lockdown
Avg. R
Date

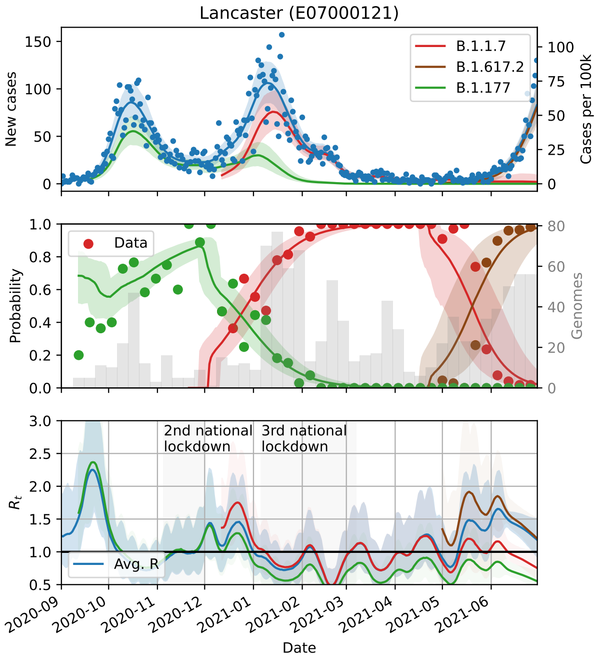
Lancaster (E07000121)
New cases
Cases per 100k
B.1.1.7
B.1.617.2
B.1.177
Probability
Genomes
Data
R_t
2nd national lockdown
3rd national lockdown
Avg. R
Date

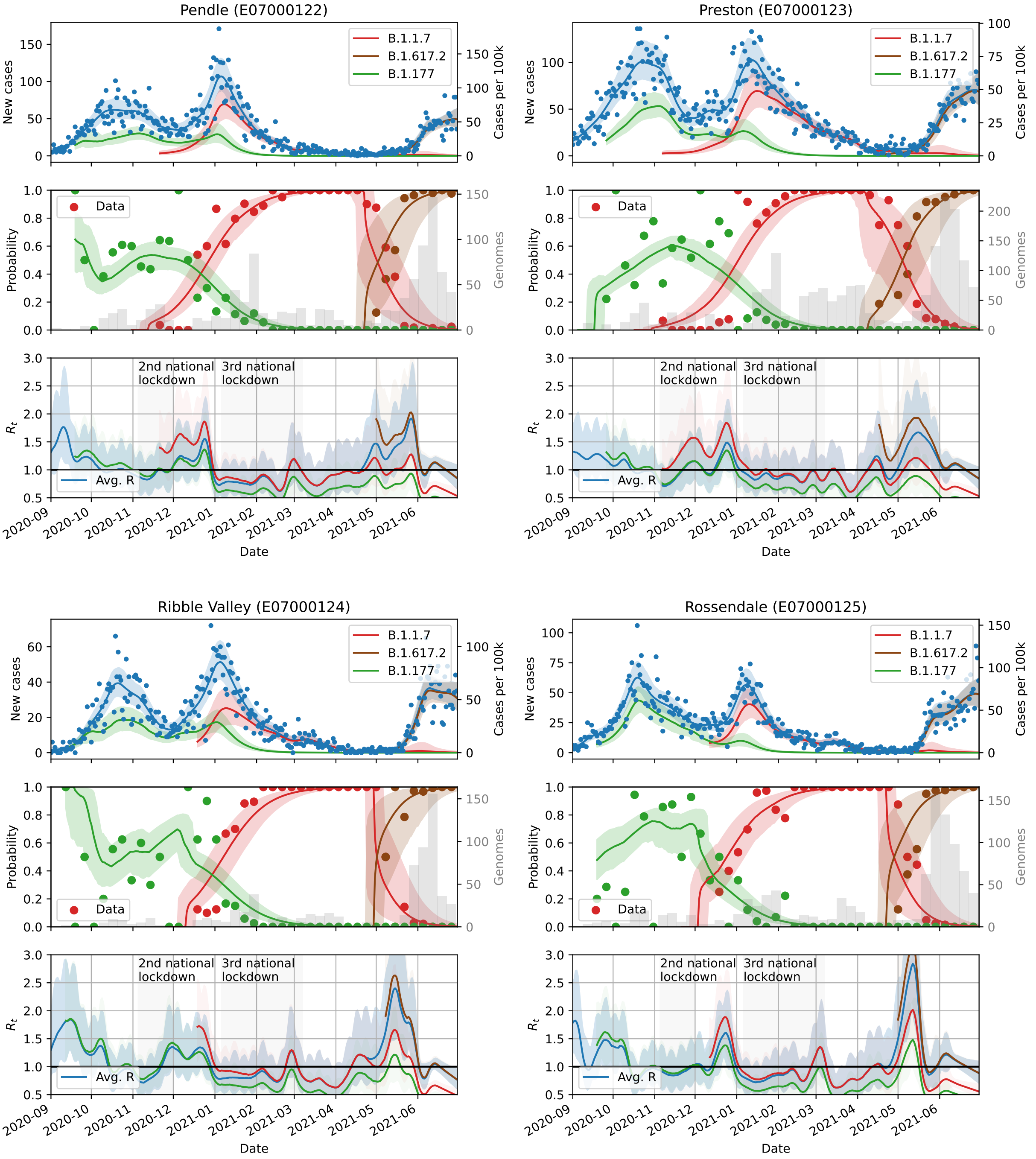
Pendle (E07000122)
Preston (E07000123)
Ribble Valley (E07000124)
Rossendale (E07000125)
B.1.1.7
B.1.617.2
B.1.177
New cases
Cases per 100k
Data
Probability
Genomes
2nd national lockdown
3rd national lockdown
R_t
Avg. R
2020-09
2020-10
2020-11
2020-12
2021-01
2021-02
2021-03
2021-04
2021-05
2021-06
Date

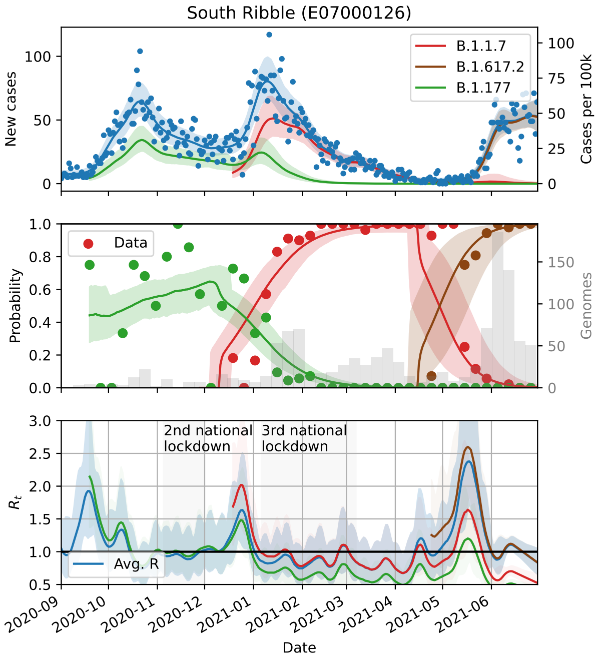
South Ribble (E07000126)
B.1.1.7
B.1.617.2
B.1.177
New cases
Cases per 100k
Data
Probability
Genomes
2nd national lockdown
3rd national lockdown
Avg. R
R_t
Date

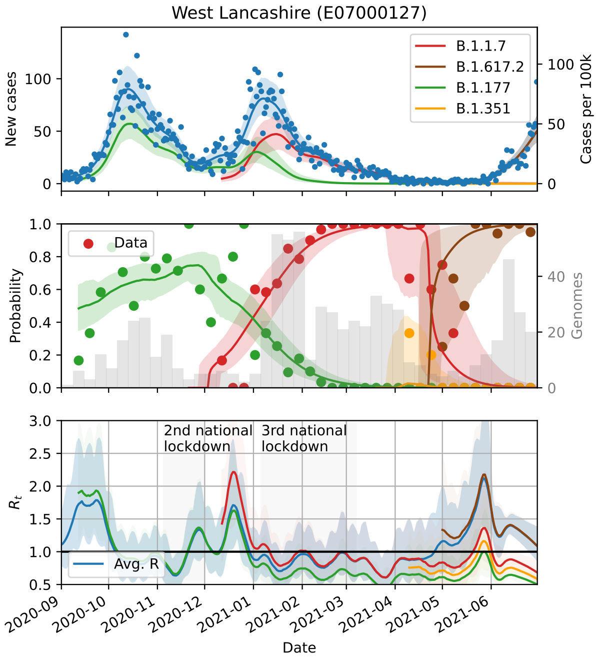
West Lancashire (E07000127)
B.1.1.7
B.1.617.2
B.1.177
B.1.351
New cases
Cases per 100k
Data
Probability
Genomes
2nd national lockdown
3rd national lockdown
Avg. R
R_t
Date

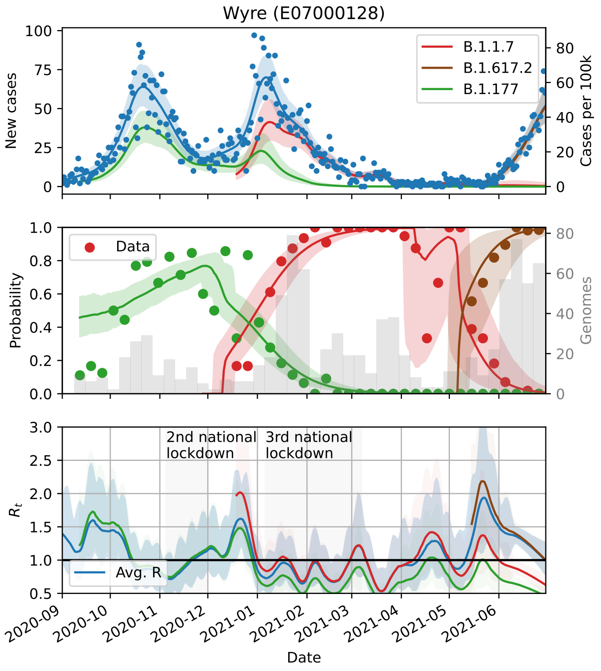
Wyre (E07000128)
B.1.1.7
B.1.617.2
B.1.177
New cases
Cases per 100k
Data
Probability
Genomes
2nd national lockdown
3rd national lockdown
Avg. R
R_t
Date

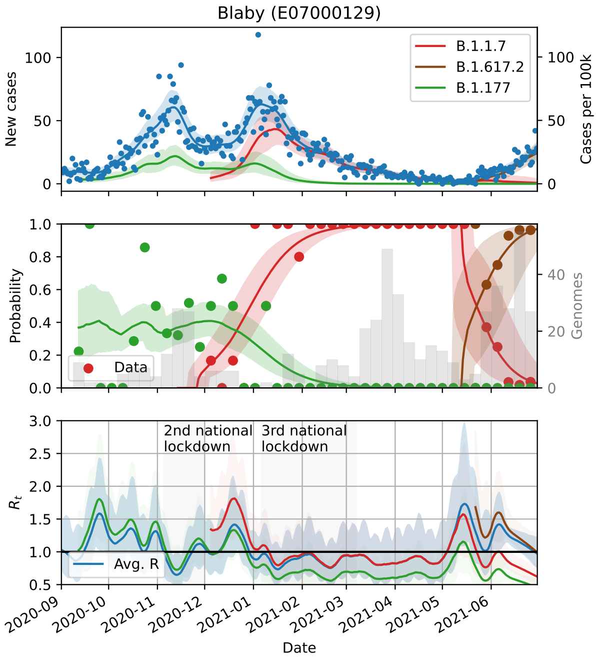
Blaby (E07000129)
B.1.1.7
B.1.617.2
B.1.177
New cases
Cases per 100k
Data
Probability
Genomes
2nd national lockdown
3rd national lockdown
Avg. R
R_t
Date

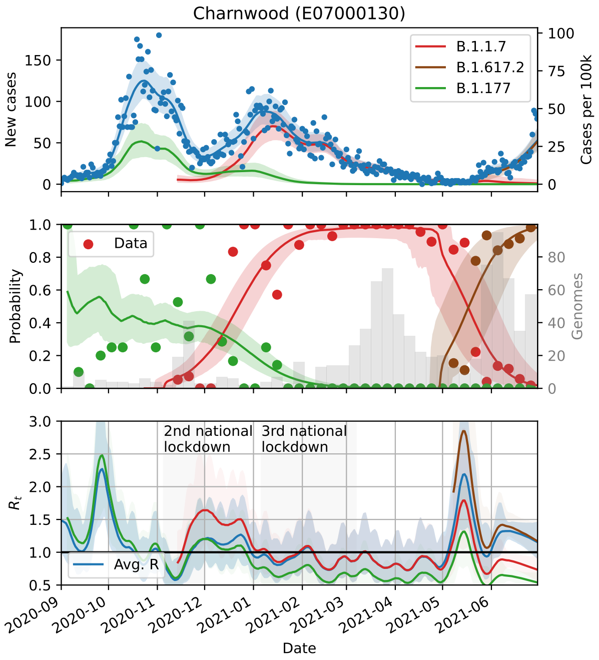
Charnwood (E07000130)
New cases
Cases per 100k
B.1.1.7
B.1.617.2
B.1.177
Probability
Data
Genomes
R_t
2nd national lockdown
3rd national lockdown
Avg. R
Date

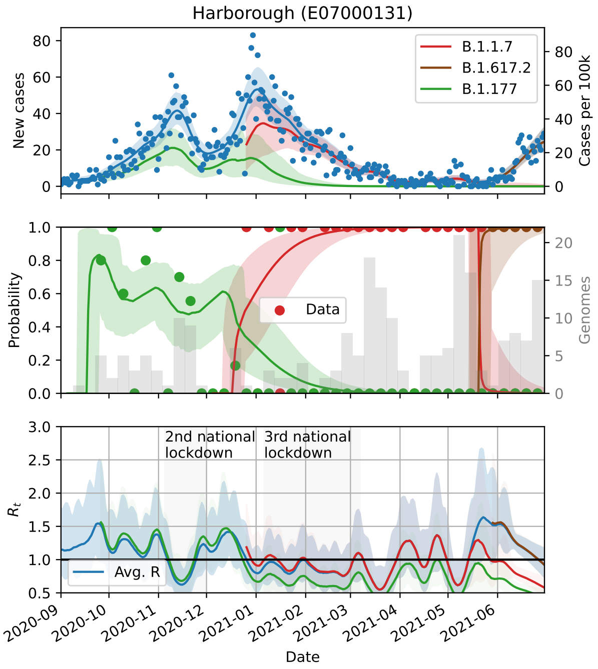
Harborough (E07000131)
New cases
Cases per 100k
B.1.1.7
B.1.617.2
B.1.177
Probability
Data
Genomes
R_t
2nd national lockdown
3rd national lockdown
Avg. R
Date

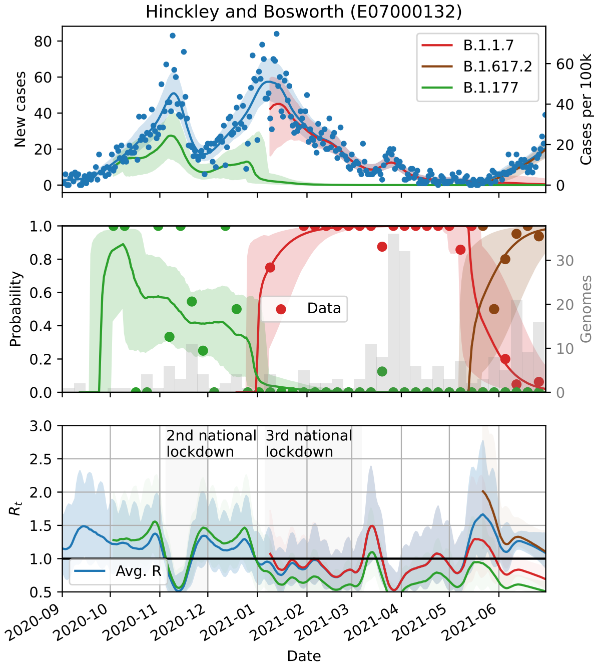
Hinckley and Bosworth (E07000132)
New cases
Cases per 100k
B.1.1.7
B.1.617.2
B.1.177
Probability
Data
Genomes
R_t
2nd national lockdown
3rd national lockdown
Avg. R
Date

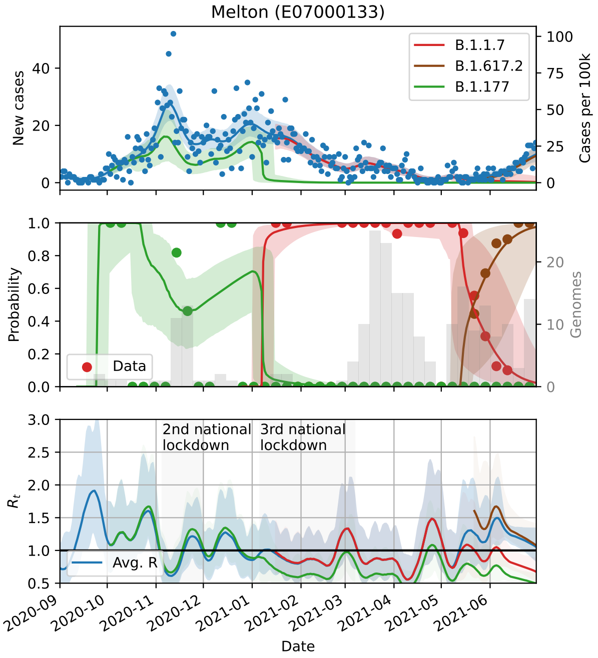
Melton (E07000133)
New cases
Cases per 100k
B.1.1.7
B.1.617.2
B.1.177
Probability
Data
Genomes
R_t
2nd national lockdown
3rd national lockdown
Avg. R
Date

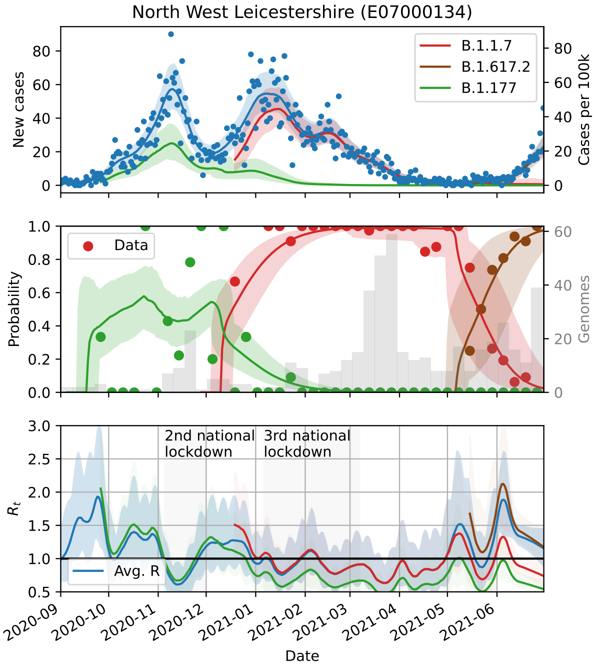
North West Leicestershire (E07000134)
New cases
Cases per 100k
B.1.1.7
B.1.617.2
B.1.177
Data
Probability
Genomes
2nd national lockdown
3rd national lockdown
R_t
Avg. R
2020-09
2020-10
2020-11
2020-12
2021-01
2021-02
2021-03
2021-04
2021-05
2021-06
Date

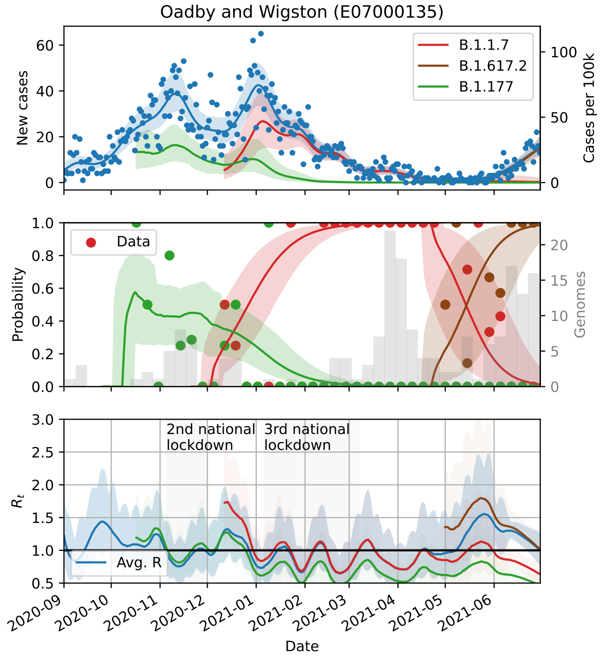
Oadby and Wigston (E07000135)
New cases
Cases per 100k
B.1.1.7
B.1.617.2
B.1.177
Data
Probability
Genomes
2nd national lockdown
3rd national lockdown
R_t
Avg. R
2020-09
2020-10
2020-11
2020-12
2021-01
2021-02
2021-03
2021-04
2021-05
2021-06
Date

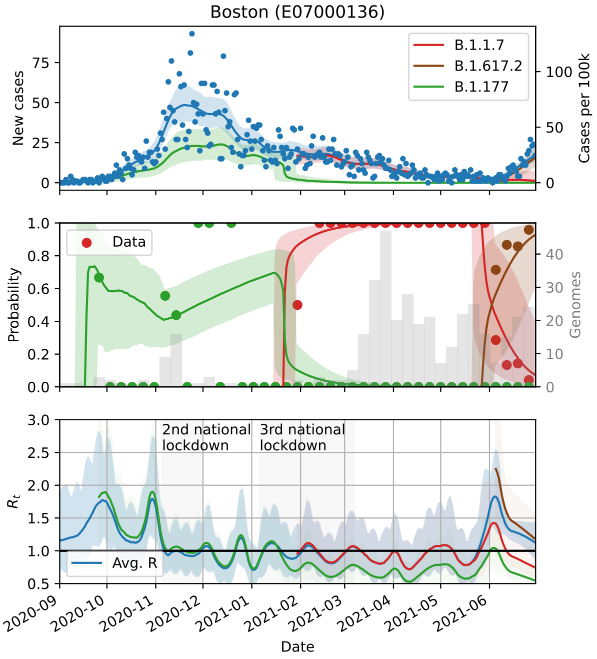
Boston (E07000136)
New cases
Cases per 100k
B.1.1.7
B.1.617.2
B.1.177
Data
Probability
Genomes
2nd national lockdown
3rd national lockdown
R_t
Avg. R
2020-09
2020-10
2020-11
2020-12
2021-01
2021-02
2021-03
2021-04
2021-05
2021-06
Date

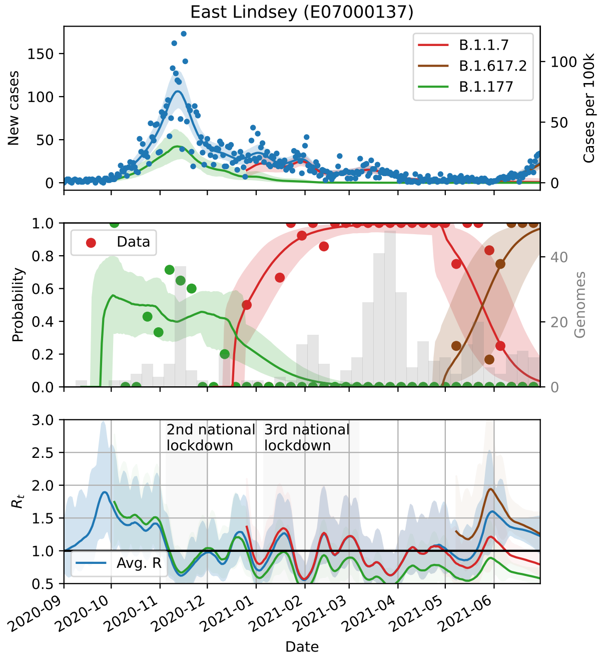
East Lindsey (E07000137)
New cases
Cases per 100k
B.1.1.7
B.1.617.2
B.1.177
Data
Probability
Genomes
2nd national lockdown
3rd national lockdown
R_t
Avg. R
2020-09
2020-10
2020-11
2020-12
2021-01
2021-02
2021-03
2021-04
2021-05
2021-06
Date

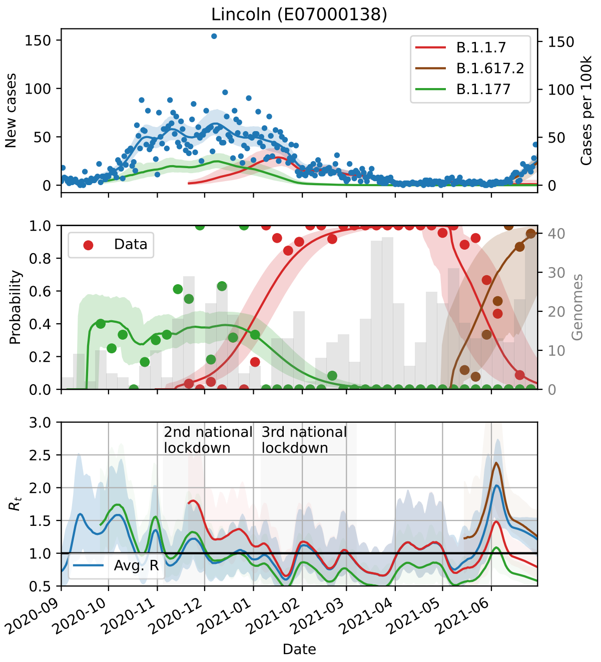
Lincoln (E07000138)
B.1.1.7
B.1.617.2
B.1.177
New cases
Cases per 100k
Data
Probability
Genomes
2nd national lockdown
3rd national lockdown
Avg. R
R_t
Date

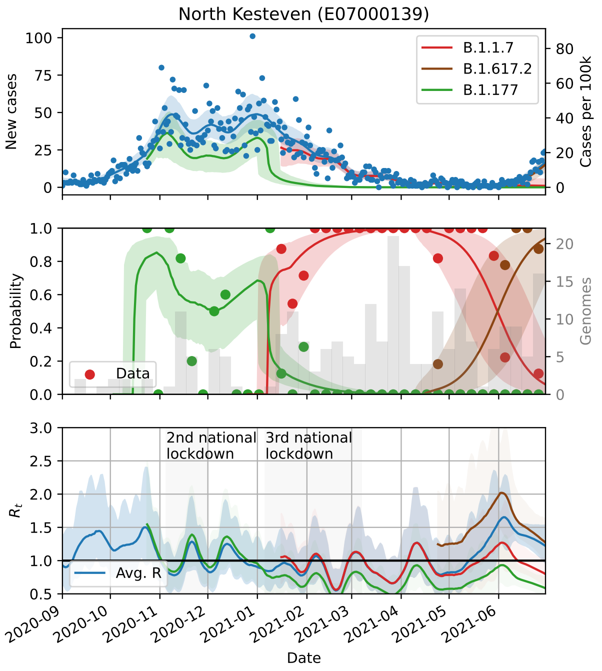
North Kesteven (E07000139)
B.1.1.7
B.1.617.2
B.1.177
New cases
Cases per 100k
Data
Probability
Genomes
2nd national lockdown
3rd national lockdown
Avg. R
R_t
Date

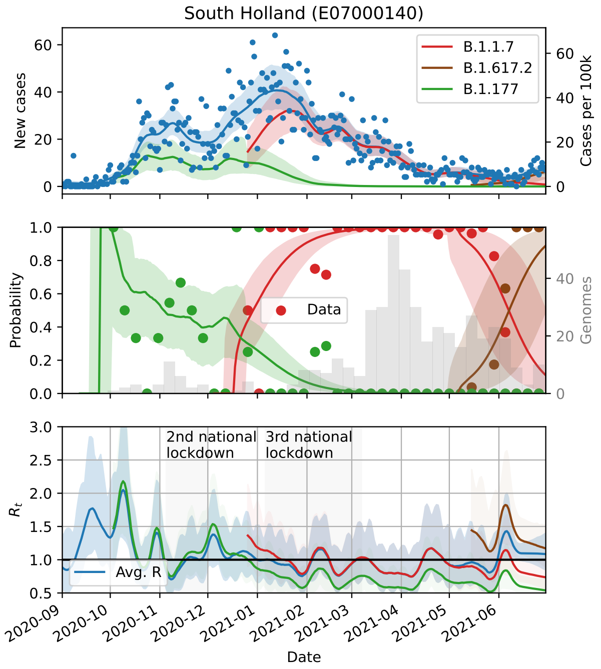
South Holland (E07000140)
B.1.1.7
B.1.617.2
B.1.177
New cases
Cases per 100k
Data
Probability
Genomes
2nd national lockdown
3rd national lockdown
Avg. R
R_t
Date

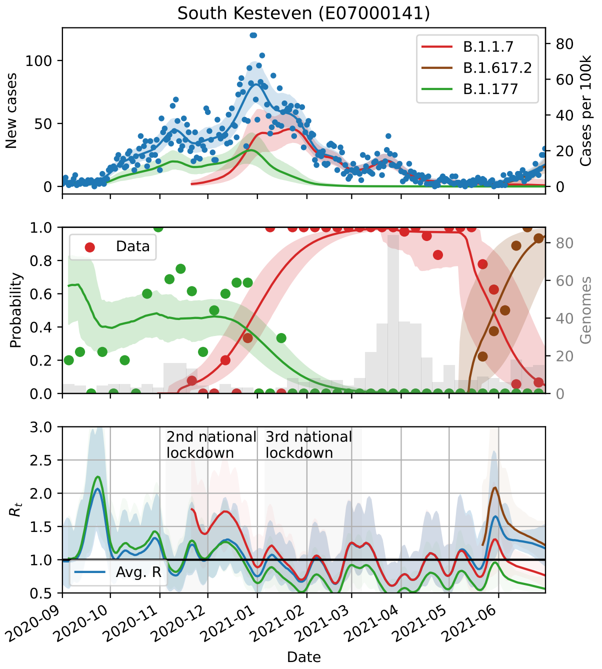
South Kesteven (E07000141)
B.1.1.7
B.1.617.2
B.1.177
New cases
Cases per 100k
Data
Probability
Genomes
2nd national lockdown
3rd national lockdown
Avg. R
R_t
Date

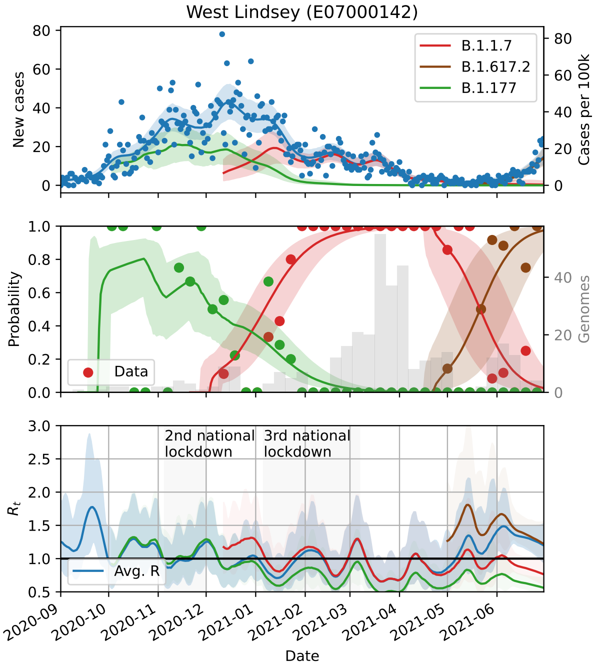
West Lindsey (E07000142)
B.1.1.7
B.1.617.2
B.1.177
New cases
Cases per 100k
Probability
Genomes
Data
2nd national lockdown
3rd national lockdown
Avg. R
R_t
Date

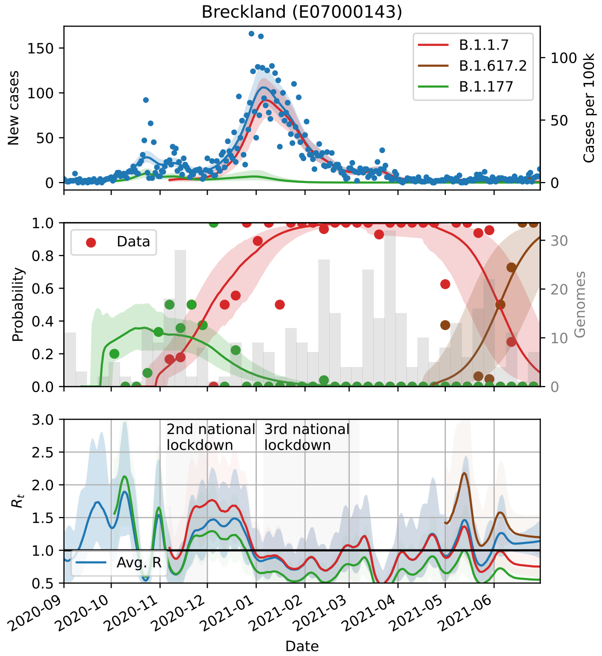
Breckland (E07000143)
B.1.1.7
B.1.617.2
B.1.177
New cases
Cases per 100k
Probability
Genomes
Data
2nd national lockdown
3rd national lockdown
Avg. R
R_t
Date

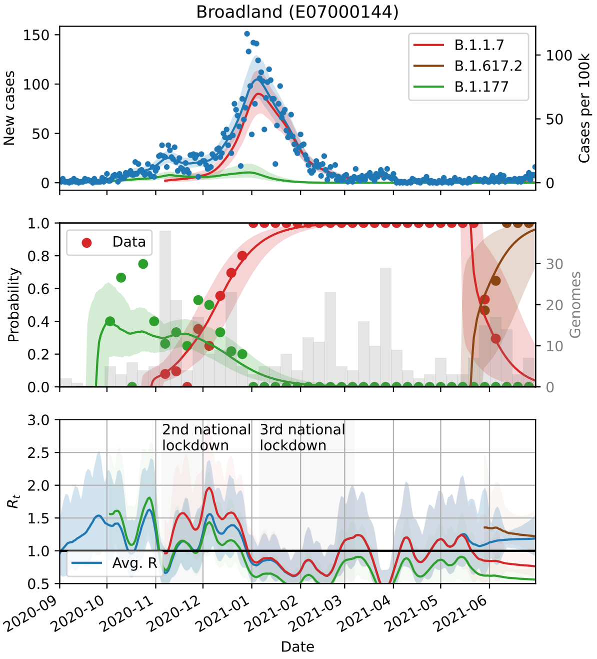
Broadland (E07000144)
B.1.1.7
B.1.617.2
B.1.177
New cases
Cases per 100k
Probability
Genomes
Data
2nd national lockdown
3rd national lockdown
Avg. R
R_t
Date

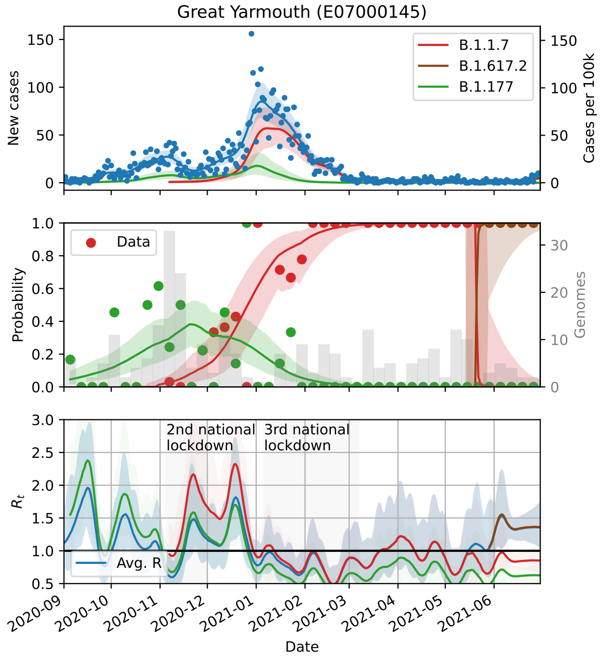
Great Yarmouth (E07000145)
B.1.1.7
B.1.617.2
B.1.177
New cases
Cases per 100k
Probability
Genomes
Data
2nd national lockdown
3rd national lockdown
Avg. R
R_t
Date

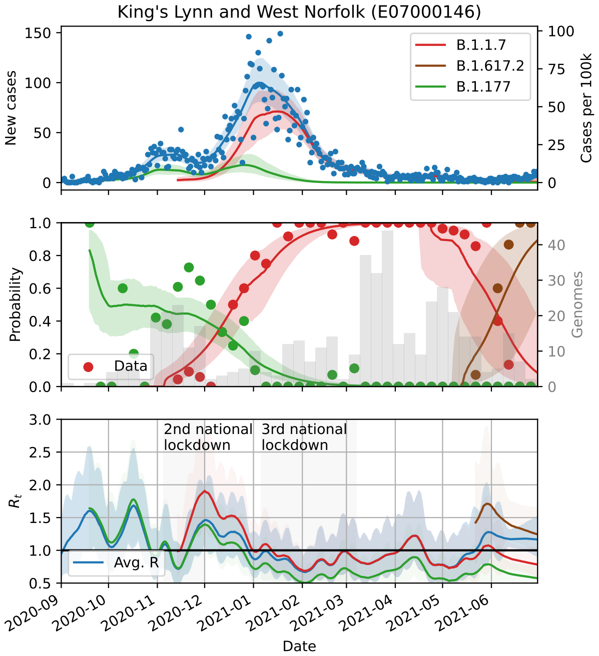
King's Lynn and West Norfolk (E07000146)
New cases
Cases per 100k
B.1.1.7
B.1.617.2
B.1.177
Probability
Genomes
Data
R_t
2nd national lockdown
3rd national lockdown
Avg. R
Date

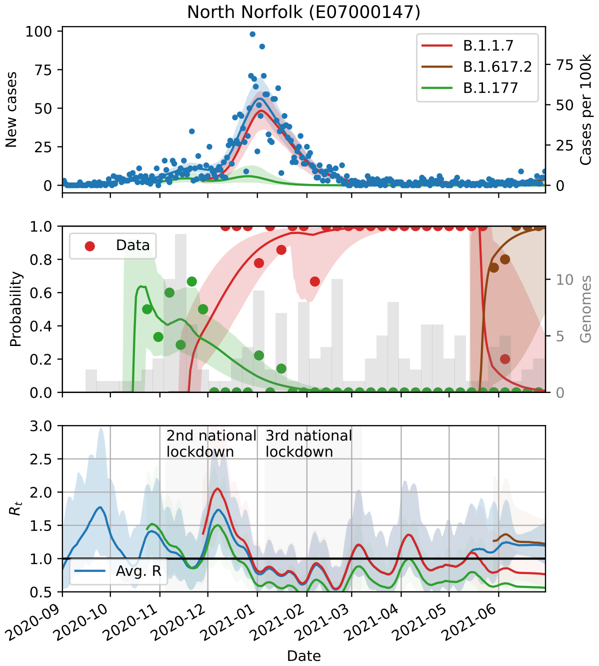
North Norfolk (E07000147)
New cases
Cases per 100k
B.1.1.7
B.1.617.2
B.1.177
Probability
Genomes
Data
R_t
2nd national lockdown
3rd national lockdown
Avg. R
Date

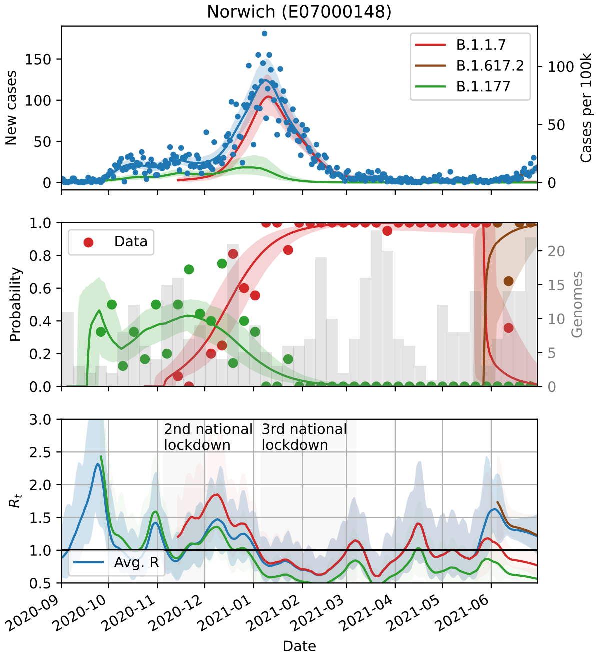
Norwich (E07000148)
New cases
Cases per 100k
B.1.1.7
B.1.617.2
B.1.177
Probability
Genomes
Data
R_t
2nd national lockdown
3rd national lockdown
Avg. R
Date

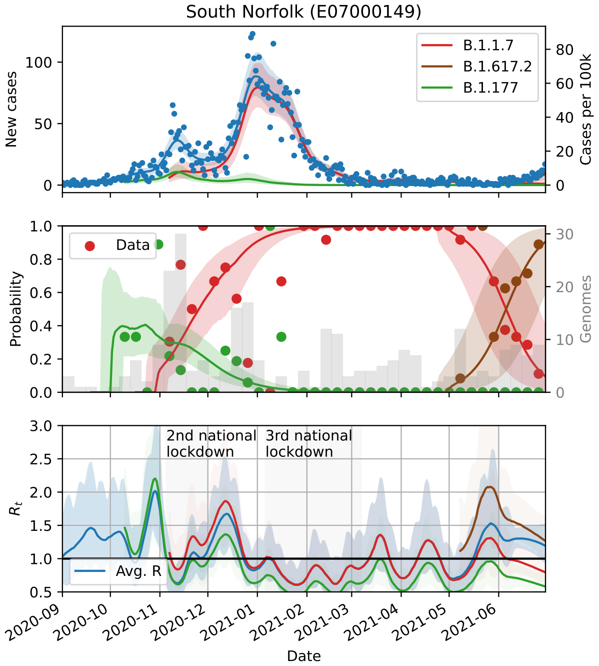
South Norfolk (E07000149)
New cases
Cases per 100k
B.1.1.7
B.1.617.2
B.1.177
Probability
Genomes
Data
R_t
2nd national lockdown
3rd national lockdown
Avg. R
Date

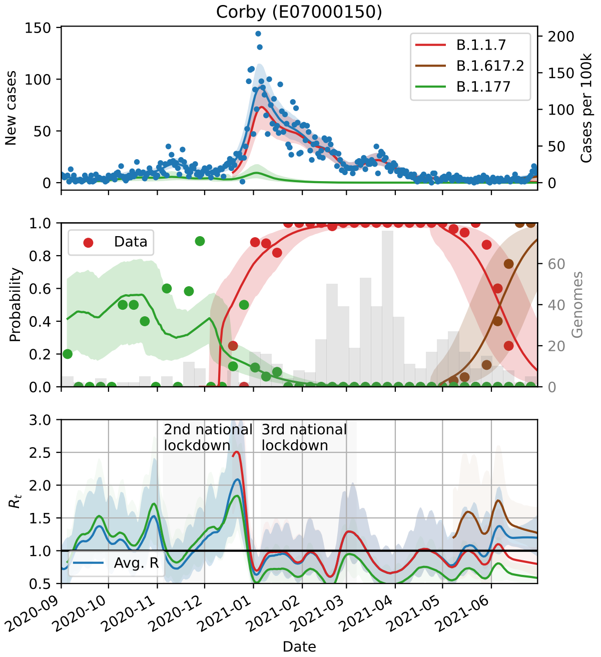
Corby (E07000150)
B.1.1.7
B.1.617.2
B.1.177
New cases
Cases per 100k
Data
Probability
Genomes
2nd national lockdown
3rd national lockdown
Avg. R
R_t
2020-09
2020-10
2020-11
2020-12
2021-01
2021-02
2021-03
2021-04
2021-05
2021-06
Date

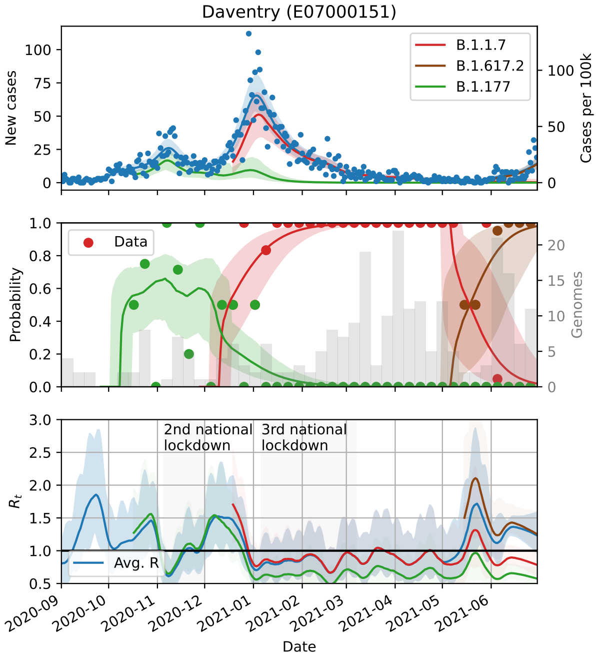
Daventry (E07000151)
B.1.1.7
B.1.617.2
B.1.177
New cases
Cases per 100k
Data
Probability
Genomes
2nd national lockdown
3rd national lockdown
Avg. R
R_t
2020-09
2020-10
2020-11
2020-12
2021-01
2021-02
2021-03
2021-04
2021-05
2021-06
Date

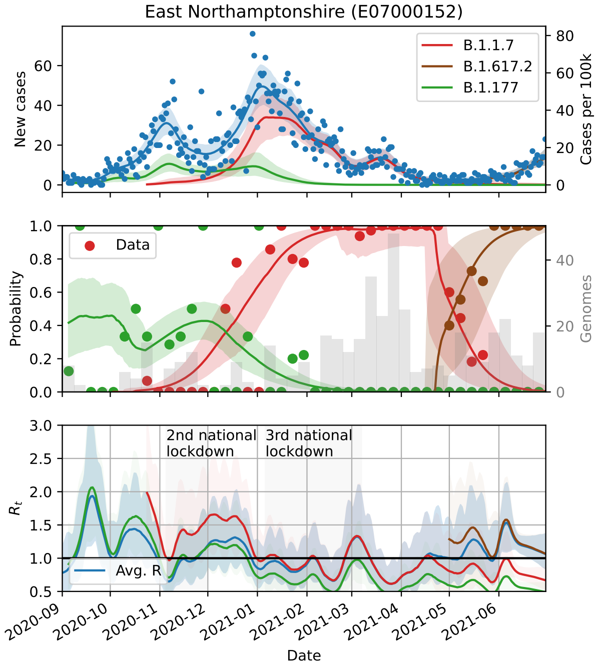
East Northamptonshire (E07000152)
B.1.1.7
B.1.617.2
B.1.177
New cases
Cases per 100k
Data
Probability
Genomes
2nd national lockdown
3rd national lockdown
Avg. R
R_t
2020-09
2020-10
2020-11
2020-12
2021-01
2021-02
2021-03
2021-04
2021-05
2021-06
Date

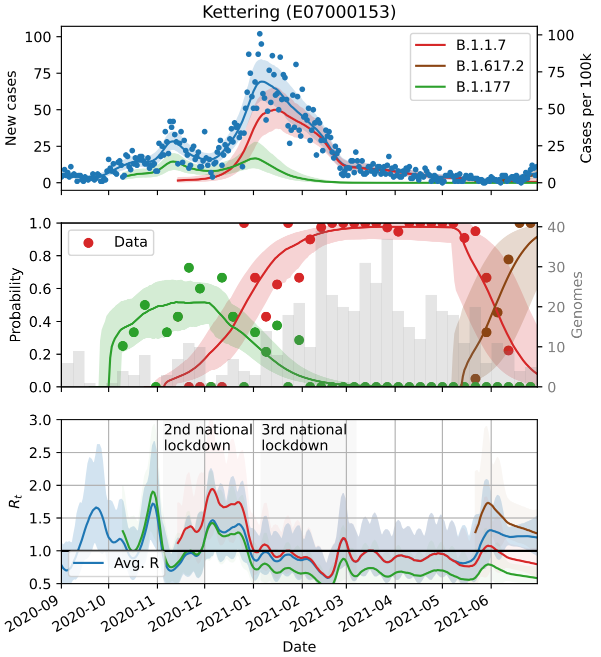
Kettering (E07000153)
B.1.1.7
B.1.617.2
B.1.177
New cases
Cases per 100k
Data
Probability
Genomes
2nd national lockdown
3rd national lockdown
Avg. R
R_t
2020-09
2020-10
2020-11
2020-12
2021-01
2021-02
2021-03
2021-04
2021-05
2021-06
Date

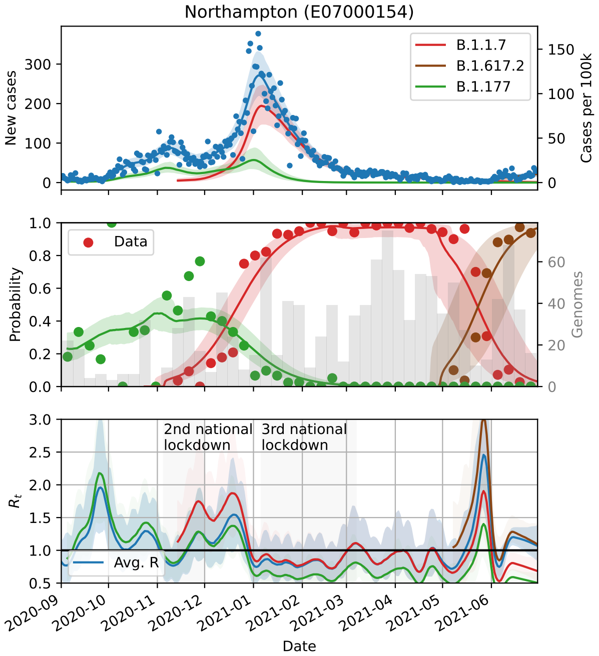
Northampton (E07000154)
B.1.1.7
B.1.617.2
B.1.177
New cases
Cases per 100k
Data
Probability
Genomes
2nd national lockdown
3rd national lockdown
Avg. R
R_t
Date

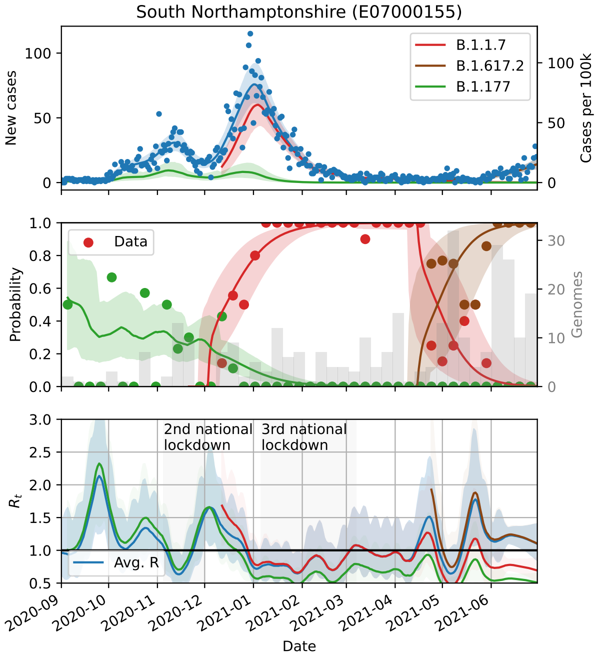
South Northamptonshire (E07000155)
B.1.1.7
B.1.617.2
B.1.177
New cases
Cases per 100k
Data
Probability
Genomes
2nd national lockdown
3rd national lockdown
Avg. R
R_t
Date

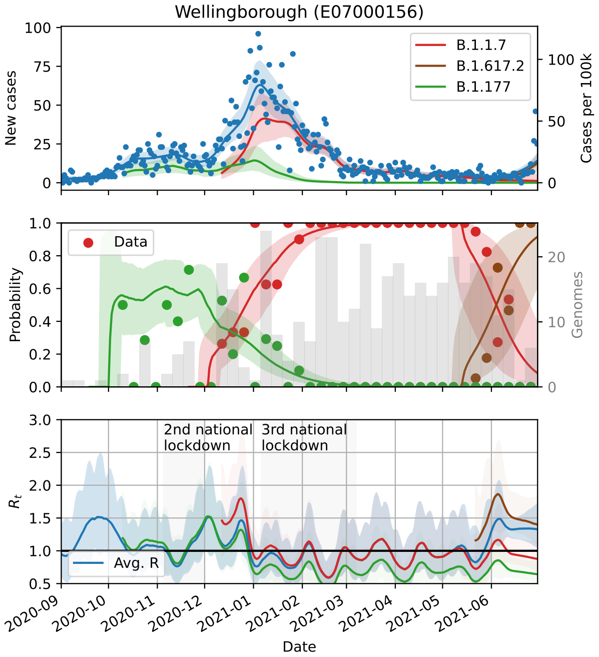
Wellingborough (E07000156)
B.1.1.7
B.1.617.2
B.1.177
New cases
Cases per 100k
Data
Probability
Genomes
2nd national lockdown
3rd national lockdown
Avg. R
R_t
Date

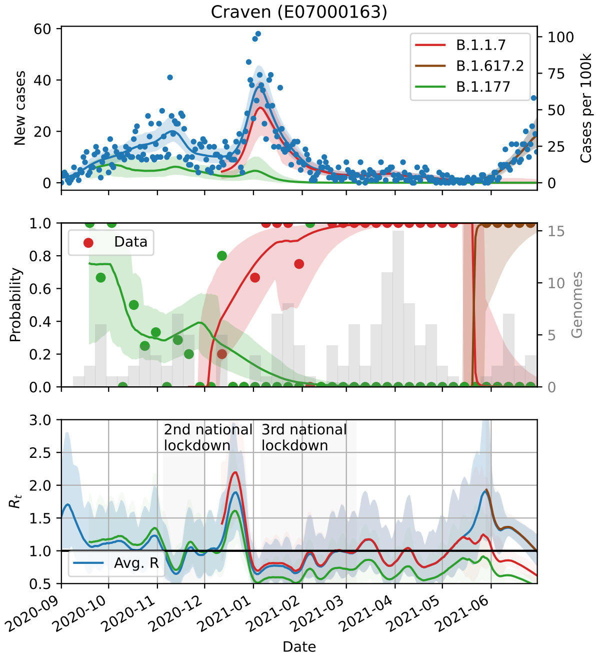
Craven (E07000163)
B.1.1.7
B.1.617.2
B.1.177
New cases
Cases per 100k
Data
Probability
Genomes
2nd national lockdown
3rd national lockdown
Avg. R
R_t
Date

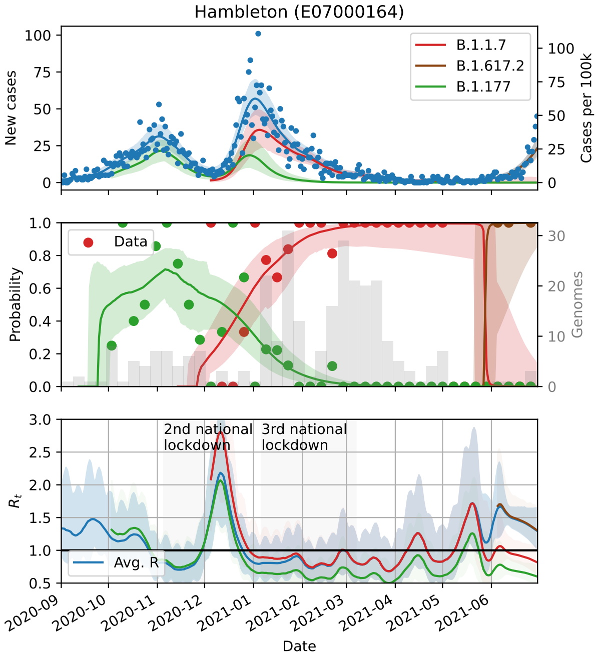

Hambleton (E07000164)
B.1.1.7
B.1.617.2
B.1.177
New cases
Cases per 100k
Data
Probability
Genomes
2nd national lockdown
3rd national lockdown
Avg. R
R_t
Date

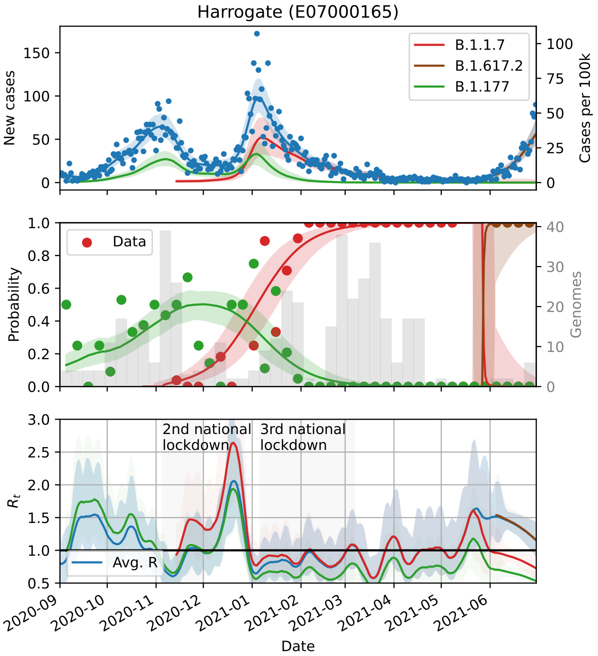

Harrogate (E07000165)
B.1.1.7
B.1.617.2
B.1.177
New cases
Cases per 100k
Data
Probability
Genomes
2nd national lockdown
3rd national lockdown
Avg. R
R_t
Date

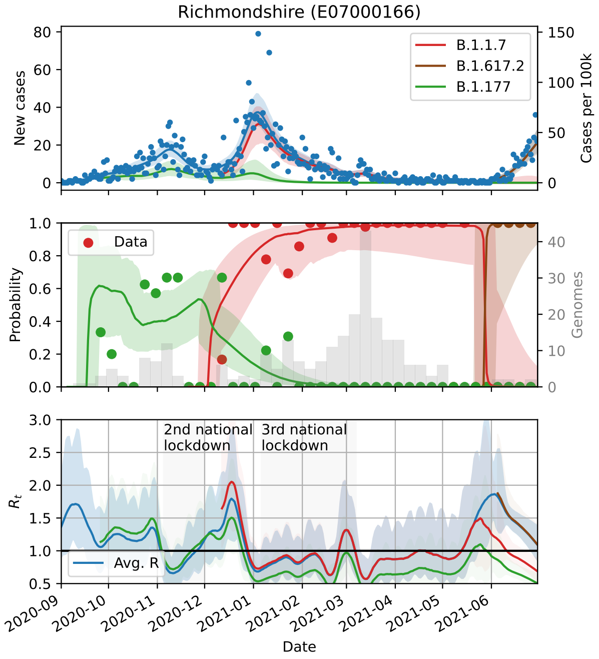

Richmondshire (E07000166)
B.1.1.7
B.1.617.2
B.1.177
New cases
Cases per 100k
Data
Probability
Genomes
2nd national lockdown
3rd national lockdown
Avg. R
R_t
Date

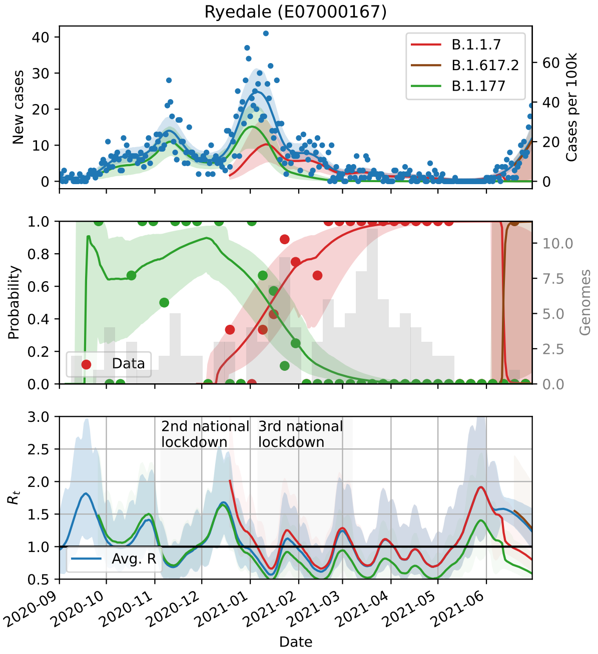

Ryedale (E07000167)
B.1.1.7
B.1.617.2
B.1.177
New cases
Cases per 100k
Data
Probability
Genomes
2nd national lockdown
3rd national lockdown
Avg. R
R_t
Date

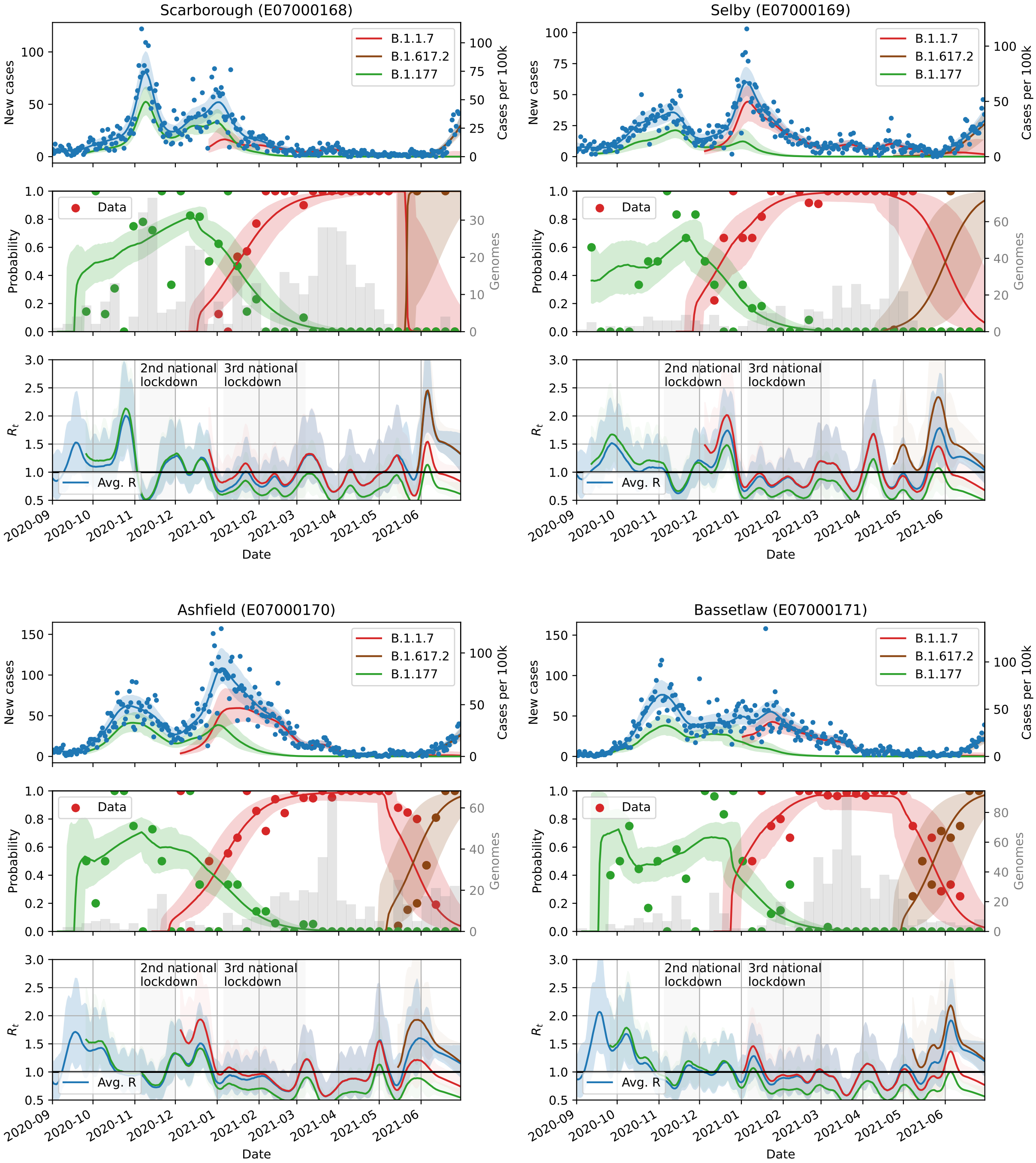
Scarborough (E07000168)
Selby (E07000169)
Ashfield (E07000170)
Bassetlaw (E07000171)
B.1.1.7
B.1.617.2
B.1.177
New cases
Cases per 100k
Data
Probability
Genomes
2nd national lockdown
3rd national lockdown
Avg. R
R_t
Date

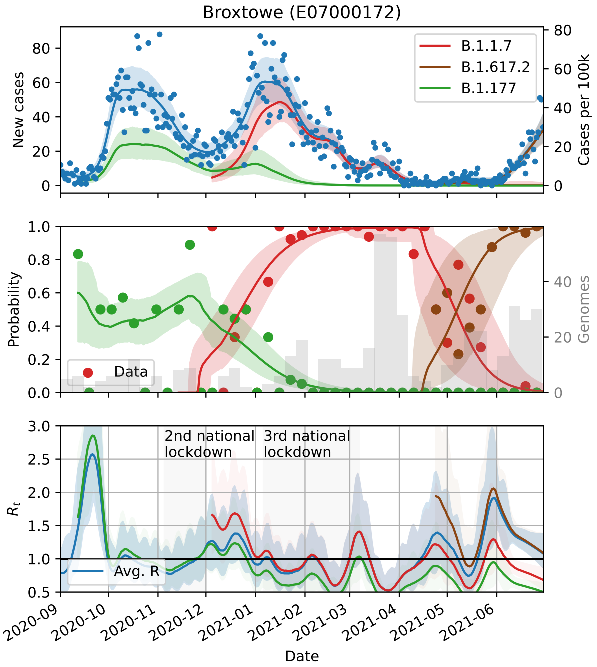
Broxtowe (E07000172)
New cases
Cases per 100k
B.1.1.7
B.1.617.2
B.1.177
Probability
Data
Genomes
R_t
2nd national lockdown
3rd national lockdown
Avg. R
Date

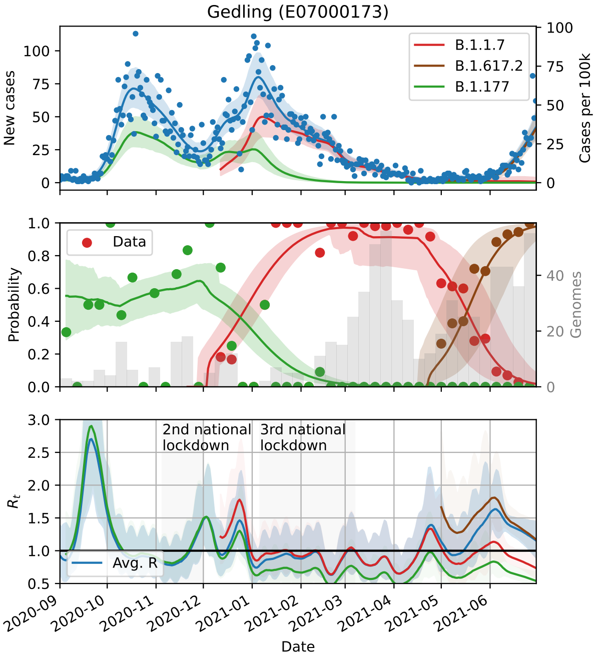
Gedling (E07000173)
New cases
Cases per 100k
B.1.1.7
B.1.617.2
B.1.177
Probability
Data
Genomes
R_t
2nd national lockdown
3rd national lockdown
Avg. R
Date

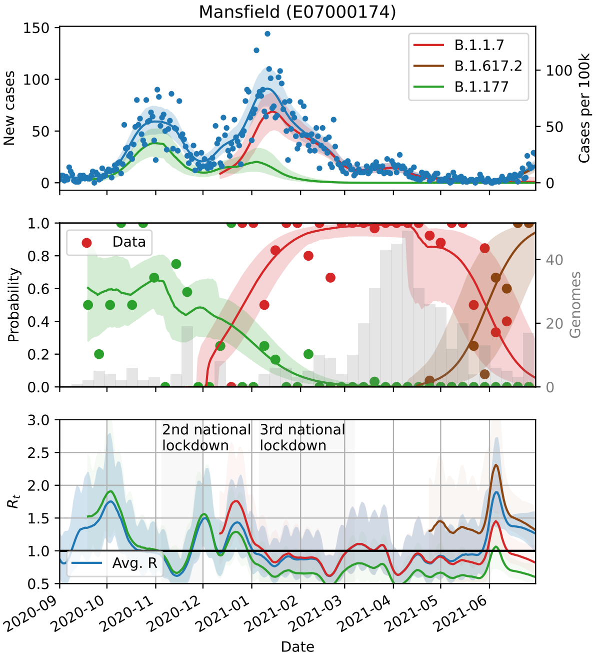
Mansfield (E07000174)
New cases
Cases per 100k
B.1.1.7
B.1.617.2
B.1.177
Probability
Data
Genomes
R_t
2nd national lockdown
3rd national lockdown
Avg. R
Date

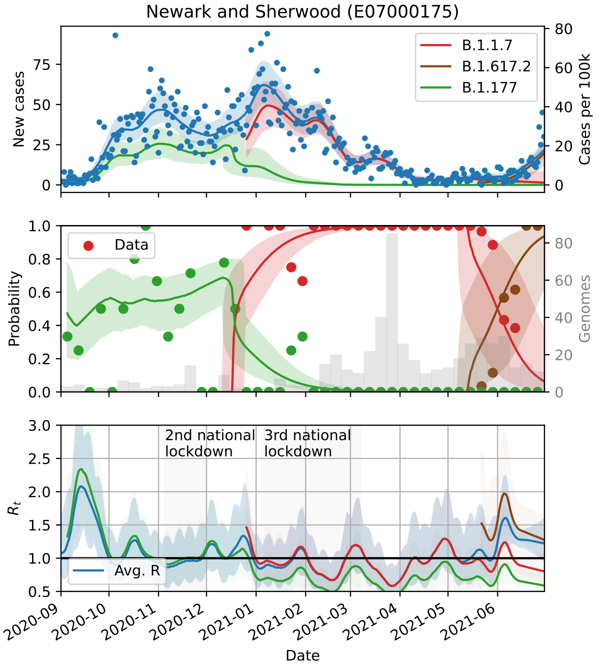
Newark and Sherwood (E07000175)
New cases
Cases per 100k
B.1.1.7
B.1.617.2
B.1.177
Probability
Data
Genomes
R_t
2nd national lockdown
3rd national lockdown
Avg. R
Date

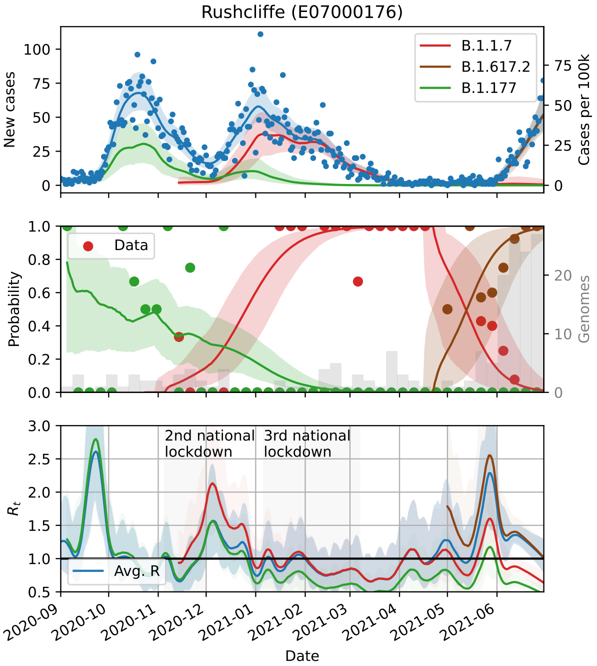
Rushcliffe (E07000176)
B.1.1.7
B.1.617.2
B.1.177
New cases
Cases per 100k
Data
Probability
Genomes
2nd national lockdown
3rd national lockdown
Avg. R
R_t
Date

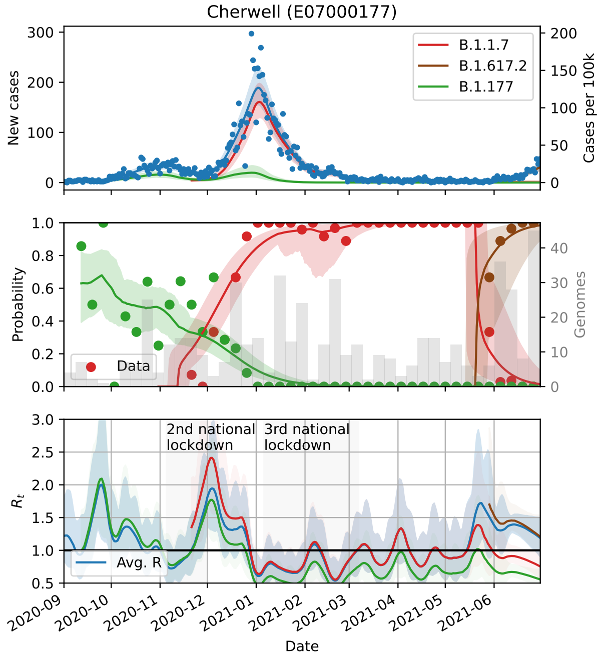
Cherwell (E07000177)
B.1.1.7
B.1.617.2
B.1.177
New cases
Cases per 100k
Data
Probability
Genomes
2nd national lockdown
3rd national lockdown
Avg. R
R_t
Date

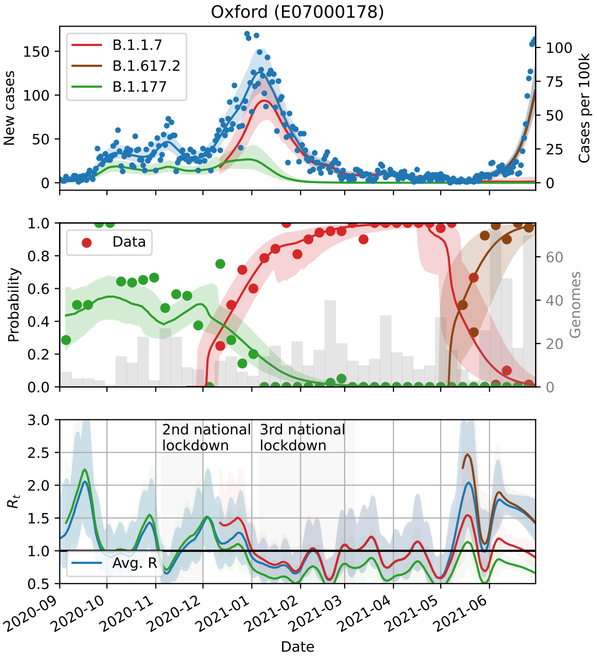
Oxford (E07000178)
B.1.1.7
B.1.617.2
B.1.177
New cases
Cases per 100k
Data
Probability
Genomes
2nd national lockdown
3rd national lockdown
Avg. R
R_t
Date

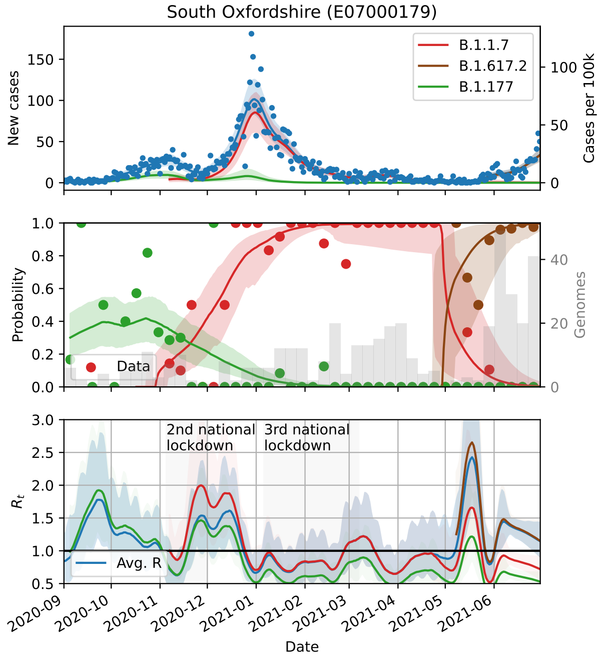
South Oxfordshire (E07000179)
B.1.1.7
B.1.617.2
B.1.177
New cases
Cases per 100k
Data
Probability
Genomes
2nd national lockdown
3rd national lockdown
Avg. R
R_t
Date

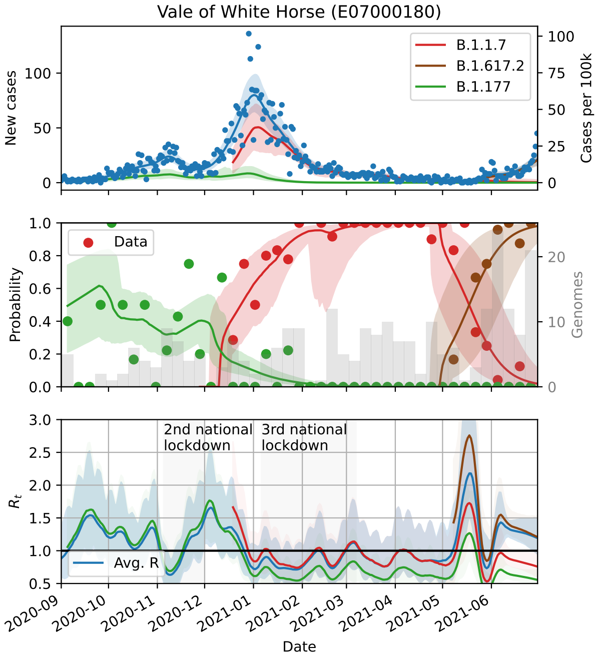
Vale of White Horse (E07000180)
B.1.1.7
B.1.617.2
B.1.177
New cases
Cases per 100k
Data
Probability
Genomes
2nd national lockdown
3rd national lockdown
Avg. R
R_t
Date

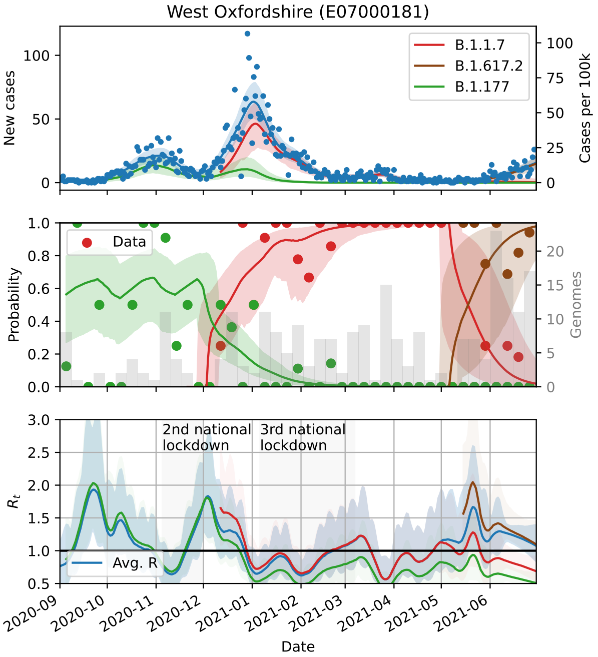
West Oxfordshire (E07000181)
B.1.1.7
B.1.617.2
B.1.177
New cases
Cases per 100k
Data
Probability
Genomes
2nd national lockdown
3rd national lockdown
Avg. R
R_t
Date

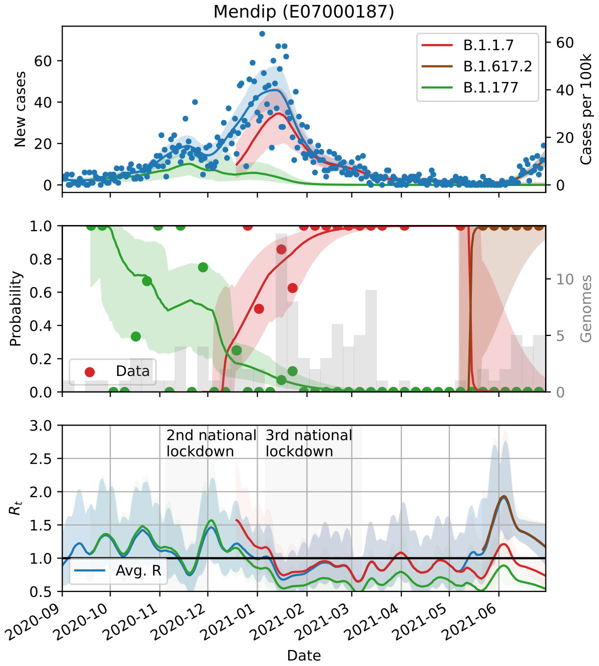
Mendip (E07000187)
B.1.1.7
B.1.617.2
B.1.177
New cases
Cases per 100k
Data
Probability
Genomes
2nd national lockdown
3rd national lockdown
Avg. R
R_t
Date

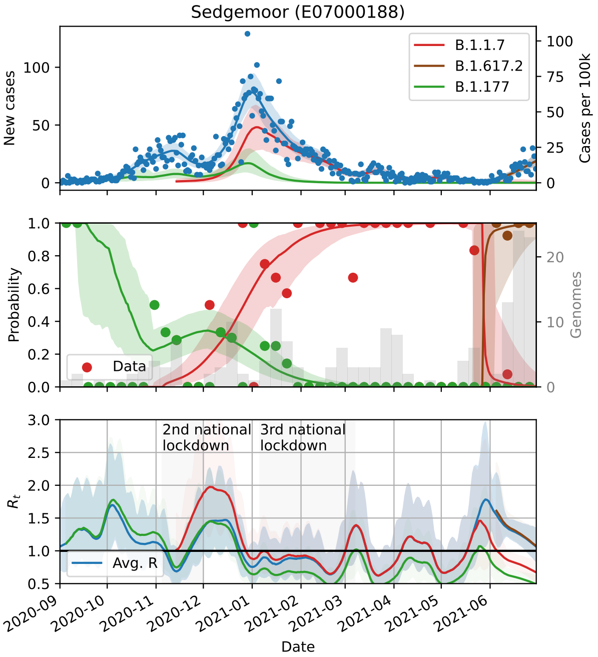
Sedgemoor (E07000188)
B.1.1.7
B.1.617.2
B.1.177
New cases
Cases per 100k
Data
Probability
Genomes
2nd national lockdown
3rd national lockdown
Avg. R
R_t
Date

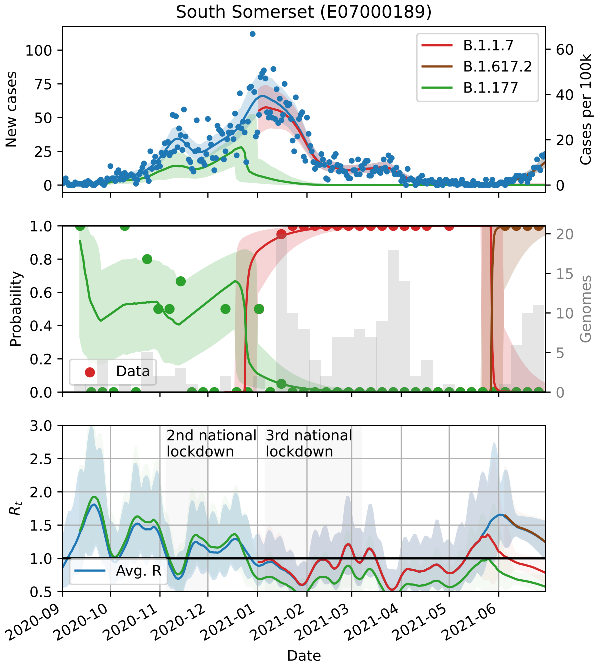
South Somerset (E07000189)
B.1.1.7
B.1.617.2
B.1.177
New cases
Cases per 100k
Probability
Genomes
Data
R_t
2nd national lockdown
3rd national lockdown
Avg. R
Date

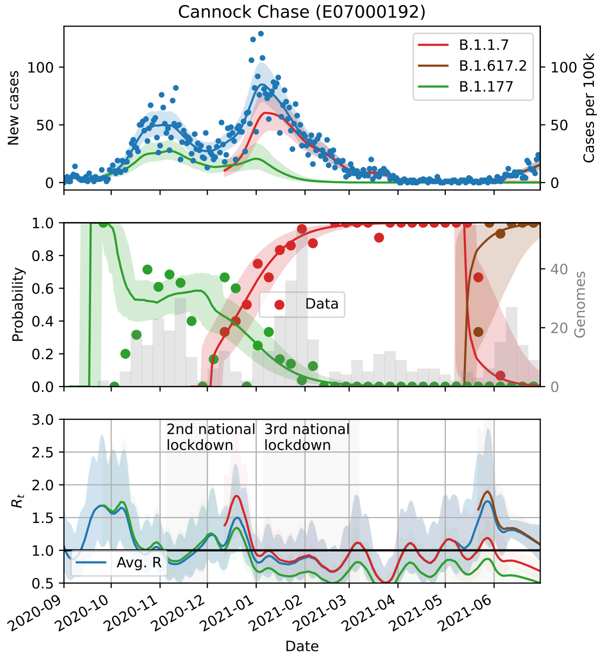
Cannock Chase (E07000192)
B.1.1.7
B.1.617.2
B.1.177
New cases
Cases per 100k
Probability
Genomes
Data
R_t
2nd national lockdown
3rd national lockdown
Avg. R
Date

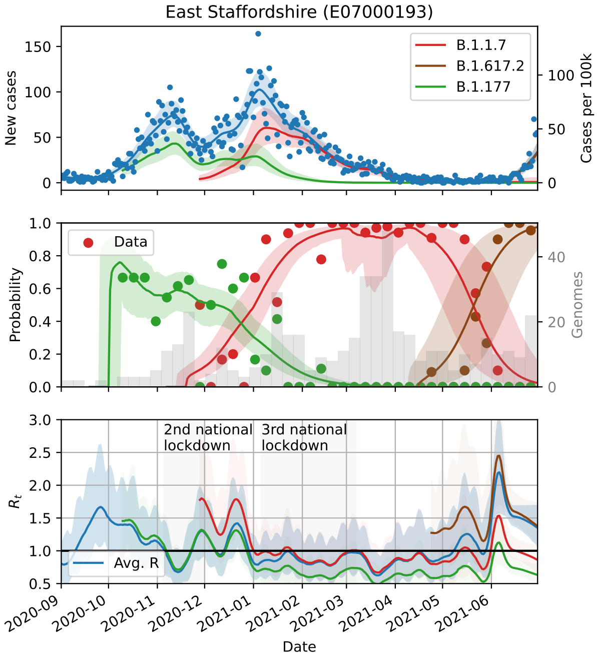
East Staffordshire (E07000193)
B.1.1.7
B.1.617.2
B.1.177
New cases
Cases per 100k
Probability
Genomes
Data
R_t
2nd national lockdown
3rd national lockdown
Avg. R
Date

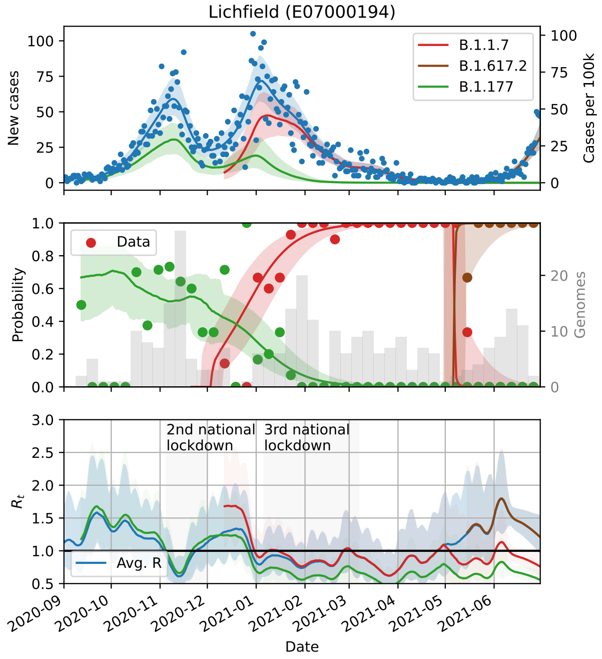
Lichfield (E07000194)
B.1.1.7
B.1.617.2
B.1.177
New cases
Cases per 100k
Probability
Genomes
Data
R_t
2nd national lockdown
3rd national lockdown
Avg. R
Date

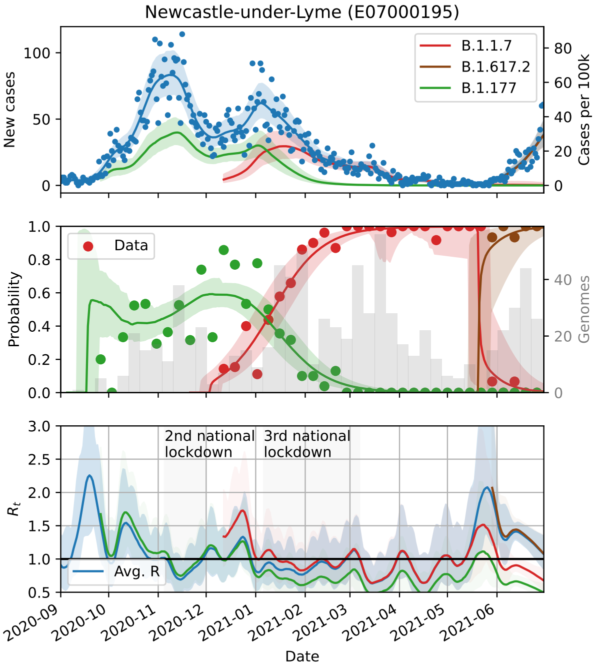
Newcastle-under-Lyme (E07000195)
New cases
Cases per 100k
B.1.1.7
B.1.617.2
B.1.177
Data
Probability
Genomes
2nd national lockdown
3rd national lockdown
R_t
Avg. R
2020-09
2020-10
2020-11
2020-12
2021-01
2021-02
2021-03
2021-04
2021-05
2021-06
Date

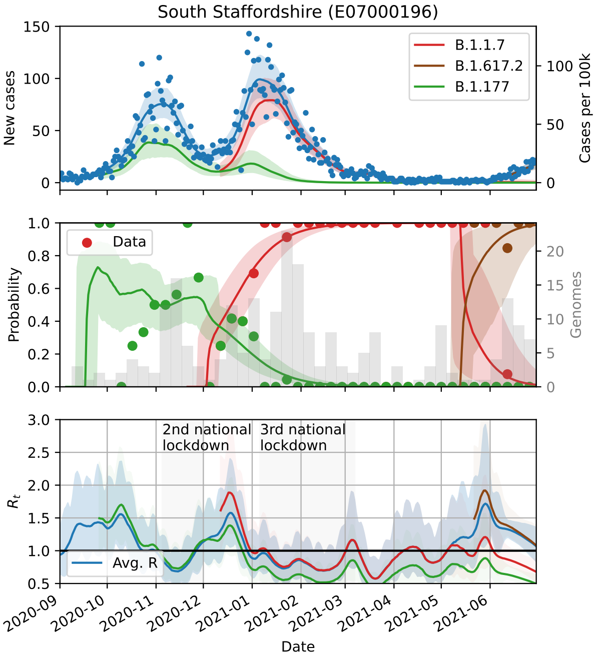
South Staffordshire (E07000196)
New cases
Cases per 100k
B.1.1.7
B.1.617.2
B.1.177
Data
Probability
Genomes
2nd national lockdown
3rd national lockdown
R_t
Avg. R
2020-09
2020-10
2020-11
2020-12
2021-01
2021-02
2021-03
2021-04
2021-05
2021-06
Date

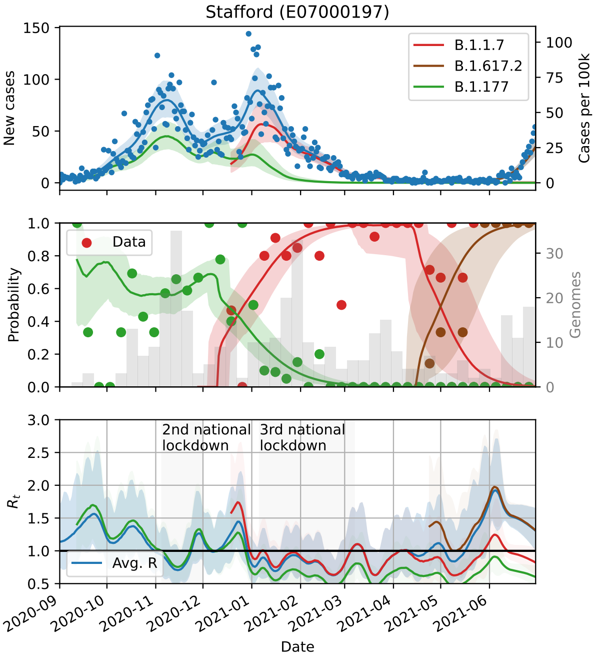
Stafford (E07000197)
New cases
Cases per 100k
B.1.1.7
B.1.617.2
B.1.177
Data
Probability
Genomes
2nd national lockdown
3rd national lockdown
R_t
Avg. R
2020-09
2020-10
2020-11
2020-12
2021-01
2021-02
2021-03
2021-04
2021-05
2021-06
Date

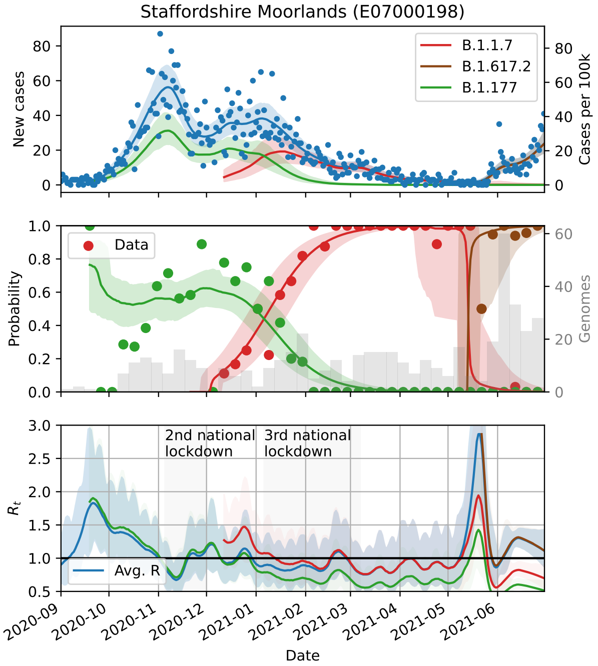
Staffordshire Moorlands (E07000198)
New cases
Cases per 100k
B.1.1.7
B.1.617.2
B.1.177
Data
Probability
Genomes
2nd national lockdown
3rd national lockdown
R_t
Avg. R
2020-09
2020-10
2020-11
2020-12
2021-01
2021-02
2021-03
2021-04
2021-05
2021-06
Date

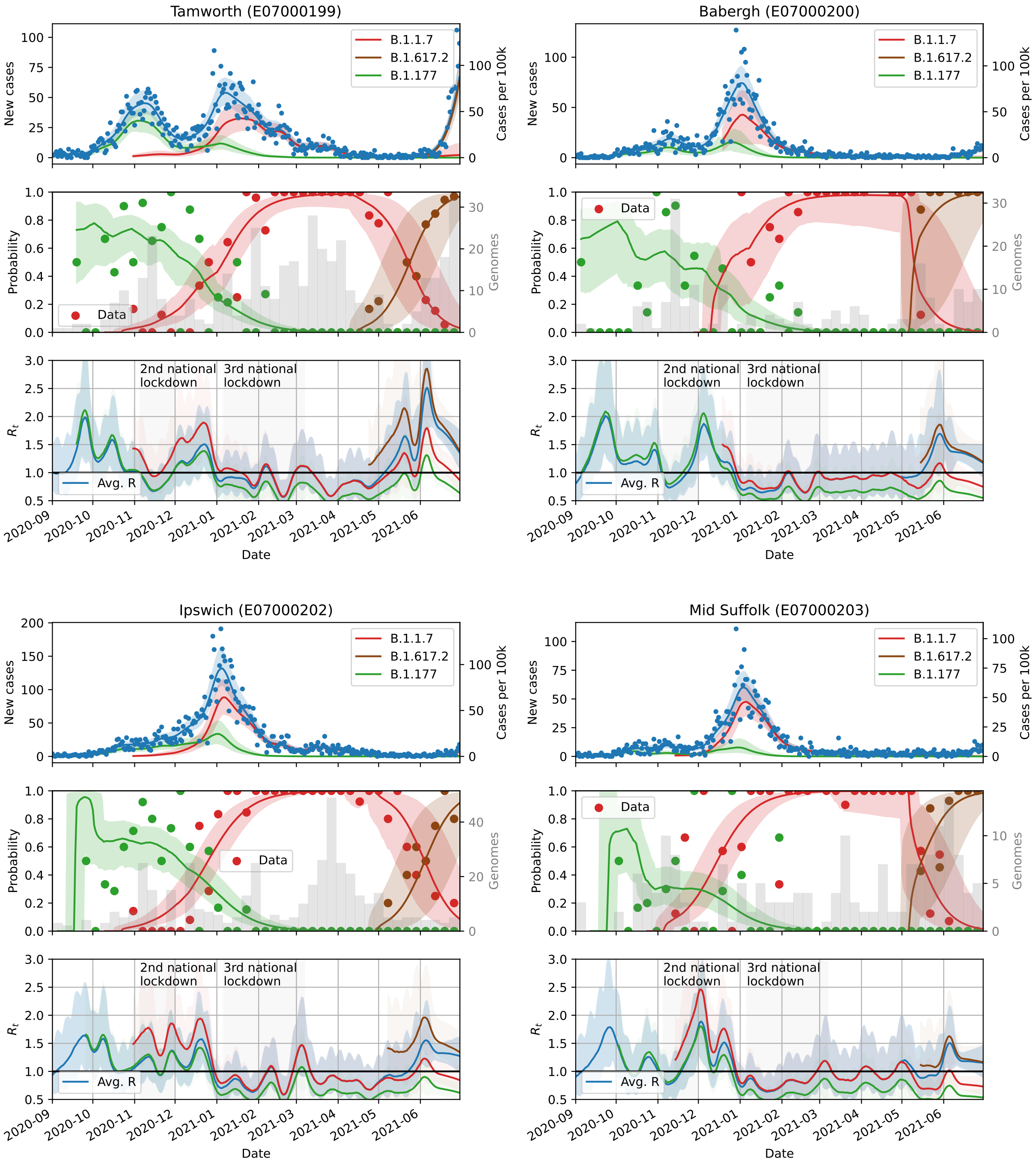

Tamworth (E07000199)
Babergh (E07000200)
Ipswich (E07000202)
Mid Suffolk (E07000203)
B.1.1.7
B.1.617.2
B.1.177
New cases
Cases per 100k
Probability
Genomes
Data
R_t
2nd national lockdown
3rd national lockdown
Avg. R
Date

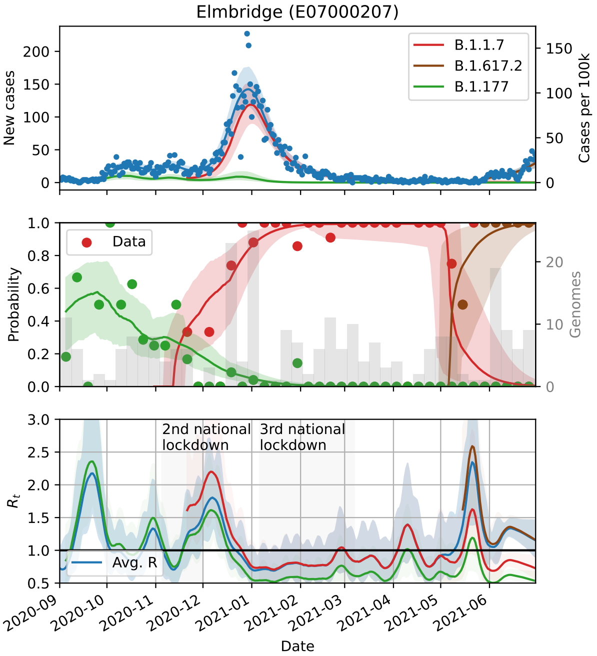
Elmbridge (E07000207)
New cases
Cases per 100k
B.1.1.7
B.1.617.2
B.1.177
Probability
Genomes
Data
R_t
2nd national lockdown
3rd national lockdown
Avg. R
Date

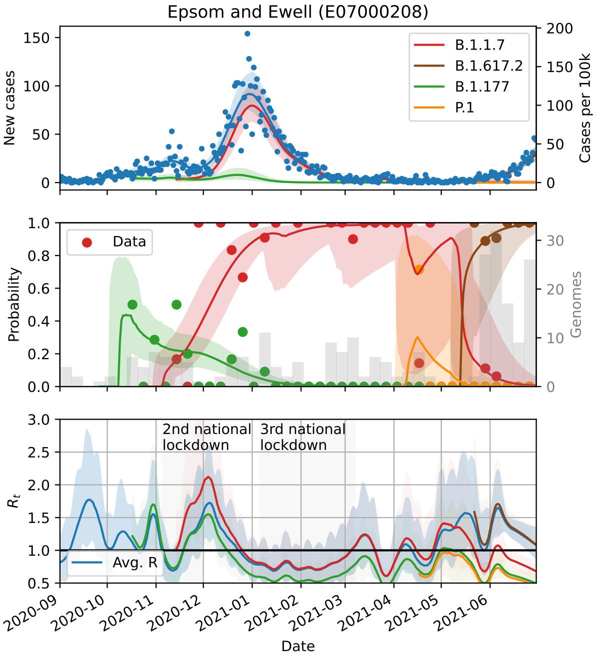
Epsom and Ewell (E07000208)
New cases
Cases per 100k
B.1.1.7
B.1.617.2
B.1.177
P.1
Probability
Genomes
Data
R_t
2nd national lockdown
3rd national lockdown
Avg. R
Date

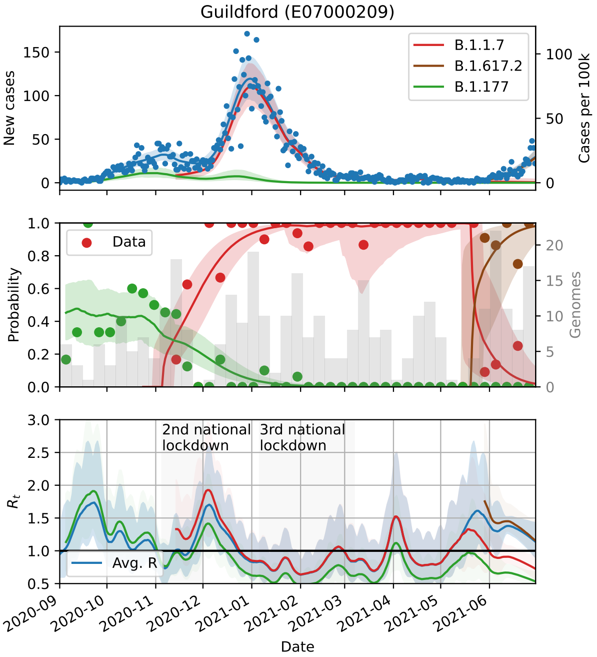
Guildford (E07000209)
New cases
Cases per 100k
B.1.1.7
B.1.617.2
B.1.177
Probability
Genomes
Data
R_t
2nd national lockdown
3rd national lockdown
Avg. R
Date

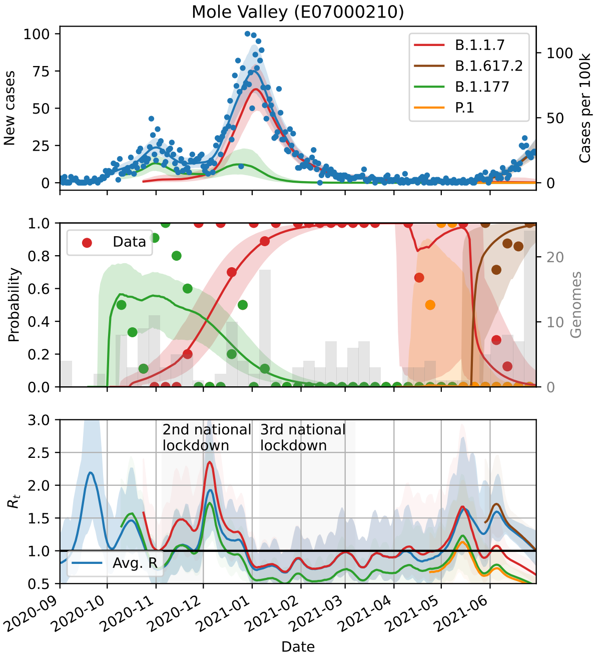
Mole Valley (E07000210)
New cases
Cases per 100k
B.1.1.7
B.1.617.2
B.1.177
P.1
Probability
Genomes
Data
R_t
2nd national lockdown
3rd national lockdown
Avg. R
Date

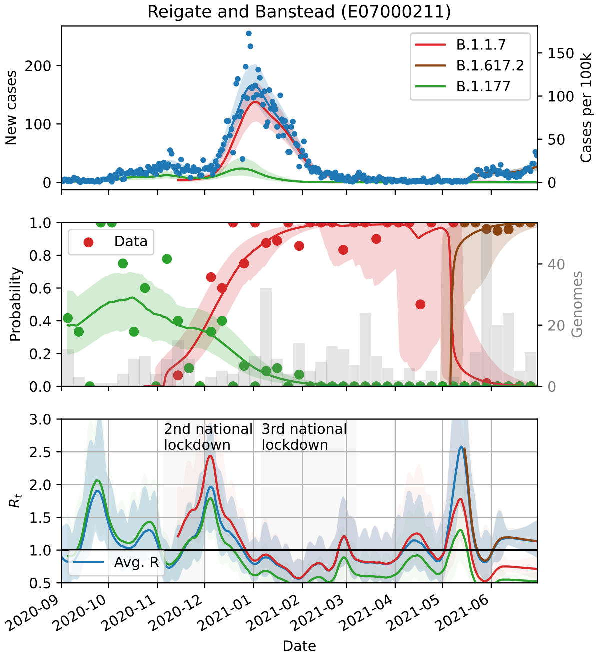
Reigate and Banstead (E07000211)
B.1.1.7
B.1.617.2
B.1.177
New cases
Cases per 100k
Data
Probability
Genomes
2nd national lockdown
3rd national lockdown
Avg. R
R_t
Date

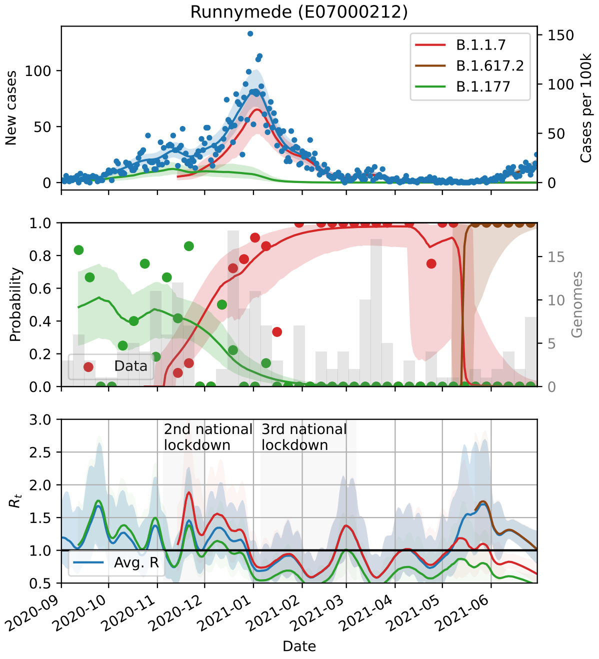
Runnymede (E07000212)
B.1.1.7
B.1.617.2
B.1.177
New cases
Cases per 100k
Data
Probability
Genomes
2nd national lockdown
3rd national lockdown
Avg. R
R_t
Date

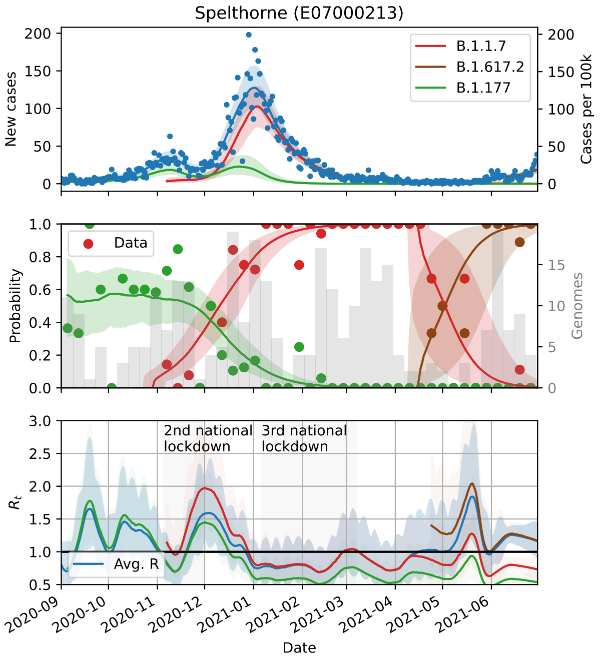
Spelthorne (E07000213)
B.1.1.7
B.1.617.2
B.1.177
New cases
Cases per 100k
Data
Probability
Genomes
2nd national lockdown
3rd national lockdown
Avg. R
R_t
Date

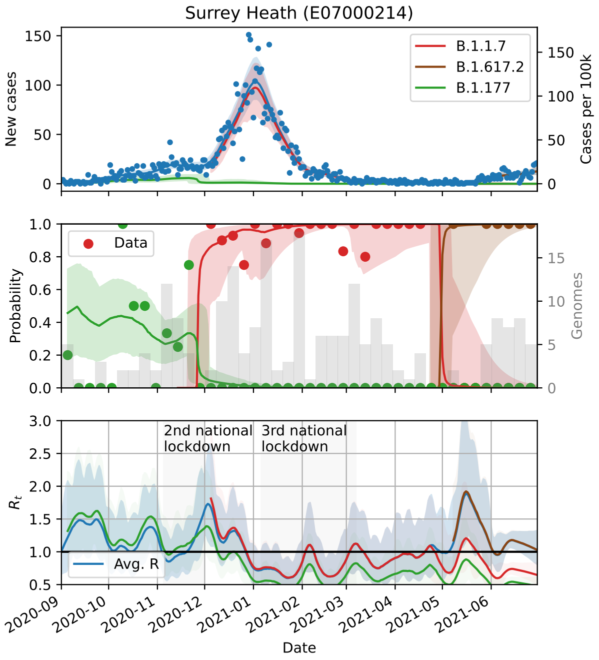
Surrey Heath (E07000214)
B.1.1.7
B.1.617.2
B.1.177
New cases
Cases per 100k
Data
Probability
Genomes
2nd national lockdown
3rd national lockdown
Avg. R
R_t
Date

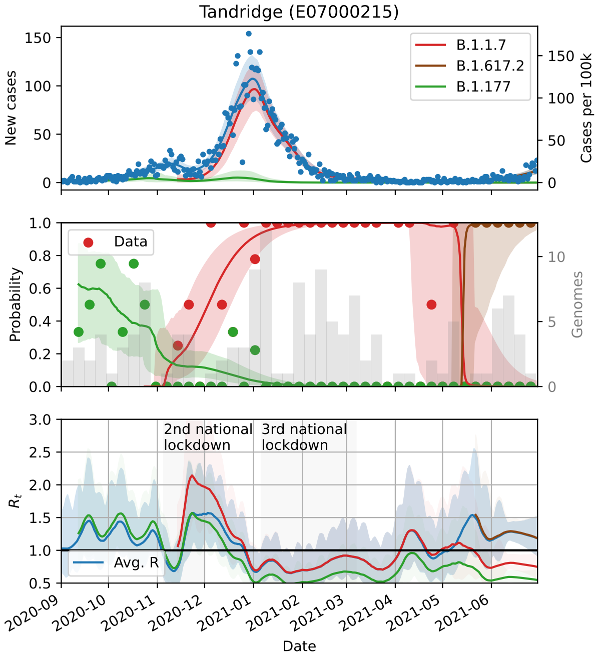
Tandridge (E07000215)
B.1.1.7
B.1.617.2
B.1.177
New cases
Cases per 100k
Data
Probability
Genomes
2nd national lockdown
3rd national lockdown
Avg. R
R_t
Date

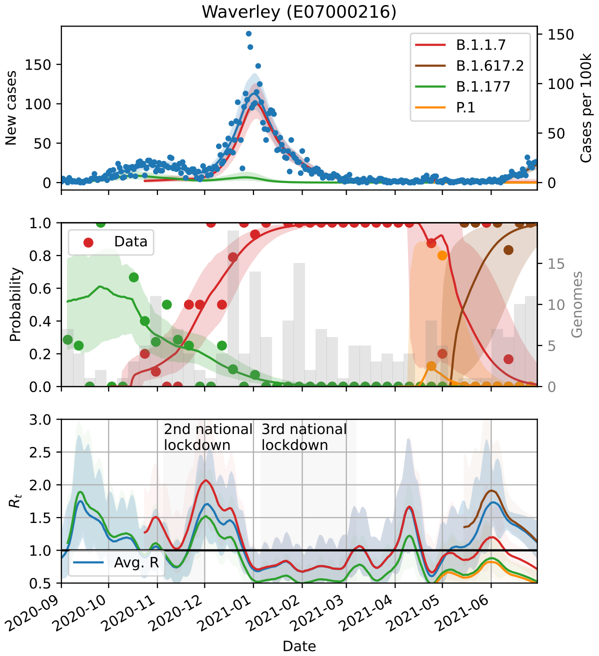
Waverley (E07000216)
B.1.1.7
B.1.617.2
B.1.177
P.1
New cases
Cases per 100k
Data
Probability
Genomes
2nd national lockdown
3rd national lockdown
Avg. R
R_t
Date

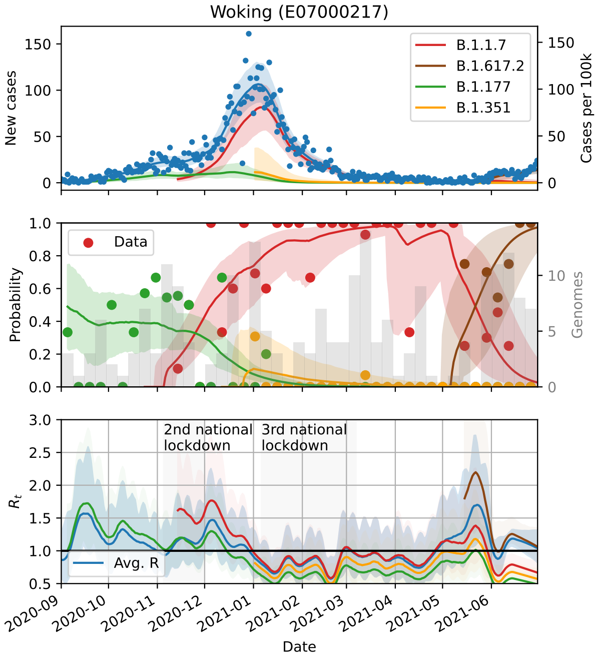
Woking (E07000217)
B.1.1.7
B.1.617.2
B.1.177
B.1.351
New cases
Cases per 100k
Data
Probability
Genomes
2nd national lockdown
3rd national lockdown
Avg. R
R_t
Date

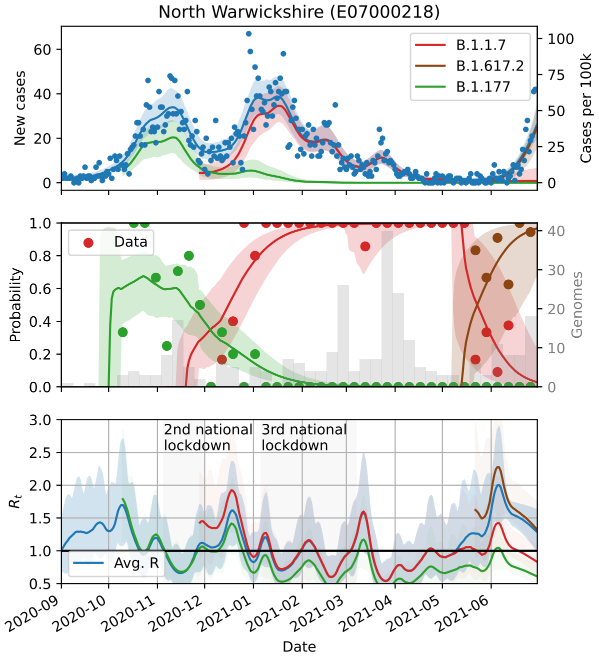
North Warwickshire (E07000218)
B.1.1.7
B.1.617.2
B.1.177
New cases
Cases per 100k
Data
Probability
Genomes
2nd national lockdown
3rd national lockdown
Avg. R
R_t
Date

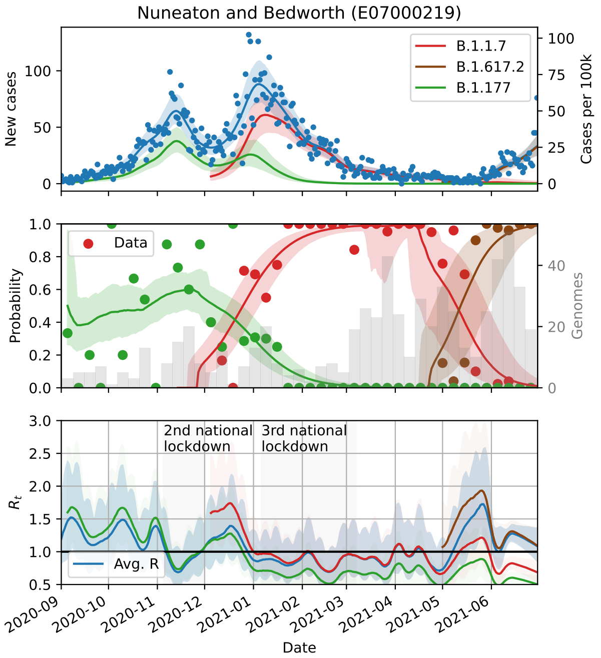
Nuneaton and Bedworth (E07000219)
New cases
Cases per 100k
B.1.1.7
B.1.617.2
B.1.177
Probability
Genomes
Data
R_t
2nd national lockdown
3rd national lockdown
Avg. R
Date

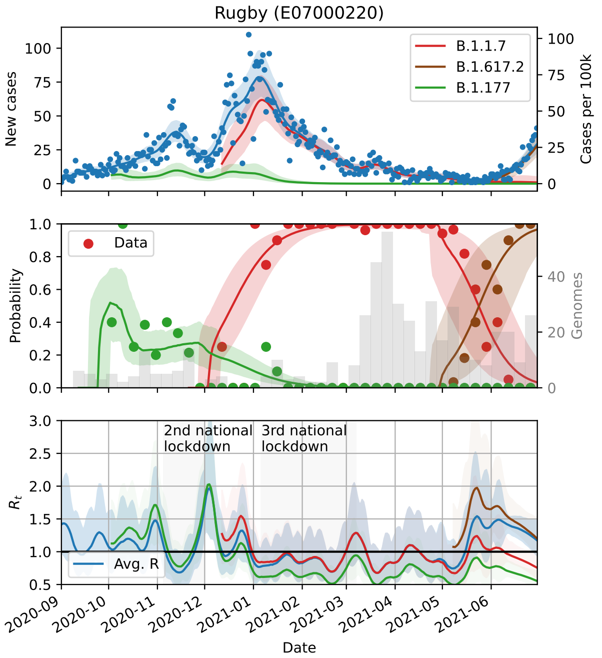
Rugby (E07000220)
New cases
Cases per 100k
B.1.1.7
B.1.617.2
B.1.177
Probability
Genomes
Data
R_t
2nd national lockdown
3rd national lockdown
Avg. R
Date

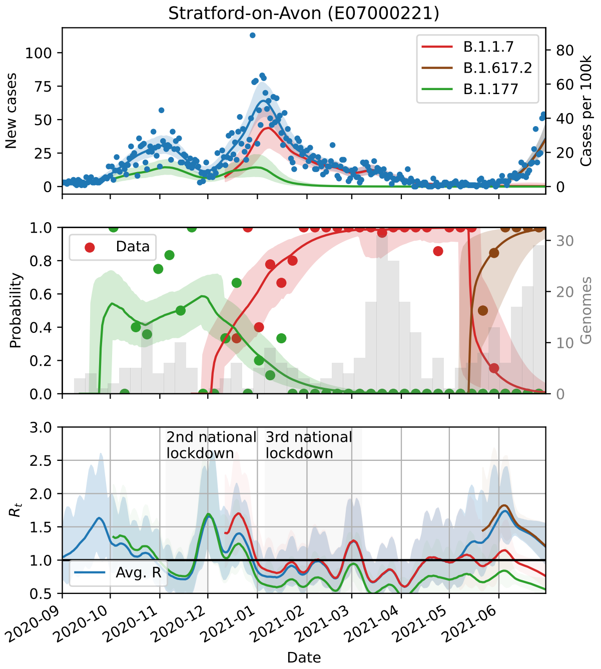
Stratford-on-Avon (E07000221)
New cases
Cases per 100k
B.1.1.7
B.1.617.2
B.1.177
Probability
Genomes
Data
R_t
2nd national lockdown
3rd national lockdown
Avg. R
Date

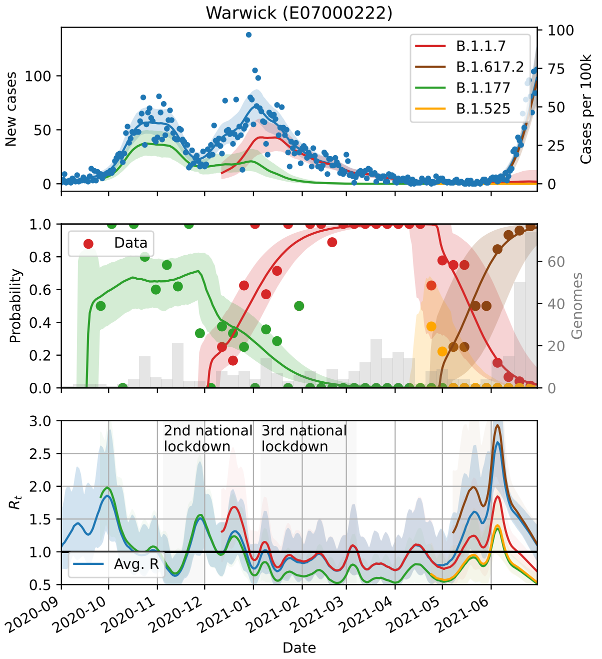
Warwick (E07000222)
New cases
Cases per 100k
B.1.1.7
B.1.617.2
B.1.177
B.1.525
Probability
Genomes
Data
R_t
2nd national lockdown
3rd national lockdown
Avg. R
Date

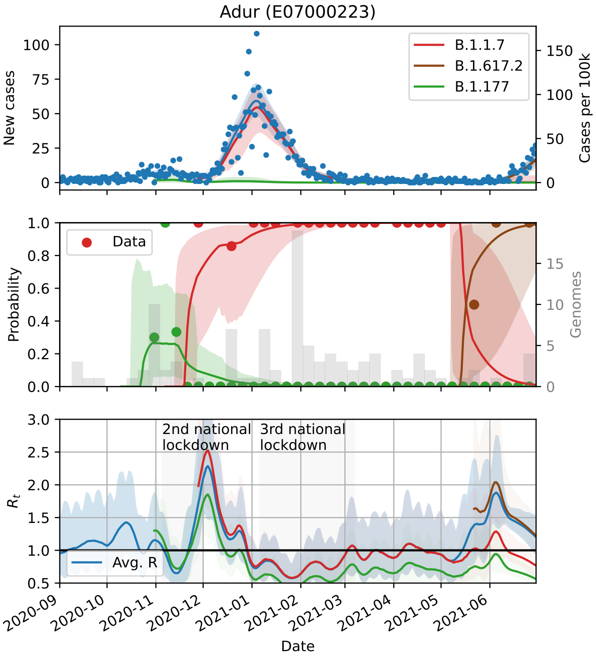
Adur (E07000223)
New cases
Cases per 100k
B.1.1.7
B.1.617.2
B.1.177
Data
Probability
Genomes
2nd national lockdown
3rd national lockdown
Avg. R
R_t
Date

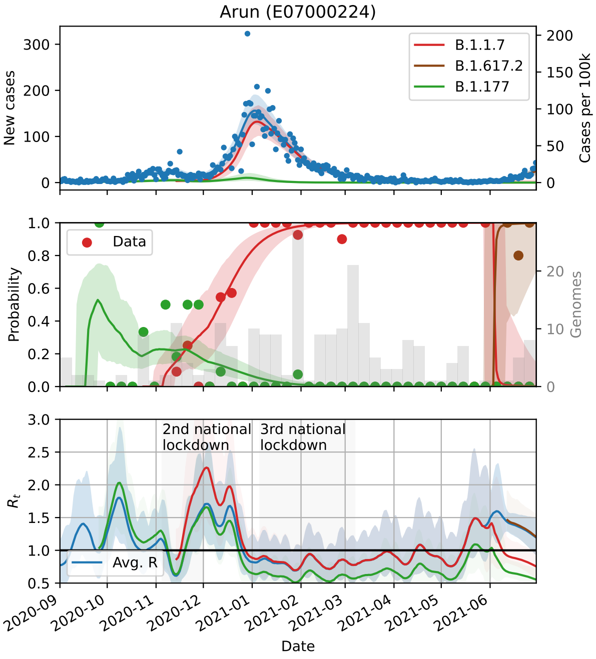
Arun (E07000224)
New cases
Cases per 100k
B.1.1.7
B.1.617.2
B.1.177
Data
Probability
Genomes
2nd national lockdown
3rd national lockdown
Avg. R
R_t
Date

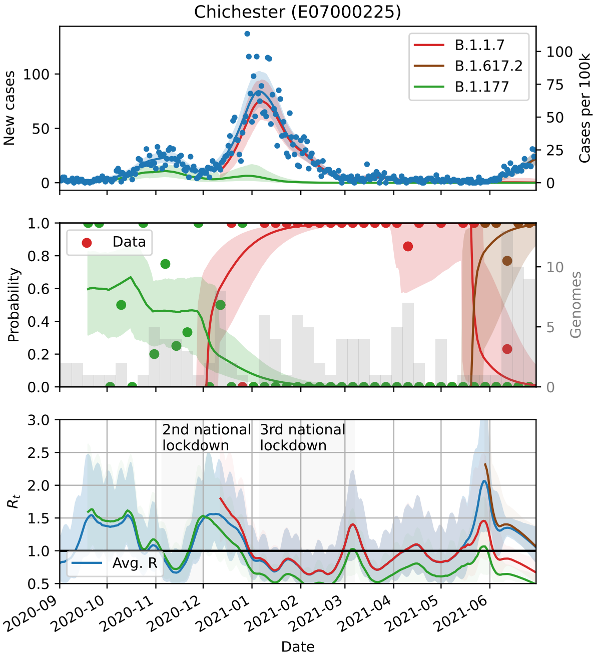
Chichester (E07000225)
New cases
Cases per 100k
B.1.1.7
B.1.617.2
B.1.177
Data
Probability
Genomes
2nd national lockdown
3rd national lockdown
Avg. R
R_t
Date

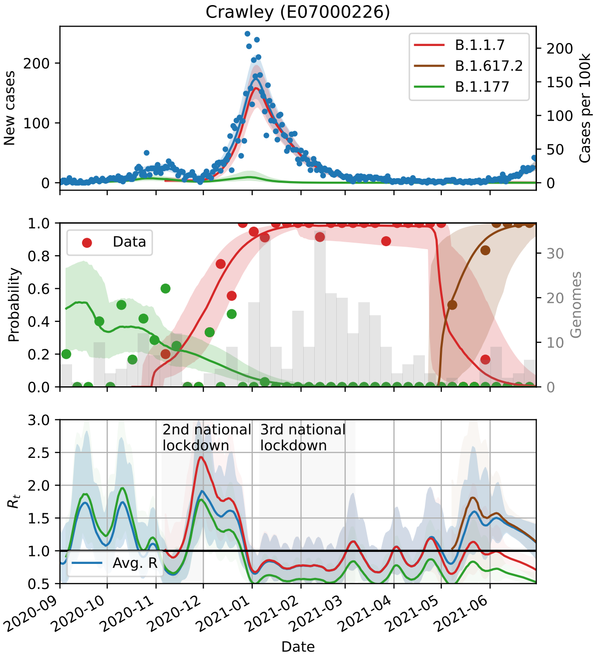
Crawley (E07000226)
New cases
Cases per 100k
B.1.1.7
B.1.617.2
B.1.177
Data
Probability
Genomes
2nd national lockdown
3rd national lockdown
Avg. R
R_t
Date

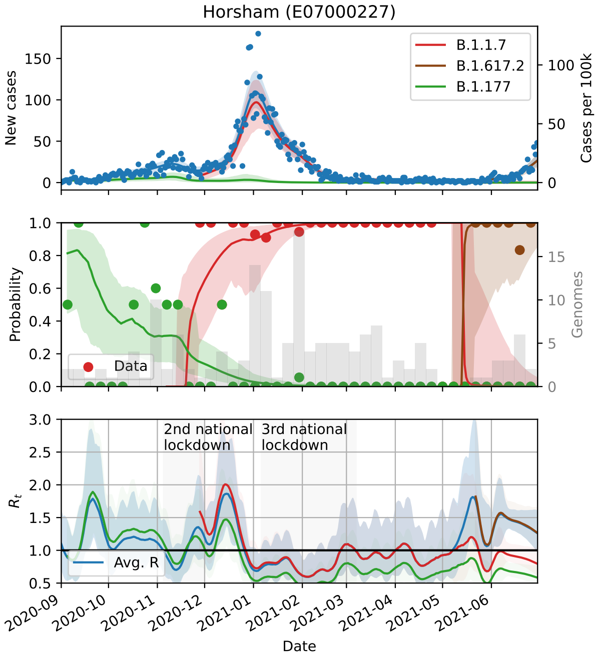
Horsham (E07000227)
New cases
Cases per 100k
B.1.1.7
B.1.617.2
B.1.177
Probability
Genomes
Data
R_t
2nd national lockdown
3rd national lockdown
Avg. R
Date

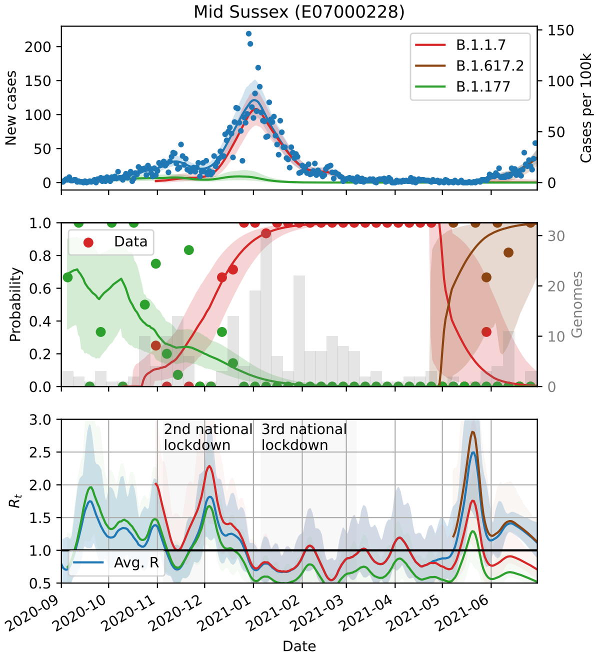
Mid Sussex (E07000228)
New cases
Cases per 100k
B.1.1.7
B.1.617.2
B.1.177
Probability
Genomes
Data
R_t
2nd national lockdown
3rd national lockdown
Avg. R
Date

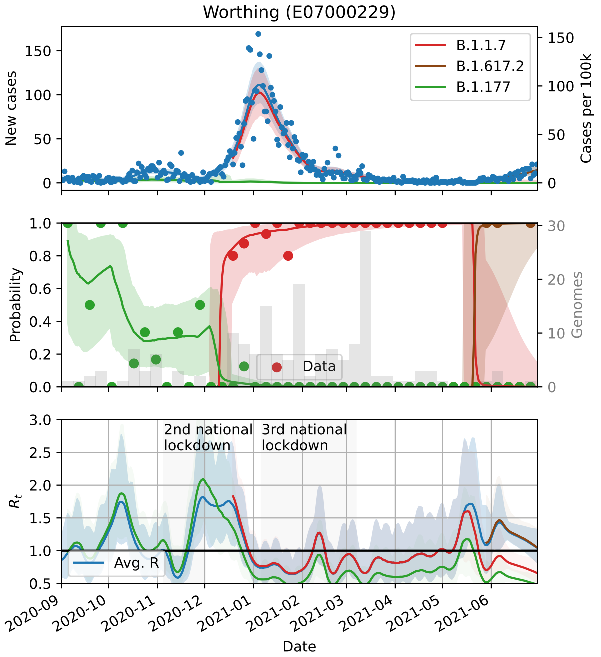
Worthing (E07000229)
New cases
Cases per 100k
B.1.1.7
B.1.617.2
B.1.177
Probability
Genomes
Data
R_t
2nd national lockdown
3rd national lockdown
Avg. R
Date

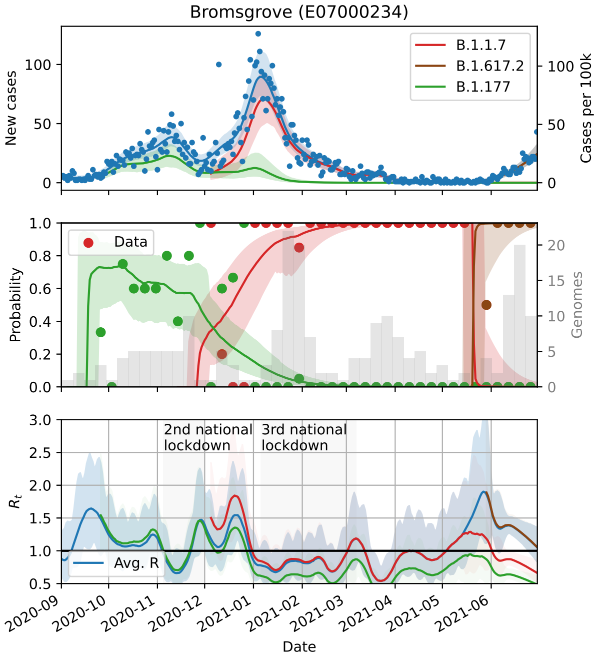
Bromsgrove (E07000234)
New cases
Cases per 100k
B.1.1.7
B.1.617.2
B.1.177
Probability
Genomes
Data
R_t
2nd national lockdown
3rd national lockdown
Avg. R
Date

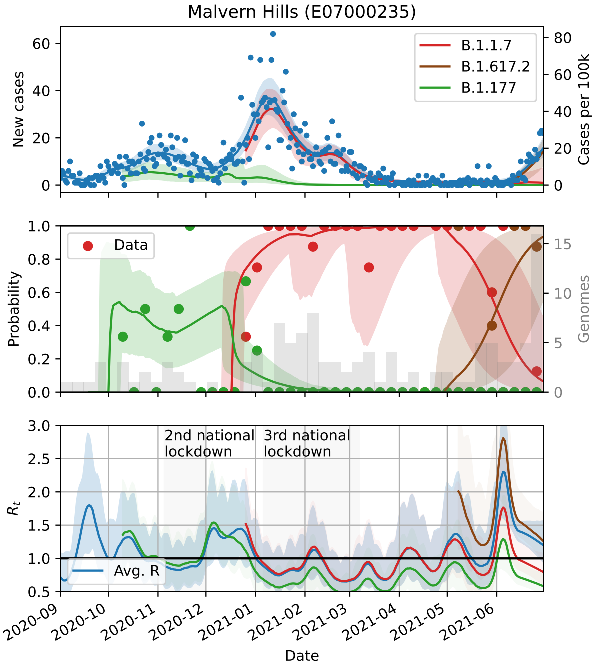
Malvern Hills (E07000235)
New cases
Cases per 100k
B.1.1.7
B.1.617.2
B.1.177
Data
Probability
Genomes
2nd national lockdown
3rd national lockdown
Avg. R
R_t
Date

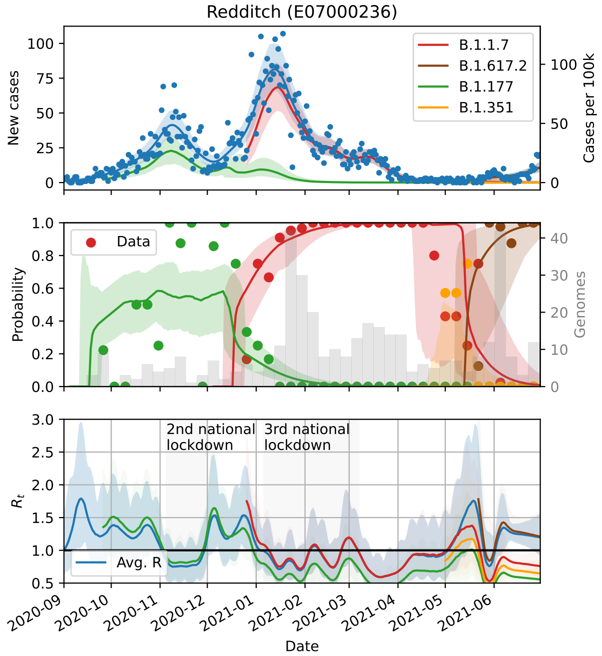
Redditch (E07000236)
New cases
Cases per 100k
B.1.1.7
B.1.617.2
B.1.177
B.1.351
Data
Probability
Genomes
2nd national lockdown
3rd national lockdown
Avg. R
R_t
Date

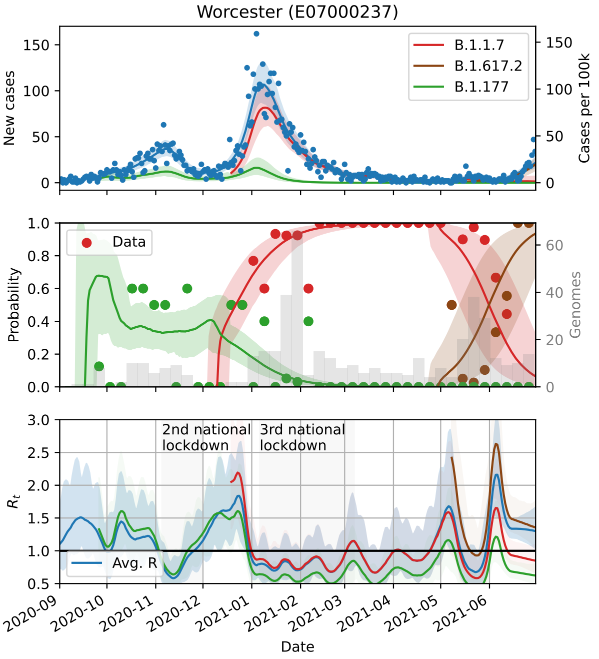
Worcester (E07000237)
New cases
Cases per 100k
B.1.1.7
B.1.617.2
B.1.177
Data
Probability
Genomes
2nd national lockdown
3rd national lockdown
Avg. R
R_t
Date

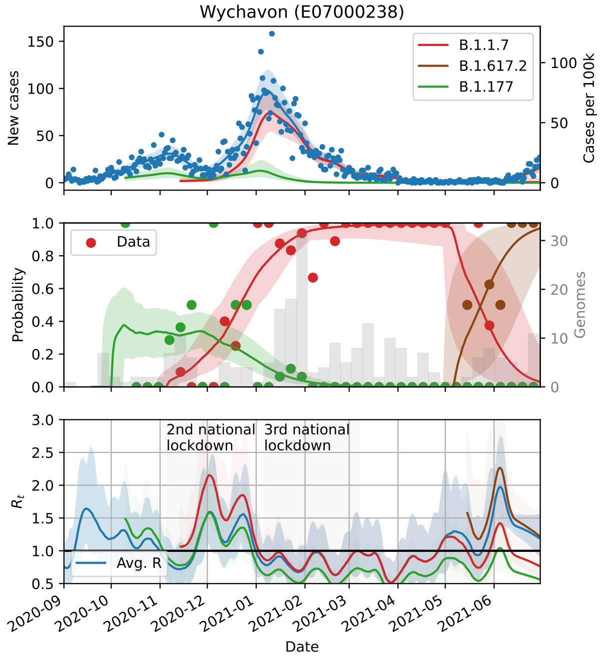
Wychavon (E07000238)
New cases
Cases per 100k
B.1.1.7
B.1.617.2
B.1.177
Data
Probability
Genomes
2nd national lockdown
3rd national lockdown
Avg. R
R_t
Date

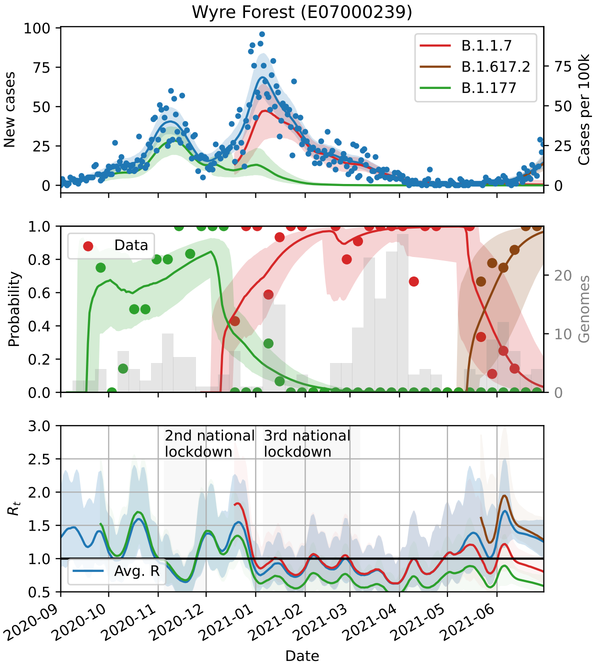
Wyre Forest (E07000239)
B.1.1.7
B.1.617.2
B.1.177
New cases
Cases per 100k
Data
Probability
Genomes
2nd national lockdown
3rd national lockdown
R_t
Avg. R
2020-09
2020-10
2020-11
2020-12
2021-01
2021-02
2021-03
2021-04
2021-05
2021-06
Date

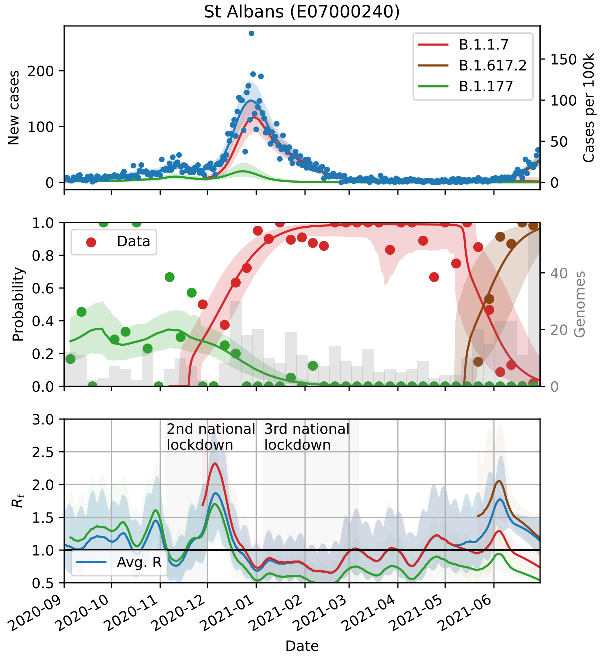
St Albans (E07000240)
B.1.1.7
B.1.617.2
B.1.177
New cases
Cases per 100k
Data
Probability
Genomes
2nd national lockdown
3rd national lockdown
R_t
Avg. R
2020-09
2020-10
2020-11
2020-12
2021-01
2021-02
2021-03
2021-04
2021-05
2021-06
Date

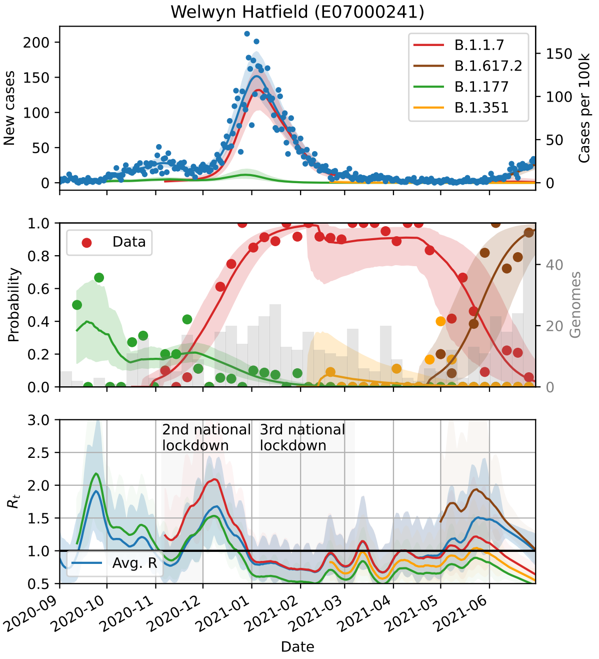
Welwyn Hatfield (E07000241)
B.1.1.7
B.1.617.2
B.1.177
B.1.351
New cases
Cases per 100k
Data
Probability
Genomes
2nd national lockdown
3rd national lockdown
R_t
Avg. R
2020-09
2020-10
2020-11
2020-12
2021-01
2021-02
2021-03
2021-04
2021-05
2021-06
Date

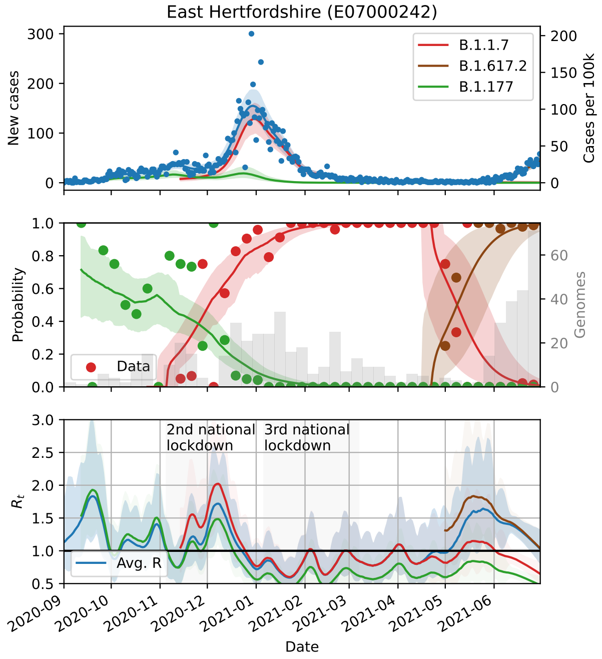
East Hertfordshire (E07000242)
B.1.1.7
B.1.617.2
B.1.177
New cases
Cases per 100k
Data
Probability
Genomes
2nd national lockdown
3rd national lockdown
R_t
Avg. R
2020-09
2020-10
2020-11
2020-12
2021-01
2021-02
2021-03
2021-04
2021-05
2021-06
Date

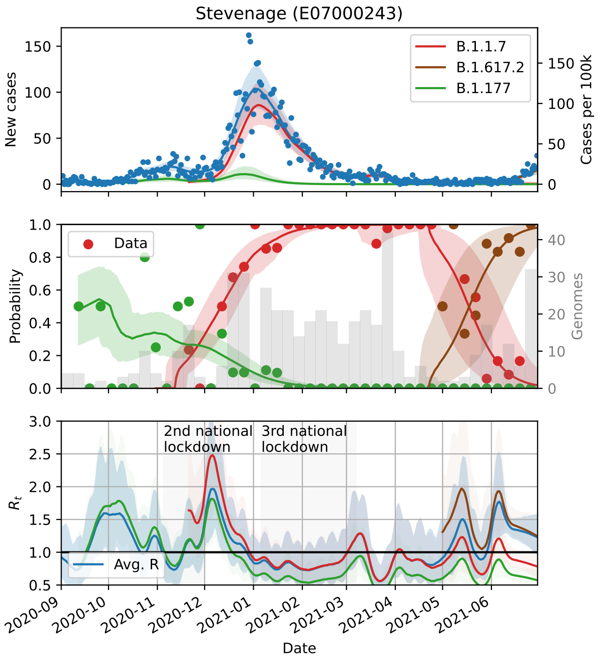
Stevenage (E07000243)
New cases
Cases per 100k
B.1.1.7
B.1.617.2
B.1.177
Data
Probability
Genomes
2nd national lockdown
3rd national lockdown
R_t
Avg. R
Date

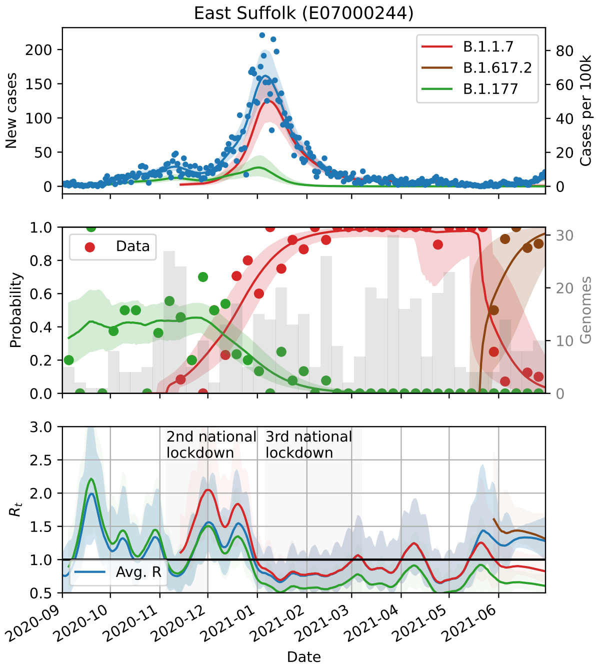
East Suffolk (E07000244)
New cases
Cases per 100k
B.1.1.7
B.1.617.2
B.1.177
Data
Probability
Genomes
2nd national lockdown
3rd national lockdown
R_t
Avg. R
Date

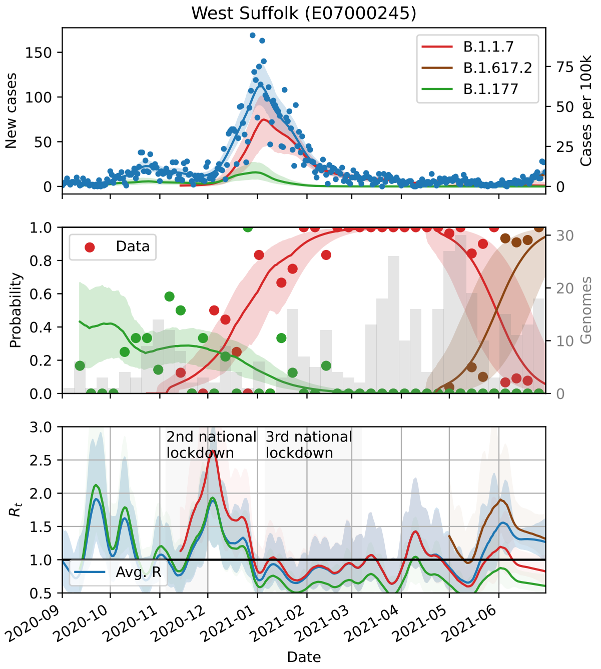
West Suffolk (E07000245)
New cases
Cases per 100k
B.1.1.7
B.1.617.2
B.1.177
Data
Probability
Genomes
2nd national lockdown
3rd national lockdown
R_t
Avg. R
Date

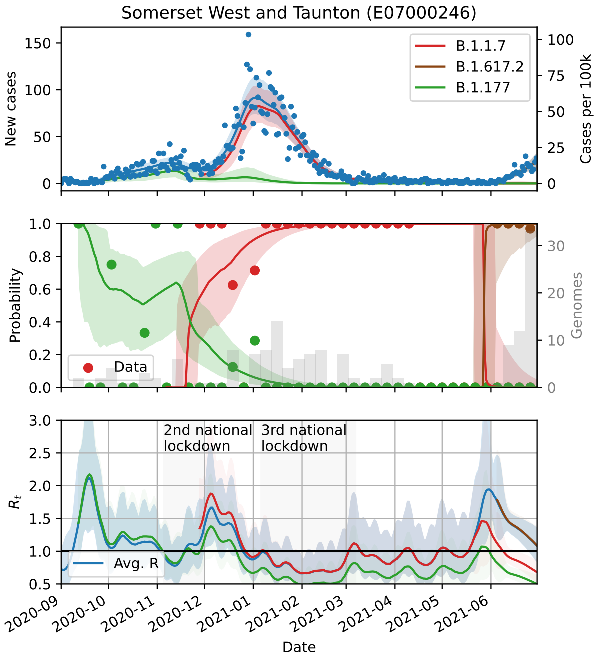
Somerset West and Taunton (E07000246)
New cases
Cases per 100k
B.1.1.7
B.1.617.2
B.1.177
Data
Probability
Genomes
2nd national lockdown
3rd national lockdown
R_t
Avg. R
Date

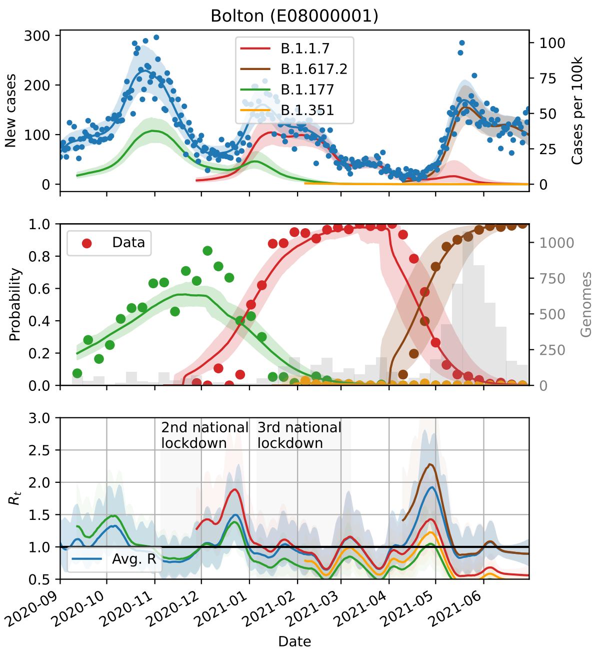

Bolton (E08000001)
New cases
Cases per 100k
B.1.1.7
B.1.617.2
B.1.177
B.1.351
Data
Probability
Genomes
2nd national lockdown
3rd national lockdown
Avg. R
R_t
Date

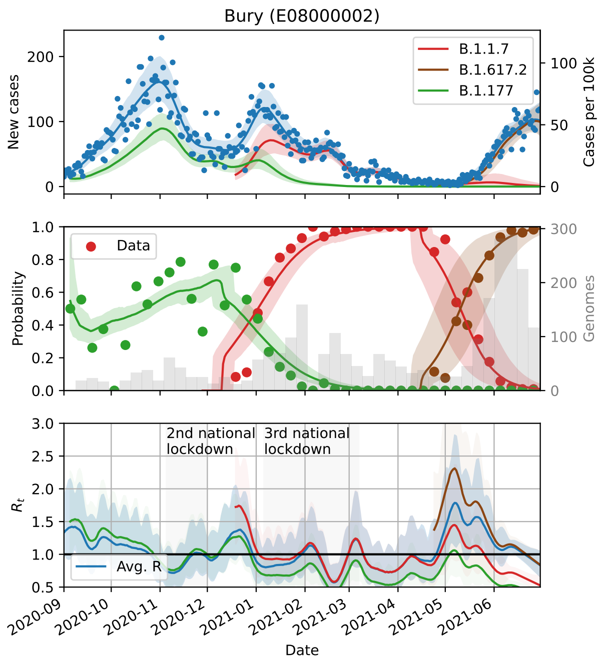

Bury (E08000002)
New cases
Cases per 100k
B.1.1.7
B.1.617.2
B.1.177
Data
Probability
Genomes
2nd national lockdown
3rd national lockdown
Avg. R
R_t
Date

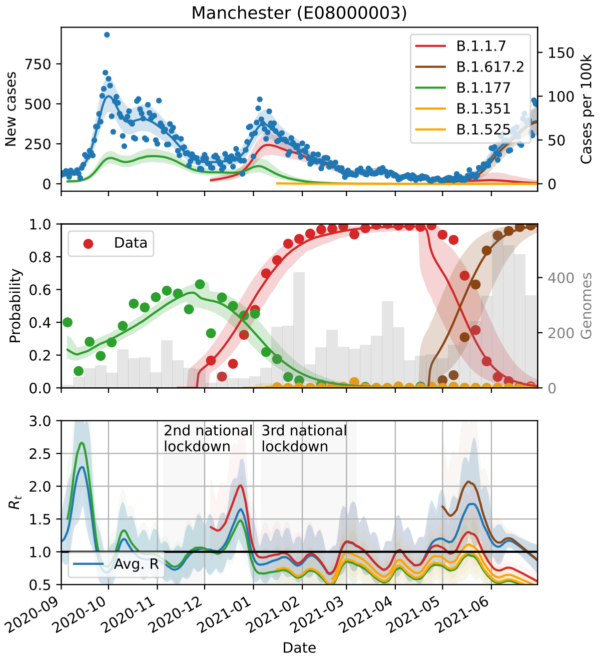

Manchester (E08000003)
New cases
Cases per 100k
B.1.1.7
B.1.617.2
B.1.177
B.1.351
B.1.525
Data
Probability
Genomes
2nd national lockdown
3rd national lockdown
Avg. R
R_t
Date

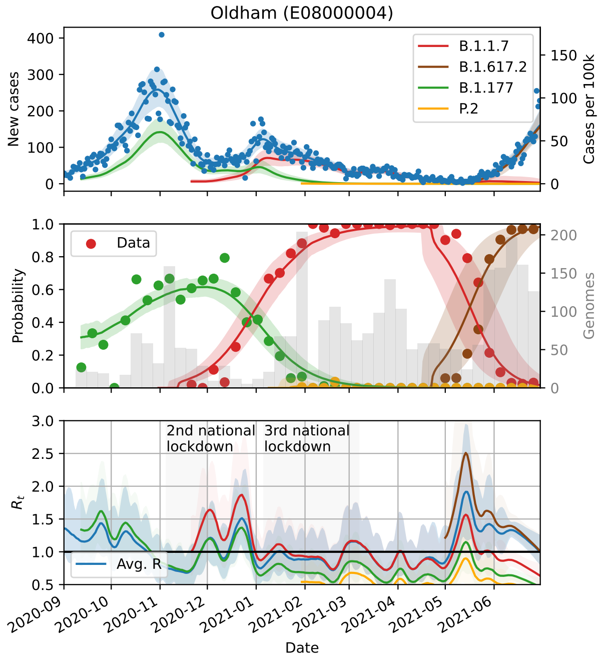

Oldham (E08000004)
New cases
Cases per 100k
B.1.1.7
B.1.617.2
B.1.177
P.2
Data
Probability
Genomes
2nd national lockdown
3rd national lockdown
Avg. R
R_t
Date

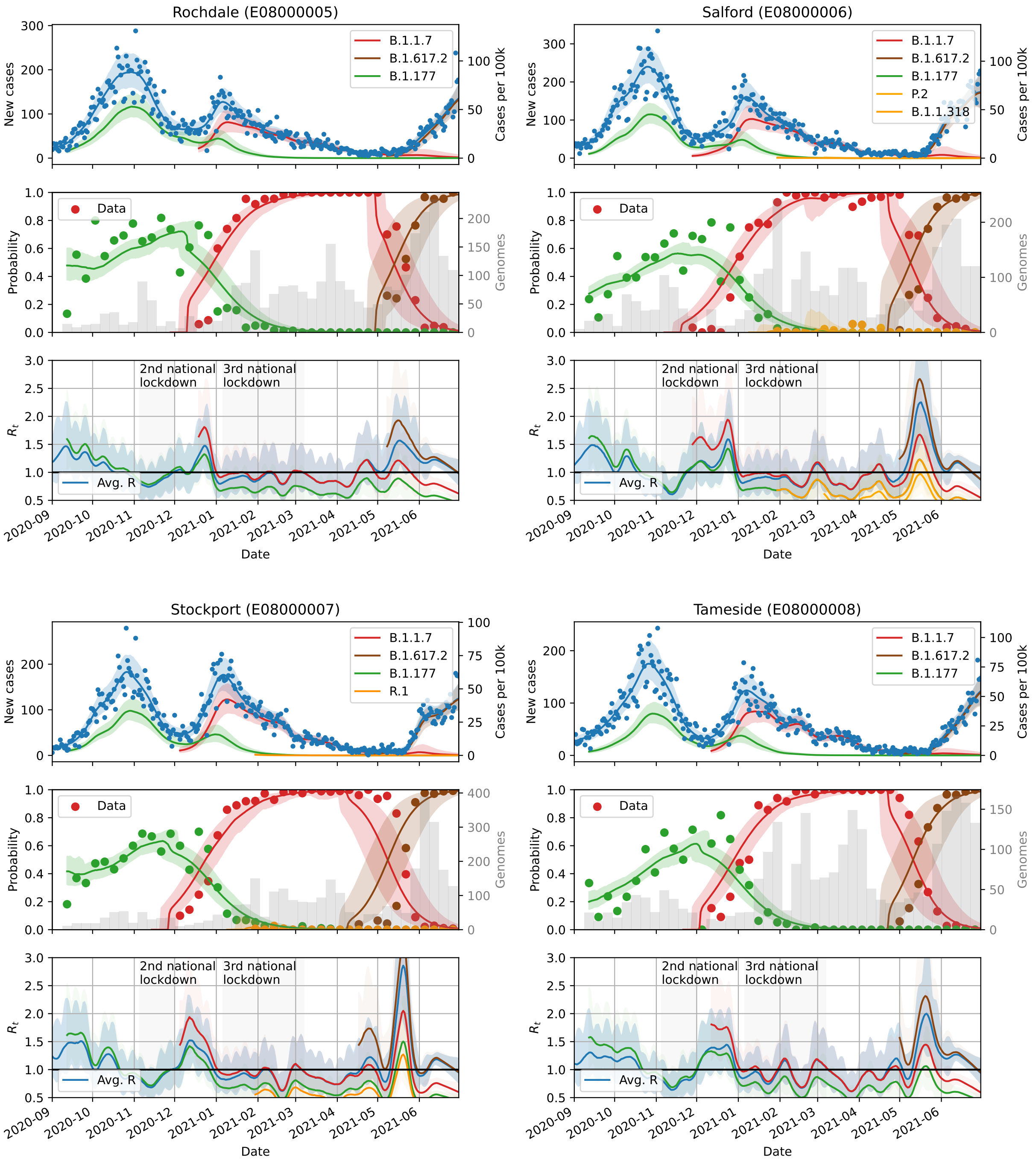

Rochdale (E08000005)
Salford (E08000006)
Stockport (E08000007)
Tameside (E08000008)
New cases
Cases per 100k
B.1.1.7
B.1.617.2
B.1.177
P.2
B.1.1.318
R.1
Probability
Genomes
Data
R_t
2nd national lockdown
3rd national lockdown
Avg. R
2020-09
2020-10
2020-11
2020-12
2021-01
2021-02
2021-03
2021-04
2021-05
2021-06
Date

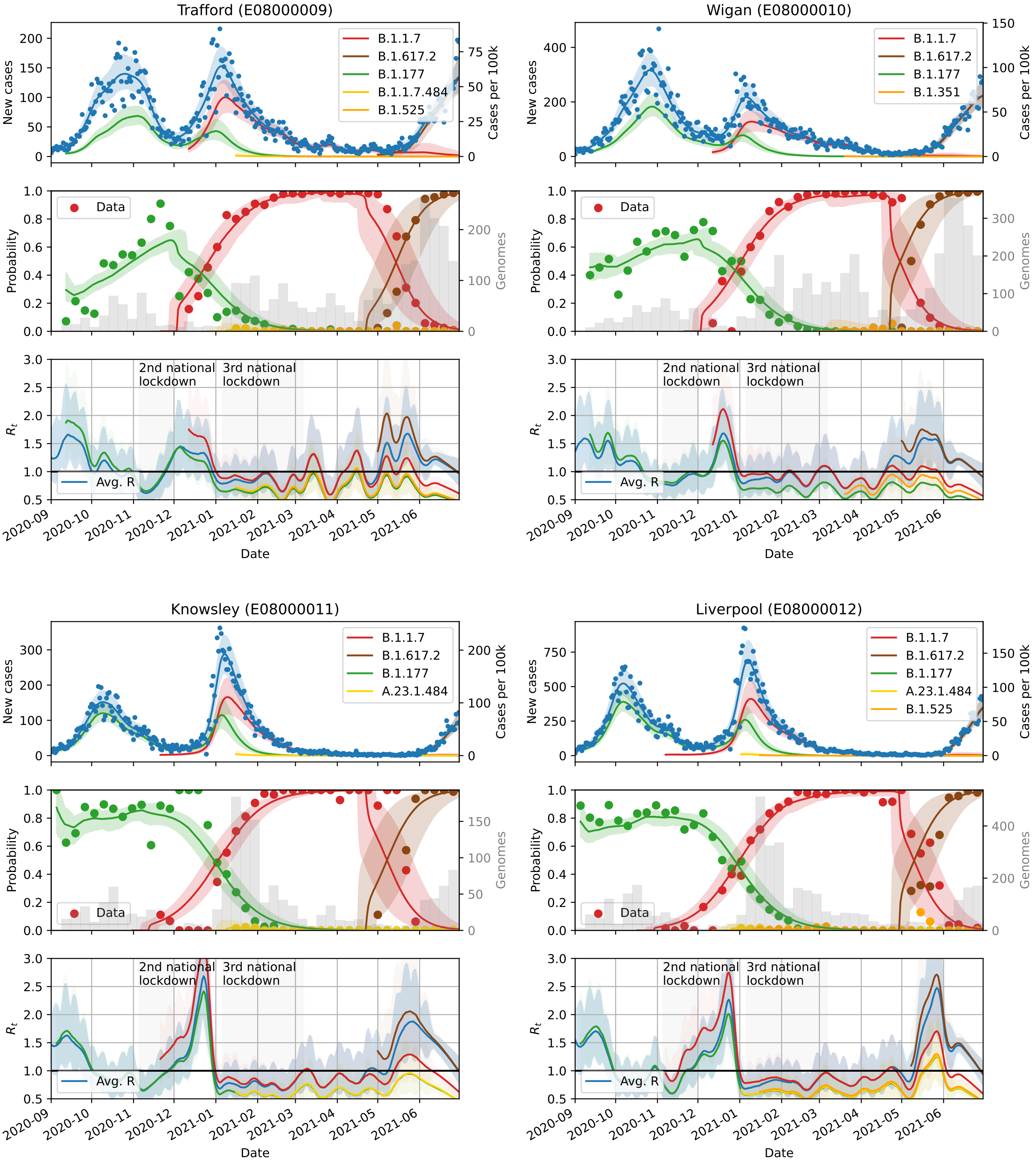

Trafford (E08000009)
B.1.1.7
B.1.617.2
B.1.177
B.1.1.7.484
B.1.525
New cases
Cases per 100k
Data
Probability
Genomes
2nd national lockdown
3rd national lockdown
Avg. R
R_t
Date
Wigan (E08000010)
B.1.1.7
B.1.617.2
B.1.177
B.1.351
Knowsley (E08000011)
B.1.1.7
B.1.617.2
B.1.177
A.23.1.484
Liverpool (E08000012)
B.1.1.7
B.1.617.2
B.1.177
A.23.1.484
B.1.525
2020-09
2020-10
2020-11
2020-12
2021-01
2021-02
2021-03
2021-04
2021-05
2021-06

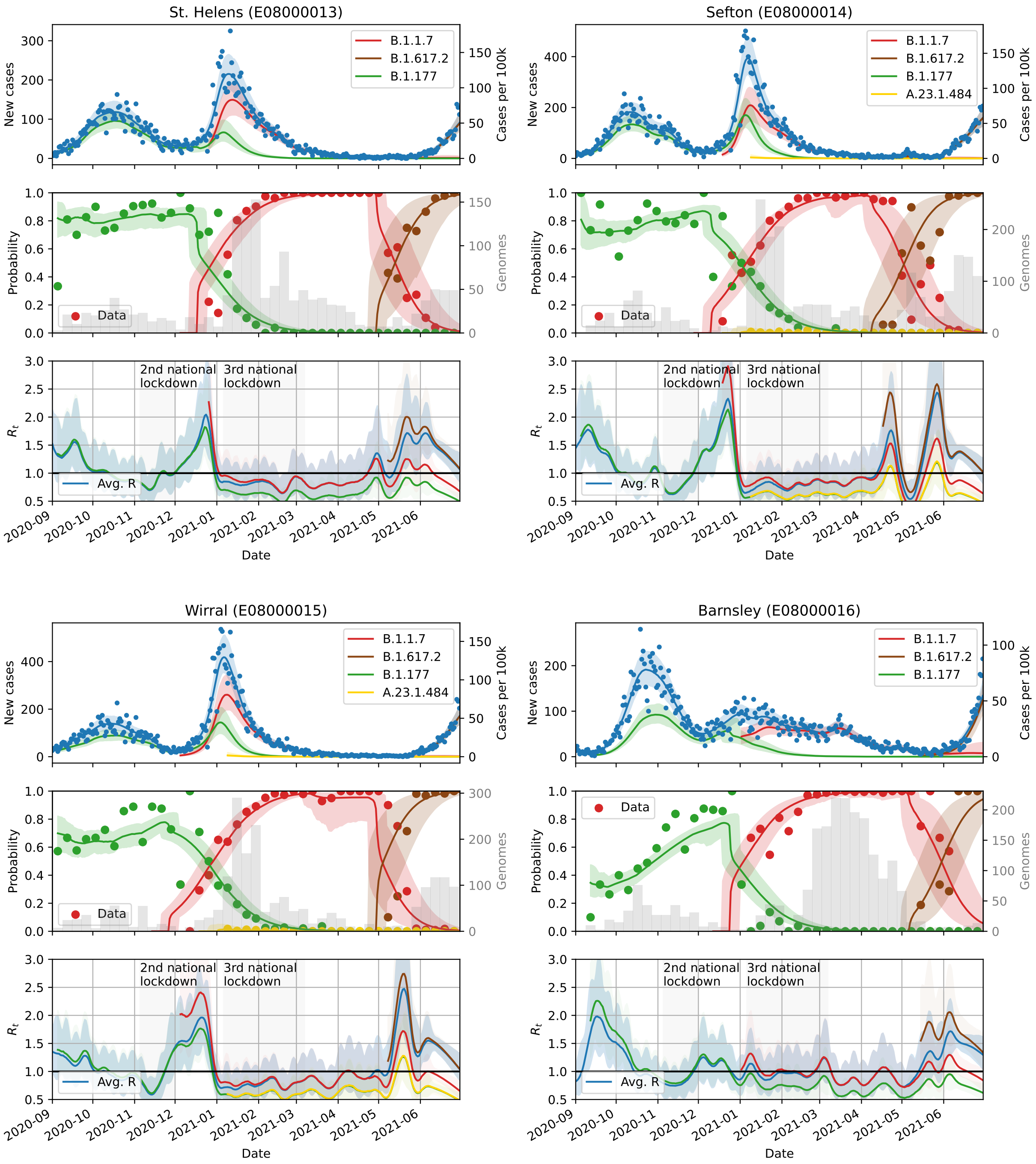

St. Helens (E08000013)
Sefton (E08000014)
Wirral (E08000015)
Barnsley (E08000016)
B.1.1.7
B.1.617.2
B.1.177
A.23.1.484
New cases
Cases per 100k
Probability
Genomes
Data
2nd national lockdown
3rd national lockdown
Avg. R
R_t
Date
2020-09
2020-10
2020-11
2020-12
2021-01
2021-02
2021-03
2021-04
2021-05
2021-06

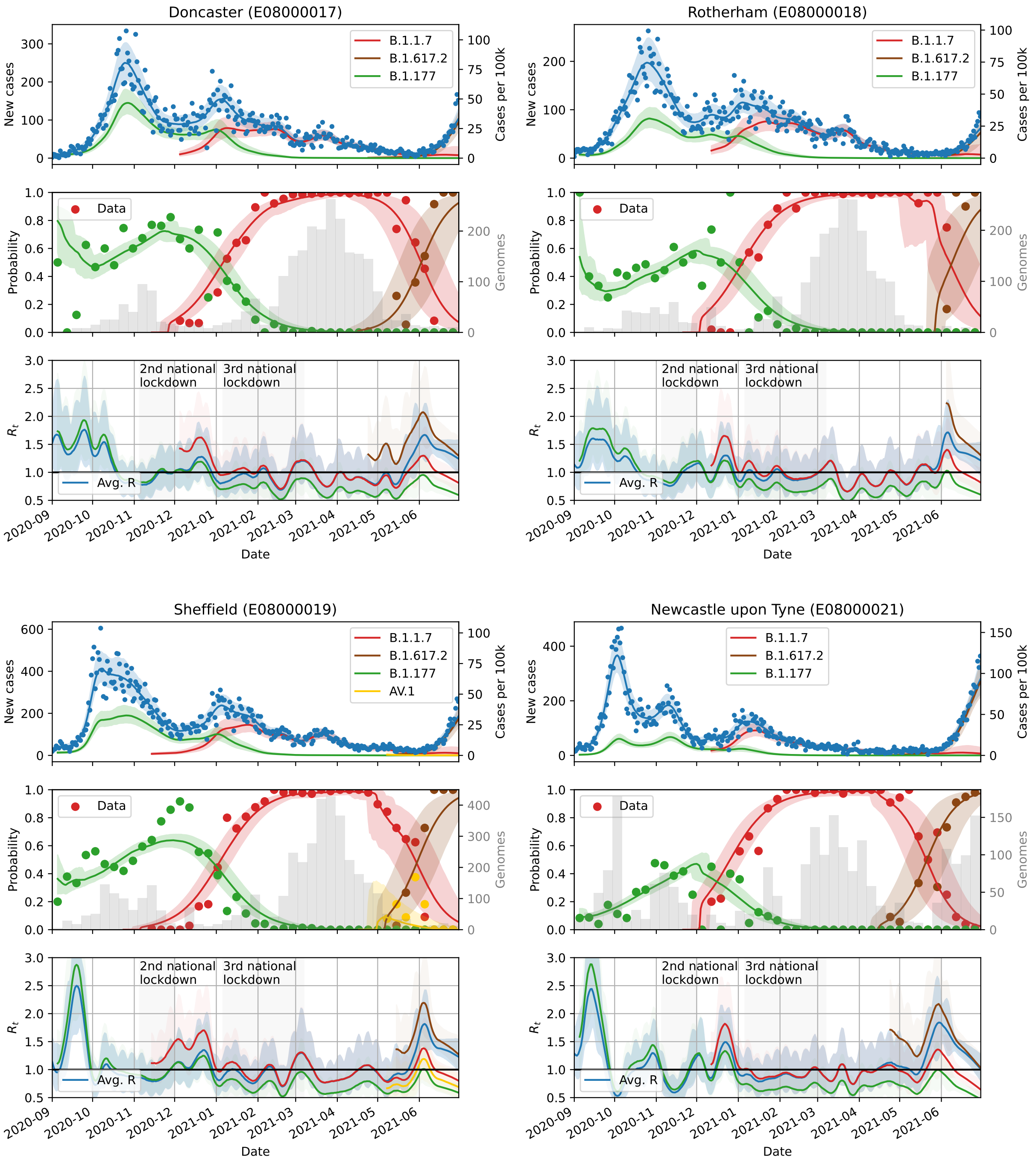
Doncaster (E08000017)
Rotherham (E08000018)
Sheffield (E08000019)
Newcastle upon Tyne (E08000021)
B.1.1.7
B.1.617.2
B.1.177
AV.1
Data
Avg. R
New cases
Cases per 100k
Probability
Genomes
R_t
2nd national lockdown
3rd national lockdown
2020-09
2020-10
2020-11
2020-12
2021-01
2021-02
2021-03
2021-04
2021-05
2021-06
Date

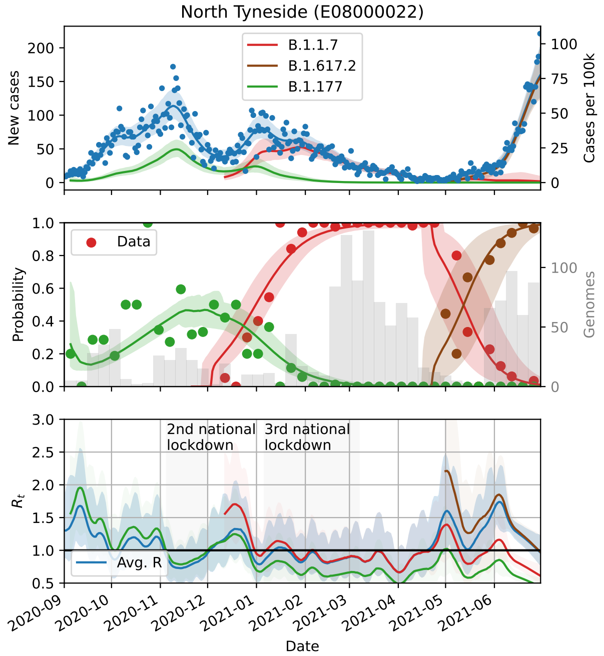
North Tyneside (E08000022)
B.1.1.7
B.1.617.2
B.1.177
New cases
Cases per 100k
Data
Probability
Genomes
2nd national lockdown
3rd national lockdown
Avg. R
R_t
Date

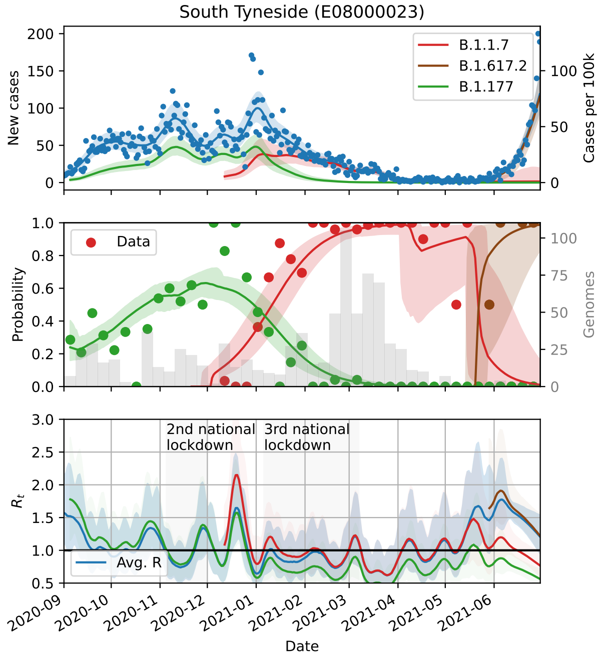
South Tyneside (E08000023)
B.1.1.7
B.1.617.2
B.1.177
New cases
Cases per 100k
Data
Probability
Genomes
2nd national lockdown
3rd national lockdown
Avg. R
R_t
Date

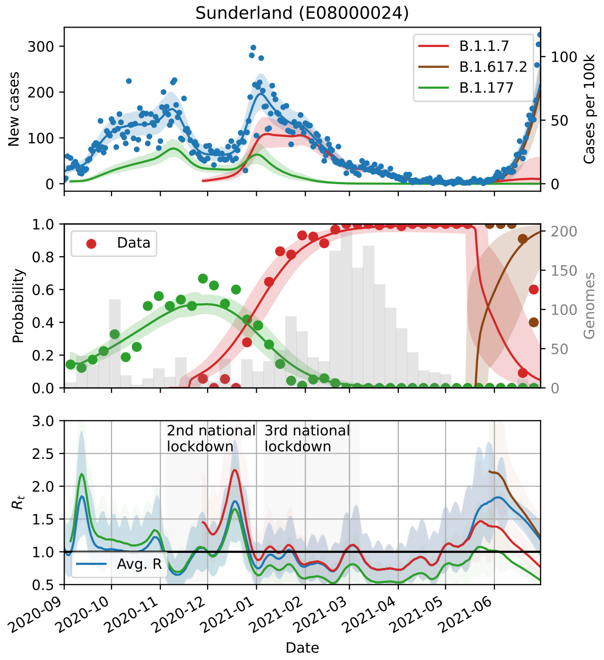
Sunderland (E08000024)
B.1.1.7
B.1.617.2
B.1.177
New cases
Cases per 100k
Data
Probability
Genomes
2nd national lockdown
3rd national lockdown
Avg. R
R_t
Date

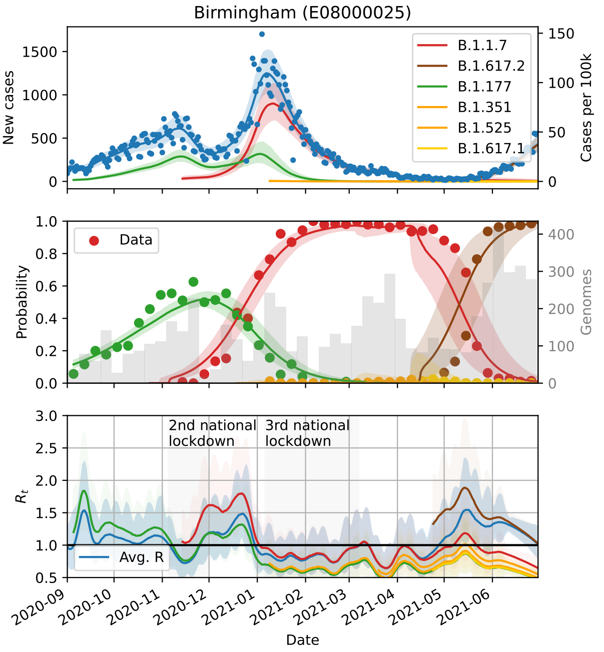
Birmingham (E08000025)
B.1.1.7
B.1.617.2
B.1.177
B.1.351
B.1.525
B.1.617.1
New cases
Cases per 100k
Data
Probability
Genomes
2nd national lockdown
3rd national lockdown
Avg. R
R_t
Date

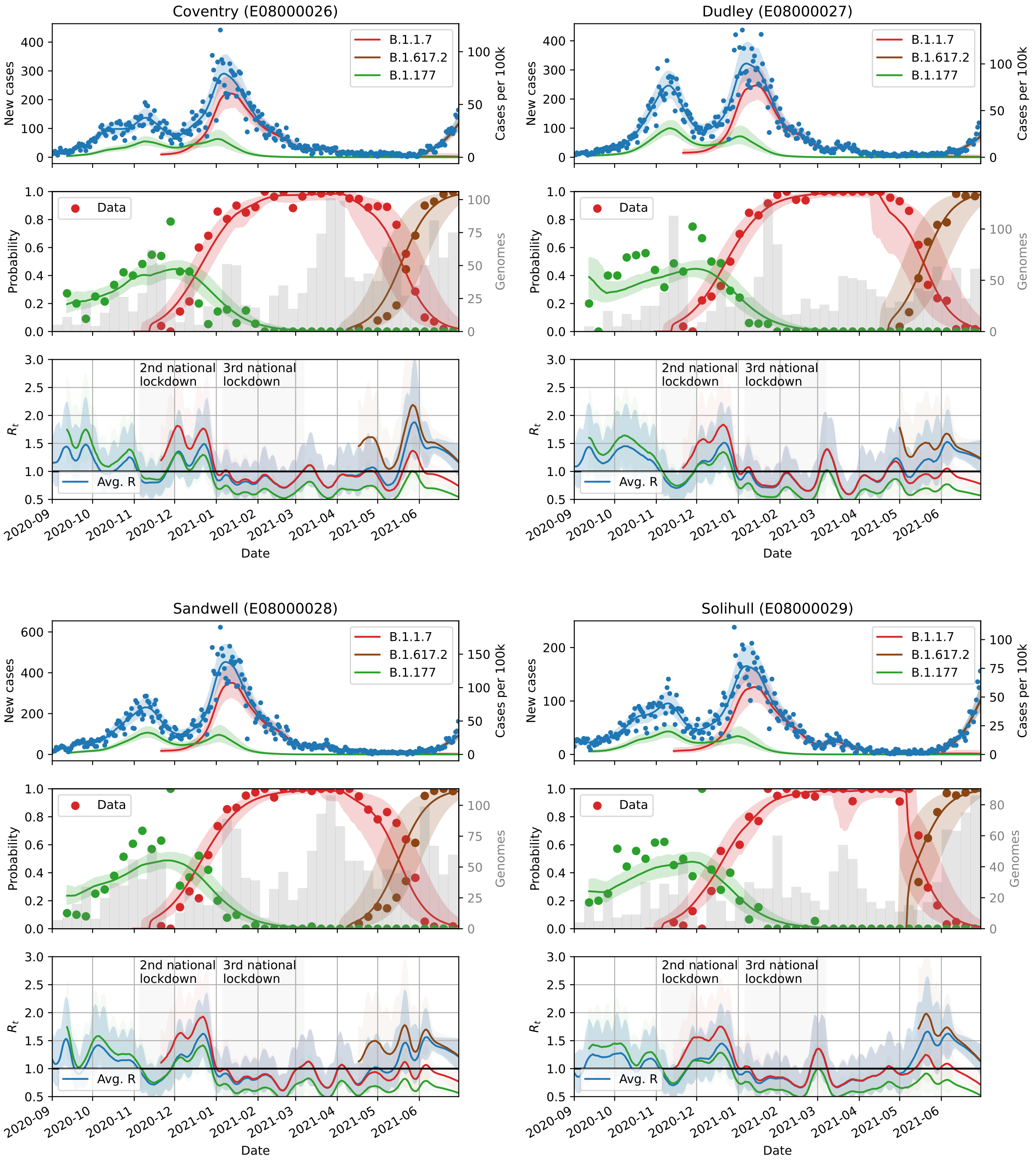
Coventry (E08000026)
Dudley (E08000027)
Sandwell (E08000028)
Solihull (E08000029)
New cases
Cases per 100k
B.1.1.7
B.1.617.2
B.1.177
Data
Probability
Genomes
R_t
Avg. R
2nd national lockdown
3rd national lockdown
2020-09
2020-10
2020-11
2020-12
2021-01
2021-02
2021-03
2021-04
2021-05
2021-06
Date

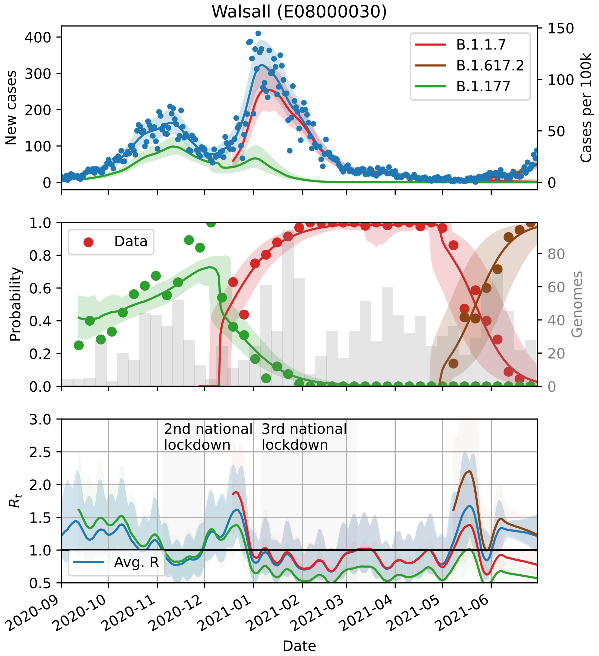

Walsall (E08000030)
New cases
Cases per 100k
B.1.1.7
B.1.617.2
B.1.177
Data
Probability
Genomes
2nd national lockdown
3rd national lockdown
R_t
Avg. R
Date

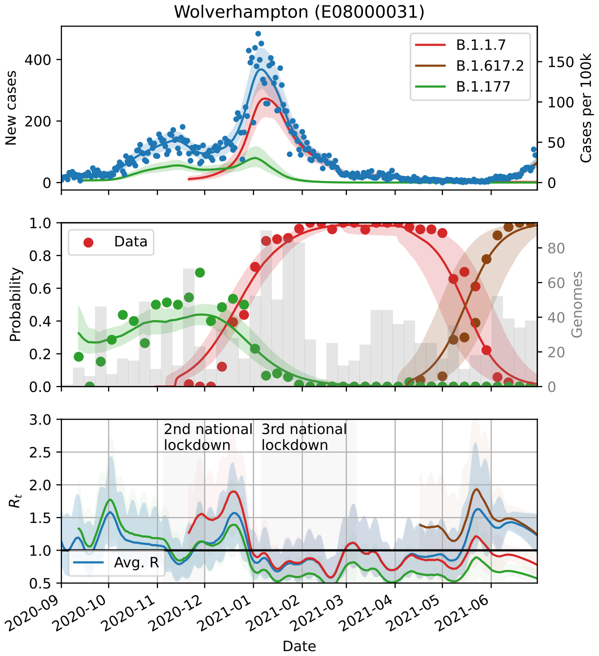

Wolverhampton (E08000031)
New cases
Cases per 100k
B.1.1.7
B.1.617.2
B.1.177
Data
Probability
Genomes
2nd national lockdown
3rd national lockdown
R_t
Avg. R
Date

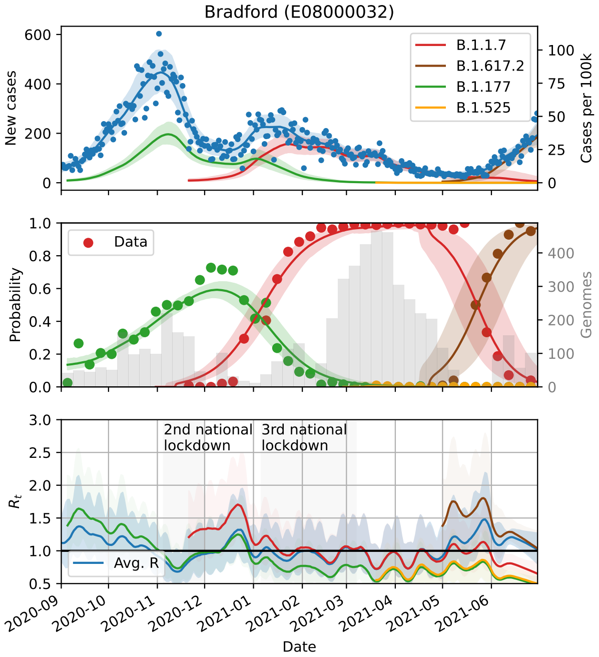

Bradford (E08000032)
New cases
Cases per 100k
B.1.1.7
B.1.617.2
B.1.177
B.1.525
Data
Probability
Genomes
2nd national lockdown
3rd national lockdown
R_t
Avg. R
Date

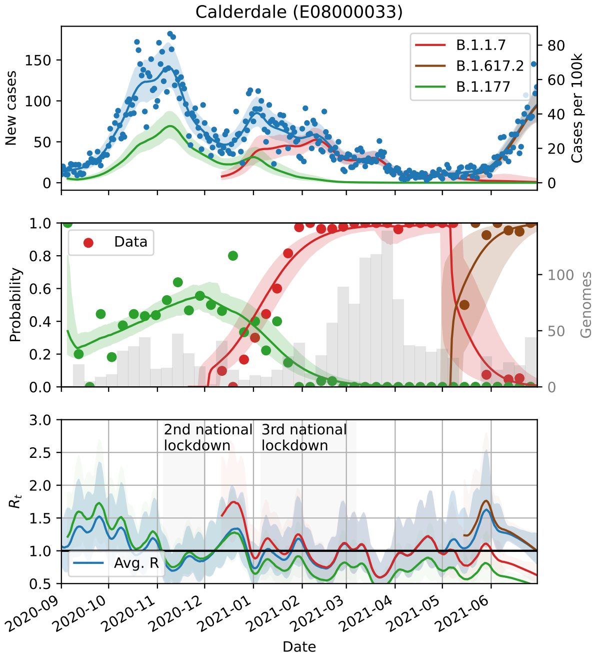

Calderdale (E08000033)
New cases
Cases per 100k
B.1.1.7
B.1.617.2
B.1.177
Data
Probability
Genomes
2nd national lockdown
3rd national lockdown
R_t
Avg. R
Date

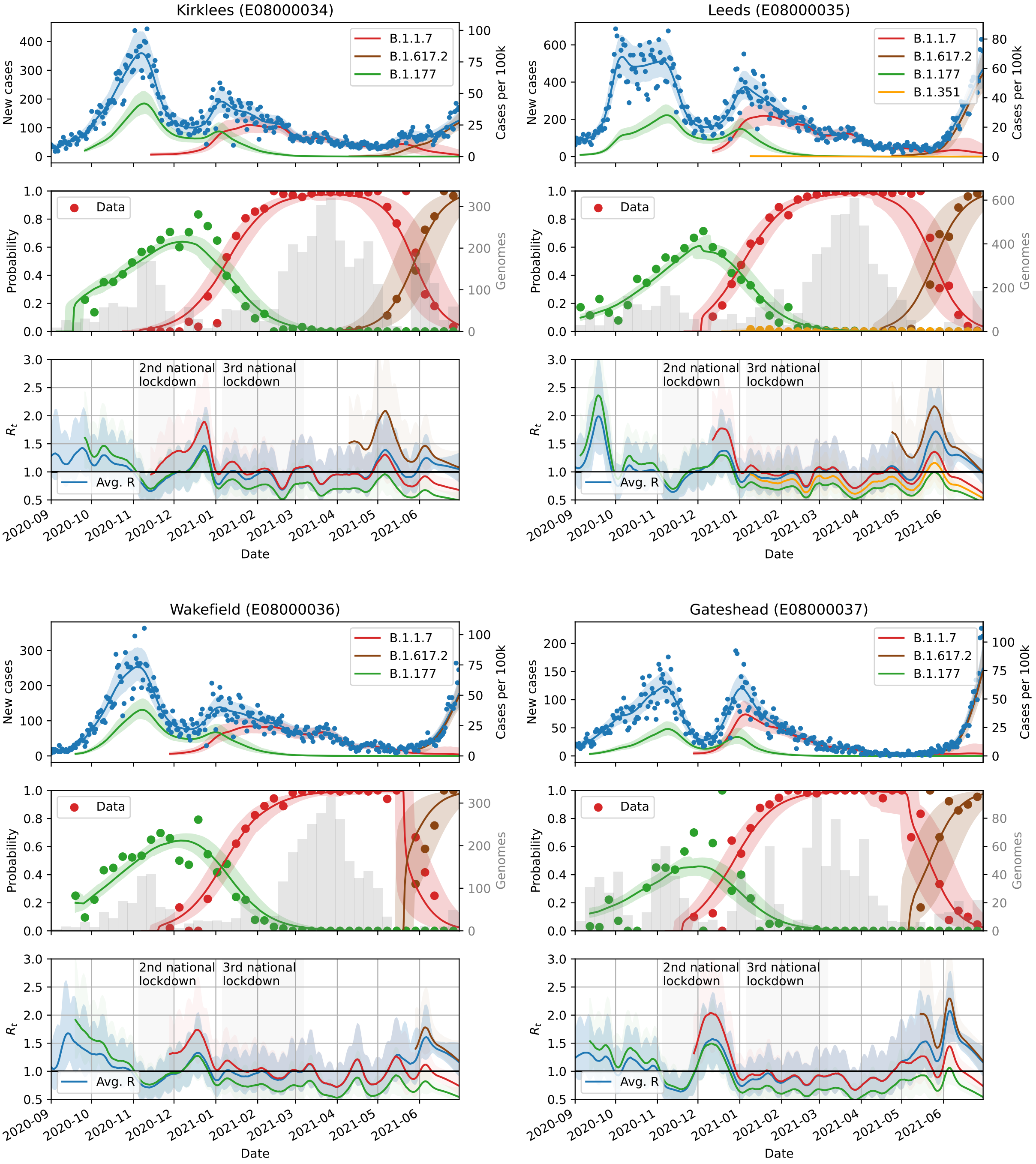

Kirklees (E08000034)
Leeds (E08000035)
Wakefield (E08000036)
Gateshead (E08000037)
New cases
Cases per 100k
B.1.1.7
B.1.617.2
B.1.177
B.1.351
Probability
Genomes
Data
R_t
2nd national lockdown
3rd national lockdown
Avg. R
Date
2020-09
2020-10
2020-11
2020-12
2021-01
2021-02
2021-03
2021-04
2021-05
2021-06

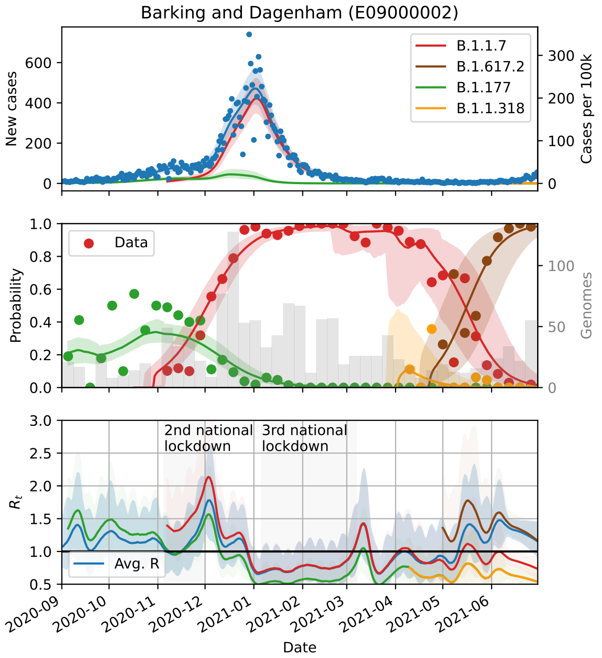
Barking and Dagenham (E09000002)
New cases
Cases per 100k
B.1.1.7
B.1.617.2
B.1.177
B.1.1.318
Probability
Data
Genomes
2nd national lockdown
3rd national lockdown
R_t
Avg. R
Date

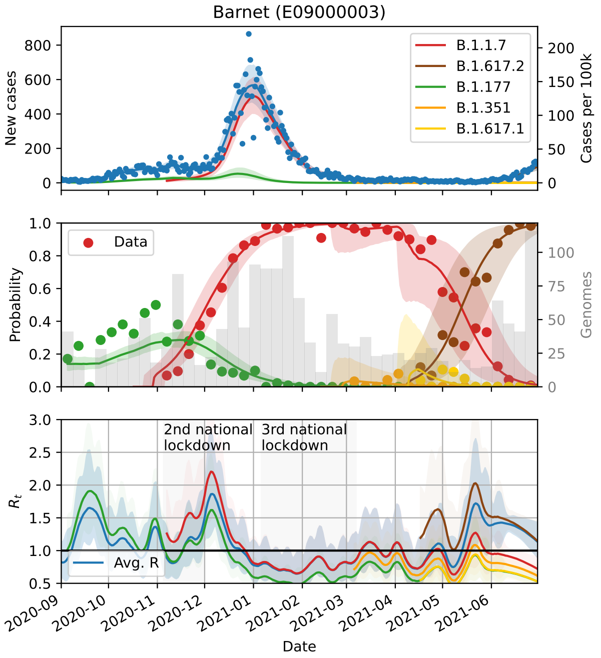
Barnet (E09000003)
New cases
Cases per 100k
B.1.1.7
B.1.617.2
B.1.177
B.1.351
B.1.617.1
Probability
Data
Genomes
2nd national lockdown
3rd national lockdown
R_t
Avg. R
Date

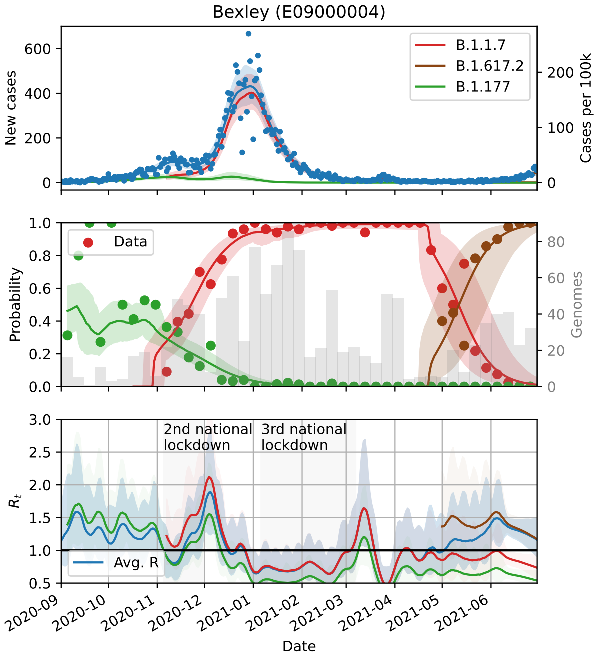
Bexley (E09000004)
New cases
Cases per 100k
B.1.1.7
B.1.617.2
B.1.177
Probability
Data
Genomes
2nd national lockdown
3rd national lockdown
R_t
Avg. R
Date

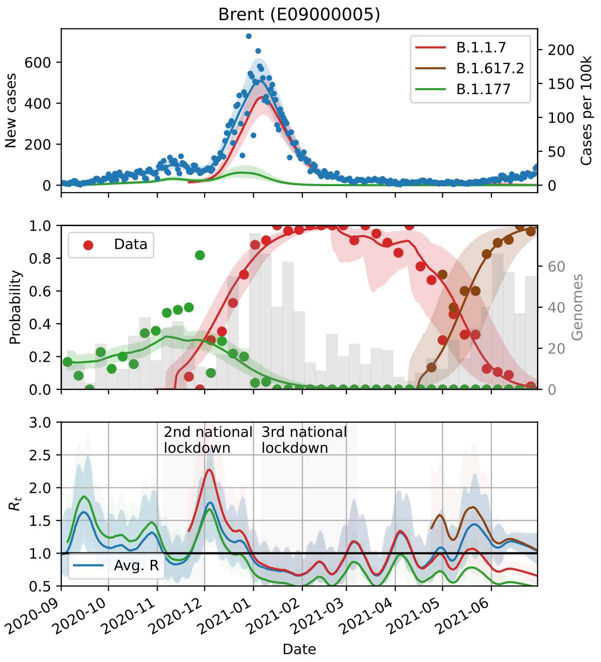
Brent (E09000005)
B.1.1.7
B.1.617.2
B.1.177
New cases
Cases per 100k
Data
Probability
Genomes
2nd national lockdown
3rd national lockdown
Avg. R
R_t
Date

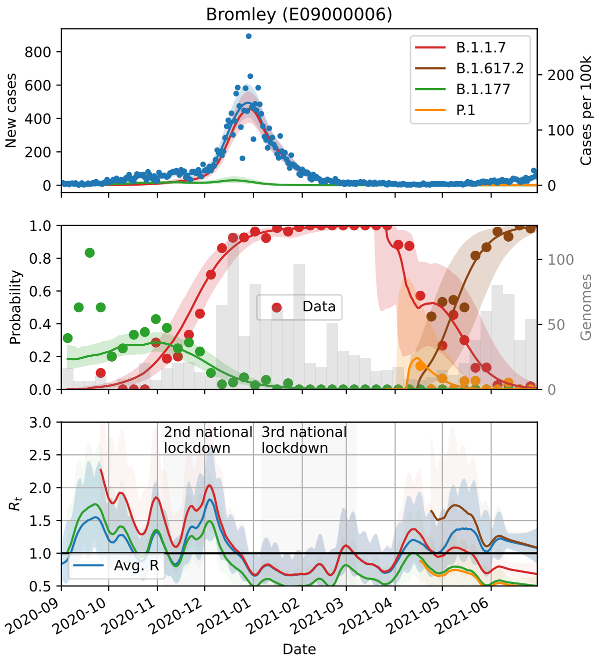
Bromley (E09000006)
B.1.1.7
B.1.617.2
B.1.177
P.1
New cases
Cases per 100k
Data
Probability
Genomes
2nd national lockdown
3rd national lockdown
Avg. R
R_t
Date

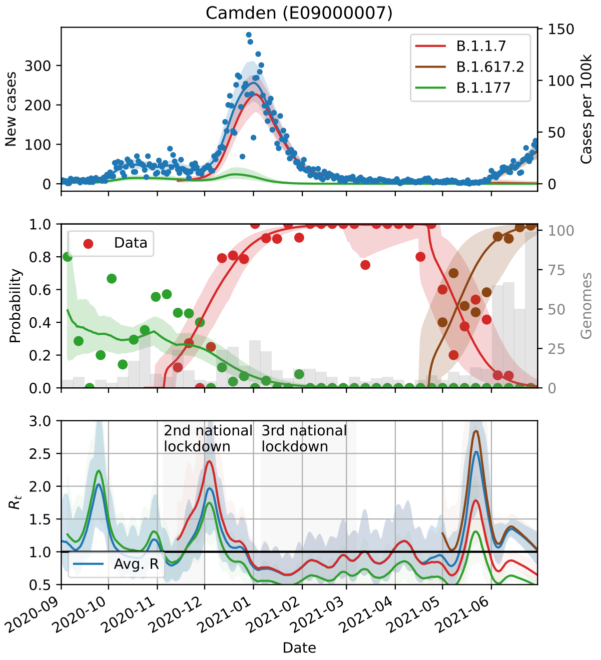
Camden (E09000007)
B.1.1.7
B.1.617.2
B.1.177
New cases
Cases per 100k
Data
Probability
Genomes
2nd national lockdown
3rd national lockdown
Avg. R
R_t
Date

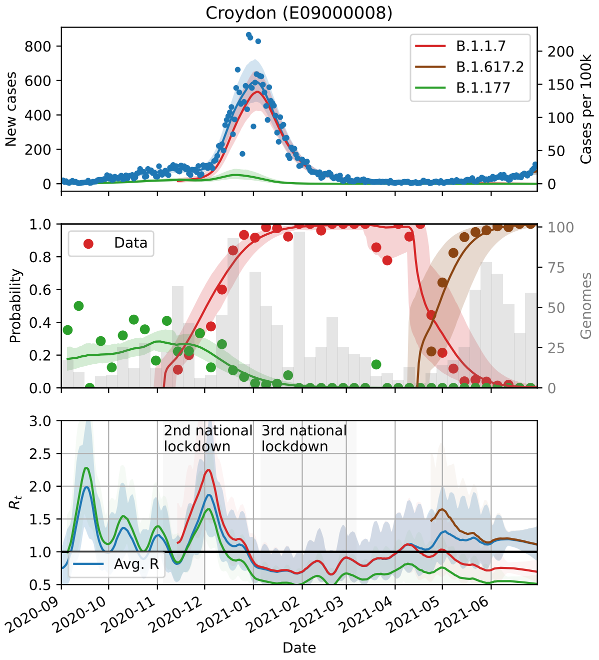
Croydon (E09000008)
B.1.1.7
B.1.617.2
B.1.177
New cases
Cases per 100k
Data
Probability
Genomes
2nd national lockdown
3rd national lockdown
Avg. R
R_t
Date

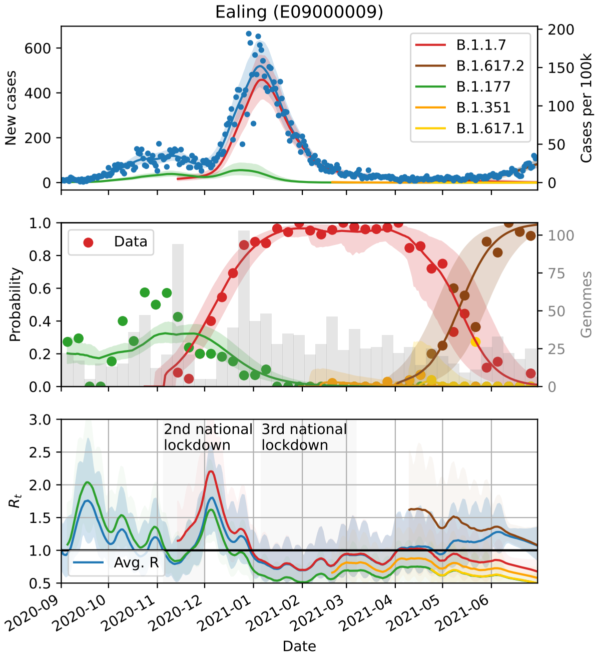

Ealing (E09000009)
New cases
Cases per 100k
B.1.1.7
B.1.617.2
B.1.177
B.1.351
B.1.617.1
Data
Probability
Genomes
2nd national lockdown
3rd national lockdown
Avg. R
Date

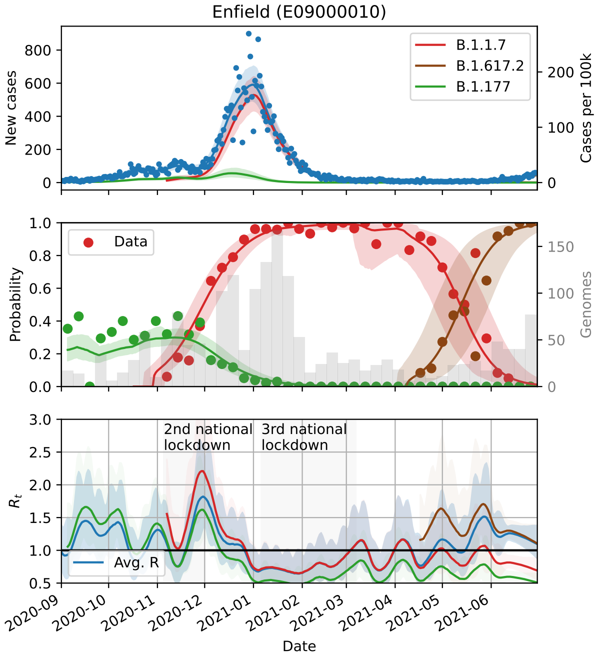

Enfield (E09000010)
New cases
Cases per 100k
B.1.1.7
B.1.617.2
B.1.177
Data
Probability
Genomes
2nd national lockdown
3rd national lockdown
Avg. R
Date

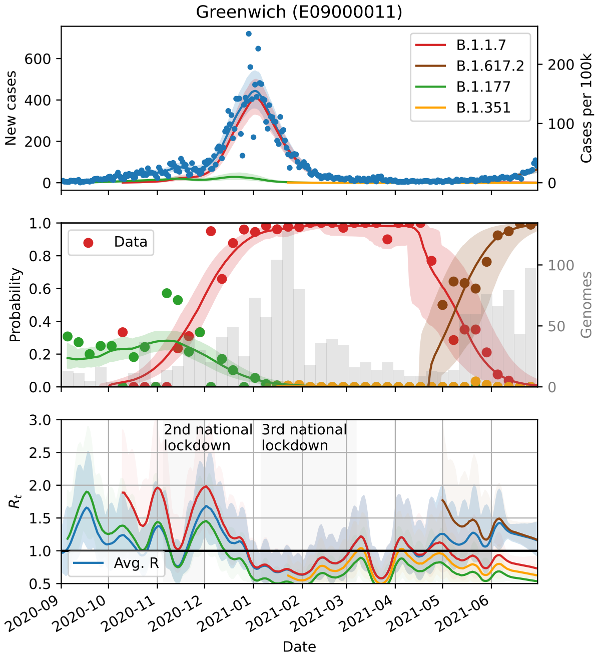

Greenwich (E09000011)
New cases
Cases per 100k
B.1.1.7
B.1.617.2
B.1.177
B.1.351
Data
Probability
Genomes
2nd national lockdown
3rd national lockdown
Avg. R
Date

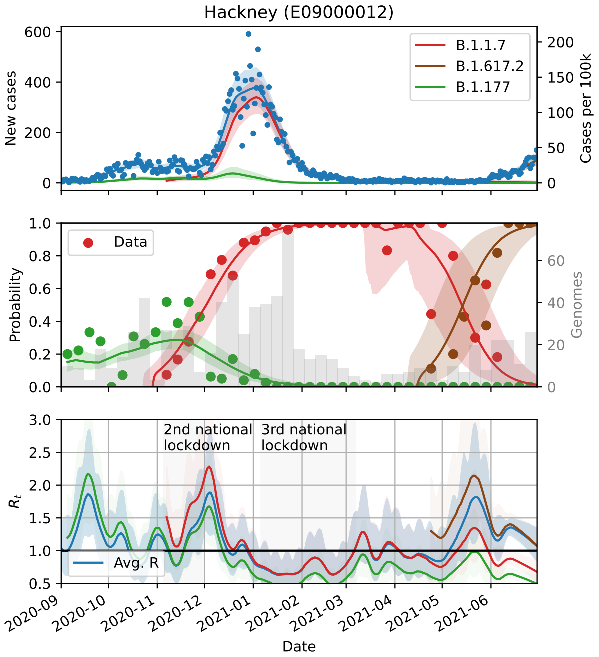

Hackney (E09000012)
New cases
Cases per 100k
B.1.1.7
B.1.617.2
B.1.177
Data
Probability
Genomes
2nd national lockdown
3rd national lockdown
Avg. R
Date

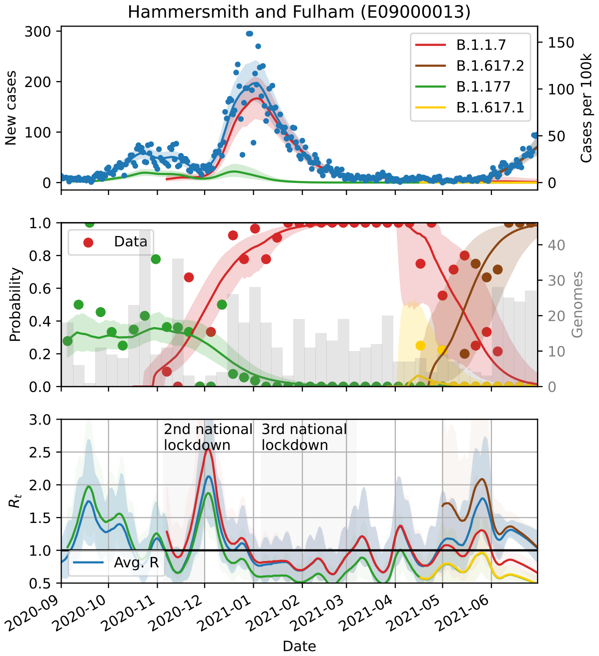
Hammersmith and Fulham (E09000013)
New cases
Cases per 100k
B.1.1.7
B.1.617.2
B.1.177
B.1.617.1
Data
Probability
Genomes
2nd national lockdown
3rd national lockdown
R_t
Avg. R
Date

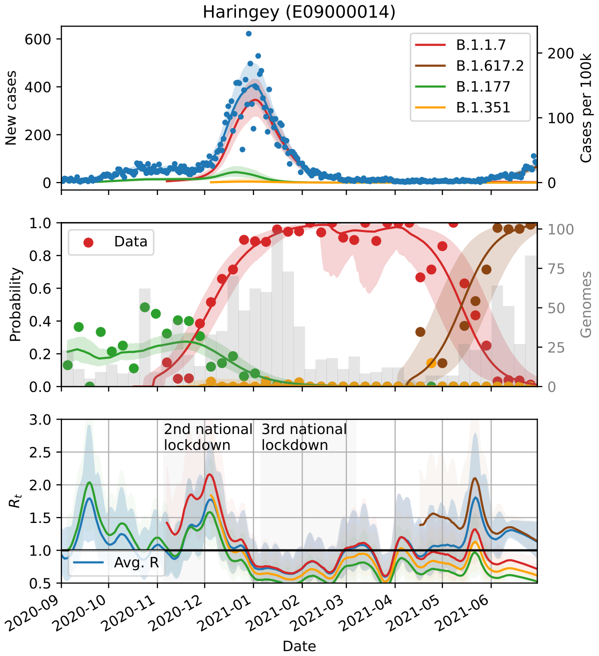
Haringey (E09000014)
New cases
Cases per 100k
B.1.1.7
B.1.617.2
B.1.177
B.1.351
Data
Probability
Genomes
2nd national lockdown
3rd national lockdown
R_t
Avg. R
Date

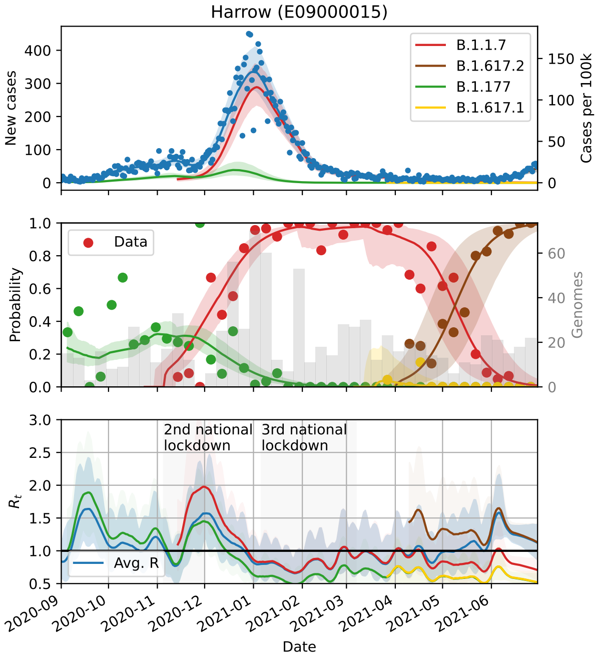
Harrow (E09000015)
New cases
Cases per 100k
B.1.1.7
B.1.617.2
B.1.177
B.1.617.1
Data
Probability
Genomes
2nd national lockdown
3rd national lockdown
R_t
Avg. R
Date

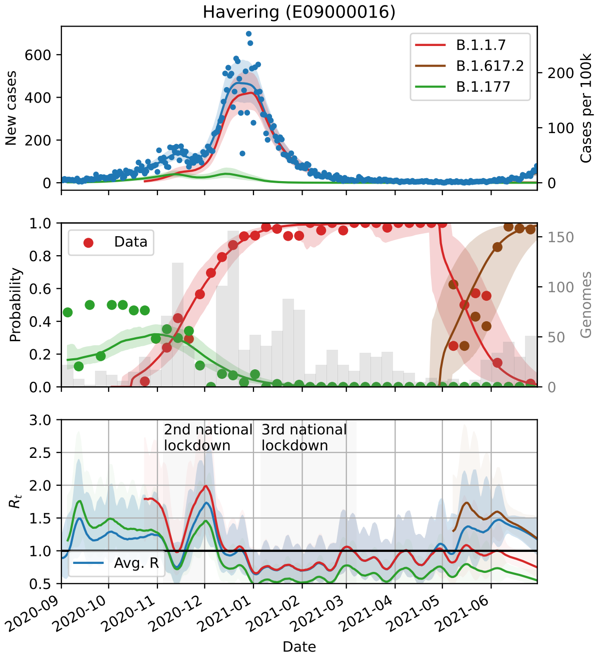
Havering (E09000016)
New cases
Cases per 100k
B.1.1.7
B.1.617.2
B.1.177
Data
Probability
Genomes
2nd national lockdown
3rd national lockdown
R_t
Avg. R
Date

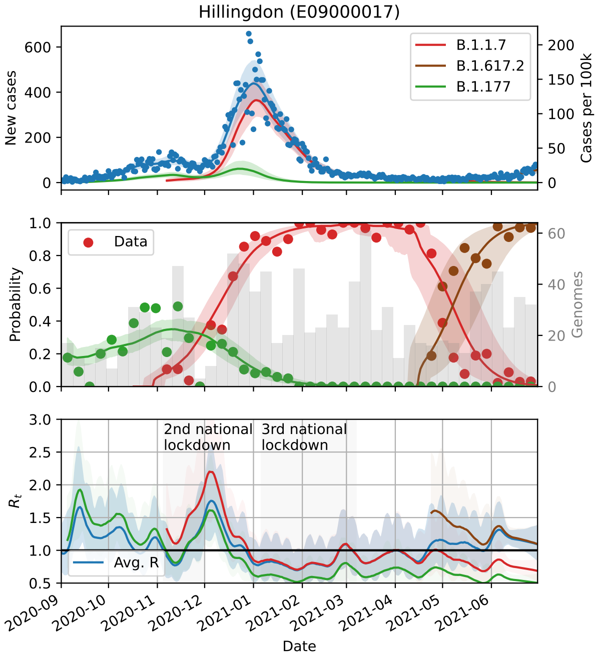
Hillingdon (E09000017)
New cases
Cases per 100k
B.1.1.7
B.1.617.2
B.1.177
Probability
Genomes
Data
R_t
2nd national lockdown
3rd national lockdown
Avg. R
Date

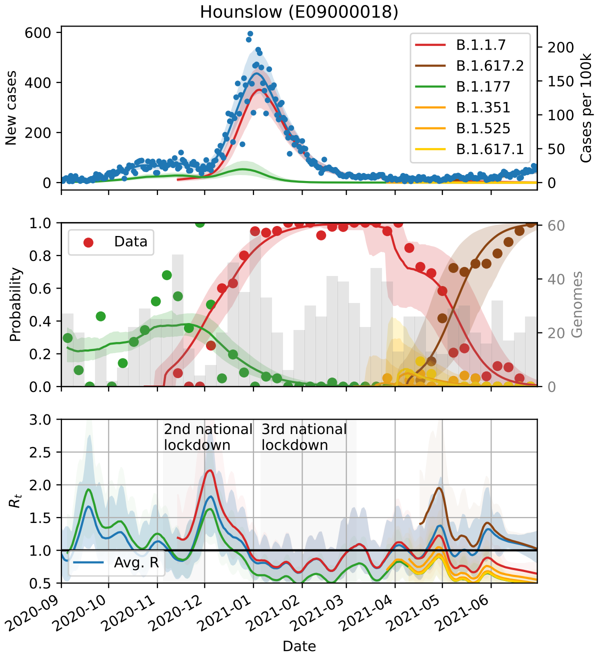
Hounslow (E09000018)
New cases
Cases per 100k
B.1.1.7
B.1.617.2
B.1.177
B.1.351
B.1.525
B.1.617.1
Probability
Genomes
Data
R_t
2nd national lockdown
3rd national lockdown
Avg. R
Date

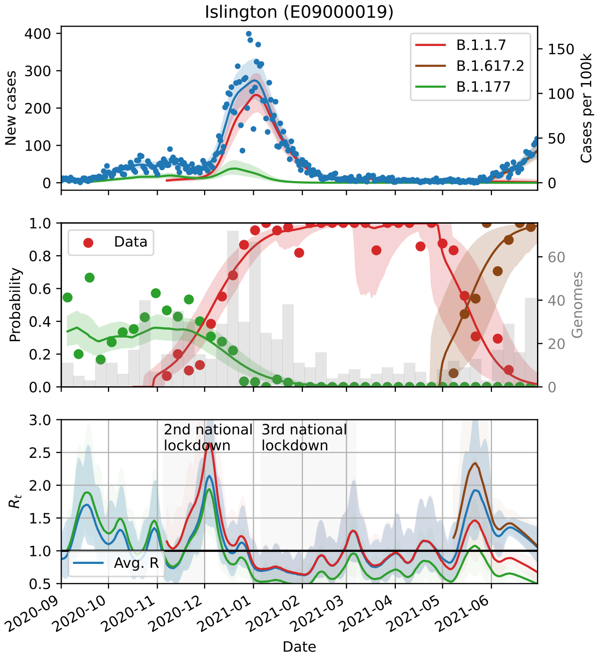
Islington (E09000019)
New cases
Cases per 100k
B.1.1.7
B.1.617.2
B.1.177
Probability
Genomes
Data
R_t
2nd national lockdown
3rd national lockdown
Avg. R
Date

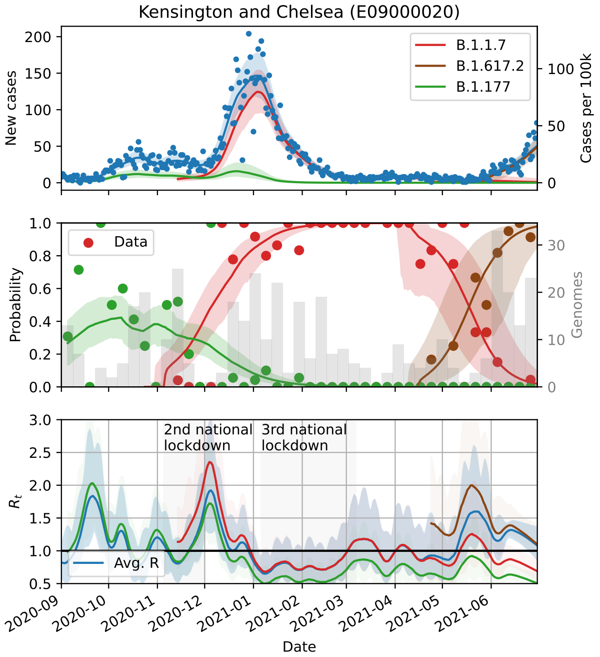
Kensington and Chelsea (E09000020)
New cases
Cases per 100k
B.1.1.7
B.1.617.2
B.1.177
Probability
Genomes
Data
R_t
2nd national lockdown
3rd national lockdown
Avg. R
Date

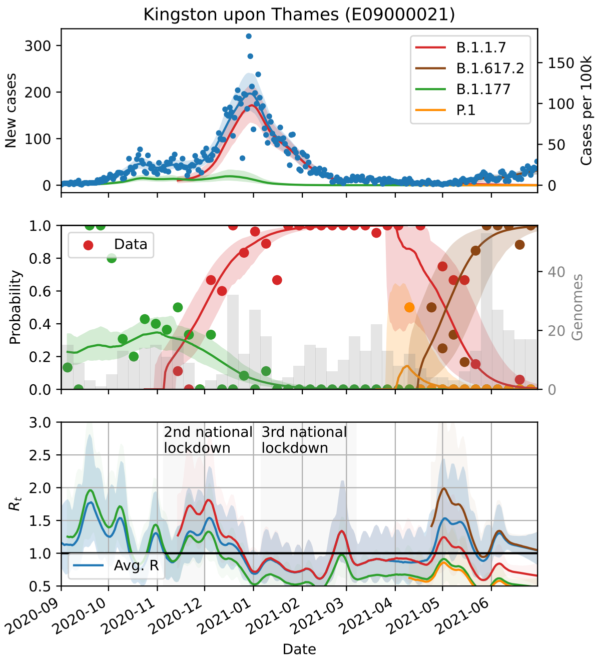
Kingston upon Thames (E09000021)
New cases
Cases per 100k
B.1.1.7
B.1.617.2
B.1.177
P.1
Data
Probability
Genomes
2nd national lockdown
3rd national lockdown
R_t
Avg. R
Date

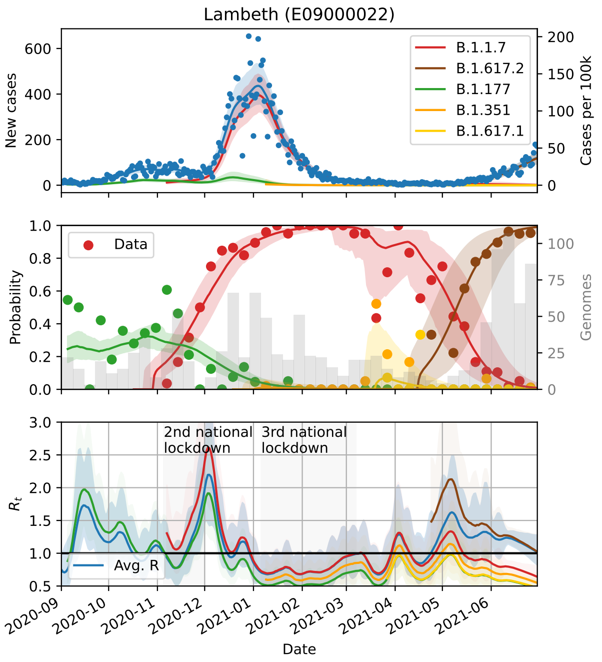
Lambeth (E09000022)
New cases
Cases per 100k
B.1.1.7
B.1.617.2
B.1.177
B.1.351
B.1.617.1
Data
Probability
Genomes
2nd national lockdown
3rd national lockdown
R_t
Avg. R
Date

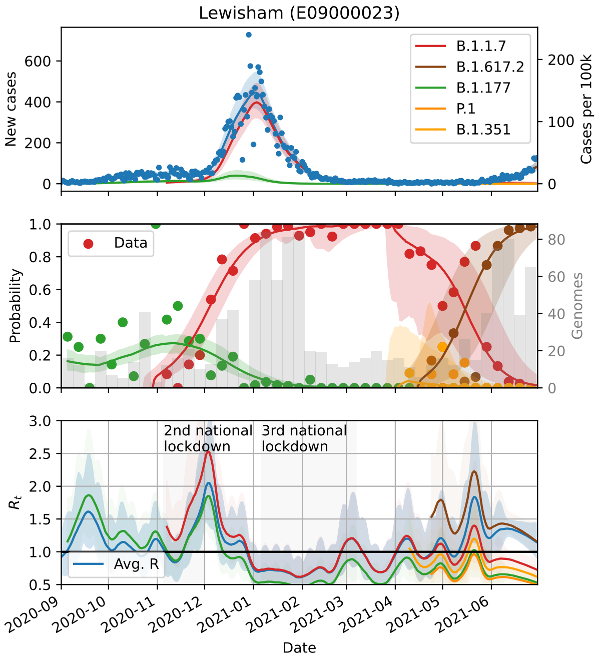
Lewisham (E09000023)
New cases
Cases per 100k
B.1.1.7
B.1.617.2
B.1.177
P.1
B.1.351
Data
Probability
Genomes
2nd national lockdown
3rd national lockdown
R_t
Avg. R
Date

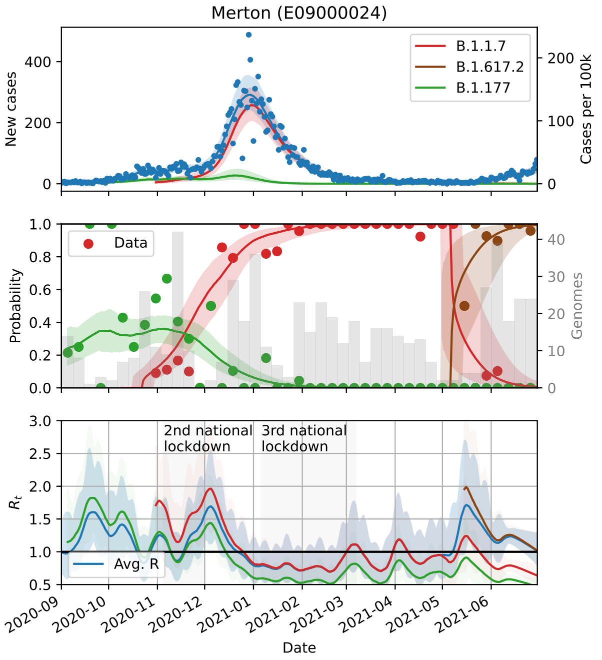
Merton (E09000024)
New cases
Cases per 100k
B.1.1.7
B.1.617.2
B.1.177
Data
Probability
Genomes
2nd national lockdown
3rd national lockdown
R_t
Avg. R
Date

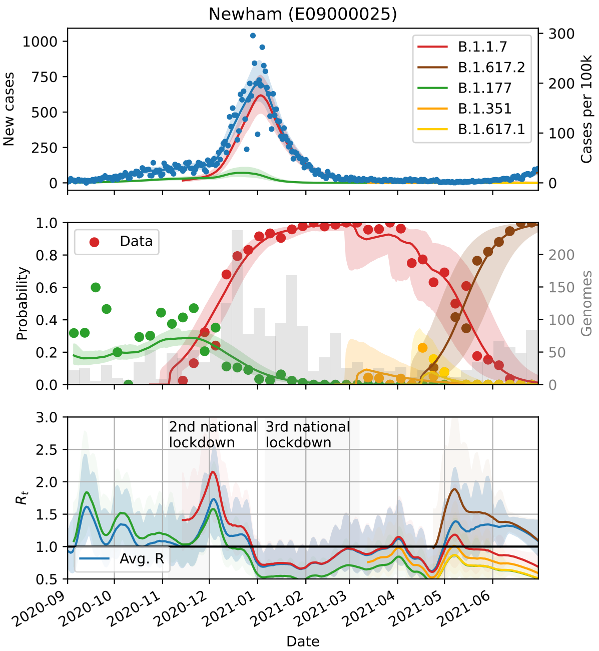

Newham (E09000025)
B.1.1.7
B.1.617.2
B.1.177
B.1.351
B.1.617.1
New cases
Cases per 100k
Data
Probability
Genomes
2nd national lockdown
3rd national lockdown
Avg. R
R_t
Date

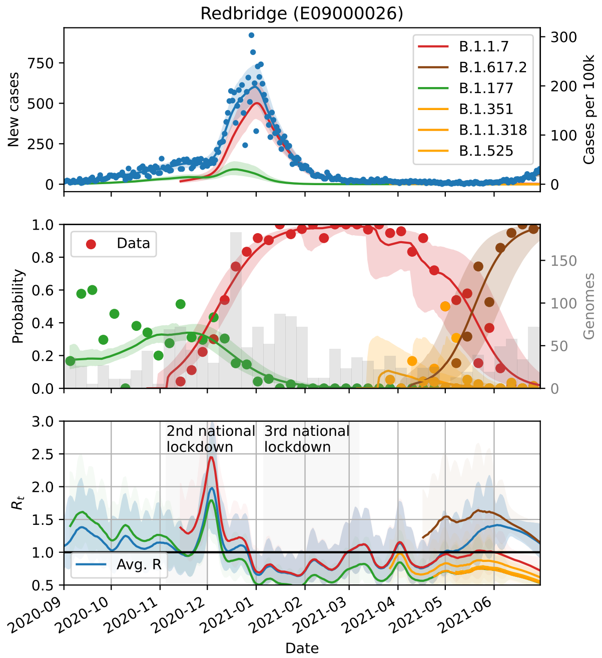

Redbridge (E09000026)
B.1.1.7
B.1.617.2
B.1.177
B.1.351
B.1.1.318
B.1.525
New cases
Cases per 100k
Data
Probability
Genomes
2nd national lockdown
3rd national lockdown
Avg. R
R_t
Date

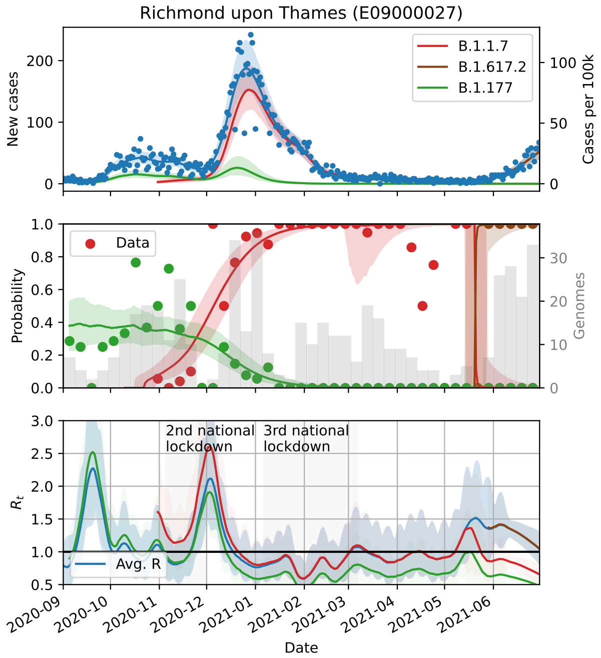

Richmond upon Thames (E09000027)
B.1.1.7
B.1.617.2
B.1.177
New cases
Cases per 100k
Data
Probability
Genomes
2nd national lockdown
3rd national lockdown
Avg. R
R_t
Date

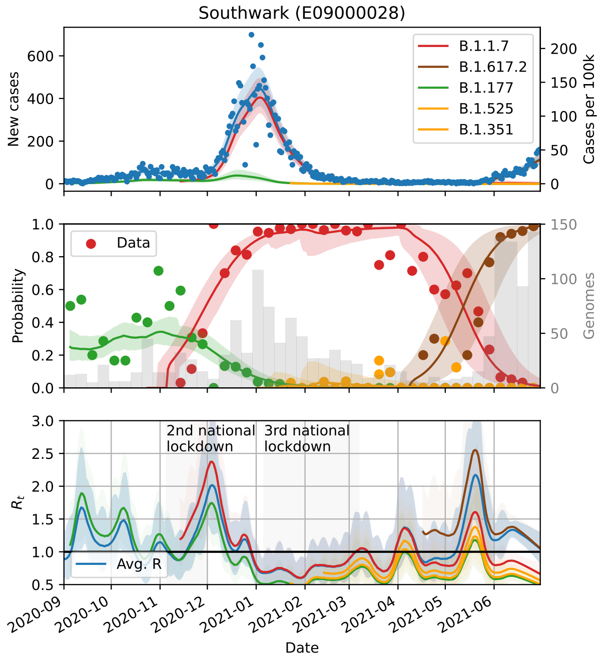

Southwark (E09000028)
B.1.1.7
B.1.617.2
B.1.177
B.1.525
B.1.351
New cases
Cases per 100k
Data
Probability
Genomes
2nd national lockdown
3rd national lockdown
Avg. R
R_t
Date

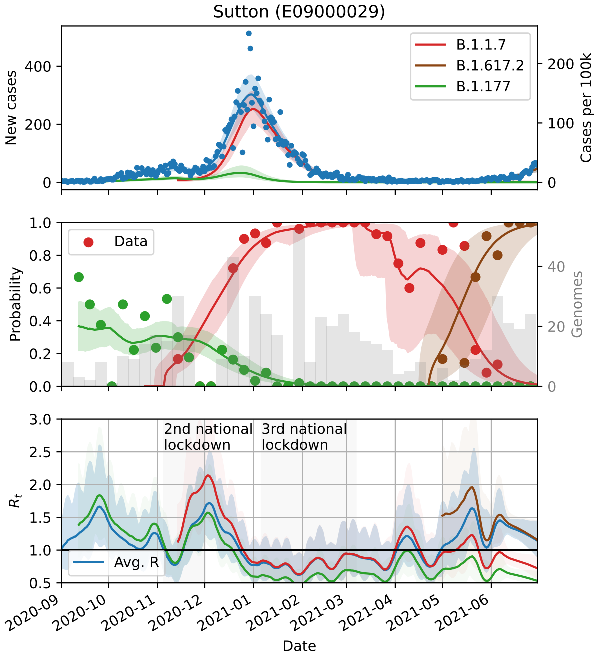
Sutton (E09000029)
New cases
Cases per 100k
B.1.1.7
B.1.617.2
B.1.177
Probability
Genomes
Data
R_t
2nd national lockdown
3rd national lockdown
Avg. R
Date

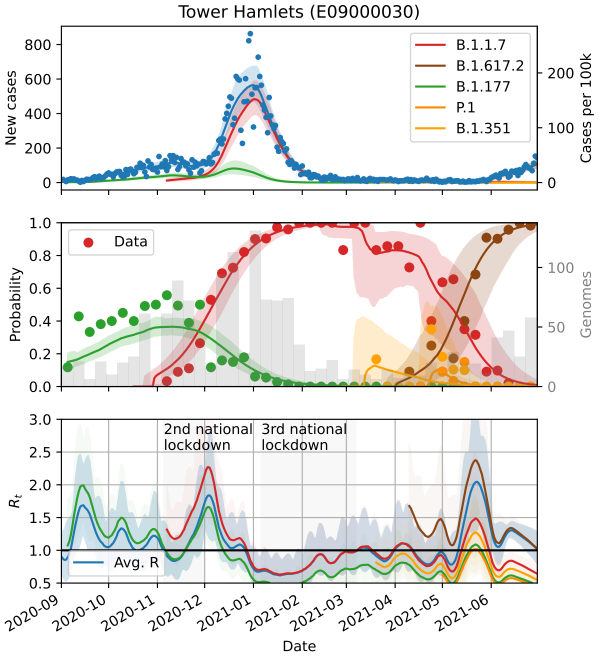
Tower Hamlets (E09000030)
New cases
Cases per 100k
B.1.1.7
B.1.617.2
B.1.177
P.1
B.1.351
Probability
Genomes
Data
R_t
2nd national lockdown
3rd national lockdown
Avg. R
Date

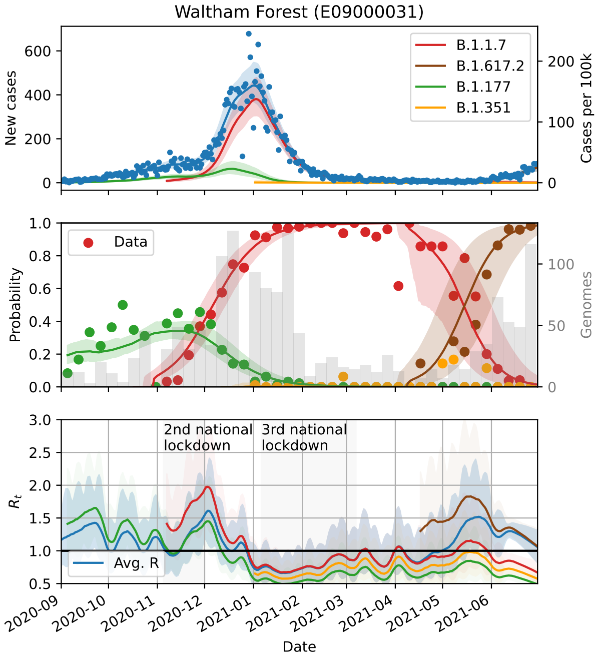
Waltham Forest (E09000031)
New cases
Cases per 100k
B.1.1.7
B.1.617.2
B.1.177
B.1.351
Probability
Genomes
Data
R_t
2nd national lockdown
3rd national lockdown
Avg. R
Date

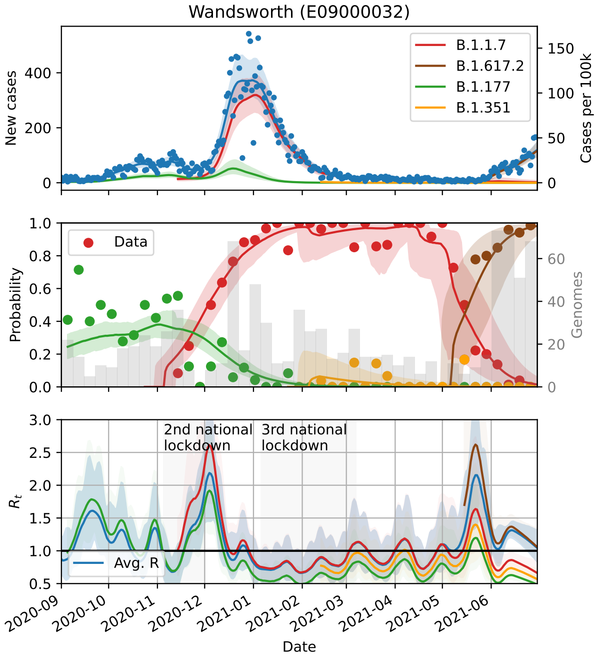
Wandsworth (E09000032)
New cases
Cases per 100k
B.1.1.7
B.1.617.2
B.1.177
B.1.351
Probability
Genomes
Data
R_t
2nd national lockdown
3rd national lockdown
Avg. R
Date

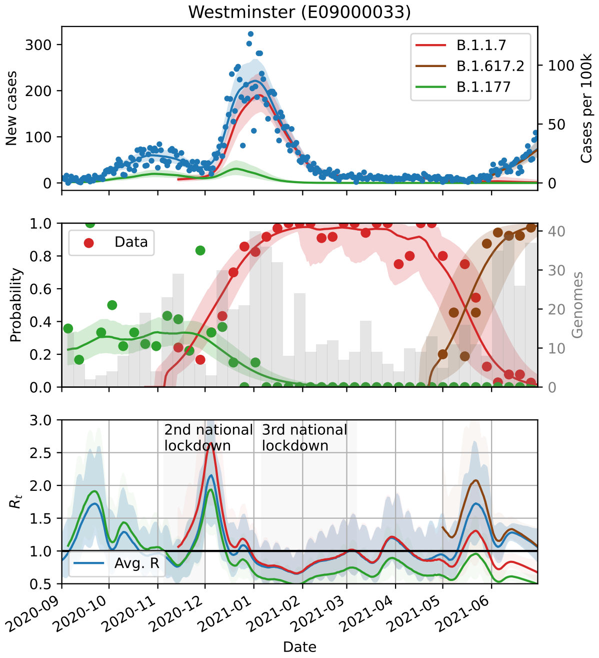
Westminster (E09000033)
New cases
Cases per 100k
B.1.1.7
B.1.617.2
B.1.177
Data
Probability
Genomes
2nd national lockdown
3rd national lockdown
R_t
Avg. R
2020-09
2020-10
2020-11
2020-12
2021-01
2021-02
2021-03
2021-04
2021-05
2021-06
Date

3 Supplementary Note 3

Supplementary Note 3 Tab. 1: **Lower Tier Local Authorities (LTLA) lookup codes and names.**

	LTLA look up code	LTLA name
0	E06000001	Hartlepool
1	E06000002	Middlesbrough
2	E06000003	Redcar and Cleveland
3	E06000004	Stockton-on-Tees
4	E06000005	Darlington
5	E06000006	Halton
6	E06000007	Warrington
7	E06000008	Blackburn with Darwen
8	E06000009	Blackpool
9	E06000010	Kingston upon Hull, City of
10	E06000011	East Riding of Yorkshire
11	E06000012	North East Lincolnshire
12	E06000013	North Lincolnshire
13	E06000014	York
14	E06000015	Derby
15	E06000016	Leicester
16	E06000017	Rutland
17	E06000018	Nottingham
18	E06000019	Herefordshire, County of
19	E06000020	Telford and Wrekin
20	E06000021	Stoke-on-Trent
21	E06000022	Bath and North East Somerset
22	E06000023	Bristol, City of
23	E06000024	North Somerset
24	E06000025	South Gloucestershire
25	E06000026	Plymouth
26	E06000027	Torbay
27	E06000030	Swindon
28	E06000031	Peterborough
29	E06000032	Luton
30	E06000033	Southend-on-Sea

Continued on next page

	LTLA look up code	LTLA name
31	E06000034	Thurrock
32	E06000035	Medway
33	E06000036	Bracknell Forest
34	E06000037	West Berkshire
35	E06000038	Reading
36	E06000039	Slough
37	E06000040	Windsor and Maidenhead
38	E06000041	Wokingham
39	E06000042	Milton Keynes
40	E06000043	Brighton and Hove
41	E06000044	Portsmouth
42	E06000045	Southampton
43	E06000046	Isle of Wight
44	E06000047	County Durham
45	E06000049	Cheshire East
46	E06000050	Cheshire West and Chester
47	E06000051	Shropshire
48	E06000052	Cornwall
49	E06000053	Isles of Scilly
50	E06000054	Wiltshire
51	E06000055	Bedford
52	E06000056	Central Bedfordshire
53	E06000057	Northumberland
54	E06000058	Bournemouth, Christchurch and Poole
55	E06000059	Dorset
56	E07000004	Aylesbury Vale
57	E07000005	Chiltern
58	E07000006	South Bucks
59	E07000007	Wycombe
60	E07000008	Cambridge
61	E07000009	East Cambridgeshire
62	E07000010	Fenland
63	E07000011	Huntingdonshire
64	E07000012	South Cambridgeshire
65	E07000026	Allerdale

Continued on next page

	LTLA look up code	LTLA name
66	E07000027	Barrow-in-Furness
67	E07000028	Carlisle
68	E07000029	Copeland
69	E07000030	Eden
70	E07000031	South Lakeland
71	E07000032	Amber Valley
72	E07000033	Bolsover
73	E07000034	Chesterfield
74	E07000035	Derbyshire Dales
75	E07000036	Erewash
76	E07000037	High Peak
77	E07000038	North East Derbyshire
78	E07000039	South Derbyshire
79	E07000040	East Devon
80	E07000041	Exeter
81	E07000042	Mid Devon
82	E07000043	North Devon
83	E07000044	South Hams
84	E07000045	Teignbridge
85	E07000046	Torridge
86	E07000047	West Devon
87	E07000061	Eastbourne
88	E07000062	Hastings
89	E07000063	Lewes
90	E07000064	Rother
91	E07000065	Wealden
92	E07000066	Basildon
93	E07000067	Braintree
94	E07000068	Brentwood
95	E07000069	Castle Point
96	E07000070	Chelmsford
97	E07000071	Colchester
98	E07000072	Epping Forest
99	E07000073	Harlow
100	E07000074	Maldon

Continued on next page

	LTLA look up code	LTLA name
101	E07000075	Rochford
102	E07000076	Tendring
103	E07000077	Uttlesford
104	E07000078	Cheltenham
105	E07000079	Cotswold
106	E07000080	Forest of Dean
107	E07000081	Gloucester
108	E07000082	Stroud
109	E07000083	Tewkesbury
110	E07000084	Basingstoke and Deane
111	E07000085	East Hampshire
112	E07000086	Eastleigh
113	E07000087	Fareham
114	E07000088	Gosport
115	E07000089	Hart
116	E07000090	Havant
117	E07000091	New Forest
118	E07000092	Rushmoor
119	E07000093	Test Valley
120	E07000094	Winchester
121	E07000095	Broxbourne
122	E07000096	Dacorum
123	E07000098	Hertsmere
124	E07000099	North Hertfordshire
125	E07000102	Three Rivers
126	E07000103	Watford
127	E07000105	Ashford
128	E07000106	Canterbury
129	E07000107	Dartford
130	E07000108	Dover
131	E07000109	Gravesham
132	E07000110	Maidstone
133	E07000111	Sevenoaks
134	E07000112	Folkestone and Hythe
135	E07000113	Swale

Continued on next page

	LTLA look up code	LTLA name
136	E07000114	Thanet
137	E07000115	Tonbridge and Malling
138	E07000116	Tunbridge Wells
139	E07000117	Burnley
140	E07000118	Chorley
141	E07000119	Fylde
142	E07000120	Hyndburn
143	E07000121	Lancaster
144	E07000122	Pendle
145	E07000123	Preston
146	E07000124	Ribble Valley
147	E07000125	Rossendale
148	E07000126	South Ribble
149	E07000127	West Lancashire
150	E07000128	Wyre
151	E07000129	Blaby
152	E07000130	Charnwood
153	E07000131	Harborough
154	E07000132	Hinckley and Bosworth
155	E07000133	Melton
156	E07000134	North West Leicestershire
157	E07000135	Oadby and Wigston
158	E07000136	Boston
159	E07000137	East Lindsey
160	E07000138	Lincoln
161	E07000139	North Kesteven
162	E07000140	South Holland
163	E07000141	South Kesteven
164	E07000142	West Lindsey
165	E07000143	Breckland
166	E07000144	Broadland
167	E07000145	Great Yarmouth
168	E07000146	King's Lynn and West Norfolk
169	E07000147	North Norfolk
170	E07000148	Norwich

Continued on next page

	LTLA look up code	LTLA name
171	E07000149	South Norfolk
172	E07000150	Corby
173	E07000151	Daventry
174	E07000152	East Northamptonshire
175	E07000153	Kettering
176	E07000154	Northampton
177	E07000155	South Northamptonshire
178	E07000156	Wellingborough
179	E07000163	Craven
180	E07000164	Hambleton
181	E07000165	Harrogate
182	E07000166	Richmondshire
183	E07000167	Ryedale
184	E07000168	Scarborough
185	E07000169	Selby
186	E07000170	Ashfield
187	E07000171	Bassetlaw
188	E07000172	Broxtowe
189	E07000173	Gedling
190	E07000174	Mansfield
191	E07000175	Newark and Sherwood
192	E07000176	Rushcliffe
193	E07000177	Cherwell
194	E07000178	Oxford
195	E07000179	South Oxfordshire
196	E07000180	Vale of White Horse
197	E07000181	West Oxfordshire
198	E07000187	Mendip
199	E07000188	Sedgemoor
200	E07000189	South Somerset
201	E07000192	Cannock Chase
202	E07000193	East Staffordshire
203	E07000194	Lichfield
204	E07000195	Newcastle-under-Lyme
205	E07000196	South Staffordshire

Continued on next page

	LTLA look up code	LTLA name
206	E07000197	Stafford
207	E07000198	Staffordshire Moorlands
208	E07000199	Tamworth
209	E07000200	Babergh
210	E07000202	Ipswich
211	E07000203	Mid Suffolk
212	E07000207	Elmbridge
213	E07000208	Epsom and Ewell
214	E07000209	Guildford
215	E07000210	Mole Valley
216	E07000211	Reigate and Banstead
217	E07000212	Runnymede
218	E07000213	Spelthorne
219	E07000214	Surrey Heath
220	E07000215	Tandridge
221	E07000216	Waverley
222	E07000217	Woking
223	E07000218	North Warwickshire
224	E07000219	Nuneaton and Bedworth
225	E07000220	Rugby
226	E07000221	Stratford-on-Avon
227	E07000222	Warwick
228	E07000223	Adur
229	E07000224	Arun
230	E07000225	Chichester
231	E07000226	Crawley
232	E07000227	Horsham
233	E07000228	Mid Sussex
234	E07000229	Worthing
235	E07000234	Bromsgrove
236	E07000235	Malvern Hills
237	E07000236	Redditch
238	E07000237	Worcester
239	E07000238	Wychavon
240	E07000239	Wyre Forest

Continued on next page

	LTLA look up code	LTLA name
241	E07000240	St Albans
242	E07000241	Welwyn Hatfield
243	E07000242	East Hertfordshire
244	E07000243	Stevenage
245	E07000244	East Suffolk
246	E07000245	West Suffolk
247	E07000246	Somerset West and Taunton
248	E08000001	Bolton
249	E08000002	Bury
250	E08000003	Manchester
251	E08000004	Oldham
252	E08000005	Rochdale
253	E08000006	Salford
254	E08000007	Stockport
255	E08000008	Tameside
256	E08000009	Trafford
257	E08000010	Wigan
258	E08000011	Knowsley
259	E08000012	Liverpool
260	E08000013	St. Helens
261	E08000014	Sefton
262	E08000015	Wirral
263	E08000016	Barnsley
264	E08000017	Doncaster
265	E08000018	Rotherham
266	E08000019	Sheffield
267	E08000021	Newcastle upon Tyne
268	E08000022	North Tyneside
269	E08000023	South Tyneside
270	E08000024	Sunderland
271	E08000025	Birmingham
272	E08000026	Coventry
273	E08000027	Dudley
274	E08000028	Sandwell
275	E08000029	Solihull

Continued on next page

	LTLA look up code	LTLA name
276	E08000030	Walsall
277	E08000031	Wolverhampton
278	E08000032	Bradford
279	E08000033	Calderdale
280	E08000034	Kirklees
281	E08000035	Leeds
282	E08000036	Wakefield
283	E08000037	Gateshead
284	E09000001	City of London
285	E09000002	Barking and Dagenham
286	E09000003	Barnet
287	E09000004	Bexley
288	E09000005	Brent
289	E09000006	Bromley
290	E09000007	Camden
291	E09000008	Croydon
292	E09000009	Ealing
293	E09000010	Enfield
294	E09000011	Greenwich
295	E09000012	Hackney
296	E09000013	Hammersmith and Fulham
297	E09000014	Haringey
298	E09000015	Harrow
299	E09000016	Havering
300	E09000017	Hillingdon
301	E09000018	Hounslow
302	E09000019	Islington
303	E09000020	Kensington and Chelsea
304	E09000021	Kingston upon Thames
305	E09000022	Lambeth
306	E09000023	Lewisham
307	E09000024	Merton
308	E09000025	Newham
309	E09000026	Redbridge
310	E09000027	Richmond upon Thames

Continued on next page

	LTLA look up code	LTLA name
311	E09000028	Southwark
312	E09000029	Sutton
313	E09000030	Tower Hamlets
314	E09000031	Waltham Forest
315	E09000032	Wandsworth
316	E09000033	Westminster
