# Supplementary material for: Genomic reconstruction of the SARS-CoV-2 epidemic in England
Source: Nature. 2021 Oct 14;600(7889):506–11. doi: 10.1038/s41586-021-04069-y (PMC8674138; doi:10.1038/s41586-021-04069-y)
Supplement: Supplementary file 2 — Reporting Summary [file 41586_2021_4069_MOESM2_ESM.pdf]

nature research

Corresponding author(s): Moritz Gerstung

Last updated by author(s): Aug 27, 2021

Reporting Summary

Nature Research wishes to improve the reproducibility of the work that we publish. This form provides structure for consistency and transparency in reporting. For further information on Nature Research policies, see our Editorial Policies and the Editorial Policy Checklist.

Statistics

For all statistical analyses, confirm that the following items are present in the figure legend, table legend, main text, or Methods section.

n/a	Confirmed	
☐	☒	The exact sample size (*n*) for each experimental group/condition, given as a discrete number and unit of measurement
☒	☐	A statement on whether measurements were taken from distinct samples or whether the same sample was measured repeatedly
☒	☐	The statistical test(s) used AND whether they are one- or two-sided<br>*Only common tests should be described solely by name; describe more complex techniques in the Methods section.*
☒	☐	A description of all covariates tested
☐	☒	A description of any assumptions or corrections, such as tests of normality and adjustment for multiple comparisons
☐	☒	A full description of the statistical parameters including central tendency (e.g. means) or other basic estimates (e.g. regression coefficient) AND variation (e.g. standard deviation) or associated estimates of uncertainty (e.g. confidence intervals)
☒	☐	For null hypothesis testing, the test statistic (e.g. *F*, *t*, *r*) with confidence intervals, effect sizes, degrees of freedom and *P* value noted<br>*Give P values as exact values whenever suitable.*
☐	☒	For Bayesian analysis, information on the choice of priors and Markov chain Monte Carlo settings
☐	☒	For hierarchical and complex designs, identification of the appropriate level for tests and full reporting of outcomes
☒	☐	Estimates of effect sizes (e.g. Cohen's *d*, Pearson's *r*), indicating how they were calculated

Our web collection on statistics for biologists contains articles on many of the points above.

Software and code

Policy information about availability of computer code

Data collection	Consensus Fasta sequences were created using the ARTIC nextflow processing pipeline and SARS-CoV-2 lineage assignments using the Pangolin software (01-04-2021 and 23-04-2021 version) and FastTree2 (2.1.11).
Data analysis	Code for spatio-temporal modeling of viral lineages is available at https://github.com/gerstung-lab/genomicsurveillance and as a PyPI package (genomicsurveillance). Analyses were performed in Python 3.7.1 (Packages: matplotlib (3.4.1), numpy (1.20.2), pandas (1.2.3), scikit-learn (0.19.1), scipy (1.6.2), seaborn (0.11.1), jax (0.2.8), genomicsurveillance (0.4.0), numpyro (0.4.0)). The phylogeographic analyses were performed using Thorney Beast (0.1.1) and https://github.com/NicolaDM/phylogeographySARS-CoV-2. Code for ONS infection survey analysis is available at https://github.com/jhellewell14/ons_severity_estimates.

For manuscripts utilizing custom algorithms or software that are central to the research but not yet described in published literature, software must be made available to editors and reviewers. We strongly encourage code deposition in a community repository (e.g. GitHub). See the Nature Research guidelines for submitting code & software for further information.

Data

Policy information about availability of data

All manuscripts must include a data availability statement. This statement should provide the following information, where applicable:
- Accession codes, unique identifiers, or web links for publicly available datasets
- A list of figures that have associated raw data
- A description of any restrictions on data availability

PCR test data are publicly available at https://coronavirus.data.gov.uk/.
SARS-CoV-2 genome data and geolocations can be obtained under controlled access from https://www.cogconsortium.uk/data/.

A filtered, privacy conserving version of the data set is publicly available at https://covid19.sanger.ac.uk/downloads.
The data and a version of the analysis with fewer lineages can be interactively explored at https://covid19.sanger.ac.uk.

Field-specific reporting

Please select the one below that is the best fit for your research. If you are not sure, read the appropriate sections before making your selection.

☒ Life sciences ☐ Behavioural & social sciences ☐ Ecological, evolutionary & environmental sciences

For a reference copy of the document with all sections, see nature.com/documents/nr-reporting-summary-flat.pdf

Life sciences study design

All studies must disclose on these points even when the disclosure is negative.

Sample size	The study is based on data from 281,178 viral genomes and 3,894,234 positive PCR tests collected in England during the time period from September 1, 2020 to June 30, 2021. This is an observational study based on an existing data set compiled by COG-UK, therefore no sample size calculation is applicable.
Data exclusions	No data was excluded in the analysis.
Replication	This is an observational study based on an existing data set compiled by COG-UK, therefore no replication is applicable.
Randomization	This is an observational study based on an existing data set compiled by COG-UK, therefore no randomization is applicable.
Blinding	This is an observational study based on an existing data set compiled by COG-UK, therefore no blinding is applicable.

Reporting for specific materials, systems and methods

We require information from authors about some types of materials, experimental systems and methods used in many studies. Here, indicate whether each material, system or method listed is relevant to your study. If you are not sure if a list item applies to your research, read the appropriate section before selecting a response.

Materials & experimental systems

n/a	Involved in the study
☒	☐ Antibodies
☒	☐ Eukaryotic cell lines
☒	☐ Palaeontology and archaeology
☒	☐ Animals and other organisms
☒	☐ Human research participants
☒	☐ Clinical data
☒	☐ Dual use research of concern

Methods

n/a	Involved in the study
☒	☐ ChIP-seq
☒	☐ Flow cytometry
☒	☐ MRI-based neuroimaging
